# Synthesis of Polycyclic Fused Indoline Scaffolds through a Substrate-Guided Reactivity Switch

**DOI:** 10.1021/acs.joc.0c01489

**Published:** 2020-08-12

**Authors:** Cecilia Ciccolini, Giacomo Mari, Francesco G. Gatti, Giuseppe Gatti, Gianluca Giorgi, Fabio Mantellini, Gianfranco Favi

**Affiliations:** † Department of Biomolecular Sciences, Section of Chemistry and Pharmaceutical Technologies, University of Urbino “Carlo Bo”, Via I Maggetti 24, 61029 Urbino, Italy; ‡ Department of Chemistry, Materials and Chemical Engineering “G. Natta”, Piazza Leonardo da Vinci 32, 20133 Milano, Italy; § Department of Biotechnologies, Chemistry & Pharmacy, University of Siena, Via A. Moro 2, 53100 Siena, Italy

## Abstract

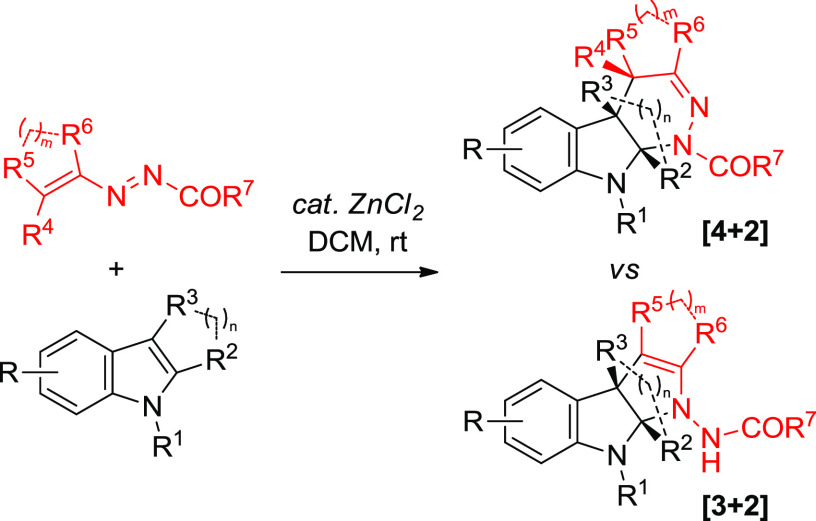

Zn(II)-catalyzed divergent synthesis of functionalized polycyclic indolines through formal [3 + 2] and [4 + 2] cycloadditions of indoles with 1,2-diaza-1,3-dienes (DDs) is reported. The nature and type of substituents of substrates are found to act as a chemical switch to trigger two distinct reaction pathways and to obtain two different types of products upon the influence of the same catalyst. The mechanism of both [4 + 2] and [3 + 2] cycloadditions was investigated and fully rationalized by density functional theory (DFT) calculations.

## Introduction

**Figure 1:**
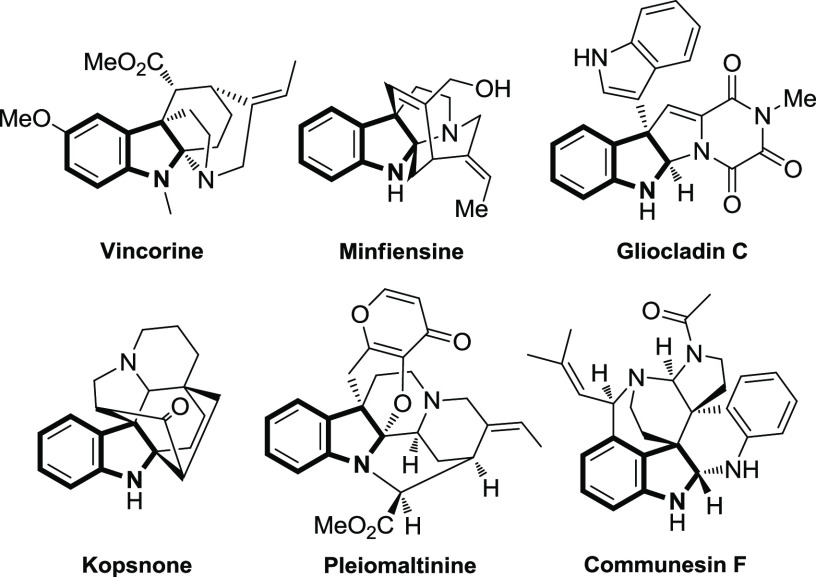
Examples of naturally occurring compounds containing 2,3-fused indolines.

Functionalized polycyclic fused indoline frameworks are central molecular architectures in nature and pharmaceuticals.^1^ As one of the indolines, C2,C3-fused indolines^2^ have attracted extensive research effort over the past decades because scaffolds of this type lead to relatively rigid structures that might be expected to show substantial selectivity in their interactions with enzymes or receptors.^3^ Representative naturally occurring polycyclic indolines such as vincorine, minfiensine, gliocladin C, kopsnone, pleiomaltinine, and communesin F are shown in Figure 1.

Among the annelated indolines, the pyrroloindoline, pyridazino indoline skeletons and their related structures, can be found in numerous natural bioactive products, marketed drugs, and other functional molecules.^4,5^ The desire to build such appealing polycyclic frameworks, particularly those with bridgehead amino acetal C2 carbons, has inspired the development of elegant methodologies over the past several years. Among the reported methods, dearomatization of indoles *via* cycloaddition reactions^6^ has been demonstrated as a reliable approach in converting simple planar aromatic molecules into structurally complex and stereoselective ring systems.

Following the initial discovery of the inverse electron-demand [4 + 2] cycloaddition reaction of electron-rich alkenes (furans, pyrroles, and indoles) with 1,2-diaza-1,3-dienes (DDs) by Gilchrist et al.,^7^ other elegant studies by the groups of Wang^8^ and Tan^9^ have been recently reported exploiting indoles as nucleophiles.

**Scheme 1:**
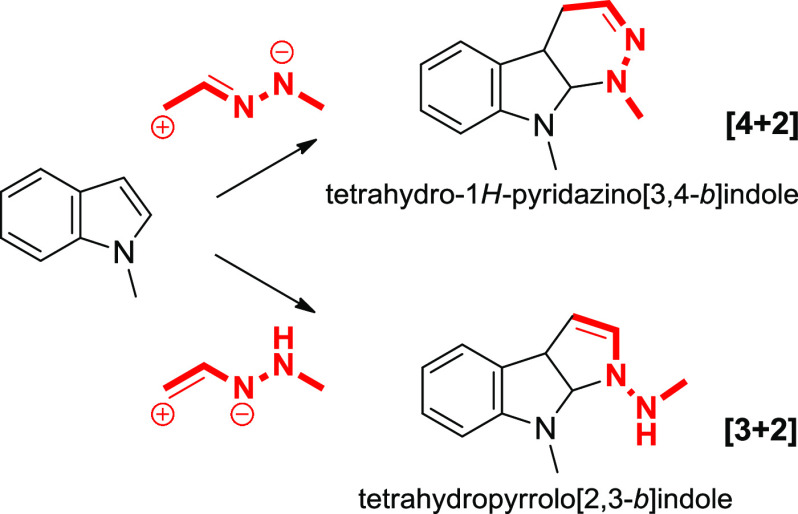
Working Hypothesis: Chemodivergent Synthesis of Polycyclic Fused Indoline Scaffolds

By taking advantage of the unique reactivity of DDs^10^ and intrigued by these and our recent findings in the manipulation of indolyl cores,^11^ we reasoned that the proper combination of indole and 1,2-diaza-1,3-diene elements might allow us to design a substrate-controlled divergent approach. In this design, DDs would be used as C2N1 or C2N2 units (1,3 or 1,4 dipole synthons) to realize [3 + 2] and [4 + 2] annulation reactions of indoles, respectively (Scheme 1). Thus, by tuning the substituents of both substrates upon the influence of the same catalyst, two series of fused indoline-based scaffolds such as tetrahydro-1*H*-pyridazino[3,4-*b*]indoles and tetrahydropyrrolo[2,3-*b*]indoles would be generated with chemodivergence.

Distinct from previous findings, we herein report our successful development of a substituent-controlled divergent synthesis of fused indoline-based scaffolds. These [4 + 2] and [3 + 2] cycloadditions were realized in a straightforward, pretty challenging, and highly atom-economical/diastereselective manner from rationally designed indole and 1,2-diaza-1,3-diene substrates with C3 and/or C4 position(s) substituted, respectively.

## Results and Discussion

We began our work by studying the reaction between indole **1a** and cyclic 1,2-diaza-1,3-diene **2a** (Table S1, Supporting Information (SI)). No reaction took place, and both compounds remained inactive in the absence of a Lewis acid catalyst. A series of Lewis acid catalysts [such as Sc(OTf)_3_, Zn(OAc)_2_, ZnSO_4_, Zn(OTf)_2_, SmCl_3_·6H_2_O, LiClO_4_, LiCl, CuCl_2_, Cu(OTf)_2_, CuBr_2_, InBr_3_, ZnBr_2_, and ZnCl_2_] and solvents [such as dichloromethane (DCM), acetone, tetrahydrofuran, acetonitrile, and cyclohexane] were examined, and the combination of ZnCl_2_ and CH_2_Cl_2_ (heterogeneous catalytic system) was found to be superior for this transformation. Noteworthy, compound **3a** was obtained as a single regio- and diastereoisomer (50% yield).

The substrate scope with respect to various 2,3-unsubstituted indoles **1a**–**n** and cyclic DDs **2a**–**h** (see the SI for details) was then examined under the optimized reaction conditions, and a variety of tetrahydro-1*H*-pyridazino[3,4-*b*]indoles (tetracyclic fused ring (6-5-6-6/7/8) systems) **3a**–**x** was synthesized (Table 1). As shown in Table 1, indoles **1a**–**n** with different electronic characters were suitable for the reaction, with six-membered cyclic DDs giving the relative fused indoline heterocycles **3a**–**d** in moderate to good yields. The Zn-catalyzed [4 + 2] cycloaddition reactions were further extended to seven- and eight-membered cyclic DDs. We were glad to find that the use of seven-membered DDs gave rise to the best results in terms of isolated yields. Also, the wide functional group tolerance was well demonstrated by the fact that both electron-donating (5-OMe, 5-, 7-Me) and electron-withdrawing (6-Cl, 5-CO_2_Me, 5-CN, 5-CHO, 5-NO_2_) groups were well tolerated, providing efficient access to the fused indoline heterocycles **3e**–**s**. Interestingly, the use of the 7-azaindole substrate also worked well to give the product **3t** in 85% isolated yield. The formal [4 + 2] annulation was then extended to DDs bearing cyclooctane, and the reactions furnished the relative products **3u**–**x** with lower yields than those of seven-membered cyclic DDs. Additionally, the generality of the N-terminal protective group on DDs as well as for the N atom of indoles was explored. Remarkably, free *N*–H indoles were also compatible with this protocol, albeit slightly lower yields were observed, probably owing to the reduced nucleophilicity at C3 and the reduced electrophilicity at C2 of the starting indole (Scheme 1, **3s***vs***3p**, and **3x***vs***3u**).

**Table 1:**
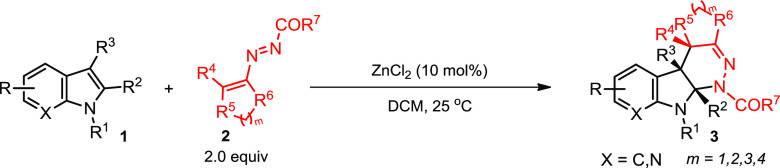

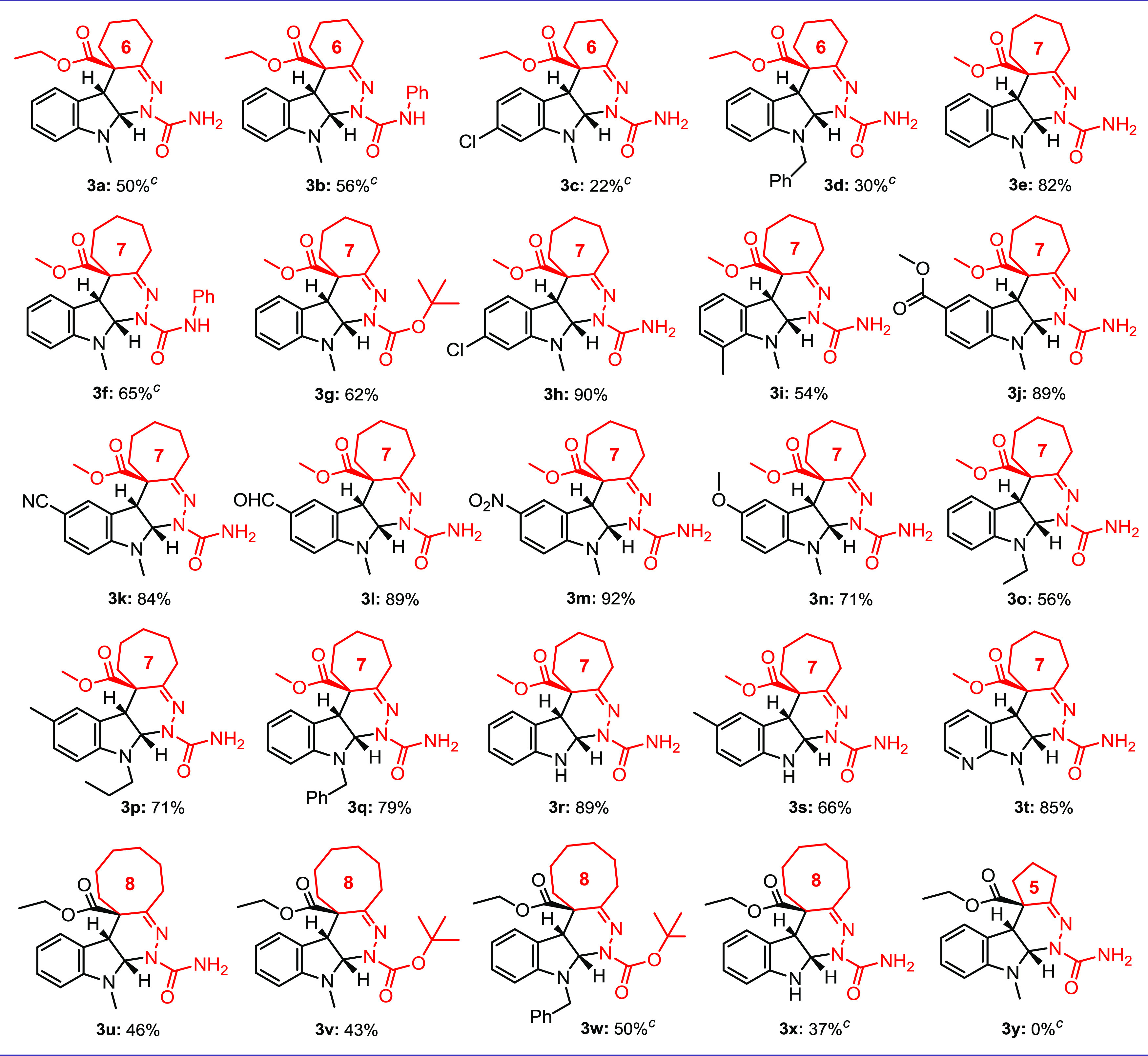
Scope of the Zn(II)-Catalyzed [4 + 2] Cycloaddition Reaction of 2,3-Unsubstituted Indoles (**1**) and Cyclic Azoalkenes (**2**)^a^^,^^b^

a Reaction conditions: **1** (2.0 mmol), **2** (1.0 mmol), ZnCl_2_ (0.1 mmol, 10 mol %), DCM (2.0 mL), 25 °C.

b Isolated yields.

c Ring-opened product **4** was also isolated.

No annulation occurred when five-membered cyclic DD was employed under the optimized reaction conditions (**3y**, 0%).^12^ The relative configurations of cycloadducts **3** were determined by X-ray diffraction analysis of **3e**(13) (see the SI for detailed X-ray crystallography data), and those of other compounds were assigned by analogy.

**Scheme 2:**
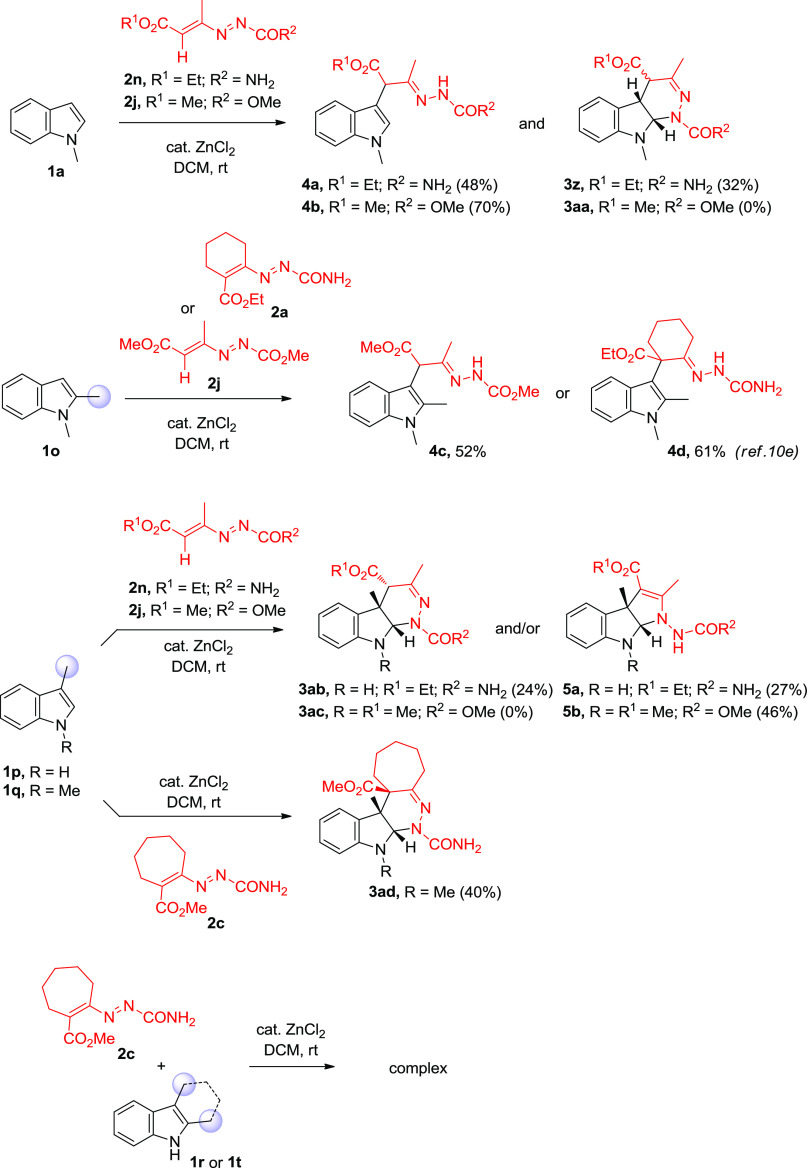
Other Substrates Scope Studies

During the investigation on the ring size effect of the 1,2-diaza-1,3-diene substrate, it was also noted the formation of ring-opened [4 + 2] byproduct **4**, highlighting the ease of rearomatization of **3** to give a more stable indole derivative. The sensitivity of **3** to the rearomatization process was confirmed by complete transformation of **3b** into **4e** in the presence of Amberlyst 15(H) (*vide infra*, Scheme 4b). This undesirable event appears to be the cause for lowering the [4 + 2] cycloaddition product yields found in some cases. Notably, this pathway remains dominant when the reaction was conducted using *N-*methyl indole (**1a**) or 1,2-dimethyl indole (**1o**) with linear DDs **2j** and **2n** (Scheme 2) in line with what was previously observed in the reactions of 2,3- (and 3-)unsubstituted indoles with cyclic and noncyclic DDs.^7a,10e^

More precisely, the reaction of *N*-methyl indole (**1a**) with linear DD **2n** afforded the more polar ring-opened [4 + 2] product **4a** (48% yield). However, thin-layer chromatography (TLC) analysis revealed the presence of a mixture of the diastereoisomers of pyridazine **3z**. Consistent with Gilchrist’s observation,^7b^ monitoring the progress of the reaction by ^1^H NMR, we detected an initial (preferential) formation of (*cis*,*cis*)*-***3z**, which then partially isomerized to its isomer (*cis*,*trans*)*-***3z** either during the course of the reaction or during chromatographic separation. Despite the isomerization side reaction, both diastereoisomers were isolated ((*cis*,*cis*)/(*cis*,*trans*) ∼ 2:1, 32% combined yield) and characterized (see the SI for details). On the other hand, the reaction of *N*-methyl indole (**1a**) with DD **2j** or 1,2-dimethyl indole (**1o**) with DD **2j** or **2a** led to the formation of the sole ring-opened [4 + 2] products **4b**–**d** (Scheme 2). Therefore, given the results with the use of both 2,3- and 3-unsubstituted indoles (associated with the [4 + 2] pyridazine-ring-opening reaction) and to further showcase the flexibility of this catalytic annulation strategy, we next moved our attention to exploring the reactivity of C3-blocked indoles (*e.g*., 3-substituted and 2,3-disubstituted indoles) with DDs. To our surprise, the reaction of 3-methyl indole (**1p**) with linear DD **2n** led to a mixture of two cycloadducts, the expected tetrahydro-1*H*-pyridazino[3,4-*b*]indole compound **3ab** and the tetrahydropyrrolo[2,3-*b*]indole compound **5a**(14) in a ratio of approximately 1:1, which could possibly be the result of the above-mentioned two competitive reaction pathways^15^ (Scheme 2). Interestingly, when 1,3-dimethyl indole (**1q**) was used in combination with DD **2j**, the exclusive formation of product **5b** (46% yield) was detected. As expected, when the reaction was repeated using cyclic DD **2c**, the exclusive formation of the corresponding [4 + 2] product **3ad** (40% yield) (Scheme 2) was observed. Intrigued by the starkly different reaction profile, we next focused our attention on the 2,3-disubstituted indole motif. Unfortunately, the reactions of 2,3-disubstituted indoles such as 2,3-dimethyl indole **1r** and 2,3,4,9-tetrahydro-1*H*-carbazole **1t** with cyclic DD such as **2c** did not work well, and only a trace amount of the respective formal [4 + 2] cycloaddition product was detected in the complex crude reaction mixture (Scheme 2). Explanations for these findings are not immediately intuited, but the steric effect seems to be playing a major role.

To our pleasure, the reaction of 2,3-dimethyl indole (**1r**) with DD **2j** proved efficient, leading to the relative [3 + 2] cycloadduct **5c** (58% yield) as the sole product. Thus, to further extend the substrate scope, a series of differently 2,3-disubstituted indole entities **1r**–**z** containing electron-donating groups (5-OMe and 5-Me) or electron-withdrawing groups (EWGs) (5-Cl) and 4-ester, 4-amide, or 4-phosphonate N-protected linear DDs **2j**–**s** were tested. Pleasantly, all of the reactions proceeded smoothly and furnished the highly crowded tetrahydropyrrolo[2,3-*b*]indole products **5c**–**s** in good to excellent yields (Table 2).

**Table 2:**
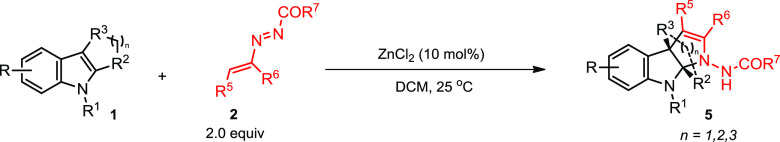

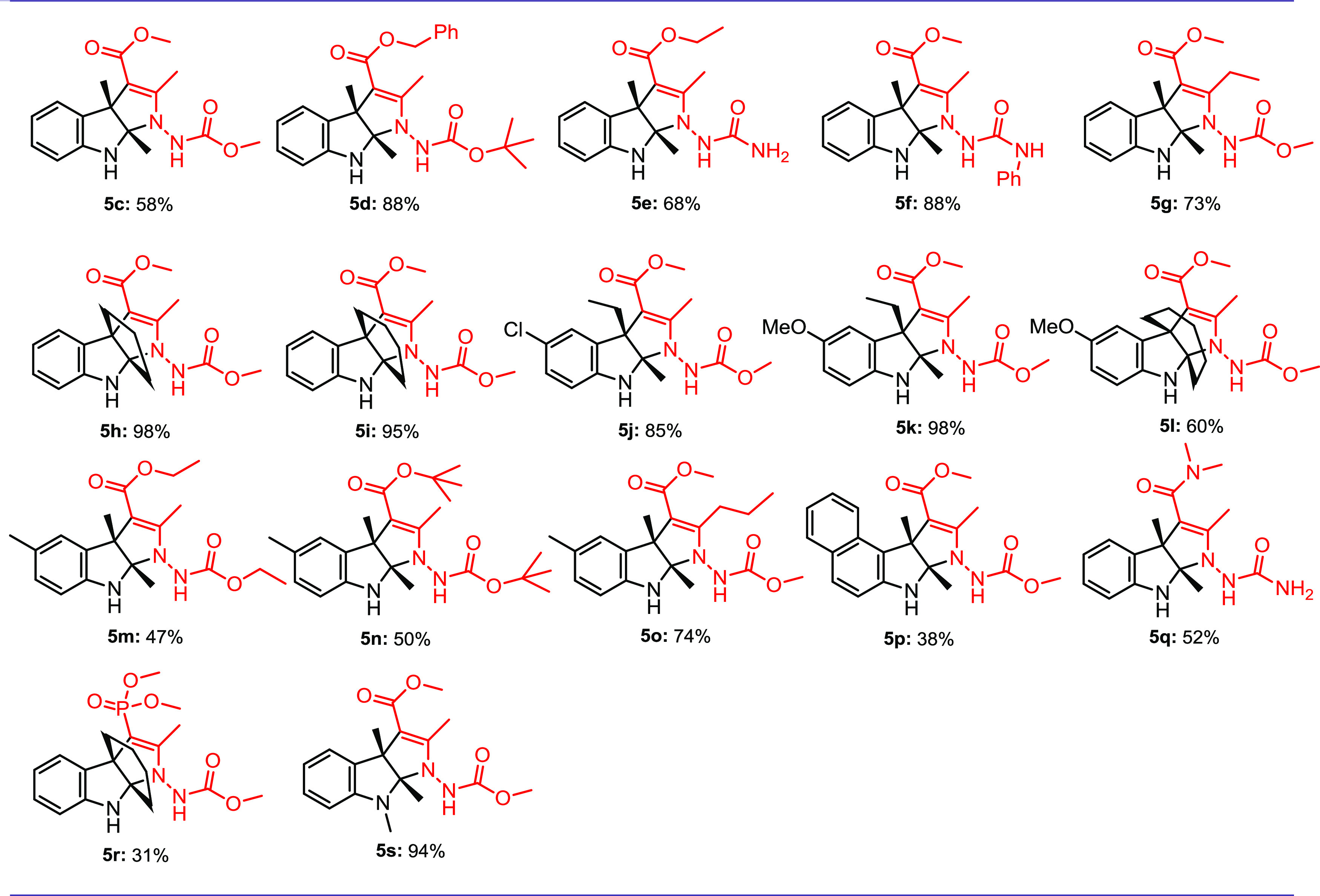
Scope of the Zn(II)-Catalyzed [3 + 2] Cycloaddition Reaction of 2,3-Substituted Indoles (**1**) and Linear Azoalkenes (**2**)^a^^,^^b^

a Reaction conditions: **1** (0.6 mmol), **2** (0.4 mmol), ZnCl_2_ (0.04 mmol, 10 mol %), DCM (2.0 mL), 25 °C.

b Isolated yields.

The structures of compounds **5a**–**s** were confirmed by subjecting **5s** to N–N bond cleavage using the Magnus method.^16^ Treatment of compound **5s** with ethyl bromoacetate/Cs_2_CO_3_/MeCN at 50 °C followed by heating to 80 °C resulted in N–N′ bond cleavage to the corresponding *N*H-free tetrahydropyrrolo[2,3-*b*]indole **6a** in 64% isolated yield (Scheme 4a).

As a synthetic strategy, this [3 + 2] annulation affords, in a single operation, the structurally rigid 6-5-5 tricyclic subunit with a substituent at the 3-position of the indole nucleus, which is the basic structure of pharmaceutically valuable natural products.^4^ Besides, this nonclassical approach provides access to functionalized pyrroloindoline systems with substitution patterns that are otherwise inaccessible using tryptamines^17^ as precursors.

**Figure 2:**
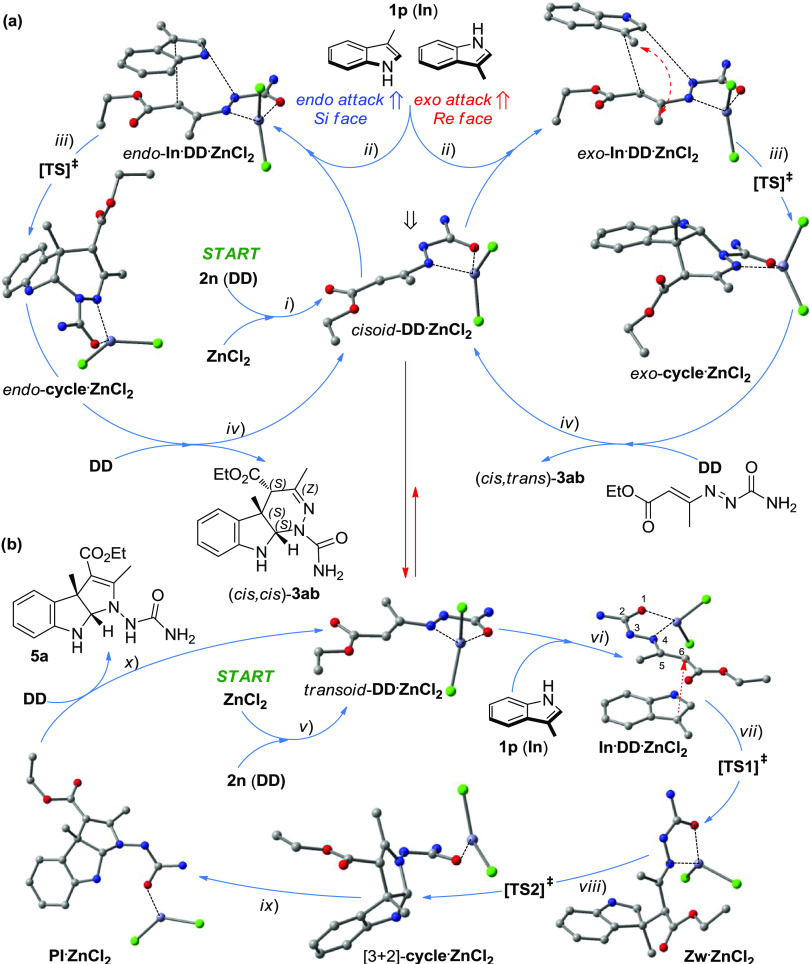
Catalytic cycles for the model reactants **2n** (**DD**) and **1p** (**In**) catalyzed by ZnCl_2_. (a) [4 + 2] cycloaddition: (i) *cisoid*-**DD·ZnCl**_**2**_ catalytic complex formation; (ii) exo or endo adduct formation, *exo*-**In·DD·ZnCl**_**2**_ or *endo*-**In·DD·ZnCl**_**2**_; (iii) cycloaddition through the transition state [TS]^‡^ affording the pyridazino indoline product complex, *endo*-**cycle·ZnCl**_**2**_ or *exo*-**cycle·ZnCl**_**2**_; (iv) substitution with **DD** affording (*cis*,*cis*)-**3ab** and *cisoid*-**DD·ZnCl**_**2**_ restoration. (b) [3 + 2] Cycloaddition: (v) *transoid*-**DD·ZnCl**_**2**_ catalytic complex formation; (vi) nonpericyclic **In·DD·ZnCl**_**2**_ adduct formation; (vii) [1,6]-addition to form the zwitterionic intermediate **Zw·ZnCl**_**2**_ through the transition state [TS1]^‡^; (viii) ring-closure through [TS2]^‡^ affording the nonchelated [3 + 2]-**cycle·ZnCl**_**2**_ complex, (ix) [1,3]-H shift (tautomerization) giving the pyrazolo indoline product complex, **PI·ZnCl**_**2**_; (x) substitution with **DD** affording **5b** and restoring the *transoid*-**DD·ZnCl**_**2**_. For clarity, the H atoms of the DFT-optimized structures are omitted.

The mechanism of the two divergent cycloadditions was studied by density functional theory (DFT) computational chemistry (model chemistry: B3LYP/6-31-G(d)/SCRF = PCM, solvent = DCM,^18,19^ Gaussian16 software;^20^ all details are available in the SI). We focused our attention on the reaction of 1,2-diaza-1,3-diene **2n** (**DD**) with 3-methyl indole **1p** (**In**), since such a combination affords both cycloaddition products, *i.e*., (*cis*,*cis*)-**3ab** (with a de of 99% by ^1^H NMR) and **5a**, in the ratio of about 1:1, after column chromatography separation (Scheme 2). To begin with, we assumed a concerted mechanism for the [4 + 2] cycloaddition (Figure 2a) and a two-step mechanism for the nonpericyclic [3 + 2] cycloaddition (Figure 2b).

**Figure 3:**
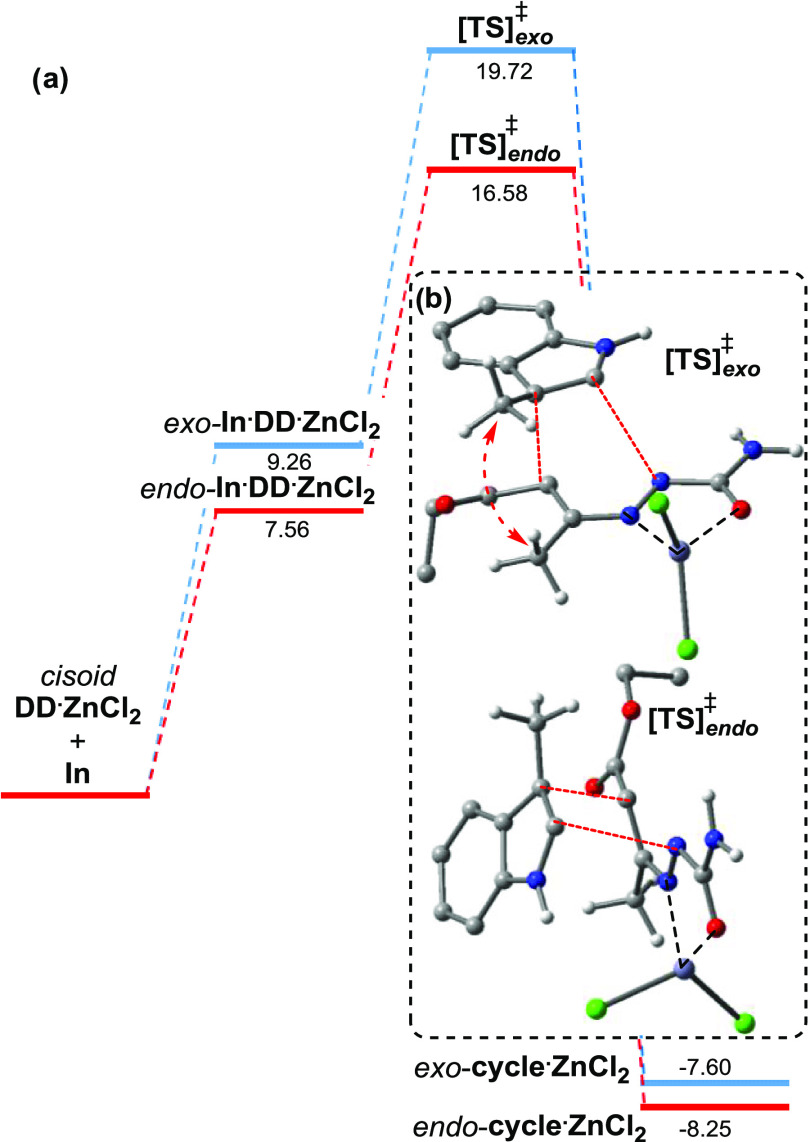
(a) DFT-computed Gibbs free energy profile of the rate-limiting step of the [4 + 2] cycloaddition in CH_2_Cl_2_ at 298 K for reagents 1,2-diaza-1,3-diene **2n** and indole **1p**. The energies (kcal mol^–1^) are reported with respect to the *cisoid*-**DD·ZnCl**_**2**_ and **In** species. (b) Structures of endo and exo transition states; for clarity, some H atoms have been omitted.

The computed [4 + 2] energy reaction paths starting from the *cisoid*-1,2-diaza-1,3-diene·ZnCl_2_·catalytic complex (*cisoid*-**DD·ZnCl**_**2**_) leading to the complex *endo*-**cycle·ZnCl**_**2**_ and to *exo*-**cycle·ZnCl**_**2**_ are reported in Figure 3a; since the reaction is highly exoergonic, both reaction trajectories go through a typical reactant-like transition state [TS]^‡^ having pericyclic topology. Both exo and endo transition states ([TS]_exo_^‡^ and [TS]_endo_^‡^) are shown in Figure 3b.

The computations show clearly that the observed high diastereoselectivity toward the formation of the slightly less stable (*cis*,*cis*)-**3ab** pyridazino indoline ((cis,cis) → (cis,trans), Δ*G*° = −2.66 kcal mol^–1^) is obtained under kinetic control. Indeed, since its endo cyclic precursor is substantially more stable than the exo adduct (ΔΔ*G*^‡^ = −1.70 kcal mol^–1^, mainly for the lack of the steric clashes of the two methyl groups; see Figure 2a), the two associated activation energy barriers are very different (Δ*G*^‡^ = 9.02 *vs* 10.46 kcal mol^–1^); thus, the endo path is kinetically more favorable. Interestingly, in both [TS]^‡^, the ratio between the two forming C–C and C–N single bonds is about 1.3 (Figure 3b), which is symptomatic of an asynchronous concerted transition state.^21^

**Figure 4:**
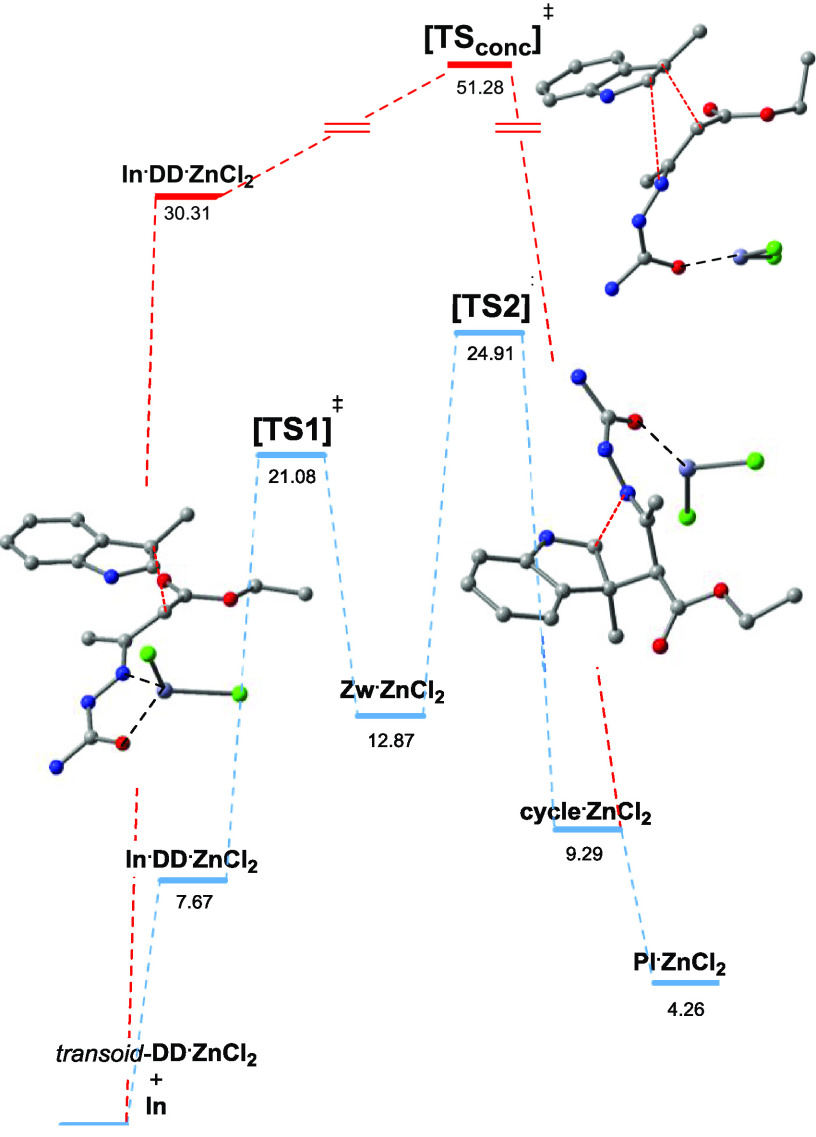
Computed Gibbs free energy profile of the [3 + 2] cyclization: stepwise mechanism (blue path) *vs* the concerted mechanism (red path) in CH_2_Cl_2_ at 298 K. The energies (kcal mol^–1^) are reported with respect to the *transoid*-**DD·ZnCl**_**2**_ and **In** species. For clarity, the H atoms of transition-state structures have been omitted.

The comparison of the [3 + 2] cycloaddition energy diagram of the two stepwise mechanisms with that of the concerted cycloaddition suggested by Gilchrist et al. with very similar substrates^7b,8^ shows clearly that the latter mechanism is not active in our case (Figure 4).

The stepwise catalytic cycle is based on the formation of the very stable *transoid*-**DD·ZnCl**_**2**_ (transoid/cisoid, 99.4:0.6; see the SI), followed by the [1,6]-addition of indole to give the zwitterionic intermediate (**Zw·ZnCl**_**2**_**)** through [TS1]^‡^; then, the latter ring closes to form the nonchelated [3 + 2]-**cycle·ZnCl**_**2**_ complex through [TS2]^‡^. According to our computations, the energy barriers associated with these two steps are very similar (Δ*G*_1_^‡^ = 13.41 kcal mol^–1^*vs* Δ*G*_2_^‡^ = 12.04 kcal mol^–1^). However, the catalytic cycle ends through the following non-rate-limiting steps: [1,3]-H shift (tautomerization), product delivery, and *transoid*-**DD·ZnCl**_**2**_ catalytic complex restoration by substitution with a new molecule of **DD**.

Finally, as a corollary of the above-reported computations, we used them to evaluate the order of magnitude of the product ratio [(*cis*,*cis*)-pyridazinio indoline (**3ab**)]/[pyrazolo indoline (**5b**)] in comparison with the value experimentally obtained (∼1:1, after column chromatography separation). To this end, we have conveniently summarized the scheme of the two divergent cyclization reactions as follows
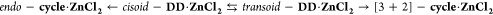
Since the two-reactant catalytic complexes (the *cisoid*-**DD·ZnCl**_**2**_ and the *transoid*-**DD·ZnCl**_**2**_) are in equilibrium, and their interconversion is much faster than the cycloaddition reaction rates, it is possible to apply the Curtin–Hammet equation,^22^ which, in our case with a ΔΔ*G*^‡^ = [TS]_endo_^‡^ – [TS1]^‡^ = 0.50 kcal mol^–1^, gave a ratio of 7:3, (*cis*,*cis*)-**3ab** and pyrazole indoline **5b**, respectively. We reckon that this result is fair enough, considering the chemical accuracy attainable *via* the used model chemistry.

**Scheme 3:**
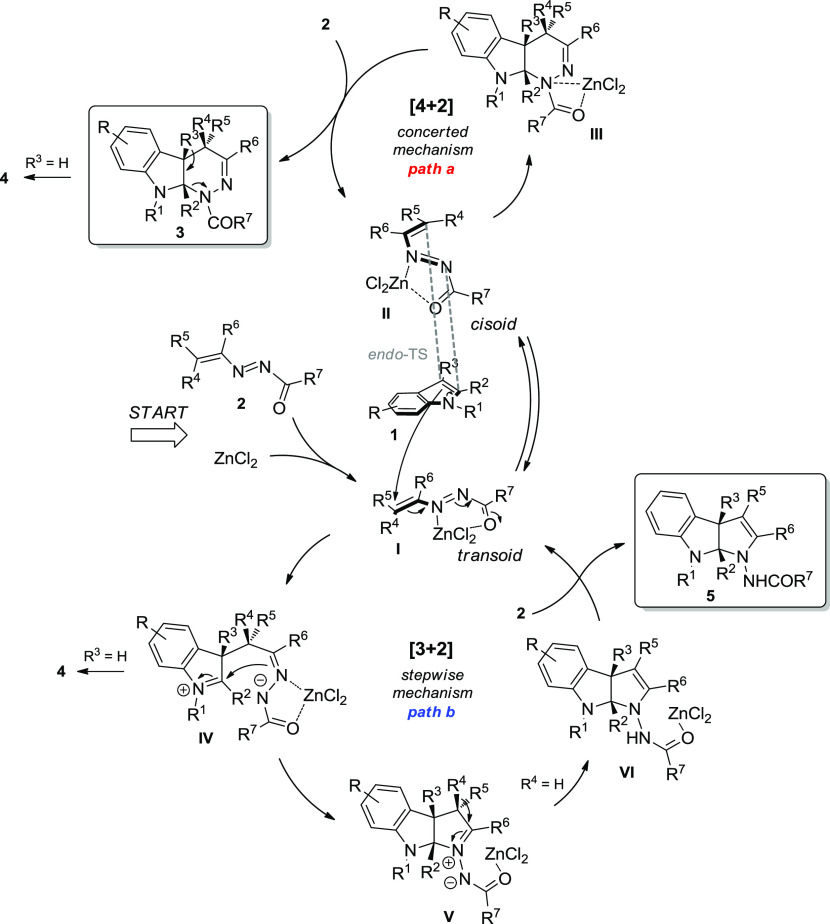
Plausible Reaction Mechanism for Zn(II)-Catalyzed Annulation Reactions

Combining the above experimental results, DFT studies, and available literature,^7,10e^ a reasonable mechanism for these annulation processes is summarized in Scheme 3. Two competing (and independent) reaction pathways for both the tetrahydro-1*H*-pyridazino[3,4-*b*]indole and tetrahydropyrrolo[2,3-*b*]indole derivatives appeared to take place upon initial ZnCl_2_ activation of the 1,2-diaza-1,3-diene substrate. The [4 + 2] cycloaddition (path a) can be simply rationalized as a concerted inverse hetero-Diels–Alder reaction. The preference for an *endo* cycloaddition transition state, which requires the cisoid conformation for DD **2** (**II**), supports the high observed diastereoselectivity for product **3**.^23^ Alternatively, [3 + 2] annulation (path b) can be viewed as proceeding *via* a stepwise process. Regioselective 1,6-addition of the indole nucleophile **1** on activated DD **2** (**I**) that is in a transoid conformation affords the zwitterionic intermediate **IV**, which undergoes intramolecular 5-*exo*-trig cyclization collapsing to the five-membered azomethine imide **V**. The subsequent 1,3-H shift furnishes *via* intermediate **VI** the tetrahydropyrrolo[2,3-*b*]indole product **5** and restores the ZnCl_2_–diene catalytic complex.^24^ The fact that the indole **1q** gave both [4 + 2] and [3 + 2] cycloadducts using cyclic (R^4^ ≠ H) and linear (R^4^ = H) DDs (**3ad***vs***5b**) supported this mechanism scenario.

**Scheme 4:**
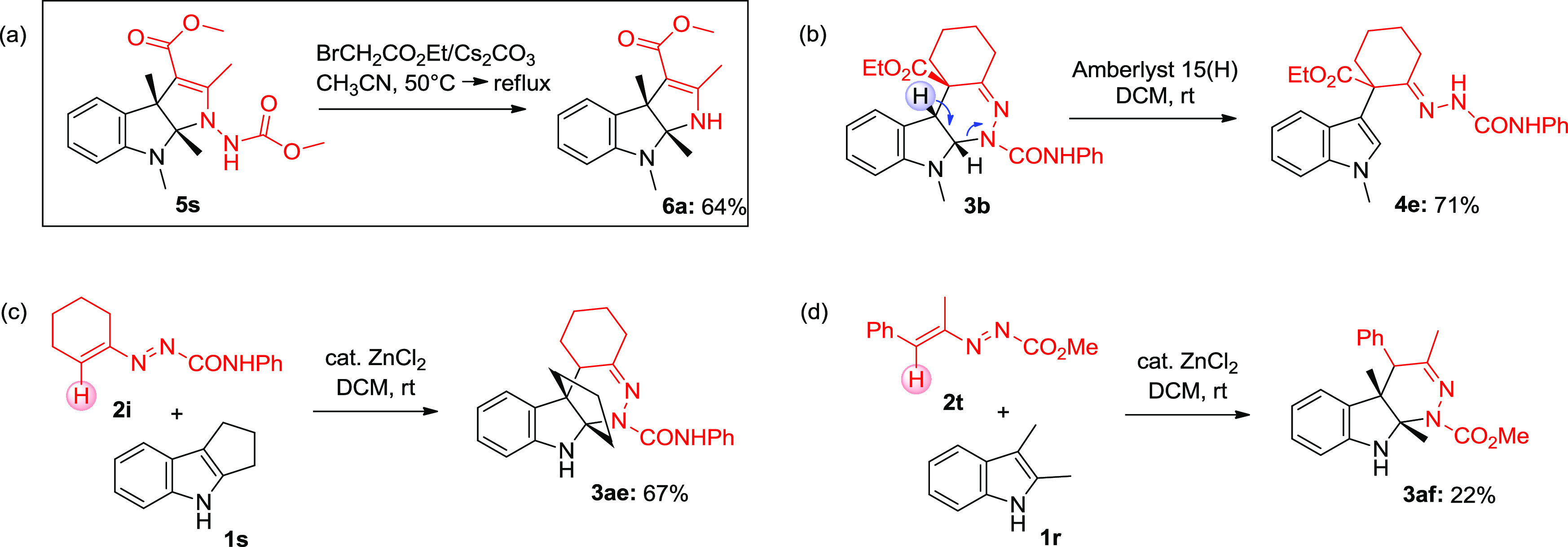
Control Experiments

Likewise, the borderline example of Scheme 2 in which both cycloadducts **3ab** and **5a** concurrently formed^15^ from **1p** and **2n** illustrates the delicate balance and subtle nuances between the two annulation processes. It is evident that, in the presence of additional substituents on the indole ring (R^3^ ≠ H), the [3 + 2] mode of addition becomes competitive since the concerted [4 + 2] pathway is more susceptible to steric inhibition. Moreover, it was quite interesting to note that when six-membered cyclic 1,2-diaza-1,3-diene **2i** was reacted with **1s**, the exclusive formation of the [4 + 2] cycloaddition product **3ae** was observed (Scheme 4c). Similarly, the use of linear 1,2-diaza-1,3-diene **2t** yielded the product **3af** (Scheme 4d). Our control experiments illustrate that the absence of EWG groups like esters, amides, or phosphonates in the C4 position of the starting DD (R^4^ = H; R^5^ ≠ CO_2_R, CONR_2_, and PO(OR)_2_), which likely disfavors the proton transfer process (**V** → **VI**), also privileged the [4 + 2] mode of addition.

With this work, we have demonstrated that the nature and type of substituents of both 1,2-diaza-1,3-diene and indole substrates are critical factors dictating chemoselectivity in the annulation process. Notably, the presence of a H atom in the C3 position of the indole ring is responsible for the observed ring-opened [4 + 2] product **4**. As already evidenced, this event becomes prevailing when *N-*methyl indole (**1a**) or 1,2-dimethyl indole (**1o**) is used as the nucleophile. To our surprise, when R^3^ = H, neither the formation of the [3 + 2] annulation product nor the ring-opened [3 + 2] product of type **7** described by Tan and co-workers was observed.^25^ This result shows that when R^3^ = H, the indole rearomatization process from **3** (and/or eventually from intermediate **IV**) to **4** is the preferred one.

## Conclusions

In conclusion, we have developed substrate-dependent divergent annulation reactions^26^ of indoles with 1,2-diaza-1,3-dienes. By virtue of the versatility of these latter in switching reactivities, efficient synthesis of two types of polycyclic fused indoline scaffolds tetrahydro-1*H*-pyridazino[3,4-*b*]indoles and tetrahydropyrrolo[2,3-*b*]indoles was achieved. The DFT study revealed that [4 + 2] cycloadditions are concerted but quite asynchronous, while [3 + 2] reactions go undoubtedly through a stepwise mechanism. Our approach expands the scope of polycyclic fused indoline synthesis and increases the flexibility of synthetic strategies toward heterocycle-based scaffolds. Remarkably, the reactions feature a high step- and atom-economy, high chemo- and diastereoselectivity, broad substrate scope, good functional group tolerance, and readily accessible starting materials. The successful construction of unique rigid polycyclic skeletons, particularly those with challenging bridgehead *N*,*N*-aminal quaternary centers, enriches the chemistry of both indoles and 1,2-diaza-1,3-dienes.

## Experimental Section

### General Experimental Details

Indoles **1a**, **1l**, **1m**, **1o**, **1p**, **1r**, and **1s** are commercially available reagents and used without further purification. *N*-Alkylindole derivatives **1b**–**k**, **1n**, and **1q** were prepared from corresponding commercially available *N*H-indoles following literature procedures.^27^ 3,4-Disubstituted indoles **1t**–**z** were synthesized from corresponding phenylhydrazine hydrochlorides as starting materials *via* Fisher indole synthesis according to the literature.^28^ 1,2-Diaza-1,3-dienes (DDs) **2a**–**t** were synthesized from the corresponding hydrazones following literature procedures.^29^ Chromatographic purification of compounds was carried out on silica gel (60–200 μm). TLC analysis was performed on preloaded (0.25 mm) glass-supported silica gel plates (Kieselgel 60); compounds were visualized by exposure to UV light and by dipping the plates in 1% Ce(SO_4_)·4H_2_O and 2.5% (NH_4_)_6_Mo_7_O_24_·4H_2_O in 10% sulfuric acid, followed by heating on a hot plate. All ^1^H NMR and ^13^C NMR spectra were recorded at 400 and 100 MHz, respectively, using dimethyl sulfoxide (DMSO)-*d*_6_ or CDCl_3_ on K_2_CO_3_ as the solvent. Chemical shifts (δ scale) are reported in parts per million (ppm) relative to the central peak of the solvent and are sorted in a descending order within each group. The following abbreviations are used to describe peak patterns where appropriate: s, singlet; d, doublet; t, triplet; q, quartet; sex, sextet; m, multiplet; and br, broad signal. All coupling constants (*J* value) are given in hertz (Hz). Structural assignments were made with additional information from gradient correlation spectroscopy (gCOSY), gradient heteronuclear multiple quantum correlation (gHMQC), gradient heteronuclear multiple bond correlation (gHMBC), and nuclear Overhauser enhancement spectroscopy (NOESY) experiments. Fourier transform infrared (FT-IR) spectra were obtained as Nujol mulls or neat. High- and low-resolution mass spectroscopies were performed on a Micromass Q-ToF Micro mass spectrometer (Micromass, Manchester, U.K.) using an electrospray ionization (ESI) source. Melting points were determined in open capillary tubes and are uncorrected. Elemental analyses were within ±0.4 of the theoretical values (C, H, N).

### General Procedure for the Formal [4 + 2] Cycloaddition Reactions of Indoles **1** with Cyclic Azoalkenes **2**

A mixture of indole **1** (2.0 mmol), azoalkene **2** (1.0 mmol), and zinc dichloride (0.1 mmol, 13.6 mg) was stirred in dry dichloromethane (2 mL). After the disappearance of azoalkene **2** (TLC check), the crude mixture was purified by column chromatography on silica gel to afford product **3**. In some cases (see Table 1), a more polar ring-opened [4 + 2] byproduct **4** was also recovered.

#### (6aS*,11bR*,11cR*)-Ethyl 6-Carbamoyl-7-methyl-2,3,4,6,6a,7,11b,11c-octahydro-1*H*-indolo[2,3-*c*]cinnoline-11c-carboxylate (**3a**)

The product **3a** was isolated by column chromatography (ethyl acetate/cyclohexane 30:70) in 50% yield (178.2 mg); white solid; mp: 183–185 °C; ^1^H NMR (400 MHz, DMSO-*d*_6_) δ 7.19 (dt, *J*_1_ = 7.6 Hz, *J*_2_ = 1.2 Hz, 1 H), 7.07 (d, *J* = 7.6 Hz, 1 H), 6.80 (br, 2 H), 6.79 (dt, *J*_1_ = 7.6 Hz, *J*_2_ = 1.2 Hz, 1H), 6.62 (d, *J* = 7.6 Hz, 1H), 5.50 (d, *J* = 7.2 Hz, 1H), 4.20–4.35 (m, 2H), 3.44 (d, *J* = 7.2 Hz, 1H), 2.58 (s, 3H), 2.44–2.51 (m, 1H), 2.26 (dt, *J*_1_ = 12.0 Hz, *J*_2_ = 4.4 Hz, 1H), 1.78–1.84 (m, 1H), 1.50–1.63 (m, 2H), 1.24–1.35 (m, 2H), 1.26 (t, *J* = 7.2 Hz, 3H), 0.95 (dt, *J*_1_ = 12.0 Hz, *J*_2_ = 4.4 Hz, 1H); ^13^C{^1^H} NMR (100 MHz, CDCl_3_) δ 173.3, 157.3, 155.3, 151.7, 129.3, 126.5, 125.7, 118.9, 108.7, 69.4, 61.8, 45.6, 42.4, 35.5, 33.8, 33.1, 27.4, 23.7, 14.3; IR (nujol): υ_max_ = 3485, 3471, 1724, 1692 cm^–1^; MS (ESI) *m*/*z* = 357 [M + H]^+^; anal. calcd for C_19_H_24_N_4_O_3_ (356.42): C 64.03, H 6.79, N 15.72; found: C 63.91, H 6.84, N 15.82.

#### (6aS*,11b*R,11cR*)-Ethyl 7-Methyl-6-(phenylcarbamoyl)-2,3,4,6,6a,7,11b,11c-octahydro-1*H*-indolo[2,3-*c*]cinnoline-11c-carboxylate (**3b**)

The product **3b** was isolated by column chromatography (ethyl acetate/cyclohexane 10:90) in 56% yield (242.3 mg); white solid; mp: 183–185 °C; ^1^H NMR (400 MHz, DMSO-*d*_6_) δ 9.31 (s, 1H), 7.68 (dd, *J*_1_ = 8.4 Hz, *J*_2_ = 1.2 Hz, 2H), 7.31 (t, *J* = 8.0 Hz, 2H), 7.18 (dt, *J*_1_ = 7. 6 Hz, *J*_2_ = 0.8 Hz, 1H), 7.02–7.08 (m, 2H), 6.78 (dt, *J*_1_ = 7.6 Hz, *J*_2_ = 0.8 Hz, 1H), 6.62 (d, *J* = 8.0 Hz, 1H), 5.58 (d, *J* = 7.2 Hz, 1H), 4.19–4.34 (m, 2H), 3.52 (d, *J* = 7.2 Hz, 1H), 2.68 (d, *J* = 13.2 Hz, 1H), 2.60 (s, 3H), 2.30 (dt, *J*_1_ = 12.8 Hz, *J*_2_ = 4.4 Hz, 1H), 1.82–1.84 (m, 1H), 1.62 (d, *J* = 13.2 Hz, 1H), 1.50–1.52 (m, 1H), 1.30–1.41 (m, 2H), 1.27 (t, *J* = 7.2 Hz, 3H), 1.02 (dt, *J*_1_ = 12.8 Hz, *J*_2_ = 4.4 Hz, 1H); ^13^C{^1^H} NMR (100 MHz, DMSO-*d*_6_) δ 172.8, 154.8, 153.4, 151.6, 139.2, 129.5, 128.9, 126.8, 125.8, 123.2, 120.3, 119.2, 109.2, 69.5, 61.9, 45.3, 41.8, 34.9, 34.3, 33.1, 27.3, 23.4, 14.4; IR (nujol): υ_max_ = 3388, 1728, 1690 cm^–1^; MS (ESI) *m*/*z* = 433 [M + H]^+^; anal. calcd for C_25_H_28_N_4_O_3_ (432.51): C 69.42, H 6.53, N 12.95; found: C 69.30, H 6.59, N 13.06.

#### (6aS*,11bR*,11cR*)-Ethyl 6-Carbamoyl-9-chloro-7-methyl-2,3,4,6,6a,7,11b,11c-octahydro-1*H*-indolo[2,3-*c*]cinnoline-11c-carboxylate (**3c**)

The product **3c** was isolated by column chromatography (ethyl acetate/cyclohexane 30:70) in 22% yield (86.1 mg); white solid; mp: 188–190 °C; ^1^H NMR (400 MHz, DMSO-*d*_6_) δ 7.01 (d, *J* = 8.0 Hz, 1H), 6.77 (br, 2H), 6.76 (dd, *J*_1_ = 8.0 Hz, *J*_2_ = 2.0 Hz, 1H), 6.63 (d, *J* = 2.0 Hz, 1H), 5.55 (d, *J* = 7.2 Hz, 1H), 4.18–4.29 (m, 2H), 3.46 (d, *J* = 7.2 Hz, 1H), 2.56 (s, 3H), 2.45 (d, *J* = 13.2 Hz, 1H), 2.23 (dt, *J*_1_ = 12.8 Hz, *J*_2_ = 4.4 Hz, 1H), 1.78–1.80 (m, 1H), 1.58 (d, *J* = 13.2 Hz, 1H), 1.51–1.53 (m, 1H), 1.27–1.32 (m, 2H), 1.25 (t, *J* = 7.2 Hz, 3H), 0.96 (dt, *J*_1_ = 12.8 Hz, *J*_2_ = 4.4 Hz, 1H); ^13^C{^1^H} NMR (100 MHz, DMSO-*d*_6_) δ 172.8, 156.8, 153.5, 153.1, 134.0, 126.9, 125.9, 118.4, 109.0, 69.2, 61.8, 44.8, 41.4, 34.8, 33.6, 32.9, 27.1, 23.3, 14.3; IR (nujol): υ_max_ = 3280, 3206, 1732, 1692 cm^–1^; MS (ESI) *m*/*z* = 413 [M + Na]^+^, 391 [M + H]^+^; anal. calcd for C_19_H_23_ClN_4_O_3_ (390.86): C 58.38, H 5.93, N 14.33; found: C 58.51, H 5.98, N 14.23.

#### (6aS*,11bR*,11cR*)-Ethyl 7-Benzyl-6-carbamoyl-2,3,4,6,6a,7,11b,11c-octahydro-1*H*-indolo[2,3-*c*]cinnoline-11c-carboxylate (**3d**)

The product **3d** was isolated by column chromatography (ethyl acetate/cyclohexane 35:65) in 30% yield (129.7 mg); white solid; mp: 162–164 °C; ^1^H NMR (400 MHz, DMSO-*d*_6_) δ 7.19–7.32 (m, 5H), 7.01–7.07 (m, 2H), 6.74 (br, 2H), 6.71 (t, *J* = 7.6 Hz, 1H), 6.27 (d, *J* = 7.6 Hz, 1H), 5.88 (d, *J* = 6.8 Hz, 1H), 4.49 (d, *J* = 16.0 Hz, 1H), 4.20–4.32. (m, 2H), 3.96 (d, *J* = 16.0 Hz, 1H), 3.49 (d, *J* = 6.8 Hz, 1H), 2.50–2.55 (m, 1H), 2.26 (dt, *J*_1_ = 12.8 Hz, *J*_2_ = 4.8 Hz, 1H), 1.85–1.89 (m, 1H), 1.50–1.59 (m, 2H), 1.30–1.37 (m, 2H), 1.27 (t, *J* = 7.2 Hz, 3H), 1.03–1.11 (m, 1H); ^13^C{^1^H} NMR (100 MHz, DMSO-*d*_6_) δ 173.1, 157.1, 154.1, 150.9, 140.1, 129.3, 128.7, 127.2, 127.1, 126.7, 125.8, 118.7, 108.5, 68.6, 61.8, 50.4, 45.1, 42.2, 35.2, 33.1, 27.6, 23.6, 14.4; IR (nujol): υ_max_ = 3271, 3194, 1738, 1688 cm^–1^; MS (ESI) *m*/*z* = 433 [M + H]^+^; anal. calcd for C_25_H_28_N_4_O_3_ (432.51): C 69.42, H 6.53, N 12.95; found: C 69.31, H 6.49, N 13.06.

#### (7aS*,12bR*,12cR*)-Methyl 7-Carbamoyl-8-methyl-1,2,3,4,5,7,7a,8,12b,12c-decahydrocyclohepta[5,6]pyridazino[3,4-*b*]indole-12c-carboxylate (**3e**)

The product **3e** was isolated by column chromatography (ethyl acetate/cyclohexane 70:30) in 82% yield (292.3 mg); white solid; mp: 171–173 °C; ^1^H NMR (400 MHz, DMSO-*d*_6_) δ 7.16 (d, *J* = 8.0 Hz, 1H), 7.01 (t, *J* = 7.6 Hz, 1H), 6.51 (t, *J* = 7.6 Hz, 1H), 6.35 (br, 2H), 6.28 (d, *J* = 8.0 Hz, 1H), 6.03 (d, *J* = 9.6 Hz, 1H), 4.57 (d, *J* = 9.6 Hz, 1H), 3.60 (s, 3H), 2.69 (s, 3H), 2.52–2.58 (m, 1H), 2.20–2.27 (m, 1H), 2.01–2.05 (m, 1H), 1.72–1.86 (m, 4H), 1.46–1.56 (m, 1H), 1.20–1.34 (m, 2H); ^13^C{^1^H} NMR (100 MHz, DMSO-*d*_6_) δ 173.1, 170.6, 157.1, 152.5, 128.6, 126.1, 124.3, 116.2, 104.8, 73.4, 54.2, 53.1, 52.1, 32.6, 31.6, 30.2, 24.9, 24.6, 24.8; IR (nujol): υ_max_ = 3262, 3194, 1718, 1696 cm^–1^; MS (ESI) *m*/*z* = 357 [M + H]^+^; anal. calcd for C_19_H_24_N_4_O_3_ (356.42): C 64.03, H 6.79, N 15.72; found: C 64.19, H 6.71, N 15.60.

#### (7aS*,12bR*,12cR*)-Methyl 8-Methyl-7-(phenylcarbamoyl)-1,2,3,4,5,7,7a,8,12b,12c-decahydrocyclohepta[5,6]pyridazino[3,4-*b*]indole-12c-carboxylate (**3f**)

The product **3f** was isolated by column chromatography (ethyl acetate/cyclohexane 30:70) in 65% yield (281.2 mg); white solid; mp: 140–142 °C; ^1^H NMR (400 MHz, DMSO-*d*_6_) δ 8.67 (s, 1H), 7.61 (dd, *J*_1_ = 8.4 Hz, *J*_2_ = 0.8 Hz, 2H), 7.29 (t, *J* = 8.0 Hz, 2H), 7.21 (d, *J* = 7.6 Hz, 1H), 7.01–7.06 (m, 2H), 6.55 (dt, *J*_1_ = 7.6 Hz, *J*_2_ = 0.8 Hz, 1H), 6.32 (d, *J* = 7.6 Hz, 1H), 6.16 (d, *J* = 9.6 Hz, 1H), 4.65 (d, *J* = 9.6 Hz, 1H), 3.60 (s, 3H), 2.73 (s, 3H), 2.70 (d, *J* = 6.8 Hz, 1H), 2.28–2.35 (m, 1H), 2.05–2.10 (m, 1H), 1.80–1.93 (m, 4H), 1.54–1.64 (m, 1H), 1.22–1.38 (m, 2H); ^13^C{^1^H} NMR (100 MHz, DMSO-*d*_6_) δ 173.4, 172.3, 154.0, 152.8, 139.3, 129.1, 128.9, 126.6, 124.7, 123.2, 120.4, 116.9, 105.5, 74.5, 55.7, 53.4, 52.7, 37.1, 33.1, 32.3, 30.6, 25.4, 25.1; IR (nujol): υ_max_ = 3345, 1725, 1691 cm^–1^; MS (ESI) *m*/*z* = 433 [M + H]^+^; anal. calcd for C_25_H_28_N_4_O_3_ (432.51): C 69.42, H 6.53, N 12.95; found: C 69.29, H 6.58, N 13.06.

#### (7aS*,12bR*,12cR*)-7-*tert*-Butyl 12c-Methyl 8-methyl-1,2,3,4,5,7a,8,12c-octahydrocyclohepta[5,6]pyridazino[3,4-*b*]indole-7,12c(12b*H*)-dicarboxylate (**3g**)

The product **3g** was isolated by column chromatography (ethyl acetate/cyclohexane 15:85) in 62% yield (256.4 mg); white solid; mp: 124–126 °C; ^1^H NMR (400 MHz, DMSO-*d*_6_) δ 7.18 (d, *J* = 7.6 Hz, 1H), 7.01 (dt, *J*_1_ = 7.6 Hz, *J*_2_ = 0.8 Hz, 1H), 6.51 (dt, *J*_1_ = 7.6 Hz, *J*_2_ = 0.8 Hz, 1H), 6.31 (d, *J* = 7.6 Hz, 1H), 5.92 (d, *J* = 9.2 Hz, 1H), 4.60 (d, *J* = 9.2 Hz, 1H), 3.59 (s, 3H), 2.71 (s, 3H), 2.47 (d, *J* = 7.6 Hz, 2H), 2.30 (t, *J* = 14.0 Hz, 1H), 2.03 (dd, *J*_1_ = 14.0 Hz, *J*_2_ = 7.2 Hz, 1H), 1.69–1.82 (m, 4H), 1.46 (s, 9H), 1.18–1.34 (m, 2H); ^13^C{^1^H} NMR (100 MHz, DMSO-*d*_6_) δ 174.0, 173.6, 152.6, 129.2, 129.1, 126.6, 124.4, 116.7, 105.2, 80.8, 76.1, 54.9, 54.1, 52.5, 36.9, 33.0, 31.4, 30.8, 28.3, 25.3, 24.9; IR (nujol): υ_max_ = 1732, 1730 cm^–1^; MS (ESI) *m*/*z* = 414 [M + H]^+^; anal. calcd for C_23_H_31_N_3_O_4_ (413.51): C 66.81, H 7.56, N 10.16; found: C 66.96, H 7.60, N 10.05.

#### (7aS*,12bR*,12cR*)-Methyl 7-Carbamoyl-10-chloro-8-methyl-1,2,3,4,5,7,7a,8,12b,12c-decahydrocyclohepta[5,6]pyridazino[3,4-*b*]indole-12c-carboxylate (**3h**)

The product **3h** was isolated by column chromatography (ethyl acetate/cyclohexane 45:55) in 90% yield (351.8 mg); white solid; mp: 161–163 °C; ^1^H NMR (400 MHz, DMSO-*d*_6_) δ 7.14 (dd, *J*_1_ = 8.0 Hz, *J*_2_ = 0.8 Hz, 1H), 6.50 (dd, *J*_1_ = 8.0 Hz, *J*_2_ = 2.0 Hz, 1H), 6.32 (d, *J* = 2.0 Hz, 1H), 6.10 (d, *J* = 9.6 Hz, 1H), 4.57 (d, *J* = 9.6 Hz, 1H), 3.60 (s, 3H), 2.69 (s, 3H), 2.52–2.60 (m, 1H), 2.15–2.21 (m, 1H), 2.01–2.04 (m, 1H), 1.74–1.89 (m, 4H), 1.51–1.56 (m, 1H), 1.16–1.35 (m, 3H), 0.81–0.87 (m, 1H); ^13^C{^1^H} NMR (100 MHz, DMSO-*d*_6_) δ 173.4, 171.6, 157.3, 154.2, 134.1, 127.7, 123.8, 115.7, 104.7, 74.0, 54.7, 53.2, 52.6, 37.1, 32.9, 31.6, 30.6, 25.3, 25.0; IR (nujol): υ_max_ = 3287, 3215, 1730, 1701 cm^–1^; MS (ESI) *m*/*z* = 391 [M + H]^+^; anal. calcd for C_19_H_23_ClN_4_O_3_ (390.86): C 58.38, H 5.93, N 14.33; found: C 58.51, H 5.97, N 14.25.

#### (7aS*,12bR*,12cR*)-Methyl 7-Carbamoyl-8,9-dimethyl-1,2,3,4,5,7,7a,8,12b,12c-decahydrocyclohepta[5,6]pyridazino[3,4-*b*]indole-12c-carboxylate (**3i**)

The product **3i** was isolated by column chromatography (ethyl acetate/cyclohexane 40:60) in 54% yield (200.1 mg); yellow oil; ^1^H NMR (400 MHz, DMSO-*d*_6_) δ 7.07 (d, *J* = 7.6 Hz, 1H), 6.82 (d, *J* = 7.6 Hz, 1H), 6.58 (t, *J* = 7.6 Hz, 1H), 6.40 (br, 2H), 5.81 (d, *J* = 10.0 Hz, 1H), 4.66 (d, *J* = 10.0 Hz, 1H), 3.59 (s, 3H), 2.91 (s, 3H), 2.53 (dd, *J*_1_ = 14.0 Hz, *J*_2_ = 7.2 Hz, 1H), 2.21–2.32 (m, 1H), 2.17 (s, 3H), 2.03 (dd, *J*_1_ = 14.0 Hz, *J*_2_ = 7.2 Hz, 1H), 1.71–1.83 (m, 4H), 1.45–1.56 (m, 1H), 1.18–1.33 (m, 2H); ^13^C{^1^H} NMR (100 MHz, DMSO-*d*_6_) δ 173.2, 169.0, 157.4, 152.1, 131.4, 126.6, 123.8, 118.8, 118.6, 76.8, 54.3, 52.9, 52.1, 36.7, 33.1, 30.3, 24.9, 24.7, 18.9, 14.1; IR (nujol): υ_max_ = 3227, 3217, 1735, 1693 cm^–1^; MS (ESI) *m*/*z* = 371 [M + H]^+^; anal. calcd for C_20_H_26_N_4_O_3_ (370.44): C 64.84, H 7.07, N 15.12; found: C 64.69, H 6.99, N 15.24.

#### (7aS*,12bR*,12cR*)-Dimethyl 7-Carbamoyl-8-methyl-1,2,3,4,5,7,7a,8,12b,12c-decahydrocyclohepta[5,6]pyridazino[3,4-*b*]indole-11,12c-dicarboxylate (**3j**)

The product **3j** was isolated by column chromatography (ethyl acetate/cyclohexane 50:50) in 89% yield (368.9 mg); white solid; mp: 218–220 °C; ^1^H NMR (400 MHz, DMSO-*d*_6_) δ 7.70 (s, 1H), 7.67 (s, 1H), 6.50 (br, 2H), 6.31 (d, *J* = 8.0 Hz, 1H), 6.20 (d, *J* = 9.6 Hz, 1H), 4.65 (d, *J* = 9.6 Hz, 1H), 3.76 (s, 3H), 3.62 (s, 3H), 2.76 (s, 3H), 2.57 (dd, *J*_1_ = 14.0 Hz, *J*_2_ = 6.8 Hz, 1H), 2.07–2.19 (m, 2H), 1.77–1.83 (m, 4H), 1.53 (q, *J* = 12.4 Hz, 1H), 1.16–1.33 (m, 2H); ^13^C{^1^H} NMR (100 MHz, DMSO-*d*_6_) δ 173.3, 172.3, 166.6, 157.3, 156.4, 132.2, 127.6, 124.6, 116.8, 103.7, 73.7, 54.8, 53.1, 52.7, 51.8, 37.0, 32.8, 30.9, 30.7, 25.3, 24.9; IR (nujol): υ_max_ = 3267, 3211, 1729, 1727, 1684 cm^–1^; MS (ESI) *m*/*z* = 415 [M + H]^+^; anal. calcd for C_21_H_26_N_4_O_5_ (414.45): C 60.86, H 6.32, N 13.52; found: C 70.01, H 6.26, N 13.41.

#### (7aS*,12bR*,12cR*)-Methyl 7-Carbamoyl-11-cyano-8-methyl-1,2,3,4,5,7,7a,8,12b,12c-decahydrocyclohepta[5,6]pyridazino[3,4-*b*]indole-12c-carboxylate (**3k**)

The product **3k** was isolated by column chromatography (ethyl acetate/cyclohexane 45:55) in 84% yield (320.4 mg); white solid; mp: 273–275 °C; ^1^H NMR (400 MHz, DMSO-*d*_6_) δ 7.49 (s, 1H), 7.44 (dd, *J*_1_ = 8.4 Hz, *J*_2_ = 1.2 Hz, 1H), 6.55 (br, 2H), 6.37 (d, *J* = 8.4 Hz, 1H), 6.21 (d, *J* = 9.6 Hz, 1H), 4.65 (d, *J* = 9.6 Hz, 1H), 3.61 (s, 3H), 2.75 (s, 3H), 2.57 (dd, *J*_1_ = 14.0 Hz, *J*_2_ = 6.8 Hz, 1H), 2.15–2.22 (m, 1H), 2.05 (dd, *J*_1_ = 14.0 Hz, *J*_2_ = 6.8 Hz, 1H), 1.78–1.91 (m, 4H), 1.48–1.59 (m, 1H), 1.32–1.41 (m, 1H), 1.16–1.25 (m, 1H); ^13^C{^1^H} NMR (100 MHz, DMSO-*d*_6_) δ 173.2, 172.5, 157.2, 155.7, 134.9, 129.9, 125.6, 121.0, 104.6, 96.7, 73.5, 54.6, 53.1, 52.7, 37.0, 32.7, 30.8, 30.5, 25.3, 24.9; IR (nujol): υ_max_ = 3293, 3219, 1724, 1686 cm^–1^; MS (ESI) *m*/*z* = 382 [M + H]^+^; anal. calcd for C_20_H_23_N_5_O_3_ (381.43): C 62.98, H 6.08, N 18.36; found: C 62.83, H 6.15, N 18.47.

#### (7aS*,12bR*,12cR*)-Dimethyl 7-Carbamoyl-8-methyl-1,2,3,4,5,7,7a,8,12b,12c-decahydrocyclohepta[5,6]pyridazino[3,4-*b*]indole-11,12c-dicarboxylate (**3l**)

The product **3l** was isolated by column chromatography (ethyl acetate/cyclohexane 60:40) in 89% yield (342.2 mg); white solid; mp: 212–214 °C; ^1^H NMR (400 MHz, DMSO-*d*_6_) δ 9.60 (s, 1H), 7.61 (s, 1H), 7.60 (d, *J* = 8.0 Hz, 1H), 6.62 (br, 2H), 6.40 (d, *J* = 8.0 Hz, 1H), 6.24 (d, *J* = 10.0 Hz, 1H), 4.68 (d, *J* = 10.0 Hz, 1H), 3.61 (s, 3H), 2.79 (s, 3H), 2.57 (dd, *J*_1_ = 14.4 Hz, *J*_2_ = 7.2 Hz, 1H), 2.08–2.24 (m, 2H), 1.73–1.87 (m, 4H), 1.48–1.57 (m, 1H), 1.15–1.35 (m, 2H); ^13^C{^1^H} NMR (100 MHz, DMSO-*d*_6_) δ 190.1, 173.3, 172.7, 157.5, 157.2, 134.8, 127.3, 126.1, 125.4, 103.9, 73.8, 54.8, 52.8, 52.7, 37.1, 32.8, 30.8, 30.7, 25.3, 24.9; IR (nujol): υ_max_ = 3261, 3213, 1736, 1725, 1690 cm^–1^; MS (ESI) *m*/*z* = 385 [M + H]^+^; anal. calcd for C_20_H_24_N_4_O_4_ (384.43): C 62.49, H 6.29, N 14.57; found: C 62.35, H 6.33, N 14.44.

#### (7aS*,12bR*,12cR*)-Methyl 7-Carbamoyl-8-methyl-11-nitro-1,2,3,4,5,7,7a,8,12b,12c-decahydrocyclohepta[5,6]pyridazino[3,4-*b*]indole-12c-carboxylate (**3m**)

The product **3m** was isolated by column chromatography (ethyl acetate/cyclohexane 20:80) in 92% yield (369.3 mg); yellow solid; mp: 180–182 °C; ^1^H NMR (400 MHz, DMSO-*d*_6_) δ 8.01 (dd, *J*_1_ = 9.2 Hz, *J*_2_ = 2.0 Hz, 1H), 7.94 (d, *J* = 2.0 Hz, 1H), 6.61 (br, 2H), 6.39 (d, *J* = 9.2 Hz, 1H), 6.31 (d, *J* = 9.6 Hz, 1H), 4.72 (d, *J* = 9.6 Hz, 1H), 3.62 (s, 3H), 2.81 (s, 3H), 2.54–2.61 (m, 1H), 2.14–2.17 (m, 2H), 1.73–1.94 (m, 4H), 1.48–1.59 (m, 1H), 1.19–1.39 (m, 2H); ^13^C{^1^H} NMR (100 MHz, DMSO-*d*_6_) δ 172.6, 172.5, 157.2, 156.6, 136.4, 127.5, 124.8, 122.6, 102.8, 73.5, 54.2, 52.3, 52.2, 36.5, 32.1, 30.4, 30.1, 24.9, 24.4; IR (nujol): υ_max_ = 3362, 3347, 1736, 1692 cm^–1^; MS (ESI) *m*/*z* = 402 [M + H]^+^; anal. calcd for C_19_H_23_N_5_O_5_ (401.41): C 56.85, H 5.78, N 17.45; found: C 57.02, H 5.69, N 17.33.

#### (7aS*,12bR*,12cR*)-Methyl 7-Carbamoyl-11-methoxy-8-methyl-1,2,3,4,5,7,7a,8,12b,12c-decahydrocyclohepta[5,6]pyridazino[3,4-*b*]indole-12c-carboxylate (**3n**)

The product **3n** was isolated by column chromatography (ethyl acetate/cyclohexane 40:60) in 71% yield (274.4 mg); white solid; mp: 164–166 °C; ^1^H NMR (400 MHz, DMSO-*d*_6_) δ 6.79 (d, *J* = 2.4 Hz, 1H), 6.64 (dd, *J*_1_ = 8.4 Hz, *J*_2_ = 2.4 Hz, 1H), 6.50 (br, 2H), 6.24 (d, *J* = 8.4 Hz, 1H), 5.95 (d, *J* = 9.6 Hz, 1H), 4.51 (d, *J* = 9.6 Hz, 1H), 3.65 (s, 3H), 3.61 (s, 3H), 2.64 (s, 3H), 2.56 (dd, *J*_1_ = 14 Hz, *J*_2_ = 6.8 Hz, 1H), 2.14–2.22 (m, 1H), 1.89–2.03 (m, 2H), 1.70–1.81 (m, 3H), 1.47–1.55 (m, 1H), 1.15–1.35 (m, 2H); ^13^C{^1^H} NMR (100 MHz, DMSO-*d*_6_) δ 173.6, 169.6, 157.7, 151.8, 147.5, 126.5, 114.5, 113.3, 106.0, 74.7, 56.0, 54.3, 53.3, 52.7, 37.1, 33.6, 32.9, 30.5, 25.4, 25.1; IR (nujol): υ_max_ = 3274, 3215, 1726, 1676 cm^–1^; MS (ESI) *m*/*z* = 387 [M + H]^+^; anal. calcd for C_20_H_26_N_4_O_4_ (386.44): C 62.16, H 6.78, N 14.50; found: C 62.31, H 6.84, N 14.39.

#### (7aS*,12bR*,12cR*)-Methyl 7-Carbamoyl-8-ethyl-1,2,3,4,5,7,7a,8,12b,12c-decahydrocyclohepta[5,6]pyridazino[3,4-*b*]indole-12c-carboxylate (**3o**)

The product **3o** was isolated by column chromatography (ethyl acetate/cyclohexane 40:60) in 56% yield (207.5 mg); white solid; mp: 115–117 °C; ^1^H NMR (400 MHz, DMSO-*d*_6_) δ 7.14 (d, *J* = 7.6 Hz, 1H), 6.98 (t, *J* = 7.6 Hz, 1H), 6.47 (t, *J* = 7.6 Hz, 1H), 6.35 (br, 2H), 6.25 (d, *J* = 7.6 Hz, 1H), 6.15 (d, *J* = 10.0 Hz, 1H), 4.59 (d, *J* = 10.0 Hz, 1H), 3.59 (s, 3H), 3.30 (q, *J* = 6.8 Hz, 2H), 3.07 (sex, *J* = 7.2 Hz, 1H), 2.57 (dd, *J*_1_ = 14.0 Hz, *J*_2_ = 7.2 Hz, 1H), 2.19–2.28 (m, 1H), 1.97–2.06 (m, 1H), 1.73–1.88 (m, 3H), 1.47–1.57 (m, 1H), 1.15–1.34 (m, 2H), 0.96 (t, *J* = 6.8 Hz, 3H); ^13^C{^1^H} NMR (100 MHz, DMSO-*d*_6_) δ 173.2, 171.1, 157.1, 151.2, 128.6, 126.4, 124.2, 115.7, 104.4, 71.6, 54.2, 53.4, 52.1, 38.5, 36.7, 32.7, 30.3, 24.9, 24.5, 11.3; IR (nujol): υ_max_ = 3372, 3346, 1729, 1691 cm^–1^; MS (ESI) *m*/*z* = 371 [M + H]^+^; anal. calcd for C_20_H_26_N_4_O_3_ (370.44): C 64.84, H 7.07, N 15.12; found: C 64.71, H 7.11, N 15.23.

#### (7aS*,12bR*,12cR*)-Methyl 7-Carbamoyl-11-methyl-8-propyl-1,2,3,4,5,7,7a,8,12b,12c-decahydrocyclohepta[5,6]pyridazino[3,4-*b*]indole-12c-carboxylate (**3p**)

The product **3p** was isolated by column chromatography (ethyl acetate/cyclohexane 40:60) in 71% yield (283.0 mg); white solid; mp: 119–121 °C; ^1^H NMR (400 MHz, DMSO-*d*_6_) δ 6.96 (s, 1H), 6.78 (d, *J* = 8.0 Hz, 1H), 6.42 (br, 2H), 6.15 (d, *J* = 8.0 Hz, 1H), 6.12 (d, *J* = 10.0 Hz, 1H), 4.56 (d, *J* = 10.0 Hz, 1H), 3.60 (s, 3H), 3.11–3.20 (m, 1H), 2.90–2.98 (m, 1H), 2.57 (dd, *J*_1_ = 13.2 Hz, *J*_2_ = 7.2 Hz, 1H), 2.23 (q, *J* = 13.2 Hz, 3H), 2.16 (s, 3H), 2.02 (dd, *J*_1_ = 13.2 Hz, *J*_2_ = 7.2 Hz, 1H), 1.71–1.93 (m, 3H), 1.16–1.59 (m, 4H), 0.78 (t, *J* = 7.2 Hz, 3H); ^13^C{^1^H} NMR (100 MHz, DMSO-*d*_6_) δ 173.8, 171.3, 157.5, 150.2, 129.2, 127.6, 124.7, 124.5, 104.8, 72.9, 54.6, 53.9, 52.6, 46.9, 37.2, 33.2, 30.7, 25.5, 25.0, 20.9, 20.1, 11.8; IR (nujol): υ_max_ = 3291, 3219, 1732, 1688 cm^–1^; MS (ESI) *m*/*z* = 399 [M + H]^+^; anal. calcd for C_22_H_30_N_4_O_3_ (398.50): C 66.31, H 7.59, N 14.06; found: C 66.46, H 7.63, N 13.96.

#### (7aS*,12bR*,12cR*)-Methyl 8-Benzyl-7-carbamoyl-1,2,3,4,5,7,7a,8,12b,12c-decahydrocyclohepta[5,6]pyridazino[3,4-*b*]indole-12c-carboxylate (**3q**)

The product **3q** was isolated by column chromatography (ethyl acetate/cyclohexane 35:65) in 79% yield (341.7 mg); white solid; mp: 158–160 °C; ^1^H NMR (400 MHz, DMSO-*d*_6_) δ 7.16–7.30 (m, 6H), 6.91 (t, *J* = 7.6 Hz, 1H), 6.51 (t, *J* = 7.6 Hz, 1H), 6.30 (d, *J* = 10.0 Hz, 1H), 6.20 (br, 2H), 6.09 (d, *J* = 8.0 Hz, 1H), 4.71 (d, *J* = 10.0 Hz, 1H), 4.61 (d, *J* = 16.8 Hz, 1H), 4.22 (d, *J* = 16.8 Hz, 1H), 3.60 (s, 3H), 2.65 (dd, *J*_1_ = 14.0 Hz, *J*_2_ = 6.8 Hz, 1H), 2.30 (t, *J* = 12.4 Hz, 1H), 2.07 (dd, *J*_1_ = 14.0 Hz, *J*_2_ = 6.8 Hz, 1H), 1.92 (t, *J* = 12.4 Hz, 1H), 1.72–1.86 (m, 3H), 1.57 (q, *J* = 12.4 Hz, 1H), 1.19–1.37 (m, 2H); ^13^C{^1^H} NMR (100 MHz, DMSO-*d*_6_) δ 173.3, 171.5, 157.2, 151.8, 138.7, 128.5, 128.3, 126.8, 126.6, 126.5, 124.3, 116.4, 105.2, 72.9, 54.3, 53.7, 52.2, 49.1, 36.8, 32.8, 30.3, 25.0, 24.6; IR (nujol): υ_max_ = 3279, 3208, 1733, 1678 cm^–1^; MS (ESI) *m*/*z* = 433 [M + H]^+^; anal. calcd for C_25_H_28_N_4_O_3_ (432.51): C 69.42, H 6.53, N 12.95; found: C 69.57, H 6.59, N 12.84.

#### (7aS*,12bR*,12cR*)-Methyl 7-Carbamoyl-1,2,3,4,5,7,7a,8,12b,12c-decahydrocyclohepta[5,6]pyridazino[3,4-*b*]indole-12c-carboxylate (**3r**)

The product **3r** was isolated by column chromatography (ethyl acetate/cyclohexane 50:50) in 89% yield (304.7 mg); white solid; mp: 165–167 °C; ^1^H NMR (400 MHz, DMSO-*d*_6_) δ 7.14 (d, *J* = 7.6 Hz, 1H), 6.92 (dt, *J*_1_ = 7.6 Hz, *J*_2_ = 1.2 Hz, 1H), 6.49 (dt, *J*_1_ = 7.6 Hz, *J*_2_ = 1.2 Hz, 1H), 6.44 (d, *J* = 7.6 Hz, 1H), 6.47 (br, 2H), 6.40 (d, *J* = 2.0 Hz, 1H), 5.75 (dd, *J*_1_ = 9.6 Hz, *J*_2_ = 2.0 Hz, 1H), 4.29 (d, *J* = 9.6 Hz, 1H), 3.65 (s, 3H), 2.55 (dd, *J*_1_ = 14.4 Hz, *J*_2_ = 5.6 Hz, 1H), 2.32 (t, *J* = 12.8 Hz, 1H), 1.98 (t, *J* = 12.8 Hz, 2H), 1.71–1.82 (m, 3H), 1.41–1.55 (m, 1H), 1.20–1.35 (m, 2H); ^13^C{^1^H} NMR (100 MHz, DMSO-*d*_6_) δ 172.8, 161.9, 157.6, 151.6, 128.2, 125.6, 124.5, 116.8, 108.0, 69.0, 53.1, 52.3, 51.1, 36.7, 32.2, 29.6, 25.2, 24.8; IR (nujol): υ_max_ = 3426, 3251, 3228, 1737, 1692 cm^–1^; MS (ESI) *m*/*z* = 343 [M + H]^+^; anal. calcd for C_18_H_22_N_4_O_3_ (342.39): C 63.14, H 6.48, N 16.36; found: C 62.97, H 6.56, N 16.49.

#### (7aS*,12bR*,12cR*)-Methyl 7-Carbamoyl-11-methyl-1,2,3,4,5,7,7a,8,12b,12c-decahydrocyclohepta[5,6]pyridazino[3,4-*b*]indole-12c-carboxylate (**3s**)

The product **3s** was isolated by column chromatography (ethyl acetate/cyclohexane 40:60) in 66% yield (235.2 mg); white solid; mp: 198–200 °C; ^1^H NMR (400 MHz, DMSO-*d*_6_) δ 6.95 (s, 1H), 6.75 (d, *J* = 7.6 Hz, 1H), 6.41 (br, 2H), 6.38 (d, *J* = 7.6 Hz, 1H), 6.09 (s, 1H), 5.74 (dd, *J*_1_ = 9.2 Hz, *J*_2_ = 2.0 Hz, 1H), 4.20 (d, *J* = 9.2 Hz, 1H), 3.67 (s, 3H), 2.56 (dd, *J*_1_ = 14.4 Hz, *J*_2_ = 5.6 Hz, 1H), 2.31 (t, *J* = 12.8 Hz, 1H), 2.05 (t, *J* = 12.8 Hz, 1H), 1.95 (dd, *J*_1_ = 14.4 Hz, *J*_2_ = 5.6 Hz, 1H), 2.16 (s, 3H), 1.71–1.86 (m, 3H), 1.23–1.51 (m, 3H); ^13^C{^1^H} NMR (100 MHz, DMSO-*d*_6_) δ 172.7, 160.7, 157.4, 149.1, 128.5, 126.1, 125.4, 124.9, 108.1, 69.1, 52.8, 52.1, 50.7, 36.6, 31.9, 29.3, 25.2, 24.7, 20.5; IR (nujol): υ_max_ = 3327, 3271, 1734, 1693 cm^–1^; MS (ESI) *m*/*z* = 357 [M + H]^+^; anal. calcd for C_19_H_24_N_4_O_3_ (356.41): C 64.03, H 6.79, N 15.72; found: C 63.90, H 6.83, N 15.84.

#### (7aS*,12bR*,12cR*)-Methyl 7-Carbamoyl-8-methyl-1,2,3,4,5,7,7a,8,12b,12c-decahydrocyclohepta[*c*]pyrido[3′,2′:4,5]pyrrolo[3,2-*e*]pyridazine-12c-carboxylate (**3t**)

The product **3t** was isolated by column chromatography (ethyl acetate/cyclohexane 80:20) in 85% yield (303.8 mg); white solid; mp: 222–224 °C; ^1^H NMR (400 MHz, DMSO-*d*_6_) δ 7.74 (d, *J* = 4.8 Hz, 1H), 7.42 (d, *J* = 7.2 Hz, 1H), 6.59 (br, 1H), 6.40 (t, *J* = 6.8 Hz, 1H), 6.27 (br, 1H), 6.12 (d, *J* = 10.0 Hz, 1H), 4.58 (d, *J* = 10.0 Hz, 1H), 3.60 (s, 3H), 2.76 (s, 3H), 2.58 (dd, *J*_1_ = 14.4 Hz, *J*_2_ = 6.8 Hz, 1H), 2.14–2.20 (m, 1H), 1.98–2.05 (m, 1H), 1.77–1.85 (m, 4H), 1.51 (q, *J* = 12.4 Hz, 1H), 1.16–1.32 (m, 2H); ^13^C{^1^H} NMR (100 MHz, DMSO-*d*_6_) δ 173.2, 172.2, 162.4, 157.4, 147.1, 133.6, 118.7, 112.0, 71.1, 54.8, 52.7, 51.7, 37.1, 32.9, 30.7, 29.2, 25.3, 24.9; IR (nujol): υ_max_ = 3355, 3296, 1736, 1689 cm^–1^; MS (ESI) *m*/*z* = 358 [M + H]^+^; anal. calcd for C_18_H_23_N_5_O_3_ (357.41): C 60.49, H 6.49, N 19.59; found: C 60.63, H 6.41, N 19.48.

#### (8aS*,13bR*,13cR*)-Ethyl 8-Carbamoyl-9-methyl-2,3,4,5,6,8,8a,9,13b,13c-decahydro-1*H*-cycloocta[5,6]pyridazino[3,4-*b*]indole-13c-carboxylate (**3u**)

The product **3u** was isolated by column chromatography (ethyl acetate/cyclohexane 30:70) in 46% yield (177.5 mg); white solid; mp: 182–184 °C; ^1^H NMR (400 MHz, DMSO-*d*_6_) δ 7.13 (dt, *J*_1_ = 7.6 Hz, *J*_2_ = 0.8 Hz, 1H), 6.98 (d, *J* = 7.2 Hz, 1H), 6.72 (s, 2H), 6.67 (dt, *J*_1_ = 7.6 Hz, *J*_2_ = 0.8 Hz, 1H), 6.50 (d, *J* = 8.0 Hz, 1H), 5.61 (dd, *J* = 8.4 Hz, 1H), 4.23 (q, *J* = 7.2 Hz, 2H), 3.72 (d, *J* = 8.4 Hz, 1H), 2.74 (s, 3H), 2.33–2.40 (m, 2H), 1.90–1.98 (m, 1H), 1.47–1.75 (m, 7H), 1.28 (t, *J* = 7.2 Hz, 3H), 1.21–1.30 (m, 2H); ^13^C{^1^H} NMR (100 MHz, DMSO-*d*_6_) δ 172.2, 157.6, 156.4, 151.7, 128.7, 125.5, 124.7, 117.7, 106.8, 71.4, 61.0, 50.2, 45.9, 34.1, 33.1, 29.0, 25.9, 25.8, 25.7, 23.4, 13.8; IR (nujol): υ_max_ = 3408, 3394, 1720, 1684 cm^–1^; MS (ESI) *m*/*z* = 385 [M + H]^+^; anal. calcd for C_21_H_28_ N_4_ O_3_ (384.47): C 65.60, H 7.34, N 14.57; found: C 65.74, H 7.39, N 14.43.

#### (8aS*,13bR*,13cR*)-8-*tert*-Butyl 13c-Ethyl 9-methyl-3,4,5,6,8a,9,13b,13c-octahydro-1*H*-cycloocta[5,6]pyridazino[3,4-*b*]indole-8,13c(2*H*)-dicarboxylate (**3v**)

The product **3v** was isolated by column chromatography (ethyl acetate/cyclohexane 10:90) in 43% yield (189.9 mg); white solid; mp: 157–159 °C; ^1^H NMR (400 MHz, DMSO-*d*_6_) δ 7.14 (d, *J* = 8.0 Hz, 1H), 7.02 (t, *J* = 7.6 Hz, 1H), 6.53 (t, *J* = 7.6 Hz, 1H), 6.36 (d, *J* = 8.0 Hz, 1H), 5.74 (d, *J* = 9.2 Hz, 1H), 4.40 (d, *J* = 9.2 Hz, 1H), 3.98–4.18 (m, 2H), 3.37 (s, 1H), 2.72 (s, 3H), 2.25–2.34 (m, 1H), 2.14–2.18 (m, 1H), 1.91–2.13 (m, 2H), 1.52–1.70 (m, 6H), 1.47 (s, 9H), 1.31–1.42 (m, 1H), 1.19 (t, *J* = 7.2 Hz, 3H); ^13^C{^1^H} NMR (100 MHz, DMSO-*d*_6_) δ 171.8, 170.2, 152.5, 151.9, 128.6, 125.2, 124.4, 116.7, 105.3, 80.4, 74.9, 60.9, 53.3, 53.2, 32.6, 31.3, 31.0, 27.8, 27.7, 25.6, 25.2, 24.7, 13.7; IR (nujol): υ_max_ = 1732, 1724 cm^–1^; MS (ESI) *m*/*z* = 442 [M + H]^+^; anal. calcd for C_25_H_35_N_3_O_4_ (441.56): C 68.00, H 7.99, N 9.52; found: C 67.87, H 7.93, N 9.64.

#### (8aS*,13bR*,13cR*)-8-*tert*-Butyl 13c-Ethyl 9-benzyl-3,4,5,6,8a,9,13b,13c-octahydro-1*H*-cycloocta[5,6]pyridazino[3,4-*b*]indole-8,13c(2*H*)-dicarboxylate (**3w**)

The product **3w** was isolated by column chromatography (ethyl acetate/cyclohexane 10:90) in 50% yield (258.8 mg); yellowish oil; ^1^H NMR (400 MHz, DMSO-*d*_6_) δ 7.17–7.32 (m, 6H), 6.92 (t, *J* = 7.6 Hz, 1H), 6.52 (t, *J* = 7.6 Hz, 1H), 6.10 (d, *J* = 8.0 Hz, 1H), 6.02 (d, *J* = 9.6 Hz, 1H), 4.73 (d, *J* = 9.6 Hz, 1H), 4.65 (d, *J* = 17.2 Hz, 1H), 4.24 (d, *J* = 17.2 Hz, 1H), 3.93–4.14 (m, 2H), 2.43–2.49 (m, 1H), 2.26–2.30 (m, 1H), 2.11–2.16 (m, 1H), 1.93–2.01 (m, 1H), 1.38–1.78 (m, 8H), 1.23 (s, 9H), 1.17 (t, *J* = 7.2 Hz, 3H); ^13^C{^1^H} NMR (100 MHz, DMSO-*d*_6_) δ 173.5, 171.9, 152.4, 151.1, 138.7, 128.5, 128.4, 126.6, 126.1, 125.6, 124.1, 116.6, 104.9, 80.2, 75.5, 60.8, 55.8, 53.8, 49.2, 32.3, 31.7, 28.3, 27.5, 25.8, 25.6, 24.5, 13.7; IR (nujol): υ_max_ = 1731, 1723 cm^–1^; MS (ESI) *m*/*z* = 518 [M + H]^+^; anal. calcd for C_31_H_39_N_3_O_4_ (517.66): C 71.93, H 7.59, N 8.12; found: C 71.76, H 7.65, N 8.26.

#### (8aS*,13bR*,13cR*)-Ethyl 8-Carbamoyl-2,3,4,5,6,8,8a,9,13b,13c-decahydro-1*H*-cycloocta[5,6]pyridazino[3,4-*b*]indole-13c-carboxylate (**3x**)

The product **3x** was isolated by column chromatography (ethyl acetate/cyclohexane 30:70) in 37% yield (136.9 mg); white solid; mp: 168–170 °C; ^1^H NMR (400 MHz, DMSO-*d*_6_) δ 7.01 (t, *J* = 7.2 Hz, 1H), 6.71 (s, 2H), 6.70 (d, *J* = 8.0 Hz, 1H), 6.64 (d, *J* = 8.0 Hz, 1H), 6.59 (t, *J* = 7.2 Hz, 1H), 6.10 (d, *J* = 4.0 Hz, 1H), 5.55 (dd, *J*_1_ = 8.8 Hz, *J*_2_ = 4.0 Hz, 1H), 4.30 (q, *J* = 7.2 Hz, 2H), 3.44 (d, *J* = 8.8 Hz, 1H), 2.66 (dd, *J*_1_ = 14.4 Hz, *J*_2_ = 6.8 Hz, 1H), 2.37–2.46 (m, 1H), 1.90–1.99 (m, 1H), 1.32–1.64 (m, 8H), 1.29 (t, *J* = 7.2 Hz, 3H), 1.07–1.19 (m, 1H); ^13^C{^1^H} NMR (100 MHz, DMSO-*d*_6_) δ 172.0, 158.1, 151.9, 150.3, 128.6, 124.9, 124.3, 117.8, 109.4, 68.3, 61.1, 49.3, 45.6, 35.1, 27.9, 26.0, 24.2, 21.9, 21.3, 13.9; IR (nujol): υ_max_ = 3332, 3315, 3298, 1731, 1688 cm^–1^; MS (ESI) *m*/*z* = 371 [M + H]^+^; anal. calcd for C_20_H_26_ N_4_ O_3_ (370.44): C 64.84, H 7.07, N 15.12; found: C 64.98, H 6.99, N 15.02.

#### (4S*,4aS*,9aS*)-Ethyl 1-Carbamoyl-3,9-dimethyl-4,4a,9,9a-tetrahydro-1*H*-pyridazino[3,4-*b*]indole-4-carboxylate ((*cis*,*cis*)-**3z**)

The more polar product was isolated by column chromatography (ethyl acetate/cyclohexane 20:80); amorphous white solid; ^1^H NMR (400 MHz, DMSO-*d*_6_) δ 7.01–7.07 (m, 2H), 6.64 (s, 2H), 6.59 (dt, *J*_1_ = 7.6 Hz, *J*_2_ = 0.8 Hz, 1H), 6.39 (d, *J* = 7.6 Hz, 1H), 5.74 (d, *J* = 8.0 Hz, 1H), 3.94 (t, *J* = 8.0 Hz, 1H), 3.71–3.88 (m, 2H), 3.54 (d, *J* = 8.0 Hz, 1H), 2.63 (s, 3H), 1.92 (s, 3H), 1.00 (t, *J* = 7.2 Hz, 3H); ^13^C{^1^H} NMR (100 MHz, DMSO-*d*_6_) δ 169.0, 157.5, 151.9, 151.7, 129.1, 127.3, 125.4, 117.8, 107.1, 70.8, 60.9, 44.6, 33.0, 23.0, 14.1; IR (nujol): υ_max_ = 3309, 3298, 1729, 1678 cm^–1^; MS (ESI) *m*/*z* = 317 [M + H]^+^; anal. calcd for C_16_H_20_ N_4_ O_3_ (316.35): C 60.75, H 6.37, N 17.71; found: C 60.64, H 6.49, N 17.56.

During the course of the reaction, the following workup, and the long standing in DMSO-*d*_6_ solution at 20 °C for 24 h, the diastereomer (*cis*,*cis*)-**3z** gives a partial isomerization to more stable (*cis*,*trans*)-**3z** together with the ring-opening reaction leading to the byproduct **4a**. Diastereomers **3z** were isolated in a combined yield of 32%, based on the amount of 1,2-diaza-1,3-diene consumed.

The relative configurations of diastereomers **3z** were assigned by means of two-dimensional (2D) NOESY experiments.

#### (4R*,4aS*,9aS*)-Ethyl 1-Carbamoyl-3,9-dimethyl-4,4a,9,9a-tetrahydro-1*H*-pyridazino[3,4-*b*]indole-4-carboxylate ((*cis*,*trans*)-**3z**)

The less polar product was isolated by column chromatography (ethyl acetate/cyclohexane 20:80); amorphous white solid; ^1^H NMR (400 MHz, DMSO-*d*_6_) δ 7.03–7.11 (m, 2H), 6.34 (dt, *J*_1_ = 7.6 Hz, *J*_2_ = 0.8 Hz, 1H), 6.58 (s, 2H), 6.46 (d, *J* = 7.6 Hz, 1H), 5.72 (d, *J* = 8.4 Hz, 1H), 4.11–4.24 (m, 2H), 3.84 (dd, *J*_1_ = 8.4 Hz, *J*_2_ = 6.0 Hz, 1H), 3.29 (d, *J* = 6.0 Hz, 1H), 2.68 (s, 3H), 1.90 (s, 3H), 1.20 (t, *J* = 7.2 Hz, 3H); ^13^C{^1^H} NMR (100 MHz, DMSO-*d*_6_) δ 170.2, 157.3, 151.8, 150.8, 129.1, 129.0, 123.6, 118.3, 107.3, 70.3, 61.6, 46.4, 33.0, 23.1, 14.4; IR (nujol): υ_max_ = 3314, 3306, 1736, 1682 cm^–1^; MS (ESI) *m*/*z* = 317 [M + H]^+^; anal. calcd for C_16_H_20_ N_4_ O_3_ (316.35): C 60.75, H 6.37, N 17.71; found: C 60.64, H 6.49, N 17.56.

#### (4S*,4aS*,9aS*)-Ethyl 1-Carbamoyl-3,4a-dimethyl-4,4a,9,9a-tetrahydro-1*H*-pyridazino[3,4-*b*]indole-4-carboxylate ((*cis*,*cis*)-**3ab**)

NOESY correlations allowed the assignment of the relative stereochemistry. The compound partially isomerized to (*cis*,*trans*)-**3ab** when allowed to stand in a CDCl_3_ solution at 20 °C for 24 h, while no conversion was observed using DMSO-*d*_6_ as a solvent. The product was isolated by column chromatography (ethyl acetate/cyclohexane 30:70) in 24% yield (75.9 mg); white solid; mp: 171–173 °C; ^1^H NMR (400 MHz, DMSO-*d*_6_) δ 7.21 (d, *J* = 7.6 Hz, 1H), 6.96 (t, *J* = 7.6 Hz, 1H), 6.58–6.65 (m, 3H), 6.54 (d, *J* = 7.6 Hz, 1H), 5.97 (d, *J* = 2.8 Hz, 1H), 5.25 (d, *J* = 2.8 Hz, 1H), 3.57–3.80 (m, 2H), 3.28 (s, 1H), 1.91 (s, 3H), 1.34 (s, 3H), 0.88 (t, *J* = 7.2 Hz, 3H); ^13^C{^1^H} NMR (100 MHz, DMSO-*d*_6_) δ 168.5, 157.5, 149.5, 145.2, 131.6, 128.5, 124.4, 118.0, 109.7, 73.1, 60.8, 51.1, 44.2, 25.4, 23.1, 13.9; IR (nujol): υ_max_ = 3338, 3306, 3301, 1728, 1694 cm^–1^; MS (ESI) *m*/*z* = 317 [M + H]^+^; anal. calcd for C_16_H_20_ N_4_ O_3_ (316.35): C 60.75, H 6.37, N 17.71; found: C 60.59, H 6.31, N 17.58.

#### (7aS*,12bR*,12cR*)-Methyl 7-Carbamoyl-8,12b-dimethyl-1,2,3,4,5,7,7a,8,12b,12c-decahydrocyclohepta[5,6]pyridazino[3,4-*b*]indole-12c-carboxylate (**3ad**)

The product **3ad** was isolated by column chromatography (ethyl acetate/cyclohexane 60:40) in 40% yield (148.2 mg); white solid; mp: 127–129 °C; ^1^H NMR (400 MHz, DMSO-*d*_6_) δ 7.17 (d, *J* = 7.6 Hz, 1H), 7.02 (dt, *J*_1_ = 7.6 Hz, *J*_2_ = 0.8 Hz, 1H), 6.58 (dt, *J*_1_ = 7.6 Hz, *J*_2_ = 0.8 Hz, 1H), 6.45 (br, 2H), 6.38 (d, *J* = 7.6 Hz, 1H), 5.39 (s, 1H), 3.59 (s, 3H), 2.71 (s, 3H), 2.53–2.57 (m, 1H), 2.13–2.24 (m, 1H), 1.91–2.05 (m, 2H), 1.65–1.80 (m, 3H), 1.52 (s, 3H), 1.12–1.43 (m, 3H); ^13^C{^1^H} NMR (100 MHz, DMSO-*d*_6_) δ 172.2, 166.0, 156.5, 150.9, 132.0, 128.2, 123.6, 117.3, 106.0, 78.6, 55.7, 52.3, 51.5, 36.2, 31.8, 29.7, 29.0, 25.1, 24.9, 22.2; IR (nujol): υ_max_ = 3347, 3298, 1729, 1698 cm^–1^; HRMS (ESI) calcd for C_20_H_27_N_4_O_3_[M + H]+: 371.2083; found: 371.2069.

#### *N*-Phenyl-3,4,7,11c-tetrahydro-1*H*-6a,11b-propanoindolo[2,3-*c*]cinnoline-6(2*H*)-carboxamide (**3ae**)

The product **3ae** was isolated by column chromatography (ethyl acetate/cyclohexane 15:85) in 67% yield (258.9 mg); whitish oil; ^1^H NMR (400 MHz, DMSO-*d*_6_) δ 8.54 (s, 1H), 7.41–7.58 (m, 2H), 7.26 (t, *J* = 7.6 Hz, 2H), 6.93–7.01 (m, 3H), 6.14–6.61 (m, 3H), 1.40–2.18 (m, 15H); ^13^C{^1^H} NMR (100 MHz, DMSO-*d*_6_) δ 156.2, 150.1, 139.5, 133.5, 128.6, 127.1, 122.9, 121.8, 118.5, 117.8, 113.2, 108.9, 100.6, 68.9, 26.4, 25.2, 25.1, 22.4, 22.1, 21.2, 20.8, 20.7; IR (nujol): υ_max_ = 3375, 3246, 1696 cm^–1^; MS (ESI) *m*/*z* = 387 [M + H]^+^; anal. calcd for C_24_H_26_N_4_O (386.49): C 74.58, H 6.78, N 14.50; found: C 74.42, H 6.86, N 14.62.

#### Methyl 3,4a,9a-Trimethyl-4-phenyl-4,4a,9,9a-tetrahydro-1*H*-pyridazino[3,4-*b*]indole-1-carboxylate (**3af**)

The product **3af** was isolated by column chromatography (ethyl acetate/cyclohexane 20:80) in 22% yield (76.9 mg); white solid; mp: 186–188 °C, ^1^H NMR (400 MHz, CDCl_3_) δ 7.01–7.12 (m, 3H), 6.88 (dt, *J*_1_ = 7.6 Hz, *J*_2_ = 1.2 Hz, 1H), 6.81–6.85 (m, 2H), 6.67 (d, *J* = 8.0 Hz, 1H), 6.62 (dt, *J*_1_ = 7.6 Hz, *J*_2_ = 1.2 Hz, 1H), 6.25 (d, *J* = 8.0 Hz, 1H), 5.38 (s, 1H), 3.90 (s, 3H), 3.39 (s, 1H), 2.10 (s, 3H), 1.67 (s, 3H), 1.49 (s, 3H); ^13^C{^1^H} NMR (100 MHz, CDCl_3_) δ 157.8, 155.3, 147.7, 134.2, 131.5, 130.0, 128.3, 127.4, 127.1, 123.9, 118.3, 108.9, 82.8, 54.9, 53.5, 52.3, 24.3, 23.4, 21.7; IR (nujol): υ_max_ = 3290, 1736 cm^–1^; MS (ESI) *m*/*z* = 433 [M + H]+; anal. calcd for C_21_H_23_N_3_O_2_ (349.43): C 72.18, H 6.63, N 12.03; found: C 72.03, H 6.72, N 12.17.

### General Procedure for the Formal [3 + 2] Cycloaddition Reactions of Indoles **1** with Linear Azoalkenes **2**

A mixture of indole **1** (0.6 mmol), azoalkene **2** (0.4 mmol), and zinc dichloride (0.04 mmol, 5.45 mg) was stirred in dry dichloromethane (2 mL). After the disappearance of azoalkene **2** (TLC check), the crude mixture was purified by column chromatography on silica gel to afford product **5**.

#### (3aR*,8aS*)-Ethyl 2,3a-Dimethyl-1-ureido-1,3a,8,8a-tetrahydropyrrolo[2,3-*b*]indole-3-carboxylate (**5a**)

The product **5a** was isolated by column chromatography (ethyl acetate/cyclohexane 40:60) in 27% yield (85.3 mg); white solid; mp: 218–220 °C; ^1^H NMR (400 MHz, DMSO-*d*_6_) δ 10.74 (s, 1H), 9.23 (s, 1H), 7.43 (d, *J* = 7.6 Hz, 1H), 7.32 (d, *J* = 7.6 Hz, 1H), 7.05 (t, *J* = 7.6 Hz, 1H), 6.96 (t, *J* = 7.6 Hz, 1H), 6.23 (br, 2H), 4.94 (s, 1H), 4.16 (q, *J* = 7.2 Hz, 2H), 2.20 (s, 3H), 1.82 (s, 3H), 1.21 (t, *J* = 7.2 Hz, 3H); ^13^C{^1^H} NMR (100 MHz, DMSO-*d*_6_) δ 170.1, 157.6, 145.1, 136.2, 128.8, 128.5, 121.5, 118.7, 118.5, 111.6, 108.4, 61.3, 52.0, 15.6, 14.5, 8.8; IR (nujol): υ_max_ = 3478, 3464, 3328, 3321, 1725, 1688 cm^–1^; MS (ESI) *m*/*z* = 317 [M + H]^+^; anal. calcd for C_16_H_20_ N_4_ O_3_ (316.35): C 60.75, H 6.37, N 17.71; found: C 60.88, H 6.29, N 17.84.

#### (3aR*,8aS*)-Methyl 1-((Methoxycarbonyl)amino)-2,3a,8-trimethyl-1,3a,8,8a-tetrahydropyrrolo[2,3-*b*]indole-3-carboxylate (**5b**)

The product **5b** was isolated by column chromatography (ethyl acetate/cyclohexane 30:70) in 46% yield (60.9 mg); white solid; mp: 127–129 °C; ^1^H NMR (400 MHz, CDCl_3_) δ 7.52 (d, *J* = 7.6 Hz, 1H), 7.09 (dt, *J*_1_ = 7.6 Hz, *J*_2_ = 0.8 Hz, 1H), 6.84 (br, 1H), 6.72 (dt, *J*_1_ = 7.6 Hz, *J*_2_ = 0.8 Hz, 1H), 6.45 (d, *J* = 7.6 Hz, 1H), 4.92 (s, 1H), 3.79 (s, 3H), 3.75 (s, 3H), 2.96 (s, 3H), 2.09 (s, 3H), 1.67 (s, 3H); ^13^C{^1^H} NMR (100 MHz, CDCl_3_) δ 166.5, 160.0, 156.1, 149.7, 134.7, 127.9, 124.9, 118.8, 106.9, 106.6, 96.1, 54.5, 53.2, 50.5, 34.8, 25.4, 12.5; IR (nujol): υ_max_ = 3287, 1739, 1701 cm^–1^; HRMS (ESI) calcd for C_17_H_22_N_3_O_4_[M + H]^+^: 332.1610; found: 332.1639.

#### (3aR*,8aS*)-Methyl 1-((Methoxycarbonyl)amino)-2,3a,8a-trimethyl-1,3a,8,8a-tetrahydropyrrolo[2,3-*b*]indole-3-carboxylate (**5c**)

The product **5c** was isolated by column chromatography on silica gel (ethyl acetate/cyclohexane 20:80) in 58% yield (76.9 mg); white solid; mp: 128–130 °C; ^1^H NMR (400 MHz, DMSO-*d*_6_) δ 9.31 (s, 1H), 7.28 (d, *J* = 7.6 Hz, 1H), 6.88 (t, *J* = 7.6 Hz, 1H), 6.55 (t, *J* = 7.6 Hz, 1H), 6.42 (d, *J* = 7.6 Hz, 1H), 6.13 (s, 1H), 3.65 (s, 3H), 3.59 (s, 3H), 2.01 (s, 3H), 1.46 (s, 3H), 1.26 (s, 3H); ^13^C{^1^H} NMR (100 MHz, DMSO-*d*_6_) δ 165.5, 159.1, 157.1, 148.7, 133.7, 126.9, 124.7, 117.3, 107.9, 102.8, 92.8, 55.3, 52.1, 49.8, 19.6, 18.6, 12.0; IR (nujol): υ_max_ = 3274, 1739, 1698 cm^–1^; MS (ESI) *m*/*z* = 332 [M + H]+; anal. calcd for C_17_H_21_N_3_O_4_ (331.36): C 61.62, H 6.39, N 12.68; found: C 61.74, H 6.31, N 12.57.

#### (3aR*,8aS*)-Benzyl 1-((*tert*-Butoxycarbonyl)amino)-2,3a,8a-trimethyl-1,3a,8,8a-tetrahydropyrrolo[2,3-*b*]indole-3-carboxylate (**5d**)

The product **5d** was isolated by column chromatography on silica gel (ethyl acetate/cyclohexane 20:80) in 88% yield (158.3 mg); white solid; mp: 149–151 °C; ^1^H NMR (400 MHz, DMSO-*d*_6_) δ 9.02 (s, 1H), 7.29–7.54 (m, 5H), 7.20 (d, *J* = 7.6 Hz, 1H), 6.86 (t, *J* = 7.6 Hz, 1H), 6.45 (t, *J* = 7.6 Hz, 1H), 6.41 (d, *J* = 7.6 Hz, 1H), 6.13 (s, 1H), 5.10 (s, 2H), 2.03 (s, 3H), 1.46 (s, 3H), 1.43 (s, 9H), 1.27 (s, 3H); ^13^C{^1^H} NMR (100 MHz, DMSO-*d*_6_) δ 164.9, 159.9, 155.6, 148.6, 137.1, 133.9, 128.3, 127.9, 127.7, 126.8, 124.9, 117.2, 107.8, 101.5, 92.8, 79.5, 64.1, 55.4, 27.9, 19.8, 18.4, 12.1; IR (nujol): υ_max_ = 3363, 3324, 1741, 1696 cm^–1^; HRMS (ESI) calcd for C_26_H_32_N_3_O_4_[M + H]^+^: 450.2393; found: 450.2411.

#### (3aR*,8aS*)-Ethyl 2,3a,8a-Trimethyl-1-ureido-1,3a,8,8a-tetrahydropyrrolo[2,3-*b*]indole-3-carboxylate (**5e**)

The product **5e** was isolated by column chromatography on silica gel (ethyl acetate/cyclohexane 70:30) in 68% yield (89.9 mg); white solid; mp: 171–173 °C; ^1^H NMR (400 MHz, DMSO-*d*_6_) δ 7.63 (s, 1H), 7.30 (d, *J* = 6.4 Hz, 1H), 6.88 (t, *J* = 6.4 Hz, 1H), 6.56 (t, *J* = 6.4 Hz, 1H), 6.43 (d, *J* = 6.4 Hz, 1H), 6.17 (br, 2H), 6.01 (br, 1H), 4.05 (q, *J* = 7.2 Hz, 2H), 2.07 (s, 3H), 1.51 (s, 3H), 1.28 (s, 3H), 1.23 (t, *J* = 7.2 Hz, 3H); ^13^C{^1^H} NMR (100 MHz, DMSO-*d*_6_) δ 164.9, 159.7, 159.3, 148.9, 133.1, 126.7, 124.9, 117.0, 107.7, 102.5, 92.9, 58.2, 55.2, 18.6, 15.1, 14.3, 12.1; IR (nujol): υ_max_ = 3482, 3467, 3338, 3325, 1731, 1684 cm^–1^; HRMS (ESI) calcd for C_17_H_23_N_4_O_3_[M + H]^+^: 331.1770; found: 331.1791.

#### (3aR*,8aS*)-Methyl 2,3a,8a-Trimethyl-1-(3-phenylureido)-1,3a,8,8a-tetrahydropyrrolo[2,3-*b*]indole-3-carboxylate (**5f**)

The product **5f** was isolated by column chromatography on silica gel (ethyl acetate/cyclohexane 70:30) in 88% yield (138.1 mg); white solid; mp: 226–228 °C; ^1^H NMR (400 MHz, DMSO-*d*_6_) δ 8.41 (br, 2H), 7.19–7.77 (m, 5H), 6.86–7.05 (m, 2H), 6.41–6.72 (m, 2H), 6.15 (br, 1H), 3.62 (s, 3H), 2.10 (s, 3H), 1.54 (s, 3H), 1.33 (s, 3H); ^13^C{^1^H} NMR (100 MHz, DMSO-*d*_6_) δ 165.5, 159.6, 155.6, 148.6, 139.1, 133.3, 128.5, 126.9, 124.8, 122.1, 118.9, 118.3, 117.6, 108.1, 93.0, 55.4, 49.8, 20.6, 18.8, 12.3; IR (nujol): υ_max_ = 3389, 3282, 3270, 1726, 1694 cm^–1^; HRMS (ESI) calcd for C_22_H_25_N_4_O_3_[M + H]^+^: 393.1927; found: 393.1963.

#### (3aR*,8aS*)-Methyl 2-Ethyl-1-((methoxycarbonyl)amino)-3a,8a-dimethyl-1,3a,8,8a-tetrahydropyrrolo[2,3-*b*]indole-3-carboxylate (**5g**)

The product **5g** was isolated by column chromatography on silica gel (ethyl acetate/cyclohexane 25:75) in 73% yield (100.9 mg); white solid; mp: 157–159 °C; ^1^H NMR (400 MHz, CDCl_3_) δ 7.48 (d, *J* = 7.6 Hz, 1H), 7.01 (dt, *J*_1_ = 7.6 Hz, *J*_2_ = 1.2 Hz, 1H), 6.80 (dt, *J*_1_ = 7.6 Hz, *J*_2_ = 1.2 Hz, 1H), 6.57 (d, *J* = 7.6 Hz, 1H), 6.38 (br, 1H), 3.86 (br, 1H), 3.80 (s, 3H), 3.68 (s, 3H), 2.47–2.68 (m, 2H), 1.62 (s, 3H), 1.42 (s, 3H), 1.07 (t, *J* = 7.2 Hz, 3H); ^13^C{^1^H} NMR (100 MHz, CDCl_3_) δ 165.8, 163.8, 156.9, 147.6, 134.2, 127.5, 126.0, 120.1, 109.5, 105.9, 93.4, 56.4, 53.1, 50.4, 20.0, 19.7, 19.4, 12.5; IR (nujol): υ_max_ = 3332, 3275, 1727, 1692 cm^–1^; HRMS (ESI) calcd for C_18_H_24_N_3_O_4_[M + H]^+^: 346.1767; found: 346.1761.

#### (3aS*,8bR*)-Methyl 11-((Methoxycarbonyl)amino)-10-methyl-1,2,3,4-tetrahydro-3a,8b-(epiminoetheno)cyclopenta[*b*]indole-9-carboxylate (**5h**)

The product **5h** was isolated by column chromatography on silica gel (ethyl acetate/cyclohexane 20:80) in 98% yield (134.8 mg); white solid; mp: 150–152 °C; ^1^H NMR (400 MHz, CDCl_3_) δ 7.54 (d, *J* = 7.6 Hz, 1H), 7.02 (dt, *J*_1_ = 7.6 Hz, *J*_2_ = 1.2 Hz, 1H), 6.79 (dt, *J*_1_ = 7.6 Hz, *J*_2_ = 1.2 Hz, 1H), 6.62 (br, 1H), 6.61 (d, *J* = 7.6 Hz, 1H), 4.16 (br, 1H), 3.79 (s, 3H), 3.75 (s, 3H), 2.16–2.45 (m, 3H), 2.14 (s, 3H), 1.60–1.88 (m, 3H); ^13^C{^1^H} NMR (100 MHz, CDCl_3_) δ 166.6, 159.5, 157.3, 148.7, 135.4, 127.8, 125.9, 120.7, 110.3, 104.3, 99.9, 67.3, 53.2, 50.5, 41.0, 40.2, 25.3, 12.4; IR (nujol): υ_max_ = 3370, 3302, 1718, 1662 cm^–1^; HRMS (ESI) calcd for C_18_H_22_N_3_O_4_ [M + H]^+^: 344.1610; found: 344.1606.

#### (4bR*,8aS*)-Methyl 10-((Methoxycarbonyl)amino)-11-methyl-6,7,8,9-tetrahydro-5*H*-8a,4b-(epiminoetheno)carbazole-12-carboxylate (**5i**)

The product **5i** was isolated by column chromatography on silica gel (ethyl acetate/cyclohexane 30:70) in 95% yield (135.8 mg); yellowish solid; mp: 121–123 °C; ^1^H NMR (400 MHz, DMSO-*d*_6_) δ 9.27 (s, 1H), 7.26 (d, *J* = 7.6 Hz, 1H), 6.89 (t, *J* = 7.6 Hz, 1H), 6.57 (t, *J* = 7.6 Hz, 1H), 6.45 (d, *J* = 7.6 Hz, 1H), 6.04 (s, 1H), 3.64 (s, 3H), 3.58 (s, 3H), 2.04 (s, 3H), 1.40 (s, 8H); ^13^C{^1^H} NMR (100 MHz, DMSO-*d*_6_) δ 165.6, 159.2, 157.1, 149.6, 132.6, 126.8, 124.4, 117.4, 108.3, 103.4, 91.3, 55.0, 52.0, 49.8, 30.3, 26.3, 18.9, 18.5, 12.0; IR (nujol): υ_max_ = 3370, 3302, 1736, 1697 cm^–1^; HRMS (ESI) calcd for C_19_H_24_N_3_O_4_ [M + H]^+^: 358.1767; found: 358.1782.

#### (3aR*,8aS*)-Methyl 5-Chloro-3a-ethyl-1-((methoxycarbonyl)amino)-2,8a-dimethyl-1,3a,8,8a-tetrahydropyrrolo[2,3-*b*]indole-3-carboxylate (**5j**)

The product **5j** was isolated by column chromatography (ethyl acetate/cyclohexane 30:70) in 85% yield (129.2 mg); white solid; mp: 113–115 °C; ^1^H NMR (400 MHz, DMSO-*d*_6_) δ 9.34 (s, 1H), 7.19 (d, *J* = 2.0 Hz, 1H), 6.89 (dd, *J*_1_ = 8.4 Hz, *J*_2_ = 2.0 Hz, 1H), 6.39 (d, *J* = 8.4 Hz, 1H), 6.27 (br, 1H), 3.64 (s, 3H), 3.58 (s, 3H), 2.26–2.42 (m, 1H), 2.05 (s, 3H), 1.63–1.79 (m, 1H), 1.31 (s, 3H), 0.60–0.89 (m, 3H); ^13^C{^1^H} NMR (100 MHz, DMSO-*d*_6_) δ 165.3, 160.0, 156.9, 148.1, 134.8, 126.5, 124.9, 120.3, 108.6, 99.5, 93.3, 59.2, 52.1, 49.9, 17.9, 15.1, 11.9, 9.0; IR (nujol): υ_max_ = 3360, 3266, 1739, 1694 cm^–1^; HRMS (ESI) calcd for C_18_H_23_N_3_O_4_Cl [M + H]^+^: 380.1377; found: 380.1374.

#### (3aR*,8aS*)-Methyl 3a-Ethyl-5-methoxy-1-((methoxycarbonyl)amino)-2,8a-dimethyl-1,3a,8,8a-tetrahydropyrrolo[2,3-*b*]indole-3-carboxylate (**5k**)

The product **5k** was isolated by column chromatography (ethyl acetate/cyclohexane 40:60) in 98% yield (147.2 mg); white solid; mp: 153–155 °C; ^1^H NMR (400 MHz, DMSO-*d*_6_) δ 9.28 (s, 1H), 6.87 (d, *J* = 2.4 Hz, 1H), 6.49 (dd, *J*_1_ = 8.0 Hz, *J*_2_ = 2.4 Hz, 1H), 6.34 (d, *J* = 8.0 Hz, 1H), 5.64 (s, 1H), 3.64 (s, 3H), 3.63 (s, 3H), 3.58 (s, 3H), 2.27–2.45 (m, 1H), 2.06 (s, 3H), 1.60–1.79 (m, 1H), 1.30 (s, 3H), 0.69–0.91 (m, 3H); ^13^C{^1^H} NMR (100 MHz, DMSO-*d*_6_) δ 166.1, 160.5, 157.6, 152.5, 143.6, 134.6, 112.5, 112.3, 108.5, 100.3, 93.9, 59.9, 55.8, 52.5, 50.3, 25.0, 18.5, 12.4, 9.6; IR (nujol): υ_max_ = 3369, 3267, 1754, 1693 cm^–1^; HRMS (ESI) calcd for C_19_H_26_N_3_O_5_[M + H]^+^: 376.1872; found: 376.1837.

#### (5aS*,10aR*)-Methyl 2-Methoxy-13-((methoxycarbonyl)amino)-12-methyl-5,6,7,8,9,10-hexahydro-5a,10a-(epiminoetheno)cyclohepta[*b*]indole-11-carboxylate (**5l**)

The product **5l** was isolated by column chromatography on silica gel (ethyl acetate/cyclohexane 30:70) in 60% yield (96.4 mg); brown solid; mp: 166–168 °C; ^1^H NMR (400 MHz, CDCl_3_) δ 7.14 (d, *J* = 2.8 Hz, 1H), 6.72 (br, 1H), 6.57 (dd, *J*_1_ = 8.4 Hz, *J*_2_ = 2.8 Hz, 1H), 6.49 (d, *J* = 8.4 Hz, 1H), 3.77 (s, 3H), 3.73 (s, 3H), 3.69 (s, 3H), 3.62 (br, 1H), 2.16 (s, 3H), 1.42 (s, 10H); ^13^C{^1^H} NMR (100 MHz, CDCl_3_) δ 166.2, 160.1, 156.9, 154.3, 140.7, 137.3, 113.1, 112.0, 110.2, 103.7, 95.9, 63.2, 55.9, 52.9, 50.3, 35.8, 33.9, 30.8, 27.0, 25.5, 12.9; IR (nujol): υ_max_ = 3392, 3317, 1738, 1691 cm^–1^; MS (ESI) *m*/*z* = 332 [M + H]^+^; anal. calcd for C_21_H_27_N_3_O_5_ (401.45): C 62.83, H 6.78, N 10.47; found: C 62.98, H 6.70, N 10.36.

#### (3aR*,8aS*)-Ethyl 1-((Ethoxycarbonyl)amino)-2,3a,5,8a-tetramethyl-1,3a,8,8a-tetrahydropyrrolo[2,3-*b*]indole-3-carboxylate (**5m**)

The product **5m** was isolated by column chromatography (ethyl acetate/cyclohexane 30:70) in 47% yield (70.2 mg); white solid; mp: 151–153 °C; ^1^H NMR (400 MHz, DMSO-*d*_6_) δ 9.19 (s, 1H), 7.13 (d, *J* = 1.2 Hz, 1H), 6.69 (dd, *J*_1_ = 8.0 Hz, *J*_2_ = 1.2 Hz, 1H), 6.33 (d, *J* = 8.0 Hz, 1H), 5.88 (s, 1H), 3.98–4.20 (m, 4H), 2.15 (s, 3H), 2.02 (s, 3H), 1.44 (s, 3H), 1.15–1.31 (m, 9H); ^13^C{^1^H} NMR (100 MHz, DMSO-*d*_6_) δ 165.2, 159.2, 156.5, 146.3, 133.8, 127.1, 125.5, 125.4, 107.7, 102.3, 93.0, 60.6, 58.1, 55.3, 20.6, 19.7, 18.4, 14.4, 14.3, 11.9; IR (nujol): υ_max_ = 3343, 3306, 1734, 1689 cm^–1^; HRMS (ESI) calcd for C_20_H_28_N_3_O_4_ [M + H]^+^: 374.2080; found: 374.2091.

#### (3aR*,8aS*)-*tert*-Butyl 1-((*tert*-Butoxycarbonyl)amino)-2,3a,8a-trimethyl-1,3a,8,8a-tetrahydropyrrolo[2,3-*b*]indole-3-carboxylate (**5n**)

The product **5n** was isolated by column chromatography (ethyl acetate/cyclohexane 20:50) in 50% yield (85.9 mg); whitish oil; ^1^H NMR (400 MHz, DMSO-*d*_6_) δ 8.88 (s, 1H), 7.17 (d, *J* = 8.0 Hz, 1H), 6.69 (dd, *J*_1_ = 8.0 Hz, *J*_2_ = 0.8 Hz, 1H), 6.32 (d, *J* = 8.0 Hz, 1H), 5.86 (s, 1H), 2.16 (s, 3H), 1.97 (s, 3H), 1.49 (s, 3H), 1.46 (s, 9H), 1.43 (s, 9H), 1.23 (s, 3H); ^13^C{^1^H} NMR (100 MHz, DMSO-*d*_6_) δ 165.1, 158.8, 155.8, 146.4, 134.4, 127.0, 125.4, 124.8, 107.8, 103.3, 92.9, 79.3, 77.9, 55.3, 28.4, 27.9, 20.6, 19.8, 18.3, 11.9; IR (nujol): υ_max_ = 3439, 3304, 1738, 1696 cm^–1^; MS (ESI) *m*/*z* = 430 [M + H]^+^; anal. calcd for C_24_H_35_N_3_O_4_ (429.52): C 67.11, H 8.21, N 9.78; found: C 67.26, H 8.12, N 9.69.

#### (3aR*,8aS*)-Methyl 1-((Methoxycarbonyl)amino)-3a,5,8a-trimethyl-2-propyl-1,3a,8,8a-tetrahydropyrrolo[2,3-*b*]indole-3-carboxylate (**5o**)

The product **5o** was isolated by column chromatography (ethyl acetate/cyclohexane 20:80) in 74% yield (110.6 mg); white solid; mp: 163–165 °C; ^1^H NMR (400 MHz, CDCl_3_) δ 7.30 (s, 1H), 6.82 (d, *J* = 8.0 Hz, 1H), 6.49 (d, *J* = 8.0 Hz, 1H), 6.31 (br, 1H), 3.80 (br, 1H), 3.79 (s, 3H), 3.68 (s, 3H), 3.47 (q, *J* = 7.2 Hz, 1H), 2.42–2.64 (m, 2H), 2.28 (s, 3H), 1.61 (s, 3H), 1.40 (s, 3H), 1.21 (t, *J* = 7.2 Hz, 1H), 0.92 (t, *J* = 7.2 Hz, 3H); ^13^C{^1^H} NMR (100 MHz, CDCl_3_) δ 165.9, 162.4, 156.9, 145.3, 134.4, 129.3, 128.0, 126.7, 109.4, 106.5, 93.7, 65.9, 56.4, 50.3, 28.1, 21.1, 20.1, 19.5, 15.4, 14.3; IR (nujol): υ_max_ = 3394, 3272, 1729, 1691 cm^–1^; HRMS (ESI) calcd for C_20_H_28_N_3_O_4_ [M + H]^+^: 374.2080; found: 374.2091.

#### (7aS*,10aR*)-Methyl 8-((Methoxycarbonyl)amino)-7a,9,10a-trimethyl-7,7a,8,10a-tetrahydrobenzo[*e*]pyrrolo[2,3-*b*]indole-10-carboxylate (**5p**)

The product **5p** was isolated by column chromatography (ethyl acetate/cyclohexane 20:80) in 38% yield (58.1 mg); white solid; mp: 137–139 °C; ^1^H NMR (400 MHz, CDCl_3_) δ 7.83 (d, *J* = 8.0 Hz, 1H), 7.73 (d, *J* = 8.0 Hz, 1H), 7.61 (d, *J* = 8.4 Hz, 1H), 7.41 (t, *J* = 7.6 Hz, 1H), 7.18 (t, *J* = 7.6 Hz, 1H), 6.93 (d, *J* = 8.4 Hz, 1H), 6.80 (s, 1H), 5.26 (s, 1H), 3.86 (s, 3H), 3.72 (s, 3H), 2.06 (s, 3H), 1.79 (s, 3H), 1.63 (s, 3H); ^13^C{^1^H} NMR (100 MHz, CDCl_3_) δ 166.8, 159.5, 157.1, 148.2, 131.3, 130.9, 129.8, 129.5, 126.9, 121.6, 121.5, 120.5, 113.7, 104.5, 79.8, 65.9, 53.4, 50.6, 21.2, 17.4, 12.5; IR (nujol): υ_max_ = 1732, 1730 cm^–1^; MS (ESI) *m*/*z* = 414 [M + H]^+^; anal. calcd for C_21_H_23_N_3_O_4_ (381.42): C 66.13, H 6.08, N 11.02; found: C 65.98, H 6.16, N 11.16.

#### (3aR*,8aS*)-*N*,*N*,2,3a,8a-Pentamethyl-1-ureido-1,3a,8,8a-tetrahydropyrrolo[2,3-*b*]indole-3-carboxamide (**5q**)

The product **5q** was isolated by column chromatography (methanol/ethyl acetate 05:95) in 52% yield (68.5 mg); white solid; mp: 191–193 °C; ^1^H NMR (400 MHz, DMSO-*d*_6_) δ 7.04 (br, 1H), 6.89 (t, *J* = 7.2 Hz, 1H), 6.77 (d, *J* = 8.0 Hz, 1H), 6.53 (t, *J* = 7.2 Hz, 1H), 6.44 (d, *J* = 8.0 Hz, 1H), 6.16 (br, 2H), 6.04 (s, 1H), 2.73 (s, 3H), 2.32 (s, 3H), 1.58 (s, 3H), 1.46 (s, 3H), 1.25 (s, 3H); ^13^C{^1^H} NMR (100 MHz, DMSO-*d*_6_) δ 167.2, 159.9, 148.9, 142.7, 132.3, 127.0, 121.8, 117.3, 109.8, 107.9, 92.6, 56.9, 20.7, 18.9, 18.0, 11.0; IR (nujol): υ_max_ = 3489, 3337, 3326, 3297, 1698, 1689 cm^–1^; HRMS (ESI) calcd for C_17_H_24_N_5_O_2_[M + H]^+^: 330.1930; found: 330.1932.

#### Methyl ((4bS*,8aS*)-12-(Dimethoxyphosphoryl)-11-methyl-6,7,8,9-tetrahydro-5*H*-8a,4b-(epiminoetheno)carbazol-10-yl)carbamate (**5r**)

The product **5r** was isolated by column chromatography (ethyl acetate/cyclohexane 90:10) in 31% yield (50.6 mg); white solid; mp: 198–200 °C; ^1^H NMR (400 MHz, DMSO-*d*_6_) δ 9.14 (s, 1H), 7.20 (d, *J* = 7.6 Hz, 1H), 6.91 (t, *J* = 7.6, 1H), 6.59 (t, *J* = 7.6, 1H), 6.46 (d, *J* = 7.6 Hz, 1H), 6.01 (s, 1H), 3.62 (s, 3H), 3.35 (s, 3H), 3.18 (s, 3H), 1.91 (s, 3H), 1.75–1.96 (m, 1H), 0.91–1.54 (m, 7H); ^13^C{^1^H} NMR (100 MHz, DMSO-*d*_6_) δ 160.0, 157.0, 149.7, 131.5, 127.0, 124.1, 117.4, 108.4, 97.0, 91.7, 59.7, 56.1 (^2^*J*_CP_ = 10.1 Hz), 51.9, 50.9 (^2^*J*_CP_ = 4.3 Hz), 31.5, 26.3, 20.7, 19.3, 11.6; IR (nujol): υ_max_ = 3319, 3283, 1695 cm^–1^; MS (ESI) *m*/*z* = 408 [M + H]^+^; anal. calcd for C_19_H_26_N_3_O_5_P (407.40): C 56.01, H 6.43, N 10.31; found: C 56.16, H 6.35, N 10.18.

#### (3aR*,8aS*)-Methyl 1-((Methoxycarbonyl)amino)-2,3a,8,8a-tetramethyl-1,3a,8,8a-tetrahydropyrrolo[2,3-*b*]indole-3-carboxylate (**5s**)

The product **5s** was isolated by column chromatography (ethyl acetate/cyclohexane 25:75) in 94% yield (129.9 mg); white solid; mp: 155–157 °C. Notably, compound 5q at NMR analysis shows two sets of peaks. This fact is probably ascribable to the presence of a second axis along the N–N bond that determines the existence of syn/anti rotamers of carbamates.^9,30^^1^H NMR (400 MHz, DMSO-*d*_6_) δ 9.59 and 9.38 (s, 1H), 7.32 and 7.29 (d, *J* = 7.6 Hz, 1H), 7.01 and 6.96 (dt, *J*_1_ = 7.6 Hz, *J*_2_ = 1.2 Hz, 1H), 6.59 and 6.56 (dt, *J*_1_ = 7.6 Hz, *J*_2_ = 1.2 Hz, 1H), 6.37 and 6.33 (d, *J* = 7.6 Hz, 1H), 3.67 and 3.66 (s, 3H), 3.63 and 3.59 (s, 3H), 2.75 and 2.69 (s, 3H), 1.99 and 1.95 (s, 3H), 1.45 and 1.39 (s, 3H), 1.30 and 1.26 (s, 3H); ^13^C{^1^H} NMR (100 MHz, DMSO-*d*_6_) δ 166.1 and 165.5, 160.9 and 158.8, 157.0 and 156.4, 149.4 and 148.8, 134.4 and 133.1, 127.4 and 127.2, 124.2 and 123.4, 117.5 and 117.1, 105.7 and 104.9, 102.8 and 102.5, 95.5 and 95.1, 55.6 and 55.0, 52.3 and 52.1, 50.1 and 49.8, 29.8 and 27.9, 21.1 and 19.8, 14.2 and 13.6, 12.0 and 11.8; IR (nujol): υ_max_ = 3369, 1741, 1693 cm^–1^; MS (ESI) *m*/*z* = 346 [M + H]^+^; anal. calcd for C_18_H_23_N_3_O_4_ (345.39): C 62.59, H 6.71, N 12.17; found: C 62.43, H 6.80, N 12.31.

#### Ethyl 3-(2-Carbamoylhydrazono)-2-(1-methyl-1*H*-indol-3-yl)butanoate (**4a**)

The product **4a** was isolated by column chromatography (ethyl acetate/cyclohexane 80:20) in 48% yield (151.8 mg); white solid; mp: 189–191 °C, ^1^H NMR (400 MHz, CDCl_3_) δ 8.73 (br, 1H), 7.56 (d, *J* = 7.6 Hz, 1H), 7.30 (d, *J* = 7.6 Hz, 1H), 7.23 (t, *J* = 7.6 Hz, 1H), 7.08–7.13 (m, 2H), 5.99 (br, 1H), 5.67 (br, 1H), 4.87 (s, 1H), 4.23 (q, *J* = 7.2 Hz, 2H), 3.76 (s, 3H), 1.86 (s, 3H), 1.29 (s, *J* = 7.8 Hz, 3H); ^13^C{^1^H} NMR (100 MHz, CDCl_3_) δ 171.0, 158.2, 147.4, 137.0, 128.0, 127.2, 121.9, 119.5, 119.2, 109.4, 108.1, 61.2, 52.0, 32.8, 14.2, 13.9; IR (nujol): υ_max_ = 3502, 3387, 3177, 1738, 1693 cm^–1^; MS (ESI) *m*/*z* = 317 [M + H]^+^; anal. calcd for C_16_H_20_N_4_O_3_ (316.35): C 60.75, H 6.37, N 17.71; found: C 60.61, H 6.25, N 17.82.

#### Methyl 2-(4-Methoxy-3-(1-methyl-1*H*-indol-3-yl)-4-oxobutan-2-ylidene)hydrazinecarboxylate (**4b**)

The product **4b** was isolated by column chromatography (ethyl acetate/cyclohexane 40:60) in 70% yield (222.2 mg); white solid; mp: 189–191 °C, ^1^H NMR (400 MHz, DMSO-*d*_6_) δ 9.88 (s, 1H), 7.45 (d, *J* = 8.0 Hz, 1H), 7.41 (d, *J* = 8.0 Hz, 1H), 7.33 (s, 1H), 7.16 (dt, *J*_1_ = 8.0 Hz, *J*_2_ = 1.2 Hz, 1H), 7.03 (dt, *J*_1_ = 8.0 Hz, *J*_2_ = 1.2 Hz, 1H), 4.87 (s, 1H), 3.77 (s, 3H), 3.68 (s, 6H), 1.79 (s, 3H); ^13^C{^1^H} NMR (100 MHz, DMSO-*d*_6_) δ 171.6, 155.1, 151.5, 137.0, 129.0, 127.3, 121.8, 119.5, 119.2, 110.3, 108.0, 52.4, 52.3, 51.8, 32.9, 14.9; IR (nujol): υ_max_ = 3354, 1740, 1726, 1696 cm^–1^; MS (ESI) *m*/*z* = 318 [M + H]^+^; anal. calcd for C_16_H_19_N_3_O_4_ (317.33): C 60.56, H 6.03, N 13.24; found: C 60.42, H 6.12, N 13.31.

#### Methyl 2-(3-(1,2-Dimethyl-1*H*-indol-3-yl)-4-methoxy-4-oxobutan-2-ylidene)hydrazinecarboxylate (**4c**)

The product **4c** was isolated by column chromatography (ethyl acetate/cyclohexane 30:70) in 52% yield (172.3 mg); white solid; mp: 223–225 °C, ^1^H NMR (400 MHz, DMSO-*d*_6_) δ 9.84 (s, 1H), 7.35–7.42 (m, 2H), 7.08 (dt, *J*_1_ = 8.0 Hz, *J*_2_ = 1.2 Hz, 1H), 6.97 (dt, *J*_1_ = 8.0 Hz, *J*_2_ = 1.2 Hz, 1H), 4.85 (s, 1H), 3.68 (s, 3H), 3.66 (s, 3H), 3.63 (s, 3H), 2.34 (s, 3H), 1.75 (s, 3H); ^13^C{^1^H} NMR (100 MHz, DMSO-*d*_6_) δ 172.1, 155.0, 152.3, 136.7, 136.1, 127.0, 120.7, 119.4, 118.6, 109.7, 104.6, 52.2, 52.1, 51.5, 29.9, 15.5, 10.6; IR (nujol): υ_max_ = 3365, 1743, 1732, 1701 cm^–1^; MS (ESI) *m*/*z* = 332 [M + H]^+^; anal. calcd for C_17_H_21_N_3_O_4_ (331.36): C 61.62, H 6.39, N 12.68; found: C 61.57, H 6.50, N 12.52.

#### Ethyl 2-(2-Carbamoylhydrazono)-1-(1,2-dimethyl-1*H*-indol-3-yl)cyclohexanecarboxylate (**4d**)^10e^

The product **4d** was isolated by column chromatography on silica gel (ethyl acetate/cyclohexane 60:40) in 61% yield (113.0 mg); white solid; mp: 207–210 °C; ^1^H NMR (400 MHz, DMSO-*d*_6_) δ 9.57 (s, 1H), 7.37 (t, *J* = 9.2 Hz, 2H), 7.04 (dt, *J*_1_ = 8.0 Hz, *J*_2_ = 0.8 Hz, 1H), 6.91 (dt, *J*_1_ = 8.0 Hz, *J*_2_ = 0.8 Hz, 1H), 5.84 (br, 2H), 3.94–4.13 (m, 2H), 3.64 (s, 3H), 2.67–2.91 (m, 2H), 2.25 (s, 3H), 2.14–2.23 (m, 2H), 1.38–1.59 (m, 4H), 1.09 (t, *J* = 7.2 Hz, 3H); ^13^C{^1^H} NMR (100 MHz, DMSO-*d*_6_) δ 173.2, 157.4, 152.6, 136.2, 135.0, 126.3, 119.8, 119.4, 118.6, 109.3, 108.1, 60.3, 56.9, 35.7, 29.3, 25.5, 24.8, 21.5, 13.9, 11.6; IR (nujol): υ_max_ = 3510, 3393, 3182, 1734, 1687 cm^–1^; MS (ESI) *m*/*z* = 371 [M + H]^+^; anal. calcd for C_20_H_26_N_4_O_3_ (370,45): C 64.84, H 7.07, N 15.12; found: C 64.69, H 6.99, N 14.99.

### Procedure for the Ring-Opening Reaction of Tetrahydro-1*H*-pyridazino[3,4-*b*]indole **(3b)**

To a solution of compound **3b** (0.4 mmol) in dichloromethane (2 mL), Amberlyst 15(H) (500 mg/mmol) was added. After the disappearance of starting **3b** (TLC check, 20 h), the crude mixture was purified by column chromatography on silica gel to afford product **4e**.

#### Ethyl 1-(1-Methyl-1*H*-indol-3-yl)-2-(2-(phenylcarbamoyl)hydrazono)cyclohexanecarboxylate (**4e**)

The product **4e** was isolated by column chromatography (ethyl acetate/cyclohexane 25:75) in 71% yield (122.8 mg); white solid; mp: 210–212 °C, ^1^H NMR (400 MHz, DMSO-*d*_6_) δ 10.02 (s, 1H), 7.51 (t, *J* = 8.4 Hz, 2H), 7.33 (s, 1H), 7.25 (s, 1H), 7.18 (dt, *J*_1_ = 8.4 Hz, *J*_2_ = 1.2 Hz, 1H), 7.07 (t, *J* = 8.4 Hz, 2H), 6.96 (dt, *J*_1_ = 8.4 Hz, *J*_2_ = 1.2 Hz, 1H), 6.85 (dt, *J*_1_ = 8.4 Hz, *J*_2_ = 1.2 Hz, 1H), 6.48 (d, *J* = 8.4 Hz, 2H), 4.05–4.19 (m, 2H), 3.79 (s, 3H), 3.04 (d, *J* = 14.4 Hz, 1H), 2.68 (d, *J* = 14.4 Hz, 1H), 2.11–2.23 (m, 2H), 1.79–1.85 (m, 2H), 1.44–1.55 (m, 2H), 1.13 (t, *J* = 7.2 Hz, 3H); ^13^C{^1^H} NMR (100 MHz, DMSO-*d*_6_) δ 172.5, 153.3, 151.0, 138.1, 137.1, 128.3, 127.5, 126.6, 121.9, 120.9, 120.8, 118.8, 117.5, 113.5, 109.8, 60.7, 55.5, 35.6, 32.4, 25.5, 25.3, 22.6, 13.9; IR (nujol): υ_max_ = 3190, 3088, 1726, 1681 cm^–1^; MS (ESI) *m*/*z* = 433 [M + H]^+^; anal. calcd for C_25_H_28_N_4_O_3_ (432.51): C 69.42, H 6.53, N 12.95; found: C 69.57, H 6.44, N 12.87.

#### Ethyl 2-(2-Carbamoylhydrazono)-1-(1-methyl-1*H*-indol-3-yl)cyclopentanecarboxylate (**4f**)

The product **4f** (see Scheme 1) was isolated by column chromatography (ethyl acetate/cyclohexane 90:10) in 67% yield (229.4 mg); white solid; mp: 168–170 °C; ^1^H NMR (400 MHz, DMSO-*d*_6_) δ 9.14 (s, 1H), 7.44 (d, *J* = 8.0 Hz, 1H), 7.38 (d, *J* = 8.0 Hz, 1H), 7.12 (dt, *J*_1_ = 8.0 Hz, *J*_2_ = 0.8 Hz, 1H), 7.09 (s, 1H), 6.98 (dt, *J*_1_ = 8.0 Hz, *J*_2_ = 0.8 Hz, 1H), 5.92 (br, 2H), 4.04–4.12 (m, 2H), 3.73 (s, 3H), 2.43–2.56 (m, 3H), 2.29–2.36 (m, 1H), 1.62–1.87 (m, 2H), 1.08 (t, *J* = 7.2 Hz, 3H); ^13^C{^1^H} NMR (100 MHz, DMSO-*d*_6_) δ 172.7, 156.9, 156.4, 137.2, 127.5, 126.1, 121.0, 120.2, 118.4, 113.4, 109.8, 60.6, 56.8, 36.2, 32.3, 27.9, 21.4, 14.1; IR (nujol): υ_max_ = 3470, 3200, 3160, 1733, 1696 cm^–1^; MS (ESI) *m*/*z* = 343 [M + H]^+^; anal. calcd for C_18_H_22_N_4_O_3_ (342.39): C 63.14, H 6.48, N 16.36; found: C 62.98, H 6.58, N 16.45.

### Ethyl Bromoacetate-Assisted Cleavage of the N–N Bond in **5s** (Magnus’ Procedure^16^)

Ethyl 2-bromoacetate (1.5 equiv) and Cs_2_CO_3_ (2.5 equiv) were added to a solution of compound **5s** (0.3 mmol) in acetonitrile (2 mL). The reaction mixture was stirred in an oil bath heated at 50 °C until the starting material was consumed (TLC check, 1 h) and then refluxed for an additional 1.5 h (TLC check). The crude mixture was filtered and then purified by column chromatography on silica gel to afford the product **6a**. The NMR experiments show that the title compound has no rotamers.

#### (3aR*,8aR*)-Methyl 2,3a,8,8a-Tetramethyl-1,3a,8,8a-tetrahydropyrrolo[2,3-*b*]indole-3-carboxylate (**6a**)

The product **6a** was isolated by column chromatography (ethyl acetate/cyclohexane 30:70) in 64% yield (52.3 mg); whitish oil; ^1^H NMR (400 MHz, DMSO-*d*_6_) δ 7.45 (s, 1H), 7.26 (dd, *J*_1_ = 7.6 Hz, *J*_2_ = 1.2 Hz, 1H), 6.92 (dt, *J*_1_ = 7.6 Hz, *J*_2_ = 1.2 Hz, 1H), 6.52 (dt, *J*_1_ = 7.6 Hz, *J*_2_ = 1.2 Hz, 1H), 6.30 (dd, *J*_1_ = 7.6 Hz, *J*_2_ = 1.2 Hz, 1H), 3.55 (s, 3H), 2.67 (s, 3H), 2.03 (s, 3H), 1.39 (s, 3H), 1.27 (s, 3H); ^13^C{^1^H} NMR (100 MHz, DMSO-*d*_6_) δ 165.9, 159.3, 149.3, 134.7, 126.8, 123.8, 116.9, 104.9, 101.6, 90.9, 56.4, 49.3, 28.2, 19.8, 17.4, 14.5; IR (nujol): υ_max_ = 3369, 1741 cm^–1^; MS (ESI) *m*/*z* = 273 [M + H]^+^; anal. calcd for C_16_H_20_N_2_O_2_ (272.34): C 70.56, H 7.40, N 10.29; found: C 70.41, H 7.47, N 10.39.

## Supporting Information Available

Structures of starting materials (Figure S1), optimization for reaction of **1a** with cyclic azoalkene **2a** (Table S1), copies of NMR spectra for all products (PDF)
Crystal data of compounds **3e** (CIF)
Cartesian coordinates of all computed structures for the reaction of **2n** with **1p** (PDF)

## Supplementary Material

jo0c01489_si_001.pdf

jo0c01489_si_002.cif
