# Supplementary material for: Synthesis of Polycyclic Fused Indoline Scaffolds through a Substrate-Guided Reactivity Switch
Source: J Org Chem. 2020 Aug 12;85(17):11409–25. doi: 10.1021/acs.joc.0c01489 (PMC8010796; doi:10.1021/acs.joc.0c01489)

Synthesis of Polycylic Fused Indoline Scaffolds through a Substrate-Guided Reactivity Switch

Cecilia Ciccolini,† Giacomo Mari,† Francesco G. Gatti,‡ Giuseppe Gatti,† Gianluca Giorgi,§ Fabio Mantellini† and Gianfranco Favi*,†

†Department of Biomolecular Sciences, Section of Chemistry and Pharmaceutical Technologies, University of Urbino "Carlo Bo", Via I Maggetti 24, 61029 Urbino, Italy

‡Department of Chemistry, Materials and Chemical Engineering "G. Natta", Piazza Leonardo da Vinci 32, 20133 Milano, Italy

§Department of Biotechnologies, Chemistry & Pharmacy, University of Siena, Via A. Moro 2, 53100 Siena, Italy

e-mail: gianfranco.favi@uniurb.it

Computational Procedures

All the computations were carried out using the programme Gaussian 16, Revision C.01. The reaction path from the reactant state to the end-product state was explored with the aim to find stationary points, *i.e.* local minima (intermediate states) and saddle points (transition states), by optimizing geometry and energy of each state and then by performing a frequency analysis. Saddles were characterized by the presence of one imaginary frequency, whereas minima showed no imaginary frequencies. Moreover, the connection of the transition states with foregoing and following intermediate states was verified with the intrinsic reaction coordinate (IRC) plot. The geometry of all species of the postulated reaction paths were first optimized *in vacuo* at the level B3LYP/631G(d) and then re-computed in dichloromethane solution at level B3LYP/6-31G(d)/SCRF=PCM.

List of Geometry and Energy of Reactants, Catalyst, Intermediates, Transition States and End-Products

[3+2] Step-wise cycloaddition structures

In (1p)

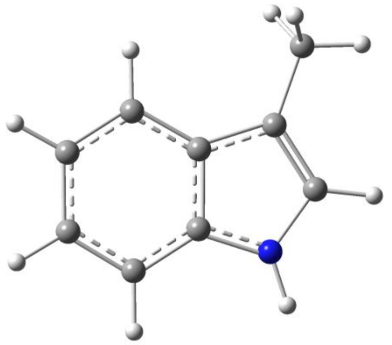

Energy = -403.141196 au

Center Number	Atomic Number	Atomic Type	Coordinates (Angstroms) X	Y	Z
1	6	0	0.404847	0.907827	0.000008
2	6	0	-0.174948	-0.393962	-0.000001
3	6	0	0.675774	-1.514502	-0.000000
4	6	0	2.052647	-1.319826	-0.000011
5	6	0	2.603924	-0.020391	-0.000005
6	6	0	1.790421	1.108431	0.000012
7	1	0	0.261858	-2.520018	0.000012
8	1	0	2.717969	-2.179273	-0.000013
9	1	0	3.683873	0.101348	-0.000011
10	1	0	2.214411	2.109191	0.000034
11	7	0	-0.632676	1.814215	0.000042
12	6	0	-1.834073	1.127741	-0.000021
13	1	0	-2.770749	1.669064	-0.000059
14	6	0	-1.608445	-0.226836	-0.000045
15	1	0	-0.536717	2.818447	-0.000113
16	6	0	-2.641380	-1.314451	0.000025
17	1	0	-2.550386	-1.963199	-0.881300
18	1	0	-2.550661	-1.962923	0.881578
19	1	0	-3.653472	-0.896331	-0.000196

DD (2n)

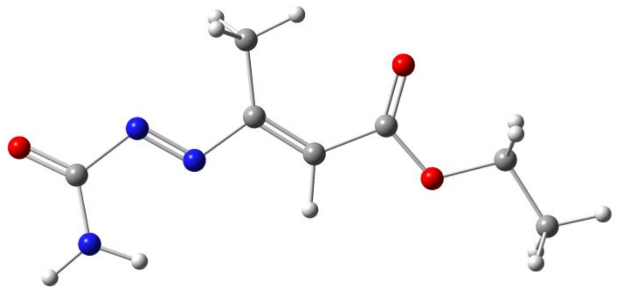

Energy = -663.272421 au

Center Number	Atomic Number	Atomic Type	Coordinates (Angstroms) X	Y	Z
1	6	0	1.984795	0.455099	0.006696
2	8	0	2.314362	1.629172	0.032503
3	8	0	2.865176	-0.563955	-0.017407
4	7	0	-1.693328	-0.210917	-0.003196
5	7	0	-2.787378	0.404001	0.011585
6	6	0	-3.951559	-0.496288	-0.018161
7	8	0	-5.050670	0.018531	-0.155186
8	6	0	-0.542944	0.629403	0.012593
9	6	0	4.270431	-0.202045	-0.012153
10	1	0	4.469380	0.427963	-0.884467
11	1	0	4.472462	0.390867	0.885089
12	6	0	-0.700749	2.117757	0.043766
13	1	0	0.271833	2.604981	0.049556
14	1	0	-1.280388	2.450644	-0.824322
15	1	0	-1.272244	2.415121	0.930047
16	6	0	0.605035	-0.086889	-0.003994
17	1	0	0.523149	-1.168168	-0.027949
18	6	0	5.072631	-1.488034	-0.040531
19	1	0	6.141696	-1.251434	-0.037710
20	1	0	4.854499	-2.105971	0.836261
21	1	0	4.850995	-2.068924	-0.941435
22	7	0	-3.702014	-1.811403	0.135892
23	1	0	-4.462786	-2.469351	0.051038
24	1	0	-2.746338	-2.141497	0.145353

$ZnCl_2$

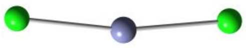

Energy = -2699.652283 au

Center Number	Atomic Number	Atomic Type	Coordinates (Angstroms) X	Y	Z
1	30	0	0.000000	-0.000000	0.102388
2	17	0	0.000000	-2.139757	-0.090342
3	17	0	0.000000	2.139757	-0.090342

***transoid*-DD·$ZnCl_2$**

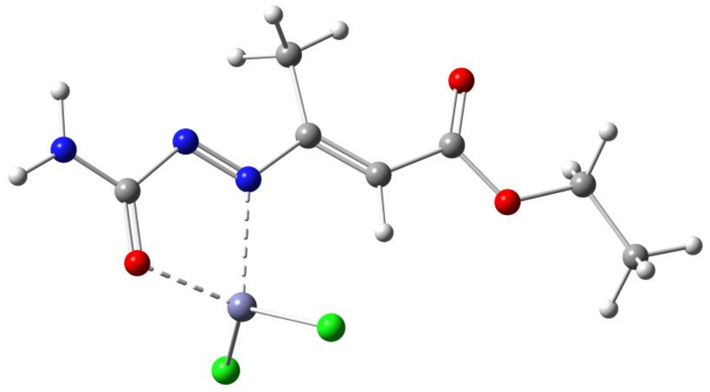

Energy = -3362.968653 au

Center Number	Atomic Number	Atomic Type	Coordinates (Angstroms) X	Y	Z
1	6	0	-2.866421	1.000199	-0.008907
2	8	0	-3.380686	2.092433	0.154424
3	8	0	-3.549333	-0.148653	-0.110909
4	7	0	0.882276	1.030218	-0.092976
5	7	0	1.833089	1.848476	-0.146018
6	6	0	3.112322	1.169648	-0.252594
7	8	0	3.240833	-0.066272	-0.206355
8	6	0	-0.406931	1.607761	0.024924
9	6	0	-4.995688	-0.057094	0.008436
10	1	0	-5.230399	0.404535	0.972137
11	1	0	-5.363887	0.602141	-0.783220
12	6	0	-0.515800	3.080656	0.265422
13	1	0	-1.557617	3.361421	0.400805
14	1	0	0.066966	3.361270	1.149269
15	1	0	-0.095733	3.632063	-0.582973
16	6	0	-1.410023	0.706873	-0.111466
17	1	0	-1.169769	-0.327994	-0.326578
18	6	0	-5.551377	-1.462068	-0.106381
19	1	0	-6.642070	-1.429181	-0.016341
20	1	0	-5.298252	-1.906670	-1.073796
21	1	0	-5.159101	-2.104677	0.687919
22	7	0	4.105005	2.033036	-0.400993
23	1	0	5.058713	1.705974	-0.492998
24	1	0	3.916599	3.027207	-0.426913
25	30	0	1.504069	-1.079423	0.040022
26	17	0	1.239612	-1.682866	2.190850
27	17	0	0.756087	-2.059571	-1.828611

In·DD·$ZnCl_2$

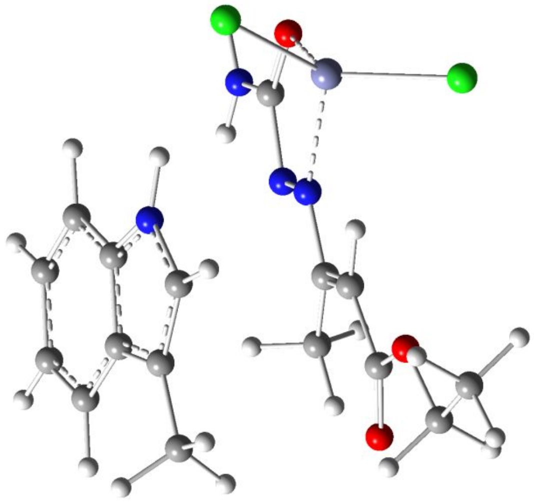

Energy = -3766.118574 au

Center Number	Atomic Number	Atomic Type	Coordinates (Angstroms) X	Y	Z
1	6	0	2.634099	-0.673894	-1.067711
2	8	0	3.232102	0.021218	-1.872604
3	8	0	3.224230	-1.605871	-0.300347
4	7	0	-1.080329	-0.193953	-1.030861
5	7	0	-1.977044	0.489812	-1.614371
6	6	0	-3.293450	0.116007	-1.186901
7	8	0	-3.530546	-0.835669	-0.413938
8	6	0	0.233261	0.066064	-1.445309
9	6	0	4.652913	-1.790615	-0.487069
10	1	0	4.835669	-2.016403	-1.541772
11	1	0	5.157041	-0.849135	-0.249508
12	6	0	0.457786	1.056100	-2.546778
13	1	0	0.069216	2.037444	-2.257138
14	1	0	1.519143	1.132488	-2.770381
15	1	0	-0.088737	0.745118	-3.444150
16	6	0	1.176682	-0.662879	-0.784555
17	1	0	0.859461	-1.374788	-0.032963
18	6	0	5.088284	-2.920144	0.424822
19	1	0	6.163832	-3.088342	0.307433
20	1	0	4.889901	-2.677440	1.473506
21	1	0	4.564698	-3.848337	0.175763
22	7	0	-4.230280	0.887348	-1.724977
23	1	0	-5.206825	0.728838	-1.513475
24	1	0	-3.971494	1.643990	-2.344607
25	7	0	0.175861	1.111823	1.915458
26	1	0	-0.492757	0.466932	2.330486
27	6	0	2.140081	1.922047	1.129657
28	6	0	1.531678	0.909023	1.855631
29	1	0	1.984189	0.067232	2.362074
30	6	0	3.605403	2.091963	0.871619
31	1	0	4.191034	1.342527	1.413806
32	1	0	3.840432	1.990149	-0.195745

33	1	0	3.954989	3.082737	1.187972
34	6	0	1.078349	2.790810	0.699688
35	6	0	-0.141131	2.249860	1.200282
36	6	0	1.037435	3.973229	-0.064681
37	1	0	1.953488	4.410115	-0.453604
38	6	0	-0.191904	4.568727	-0.318574
39	1	0	-0.238240	5.479740	-0.908636
40	6	0	-1.388410	4.009577	0.183119
41	1	0	-2.334404	4.500579	-0.027937
42	6	0	-1.380482	2.848978	0.952939
43	1	0	-2.299703	2.429146	1.351277
44	17	0	-0.965718	-3.655784	-0.374550
45	17	0	-2.014272	-1.354978	2.562813
46	30	0	-1.858614	-1.701713	0.310614

[TS1]‡

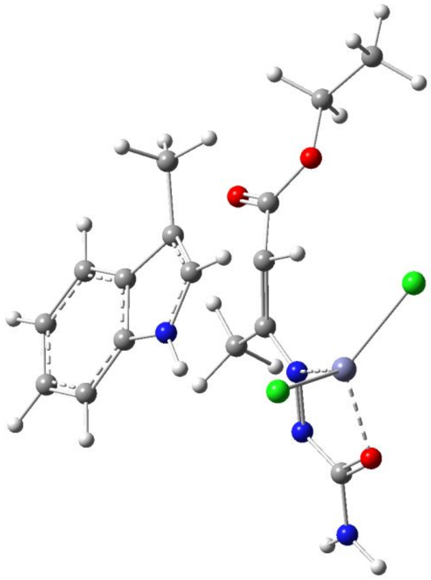

Energy = -3766.101435 au   Imaginary frequency = 294i cm^{-1}

Center Number	Atomic Number	Atomic Type	Coordinates (Angstroms) X	Y	Z
1	6	0	-2.364149	-1.130256	0.811479
2	8	0	-3.194710	-0.557793	1.492350
3	8	0	-2.476675	-2.402223	0.403796
4	7	0	1.062384	0.109079	1.031383
5	7	0	1.812373	0.755192	1.918672
6	6	0	3.147055	0.463440	1.780524
7	8	0	3.650959	-0.338135	0.931625
8	6	0	-0.256402	0.238230	1.180983
9	6	0	-3.657497	-3.126112	0.854073
10	1	0	-3.668498	-3.114689	1.947550
11	1	0	-4.544560	-2.593512	0.498964
12	6	0	-0.795727	1.105278	2.283508
13	1	0	-0.450150	2.137119	2.155503
14	1	0	-1.881974	1.083173	2.308370
15	1	0	-0.398426	0.764700	3.247008
16	6	0	-1.076288	-0.547142	0.288849
17	1	0	-0.517971	-1.289463	-0.272423
18	6	0	-3.569222	-4.531448	0.294780
19	1	0	-4.442301	-5.106422	0.620532
20	1	0	-3.553606	-4.518776	-0.799518
21	1	0	-2.668218	-5.039662	0.651543
22	7	0	3.947856	1.111460	2.646300
23	1	0	4.932426	0.892166	2.675313
24	1	0	3.543709	1.679433	3.376328
25	7	0	0.051043	1.625962	-1.721828
26	1	0	1.034413	1.705565	-1.992013
27	6	0	-1.910147	0.585360	-1.269676
28	6	0	-0.655631	0.533578	-1.964717
29	1	0	-0.240984	-0.278243	-2.547084

30	6	0	-3.128858	-0.147252	-1.776810
31	1	0	-2.884922	-1.165756	-2.089280
32	1	0	-3.912722	-0.188775	-1.016559
33	1	0	-3.538119	0.387189	-2.641635
34	6	0	-1.957763	1.953942	-0.724524
35	6	0	-0.704358	2.548818	-0.973525
36	6	0	-2.939372	2.684197	-0.052756
37	1	0	-3.914376	2.253723	0.151502
38	6	0	-2.632525	3.982648	0.360008
39	1	0	-3.383957	4.569032	0.879876
40	6	0	-1.368150	4.545321	0.118711
41	1	0	-1.159956	5.556062	0.455910
42	6	0	-0.374829	3.834827	-0.558793
43	1	0	0.598479	4.269447	-0.761480
44	17	0	1.667571	-3.107337	-0.645664
45	17	0	2.831954	0.153003	-2.314043
46	30	0	2.269026	-0.952195	-0.339664

Zw·$ZnCl_2$

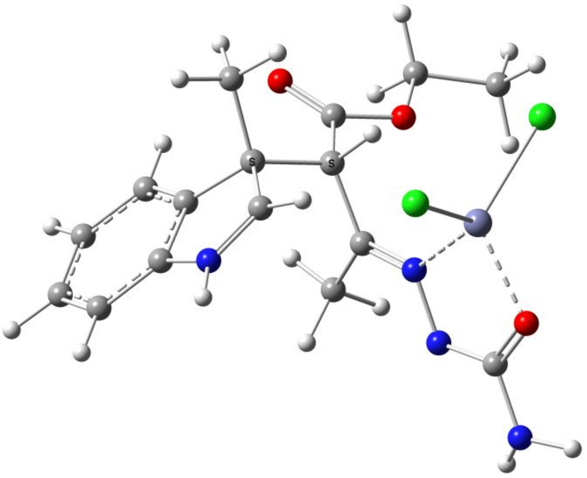

Energy = -3766.117799 au

Center Number	Atomic Number	Atomic Type	Coordinates (Angstroms) X	Y	Z
1	6	0	1.162403	2.210161	-0.357589
2	8	0	2.249146	2.629221	-0.707295
3	8	0	0.192104	2.973020	0.149654
4	7	0	-1.001945	-0.301559	0.948990
5	7	0	-1.412644	-0.733919	2.182719
6	6	0	-2.652322	-1.247565	2.149158
7	8	0	-3.428017	-1.335158	1.126791
8	6	0	0.161447	0.275729	0.904125
9	6	0	0.470027	4.398914	0.269320
10	1	0	1.359318	4.523148	0.893576
11	1	0	0.693991	4.786449	-0.728470
12	6	0	0.977390	0.490629	2.149466
13	1	0	1.223003	-0.466684	2.623180
14	1	0	1.905231	1.028042	1.943171
15	1	0	0.390398	1.053391	2.884171
16	6	0	0.677529	0.755075	-0.445084
17	1	0	-0.173896	0.792531	-1.133286
18	6	0	-0.756098	5.049398	0.876107
19	1	0	-0.585143	6.126857	0.970665
20	1	0	-1.635148	4.892129	0.243891
21	1	0	-0.963747	4.644083	1.871259
22	7	0	-3.107577	-1.765945	3.332001
23	1	0	-4.110646	-1.860716	3.414744
24	1	0	-2.622546	-1.469293	4.168314
25	7	0	1.869648	-2.466131	-0.584431
26	1	0	1.644032	-3.458126	-0.533117
27	6	0	1.731818	-0.214734	-1.099107
28	6	0	1.109274	-1.584432	-1.152624
29	1	0	0.156402	-1.854261	-1.607695
30	6	0	2.041736	0.196273	-2.572864
31	1	0	1.113921	0.297196	-3.143508
32	1	0	2.566212	1.151624	-2.567716

33	1	0	2.673793	-0.553578	-3.056621
34	6	0	3.010348	-0.514945	-0.316471
35	6	0	3.054347	-1.889260	-0.047048
36	6	0	4.089971	0.269388	0.078563
37	1	0	4.099450	1.333505	-0.121191
38	6	0	5.155332	-0.347897	0.747977
39	1	0	5.998720	0.256742	1.066971
40	6	0	5.159873	-1.722741	1.012528
41	1	0	6.000869	-2.169975	1.532430
42	6	0	4.094467	-2.531275	0.608137
43	1	0	4.084075	-3.600149	0.793668
44	17	0	-3.210827	1.341002	-1.383840
45	17	0	-2.170374	-2.262634	-2.055760
46	30	0	-2.526712	-0.620175	-0.452565

[TS2]‡

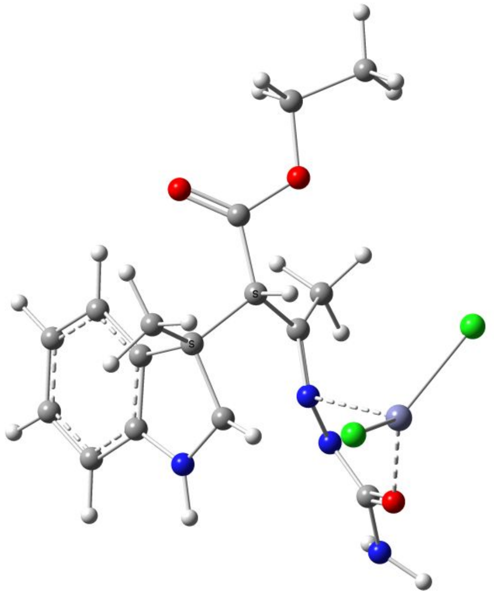

Energy = -3766.099390 au   Imaginary frequency = 147i cm^{-1}

Center Number	Atomic Number	Atomic Type	Coordinates (Angstroms) X	Y	Z
1	6	0	-1.687316	2.194371	0.308326
2	8	0	-2.834050	2.280065	0.701997
3	8	0	-1.004997	3.207784	-0.235698
4	7	0	0.467450	-0.648719	-0.697393
5	7	0	0.801735	-1.485682	-1.731055
6	6	0	1.960364	-2.110935	-1.529707
7	8	0	2.829217	-1.854838	-0.607122
8	6	0	-0.245952	0.412315	-0.902394
9	6	0	-1.708933	4.476679	-0.372141
10	1	0	-2.600778	4.310880	-0.983035
11	1	0	-2.030136	4.795817	0.623231
12	6	0	-0.554589	0.982490	-2.245695
13	1	0	-0.210385	0.301442	-3.024000
14	1	0	-1.631385	1.157591	-2.355500
15	1	0	-0.056473	1.951967	-2.359370
16	6	0	-0.790799	0.963895	0.417266
17	1	0	0.073958	1.317923	0.997647
18	6	0	-0.749652	5.461302	-1.009180
19	1	0	-1.246159	6.430983	-1.119521
20	1	0	0.141250	5.598168	-0.388709
21	1	0	-0.436084	5.120110	-2.000736
22	7	0	2.239843	-3.157861	-2.365823
23	1	0	3.220041	-3.371343	-2.493564
24	1	0	1.666347	-3.231691	-3.195840
25	7	0	-0.897992	-2.523196	0.784977
26	1	0	-0.411991	-3.411980	0.785845
27	6	0	-1.388456	-0.263334	1.158085
28	6	0	-0.323150	-1.362920	1.090515

29	1	0	0.588747	-1.347266	1.674429
30	6	0	-1.711772	0.034587	2.642772
31	1	0	-0.831738	0.433622	3.157818
32	1	0	-2.517250	0.769303	2.697102
33	1	0	-2.034349	-0.875440	3.156730
34	6	0	-2.552322	-0.982733	0.456491
35	6	0	-2.208885	-2.334000	0.293861
36	6	0	-3.821888	-0.564793	0.068171
37	1	0	-4.121490	0.467900	0.195040
38	6	0	-4.702383	-1.501995	-0.490380
39	1	0	-5.691140	-1.180406	-0.803708
40	6	0	-4.330926	-2.840714	-0.644910
41	1	0	-5.030580	-3.549759	-1.077157
42	6	0	-3.065112	-3.283186	-0.246917
43	1	0	-2.766503	-4.321101	-0.355593
44	17	0	3.157641	1.822531	-0.614420
45	17	0	2.817402	-0.418254	2.553011
46	30	0	2.714746	-0.176049	0.304650

[3+2]·cycle·$ZnCl_2$

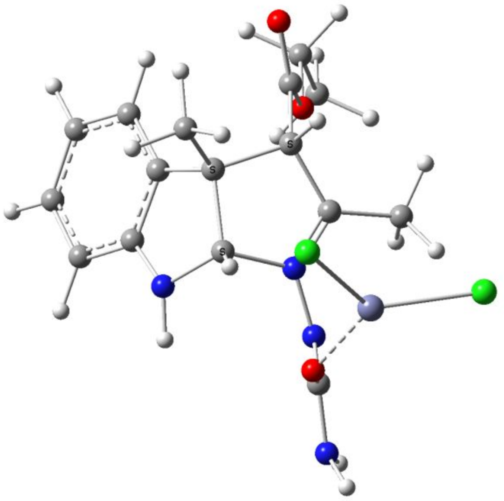

Energy = -3766.123238 au

Center	Atomic	Atomic	Coordinates (Angstroms)		
Number	Number	Type	X	Y	Z
1	6	0	-2.206344	1.710964	1.040072
2	8	0	-2.695153	1.995286	2.114860
3	8	0	-2.746913	1.999824	-0.147324
4	7	0	0.282783	-0.087250	-0.801004
5	7	0	0.838307	-0.270330	-2.044040
6	6	0	2.037083	-0.860791	-2.040864
7	8	0	2.756255	-1.151023	-1.010774
8	6	0	-0.186800	1.056496	-0.406019
9	6	0	-4.026715	2.698326	-0.135035
10	1	0	-4.751662	2.065279	0.383974
11	1	0	-3.906242	3.623532	0.434874
12	6	0	0.055605	2.308200	-1.172273
13	1	0	1.127541	2.413435	-1.371733
14	1	0	-0.456393	2.255300	-2.139946
15	1	0	-0.306745	3.180545	-0.628022
16	6	0	-0.901072	0.930367	0.911232
17	1	0	-0.246995	1.329746	1.697575
18	6	0	-4.418680	2.953324	-1.576137
19	1	0	-5.381985	3.473049	-1.602571
20	1	0	-3.676352	3.579098	-2.081543
21	1	0	-4.519337	2.012863	-2.126796
22	7	0	2.505144	-1.240465	-3.262239
23	1	0	3.500629	-1.393098	-3.342014
24	1	0	2.031470	-0.885329	-4.081704
25	7	0	-0.685302	-2.336055	-0.495158
26	1	0	-0.272501	-2.729190	-1.331656
27	6	0	-1.077654	-0.620060	1.129705
28	6	0	-0.031450	-1.243664	0.151469
29	1	0	0.916043	-1.530318	0.604702
30	6	0	-0.843817	-1.014031	2.595887
31	1	0	0.152391	-0.705676	2.931736
32	1	0	-1.590091	-0.532919	3.235628

33	1	0	-0.931748	-2.097626	2.719468
34	6	0	-2.379019	-1.209567	0.578604
35	6	0	-2.072340	-2.180439	-0.387936
36	6	0	-3.702819	-0.996246	0.949244
37	1	0	-3.955225	-0.277965	1.723404
38	6	0	-4.718912	-1.717903	0.307294
39	1	0	-5.755560	-1.549854	0.583215
40	6	0	-4.399853	-2.654516	-0.679795
41	1	0	-5.193757	-3.207386	-1.174591
42	6	0	-3.070617	-2.905414	-1.036901
43	1	0	-2.822775	-3.649369	-1.788261
44	17	0	3.879649	2.121952	-0.311232
45	17	0	3.462421	-0.882335	2.353654
46	30	0	3.459483	0.026938	0.327493

PI·$ZnCl_2$

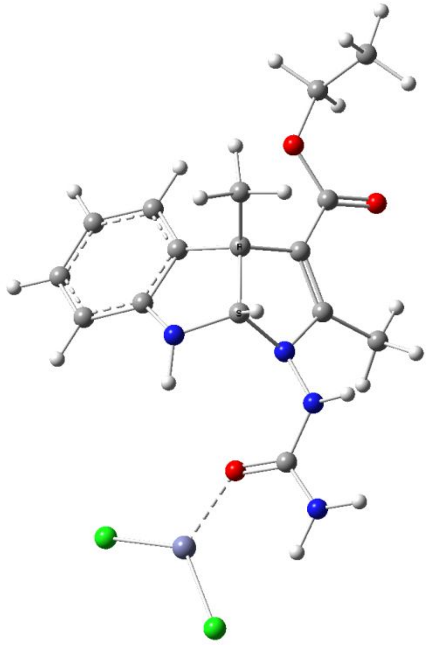

Energy = -3766.131314 au

Center Number	Atomic Number	Atomic Type	Coordinates (Angstroms) X	Y	Z
1	6	0	3.834057	-1.017761	-0.788878
2	8	0	4.098591	-1.819091	-1.678009
3	8	0	4.760186	-0.143659	-0.310609
4	7	0	0.330792	-1.182646	0.315786
5	6	0	-1.978189	-1.812421	0.390641
6	8	0	-2.310552	-0.660814	-0.010754
7	6	0	1.428094	-1.572403	-0.460233
8	6	0	6.092643	-0.219675	-0.875019
9	1	0	6.011967	-0.435459	-1.942176
10	1	0	6.499090	0.785117	-0.739668
11	6	0	1.213707	-2.594990	-1.528673
12	1	0	0.318264	-2.346396	-2.108945
13	1	0	2.083936	-2.640536	-2.179669
14	1	0	1.051804	-3.588604	-1.093118
15	6	0	2.546809	-0.878791	-0.106099
16	6	0	6.939769	-1.261305	-0.159623
17	1	0	7.959545	-1.245703	-0.560552
18	1	0	6.990169	-1.053263	0.914229
19	1	0	6.528214	-2.264072	-0.306454
20	7	0	-2.861765	-2.779626	0.668709
21	1	0	-3.854578	-2.561326	0.639477
22	1	0	-2.579233	-3.707559	0.951316
23	30	0	-4.069998	0.135101	-0.384218
24	17	0	-5.744396	-1.143568	0.403801
25	17	0	-4.049330	2.111790	-1.353473
26	7	0	0.020266	0.889153	1.624433
27	1	0	-0.981552	0.796490	1.490079

28	6	0	2.228251	0.100045	1.033987
29	6	0	0.778380	-0.336239	1.451054
30	1	0	0.748132	-0.942529	2.364063
31	6	0	3.185179	0.047301	2.240337
32	1	0	3.319375	-0.985221	2.583224
33	1	0	4.162948	0.455843	1.982048
34	1	0	2.770414	0.634366	3.067614
35	6	0	1.973463	1.537007	0.565245
36	6	0	0.663313	1.910008	0.902841
37	6	0	2.807818	2.441486	-0.081621
38	1	0	3.820928	2.156950	-0.346277
39	6	0	2.319124	3.715249	-0.406391
40	1	0	2.961311	4.425168	-0.920036
41	6	0	1.007272	4.069249	-0.079050
42	1	0	0.632170	5.054992	-0.341898
43	6	0	0.162085	3.172342	0.584096
44	1	0	-0.856675	3.450631	0.839799
45	7	0	-0.657865	-2.121343	0.559200
46	1	0	-0.409387	-2.946562	1.100309

Pyrrolo Indoline (*cis*,*cis*)-5b

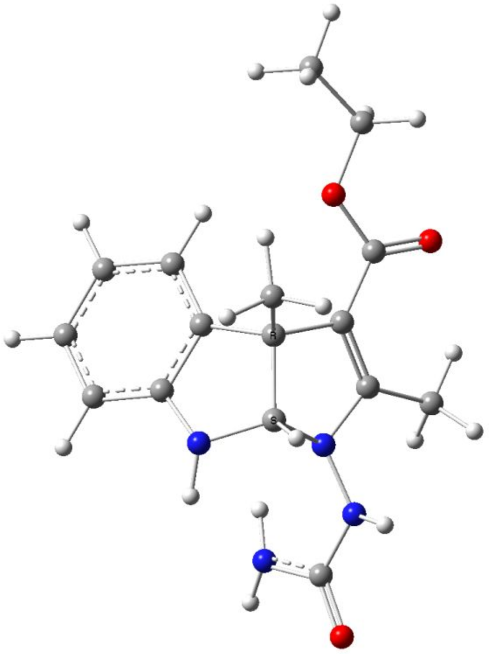

Energy = -1066.449166 au

Center Number	Atomic Number	Atomic Type	Coordinates (Angstroms) X	Y	Z
1	6	0	1.933327	-1.633535	-0.352265
2	8	0	2.118684	-2.660030	-0.997014
3	8	0	2.973006	-0.859521	0.059600
4	7	0	-1.621187	-0.843378	0.163474
5	7	0	-2.857671	-1.452106	0.344112
6	6	0	-4.032334	-0.879362	-0.139401
7	8	0	-5.123744	-1.305924	0.224860
8	6	0	-0.558059	-1.615457	-0.320925
9	6	0	4.295545	-1.333045	-0.287643
10	1	0	4.421702	-2.345423	0.108134
11	1	0	4.375759	-1.388838	-1.377727
12	6	0	-0.871767	-2.789270	-1.189183
13	1	0	-1.316274	-3.597063	-0.595475
14	1	0	-1.610314	-2.509122	-1.948182
15	1	0	0.035884	-3.159380	-1.660952
16	6	0	0.640547	-1.100546	0.076330
17	6	0	5.303325	-0.365902	0.304269
18	1	0	6.317616	-0.700966	0.062301
19	1	0	5.170180	0.642168	-0.101872
20	1	0	5.208849	-0.317265	1.393938
21	7	0	-3.862908	0.167570	-1.008205
22	1	0	-4.674846	0.396628	-1.565215
23	1	0	-2.961693	0.287292	-1.452636
24	7	0	-1.606498	1.440462	1.123785
25	1	0	-2.599479	1.554903	0.948624
26	6	0	0.414920	0.144401	0.945603

27	6	0	-1.126333	0.076713	1.222913
28	1	0	-1.381180	-0.354299	2.198731
29	6	0	1.209295	0.186371	2.264838
30	1	0	1.096077	-0.755686	2.813587
31	1	0	2.270944	0.351874	2.076895
32	1	0	0.836393	1.000650	2.896400
33	6	0	0.504266	1.473413	0.184959
34	6	0	-0.717128	2.157011	0.306211
35	6	0	1.551602	2.046265	-0.526219
36	1	0	2.495810	1.520475	-0.624473
37	6	0	1.368240	3.299624	-1.129425
38	1	0	2.179129	3.751004	-1.694160
39	6	0	0.144991	3.964740	-1.010431
40	1	0	0.008569	4.933105	-1.485081
41	6	0	-0.915114	3.403168	-0.288744
42	1	0	-1.864852	3.923099	-0.195783
43	1	0	-2.985932	-2.024733	1.175898

[3+2] Concerted cycloaddition structures

In·DD·$ZnCl_2$

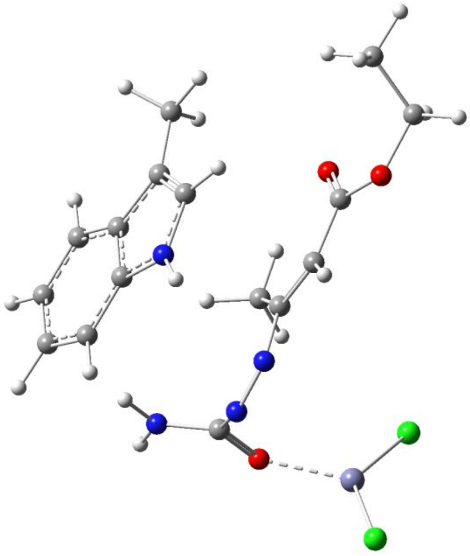

Energy= -3766.079050

Center Number	Atomic Number	Atomic Type	Coordinates (Angstroms) X	Y	Z
1	6	0	-1.635095	2.541094	-0.108214
2	8	0	-1.839879	2.645547	-1.305923
3	8	0	-2.038972	3.438416	0.809015
4	7	0	0.362028	-0.392955	1.012368
5	7	0	1.155690	-1.335623	0.805160
6	6	0	1.792236	-1.725230	-0.408399
7	8	0	2.893060	-1.221164	-0.741597
8	6	0	-0.181941	0.458144	-0.002423
9	6	0	-2.731496	4.622380	0.325000
10	1	0	-2.511390	5.381990	1.077391
11	1	0	-2.290404	4.917675	-0.629211
12	6	0	0.029113	0.263880	-1.475686
13	1	0	1.085493	0.354586	-1.746674
14	1	0	-0.325888	-0.721485	-1.793089
15	1	0	-0.530972	1.023706	-2.018494
16	6	0	-0.915828	1.437259	0.579762
17	1	0	-0.975192	1.441708	1.661962
18	6	0	-4.226321	4.375701	0.199787
19	1	0	-4.725152	5.308345	-0.085966
20	1	0	-4.437236	3.625176	-0.567271
21	1	0	-4.647136	4.039399	1.152757
22	7	0	1.290213	-2.797311	-1.011067
23	1	0	1.808964	-3.228647	-1.766298
24	1	0	0.400414	-3.201023	-0.728483

25	7	0	-3.063899	-1.403002	1.929921
26	1	0	-2.827762	-1.458098	2.909538
27	6	0	-3.953191	-0.628427	-0.001902
28	6	0	-3.828549	-0.409099	1.350127
29	1	0	-4.236767	0.387932	1.956921
30	6	0	-4.695439	0.220107	-0.991130
31	1	0	-4.013557	0.710181	-1.698120
32	1	0	-5.403725	-0.373671	-1.582727
33	1	0	-5.264729	1.006354	-0.484446
34	6	0	-3.219154	-1.837894	-0.277436
35	6	0	-2.675301	-2.297750	0.958689
36	6	0	-2.974998	-2.583523	-1.445855
37	1	0	-3.377982	-2.257713	-2.401338
38	6	0	-2.221569	-3.749621	-1.361027
39	1	0	-2.039605	-4.340010	-2.254919
40	6	0	-1.698641	-4.190334	-0.122887
41	1	0	-1.137717	-5.120805	-0.079622
42	6	0	-1.913939	-3.468260	1.050240
43	1	0	-1.515079	-3.809604	2.001267
44	17	0	4.173993	1.861055	-1.567951
45	17	0	4.539870	0.032989	2.101509
46	30	0	3.960284	0.284818	-0.028406

[TS$_{conc}$]‡

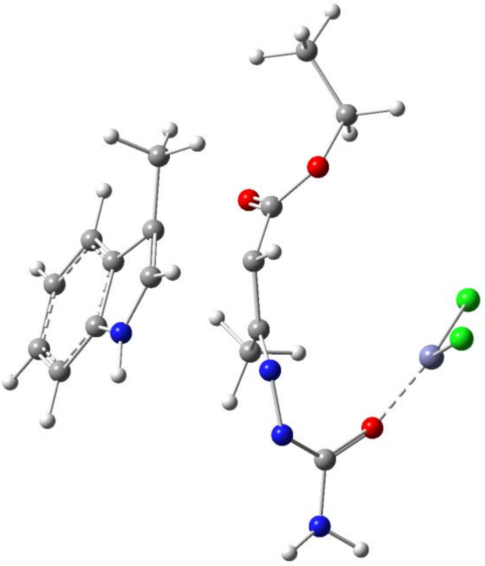

Energy = -3766.051482 au   Imaginary frequency = 239i cm^{-1}

Center Number	Atomic Number	Atomic Type	Coordinates (Angstroms) X	Y	Z
1	6	0	-0.704827	1.835234	-0.200341
2	8	0	-1.232002	1.944775	-1.290219
3	8	0	0.008903	2.800085	0.405455
4	7	0	0.216595	-1.457519	1.050719
5	7	0	0.786294	-2.619871	0.778802
6	6	0	1.698526	-2.778551	-0.196634
7	8	0	2.413890	-1.861235	-0.744785
8	6	0	-0.268757	-0.644366	0.112576
9	6	0	0.245032	4.031130	-0.340876
10	1	0	1.200481	4.389593	0.045363
11	1	0	0.352857	3.778895	-1.397404
12	6	0	-0.386574	-0.981159	-1.351228
13	1	0	0.518063	-0.699690	-1.902171
14	1	0	-0.514891	-2.062310	-1.470207
15	1	0	-1.234885	-0.465801	-1.800078
16	6	0	-0.712686	0.607959	0.671696
17	1	0	-0.327826	0.799689	1.665819
18	6	0	-0.865984	5.042181	-0.112044
19	1	0	-0.603962	5.983172	-0.608343
20	1	0	-1.813715	4.689686	-0.529183
21	1	0	-0.998404	5.243412	0.955693
22	7	0	1.937785	-4.065345	-0.581486
23	1	0	2.840372	-4.262169	-0.992956
24	1	0	1.525713	-4.799950	-0.022868
25	7	0	-2.828771	-1.593403	1.730139
26	1	0	-2.627175	-2.469120	2.198701
27	6	0	-2.785497	0.614077	1.241848
28	6	0	-2.532168	-0.392593	2.221297
29	1	0	-2.088585	-0.281079	3.200887
30	6	0	-3.066072	2.050978	1.607160
31	1	0	-3.034004	2.696717	0.726382

32	1	0	-4.069824	2.136340	2.038906
33	1	0	-2.349283	2.428722	2.342166
34	6	0	-3.446379	-0.103836	0.142772
35	6	0	-3.407924	-1.478145	0.455983
36	6	0	-4.036796	0.304814	-1.056238
37	1	0	-4.082853	1.354478	-1.325511
38	6	0	-4.556089	-0.671962	-1.906846
39	1	0	-5.021476	-0.372824	-2.841166
40	6	0	-4.488753	-2.037568	-1.579332
41	1	0	-4.899423	-2.772347	-2.265039
42	6	0	-3.911317	-2.467440	-0.384378
43	1	0	-3.866444	-3.519031	-0.119810
44	17	0	3.516025	1.229606	-1.818934
45	17	0	3.786742	0.094841	1.982055
46	30	0	3.199872	-0.227085	-0.147852

[3+2]$_{conc}$-Cycle·ZnCl$_2$

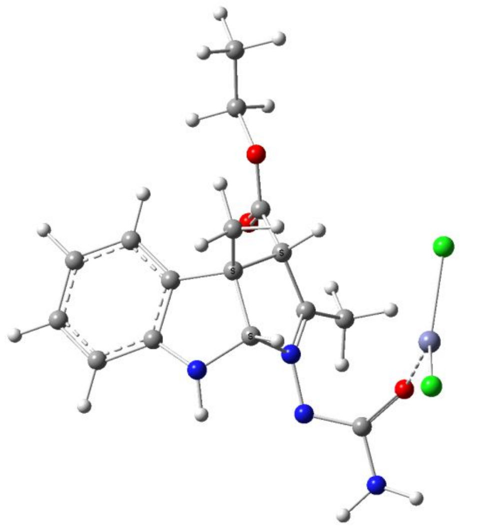

Energy = -3766.123756 au

Center Number	Atomic Number	Atomic Type	Coordinates (Angstroms) X	Y	Z
1	6	0	2.206978	1.417276	0.888116
2	8	0	2.674682	1.125998	1.969713
3	8	0	2.708864	2.353880	0.071472
4	7	0	-0.380635	-1.047492	0.640408
5	7	0	-1.232971	-1.998231	1.142175
6	6	0	-2.461609	-1.547572	1.421718
7	8	0	-2.892103	-0.348848	1.247767
8	6	0	0.095247	0.017983	1.211680
9	6	0	3.888142	3.069518	0.543190
10	1	0	3.633386	3.556152	1.488659
11	1	0	4.680379	2.340831	0.736819
12	6	0	-0.276577	0.441796	2.583469
13	1	0	-1.113418	1.148723	2.527615
14	1	0	-0.601519	-0.420181	3.171917
15	1	0	0.570058	0.931148	3.065353
16	6	0	0.979067	0.775707	0.252326
17	1	0	0.371522	1.591894	-0.165322
18	6	0	4.273122	4.066154	-0.531134
19	1	0	5.154484	4.628232	-0.205480
20	1	0	4.516462	3.558661	-1.469993
21	1	0	3.460976	4.775765	-0.716877
22	7	0	-3.297943	-2.450117	2.005827
23	1	0	-4.287123	-2.250011	1.962795
24	1	0	-3.028392	-3.424282	1.991713
25	7	0	0.732852	-2.590129	-0.897416
26	1	0	0.205917	-3.363913	-0.506550
27	6	0	1.292762	-0.251504	-0.902398
28	6	0	0.142536	-1.291161	-0.753148
29	1	0	-0.708326	-1.149315	-1.424555
30	6	0	1.321625	0.420516	-2.283116
31	1	0	2.147023	1.135657	-2.338845

32	1	0	1.461767	-0.328679	-3.068277
33	1	0	0.387616	0.959428	-2.477934
34	6	0	2.514401	-1.141943	-0.666561
35	6	0	2.108903	-2.485905	-0.640004
36	6	0	3.864950	-0.824355	-0.570773
37	1	0	4.200818	0.206316	-0.632823
38	6	0	4.800542	-1.852795	-0.395482
39	1	0	5.855710	-1.612451	-0.307832
40	6	0	4.377901	-3.183636	-0.334765
41	1	0	5.109238	-3.974800	-0.193600
42	6	0	3.026243	-3.520573	-0.464267
43	1	0	2.701949	-4.556847	-0.437839
44	17	0	-2.378570	2.720050	-0.233518
45	17	0	-4.556444	-0.267796	-1.861316
46	30	0	-3.347645	0.715196	-0.269615

[4+2] Concerted cycloaddition structures

cisoid-DD·$ZnCl_2$

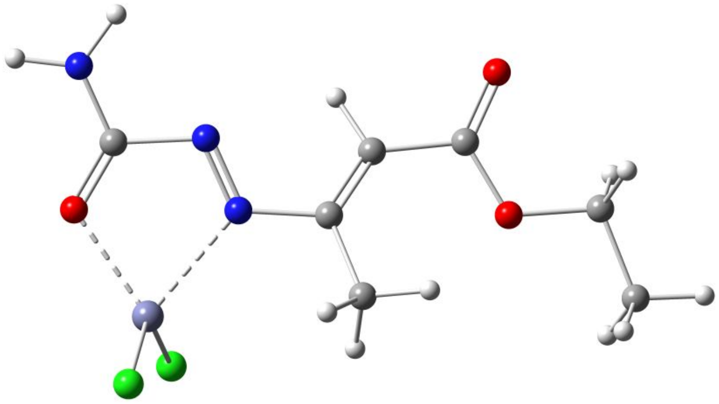

Energy = -3362.960776 au

Center Number	Atomic Number	Atomic Type	Coordinates (Angstroms) X	Y	Z
1	6	0	-1.877476	1.195578	0.087962
2	1	0	-1.538678	2.119035	0.545038
3	6	0	-0.954274	0.352094	-0.423743
4	6	0	-1.151036	-0.980063	-1.085762
5	1	0	-0.443582	-1.101943	-1.911560
6	1	0	-0.968534	-1.790289	-0.369077
7	1	0	-2.168557	-1.080266	-1.457428
8	6	0	-3.355983	1.059876	0.087669
9	8	0	-4.073671	2.037016	0.211424
10	8	0	-3.801068	-0.196461	-0.037390
11	7	0	0.421283	0.693578	-0.245754
12	7	0	0.714656	1.907895	-0.266361
13	6	0	2.128936	2.114437	0.020653
14	7	0	2.445281	3.398339	-0.036403
15	1	0	1.739873	4.087873	-0.264242
16	1	0	3.395297	3.700723	0.139920
17	8	0	2.909909	1.187357	0.284485
18	6	0	-5.247016	-0.373196	-0.022230
19	1	0	-5.665908	0.156954	-0.882403
20	1	0	-5.637987	0.090171	0.887712
21	6	0	-5.524392	-1.861832	-0.072990
22	1	0	-5.084269	-2.372098	0.789388
23	1	0	-6.606355	-2.028984	-0.056810
24	1	0	-5.121518	-2.307974	-0.987701
25	30	0	2.093711	-0.681004	0.161955
26	17	0	2.839286	-1.615790	-1.726497
27	17	0	1.621092	-1.578706	2.157711

endo-**In·DD·ZnCl$_2$**

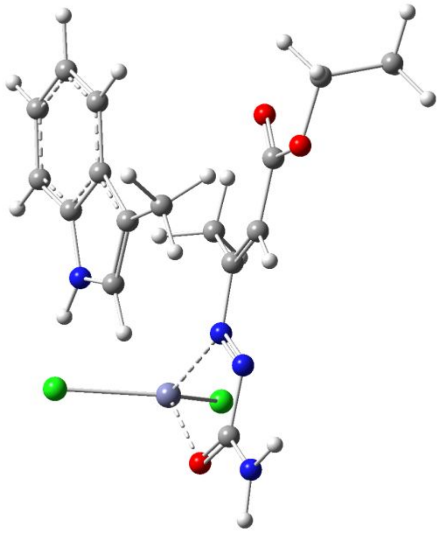

Energy = -3766.110658 au

Center Number	Atomic Number	Atomic Type	Coordinates (Angstroms) X	Y	Z
1	6	0	2.537734	-1.378933	-0.794475
2	8	0	2.823548	-0.934810	-1.892725
3	8	0	3.425795	-1.976973	0.028920
4	7	0	-1.148040	-0.979019	0.049634
5	1	0	0.074177	0.558810	2.964561
6	6	0	0.673735	1.017334	2.190225
7	6	0	2.318305	1.784712	0.842831
8	6	0	1.074380	2.305794	0.393235
9	6	0	0.974898	3.159950	-0.708493
10	1	0	0.015457	3.551177	-1.034103
11	6	0	3.400983	2.992439	-0.938725
12	1	0	4.302186	3.276074	-1.475112
13	6	0	2.156725	3.494595	-1.367627
14	1	0	2.115884	4.156945	-2.227743
15	6	0	3.492875	2.142079	0.160570
16	1	0	4.458665	1.763591	0.485242
17	6	0	2.041090	0.949619	1.991012
18	6	0	4.789640	-2.112344	-0.457657
19	1	0	5.047317	-1.216653	-1.026223
20	1	0	5.392697	-2.151801	0.451295
21	7	0	-1.130815	-1.646536	1.123654
22	6	0	3.038754	0.218611	2.835119
23	1	0	2.550119	-0.294348	3.669602
24	1	0	3.583778	-0.532844	2.250689
25	1	0	3.785703	0.904734	3.255141
26	6	0	4.950784	-3.373457	-1.291679
27	1	0	6.000724	-3.485218	-1.583816
28	1	0	4.656525	-4.259231	-0.719796
29	1	0	4.345447	-3.321257	-2.201191
30	7	0	-2.389511	-2.342721	2.906055
31	1	0	-3.231821	-2.448156	3.456916

32	1	0	-1.534029	-2.784196	3.217217
33	6	0	-2.416475	-1.663697	1.765122
34	8	0	-3.431240	-1.114990	1.295260
35	7	0	0.092058	1.810755	1.232794
36	1	0	-0.899936	2.020310	1.152741
37	6	0	0.055773	-0.899413	-0.694000
38	6	0	1.206137	-1.372259	-0.144305
39	1	0	1.168240	-1.823182	0.838032
40	6	0	-0.115538	-0.263873	-2.041164
41	1	0	-0.421752	0.783903	-1.932672
42	1	0	0.817110	-0.296106	-2.599453
43	1	0	-0.901457	-0.779723	-2.604321
44	30	0	-3.056270	-0.069281	-0.409756
45	17	0	-4.012456	-0.891291	-2.256055
46	17	0	-3.118510	2.112792	0.190123

[TS]‡$_{endo}$

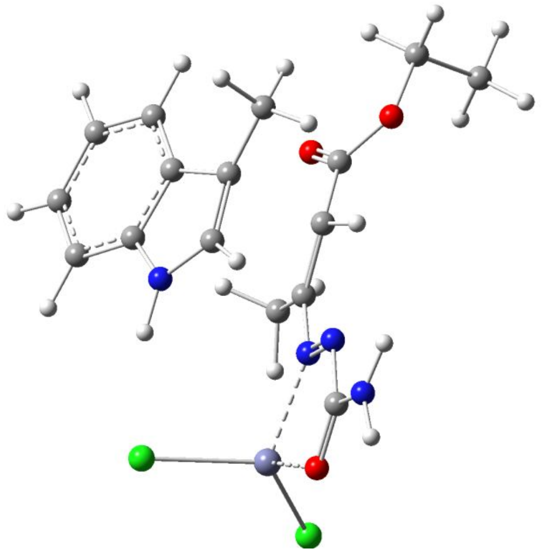

Energy = -3766.101776 au Imaginary frequency = 259i cm^{-1}

Center Number	Atomic Number	Atomic Type	Coordinates (Angstroms) X	Y	Z
1	6	0	2.691289	-1.117110	-0.545893
2	8	0	3.037476	-0.477531	-1.521963
3	8	0	3.450383	-2.058118	0.053942
4	7	0	-0.986740	-0.912093	0.043231
5	1	0	0.038127	0.186426	2.845995
6	6	0	0.546297	0.754610	2.083505
7	6	0	2.091665	1.747750	0.705092
8	6	0	0.838507	2.372401	0.545781
9	6	0	0.632322	3.458402	-0.297817
10	1	0	-0.346371	3.911977	-0.412514
11	6	0	3.015344	3.352270	-0.820482
12	1	0	3.865671	3.760886	-1.357885
13	6	0	1.751438	3.941050	-0.982327
14	1	0	1.639546	4.792906	-1.646066
15	6	0	3.198005	2.248211	0.015727
16	1	0	4.178244	1.795782	0.124610
17	6	0	1.895872	0.610970	1.615506
18	6	0	4.743365	-2.351601	-0.546339
19	1	0	5.173778	-1.421133	-0.922120
20	1	0	5.344367	-2.719839	0.287256
21	7	0	-0.966461	-1.476517	1.241064
22	6	0	2.977970	-0.010822	2.456907
23	1	0	2.621737	-0.913144	2.962242
24	1	0	3.855432	-0.275666	1.863576
25	1	0	3.299885	0.702560	3.225275
26	6	0	4.610624	-3.393826	-1.644905
27	1	0	5.604934	-3.649125	-2.027371
28	1	0	4.144327	-4.306836	-1.261404
29	1	0	4.010960	-3.011817	-2.476146
30	7	0	-2.260635	-2.298877	2.935913

31	1	0	-3.145478	-2.565007	3.343255
32	1	0	-1.405525	-2.658408	3.335123
33	6	0	-2.239928	-1.695849	1.734901
34	8	0	-3.316977	-1.372253	1.156256
35	7	0	-0.067158	1.718404	1.398159
36	1	0	-1.071230	1.915405	1.413173
37	6	0	0.194078	-0.671630	-0.546663
38	6	0	1.388352	-0.995016	0.168702
39	1	0	1.259001	-1.705212	0.974821
40	6	0	0.165397	-0.069983	-1.926164
41	1	0	0.551856	0.953931	-1.932869
42	1	0	0.789159	-0.646139	-2.614733
43	1	0	-0.856951	-0.062015	-2.311544
44	30	0	-2.924853	-0.282014	-0.474420
45	17	0	-3.822846	-0.904846	-2.435497
46	17	0	-3.130658	1.925005	0.193867

----

endo-**cycle·ZnCl$_2$**

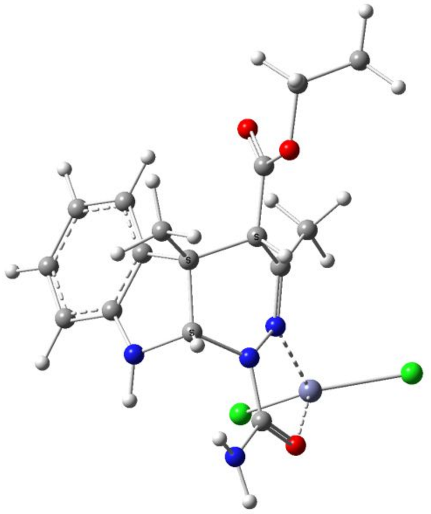

Energy = -3766.146345 au

Center Number	Atomic Number	Atomic Type	Coordinates (Angstroms) X	Y	Z
1	6	0	2.984128	-0.982157	-0.184572
2	8	0	3.306596	-0.741535	-1.331207
3	8	0	3.749548	-1.634109	0.700620
4	7	0	-0.711495	-0.551121	0.224859
5	1	0	0.617129	1.041047	2.819856
6	6	0	0.363934	1.147611	1.763625
7	6	0	1.373216	1.957066	-0.251498
8	6	0	0.346556	2.821740	0.159299
9	6	0	-0.080024	3.882425	-0.636174
10	1	0	-0.881489	4.539634	-0.311746
11	6	0	1.584879	3.224446	-2.287264
12	1	0	2.063439	3.385778	-3.248557
13	6	0	0.553621	4.069284	-1.869990
14	1	0	0.231648	4.885054	-2.511474
15	6	0	2.005658	2.162781	-1.473119
16	1	0	2.799094	1.501682	-1.802651
17	6	0	1.636064	0.953075	0.874262
18	6	0	5.068944	-2.070400	0.254848
19	1	0	5.472131	-1.315678	-0.423073
20	1	0	5.660754	-2.090601	1.171517
21	7	0	-0.656113	0.073118	1.480282
22	6	0	2.891240	1.350972	1.675764
23	1	0	3.074588	0.655915	2.502379
24	1	0	3.772695	1.361538	1.028072
25	1	0	2.765636	2.357781	2.085124
26	6	0	4.997441	-3.439345	-0.399988
27	1	0	6.009663	-3.773012	-0.653131
28	1	0	4.553119	-4.174114	0.278713
29	1	0	4.408169	-3.403883	-1.320842
30	7	0	-1.955318	0.705615	3.327432

31	1	0	-2.821718	0.570793	3.832844
32	1	0	-1.143838	0.923101	3.888581
33	6	0	-1.866977	0.052759	2.148922
34	8	0	-2.866085	-0.532410	1.670084
35	7	0	-0.086823	2.481312	1.450614
36	1	0	-1.012163	2.758564	1.756884
37	6	0	0.419550	-0.881750	-0.302477
38	6	0	1.673687	-0.561796	0.475689
39	1	0	1.603209	-1.119946	1.419070
40	6	0	0.435495	-1.539217	-1.642993
41	1	0	0.891590	-0.869395	-2.379224
42	1	0	1.051663	-2.444000	-1.612415
43	1	0	-0.570888	-1.808769	-1.963623
44	30	0	-2.714332	-0.971603	-0.296543
45	17	0	-2.989437	-3.155594	-0.762029
46	17	0	-3.662986	0.660512	-1.542930

(*cis*,*cis*)-**3ab**

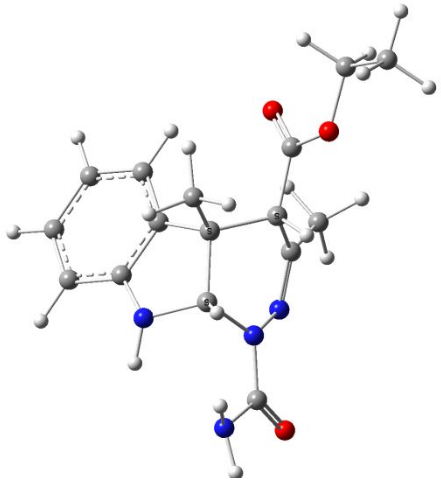

Energy = -1066.438593 au

Center Number	Atomic Number	Atomic Type	Coordinates (Angstroms) X	Y	Z
1	6	0	-2.349044	0.086345	0.331807
2	8	0	-2.583259	1.119531	0.930047
3	8	0	-3.292443	-0.637765	-0.291821
4	7	0	0.939600	-1.368926	1.394465
5	1	0	1.038228	-1.360120	-1.919244
6	6	0	1.204440	-0.731826	-1.040473
7	6	0	0.645586	1.470748	-0.289143
8	6	0	2.013322	1.397823	-0.604241
9	6	0	2.887233	2.442820	-0.307470
10	1	0	3.944379	2.372902	-0.548324
11	6	0	1.003576	3.672349	0.623030
12	1	0	0.614348	4.563922	1.106235
13	6	0	2.362747	3.582010	0.313959
14	1	0	3.028671	4.405086	0.560162
15	6	0	0.134925	2.614000	0.315432
16	1	0	-0.917538	2.678352	0.566771
17	6	0	-0.031646	0.198582	-0.808708
18	6	0	-4.651203	-0.121427	-0.235563
19	1	0	-4.944566	-0.038026	0.814828
20	1	0	-4.657639	0.882296	-0.670343
21	7	0	1.433413	-1.649603	0.117018
22	6	0	-0.726781	0.477505	-2.156848
23	1	0	-1.185271	-0.431836	-2.562245
24	1	0	-1.507780	1.234876	-2.036051
25	1	0	-0.002257	0.856292	-2.884224
26	6	0	-5.535383	-1.084376	-1.002561
27	1	0	-6.569874	-0.726307	-0.979862
28	1	0	-5.220011	-1.159274	-2.048077
29	1	0	-5.507150	-2.083481	-0.556338
30	7	0	3.168782	-2.674463	-1.097946

31	1	0	3.806111	-3.461872	-1.089720
32	1	0	2.628468	-2.617033	-1.952733
33	6	0	2.527355	-2.526104	0.126516
34	8	0	2.924276	-3.098293	1.131972
35	7	0	2.298425	0.201275	-1.275348
36	1	0	3.233694	-0.185803	-1.209467
37	6	0	-0.228178	-0.838655	1.451102
38	6	0	-0.983041	-0.564950	0.165501
39	1	0	-1.161089	-1.540848	-0.306608
40	6	0	-0.787892	-0.522843	2.806872
41	1	0	-0.949559	0.553995	2.922608
42	1	0	-1.760984	-1.009075	2.951707
43	1	0	-0.096405	-0.871813	3.576742

----

exo-**In·DD·$ZnCl_2$**

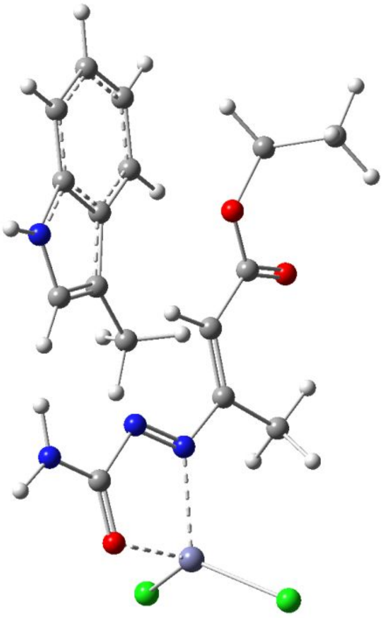

Energy = -3766.105869 au

Center Number	Atomic Number	Atomic Type	Coordinates (Angstroms) X	Y	Z
1	6	0	-1.677936	1.700356	0.061072
2	8	0	-1.819695	2.260375	-1.012974
3	8	0	-2.602361	1.682158	1.037197
4	7	0	1.781461	0.166553	0.475893
5	7	0	1.502045	-0.578186	1.456590
6	6	0	2.650829	-1.311957	1.919832
7	8	0	3.776873	-1.241378	1.393311
8	6	0	0.738337	0.954004	-0.075108
9	6	0	-3.851360	2.385307	0.786168
10	1	0	-4.112252	2.263515	-0.266199
11	1	0	-4.579984	1.853570	1.400436
12	6	0	1.154356	1.753332	-1.275274
13	1	0	1.487795	1.088673	-2.080944
14	1	0	0.327254	2.359033	-1.638061
15	1	0	1.998743	2.403964	-1.017908
16	6	0	-0.488510	0.932292	0.509702
17	1	0	-0.624325	0.361180	1.418632
18	6	0	-3.741145	3.849804	1.178561
19	1	0	-4.714728	4.335347	1.049423
20	1	0	-3.442049	3.952582	2.226577
21	1	0	-3.012749	4.370294	0.549830
22	7	0	2.359103	-2.068006	2.971073
23	1	0	3.076377	-2.633363	3.406840
24	1	0	1.424433	-2.067019	3.357812
25	30	0	3.834510	-0.023033	-0.224385
26	17	0	5.054634	1.830133	0.119215

27	17	0	3.795574	-1.258685	-2.108450
28	6	0	-3.563822	-1.191887	-0.854355
29	6	0	-3.883889	-1.624083	0.463691
30	7	0	-2.720572	-2.111288	1.026520
31	1	0	-2.636681	-2.505101	1.952033
32	6	0	-5.177041	-1.531643	0.986684
33	1	0	-5.404534	-1.868956	1.993982
34	6	0	-6.164052	-0.994495	0.163696
35	1	0	-7.180665	-0.911875	0.538138
36	6	0	-5.871486	-0.559957	-1.146794
37	1	0	-6.668277	-0.148785	-1.760339
38	6	0	-4.583211	-0.652686	-1.661276
39	1	0	-4.366476	-0.317461	-2.672073
40	6	0	-2.158784	-1.435984	-1.059763
41	6	0	-1.694113	-1.999114	0.115205
42	1	0	-0.702103	-2.353536	0.359661
43	6	0	-1.377021	-1.170322	-2.309812
44	1	0	-1.777913	-1.735744	-3.160927
45	1	0	-1.414069	-0.108324	-2.582800
46	1	0	-0.326338	-1.452179	-2.189757

[TS]$^{‡}_{exo}$

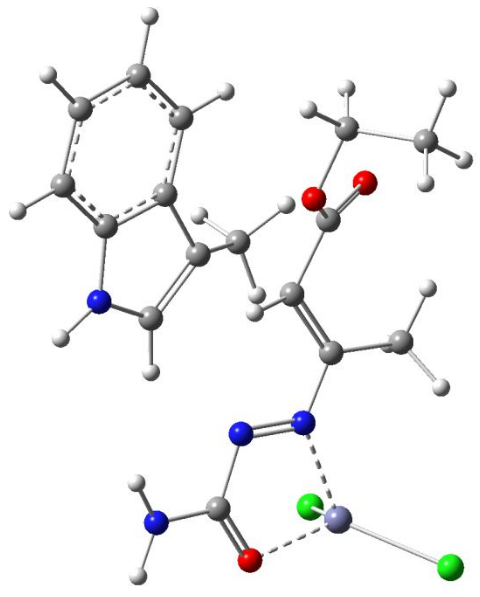

Energy = -3766.096573 au Imaginary frequency = 272i cm^{-1}

Center Number	Atomic Number	Atomic Type	Coordinates (Angstroms) X	Y	Z
1	6	0	1.934763	1.704069	0.069010
2	8	0	2.056728	2.389669	1.069913
3	8	0	2.750852	1.761945	-0.995427
4	7	0	-1.419797	0.105838	-0.377472
5	7	0	-1.017564	-0.890987	-1.141523
6	6	0	-2.083982	-1.663623	-1.585517
7	8	0	-3.293272	-1.493720	-1.264770
8	6	0	-0.484853	0.925158	0.133422
9	6	0	3.822921	2.747785	-0.966291
10	1	0	4.202140	2.821096	0.054481
11	1	0	4.592521	2.317716	-1.609532
12	6	0	-0.983605	2.032656	1.028505
13	1	0	-1.280309	1.645297	2.010335
14	1	0	-0.217622	2.789992	1.186730
15	1	0	-1.865443	2.502944	0.581669
16	6	0	0.879411	0.673956	-0.172222
17	1	0	1.052160	0.072587	-1.055396
18	6	0	3.339535	4.091447	-1.486633
19	1	0	4.181469	4.791353	-1.523955
20	1	0	2.927866	3.994044	-2.496100
21	1	0	2.572433	4.512851	-0.830369
22	7	0	-1.718101	-2.665034	-2.403791
23	1	0	-2.431248	-3.227992	-2.845148
24	1	0	-0.775333	-2.697861	-2.764818
25	30	0	-3.481137	0.041197	0.011373
26	17	0	-4.688668	1.777520	-0.774460
27	17	0	-3.821708	-0.699625	2.138580
28	6	0	3.036485	-1.024610	0.809422

29	6	0	3.112263	-2.070120	-0.135951
30	7	0	1.830088	-2.626350	-0.240930
31	1	0	1.582482	-3.406134	-0.837346
32	6	0	4.295985	-2.440745	-0.766202
33	1	0	4.327027	-3.246736	-1.492587
34	6	0	5.446719	-1.736708	-0.406993
35	1	0	6.393879	-1.997954	-0.869014
36	6	0	5.403207	-0.706642	0.547729
37	1	0	6.320209	-0.190474	0.815297
38	6	0	4.204632	-0.339871	1.161540
39	1	0	4.181064	0.459423	1.895619
40	6	0	1.633789	-0.900587	1.224913
41	6	0	0.973700	-1.988470	0.565838
42	1	0	-0.038829	-2.334033	0.699310
43	6	0	1.188061	-0.366435	2.561114
44	1	0	1.600287	-0.989905	3.363617
45	1	0	1.536150	0.657939	2.714115
46	1	0	0.098591	-0.385247	2.650026

----

exo-**cycle·ZnCl$_2$**

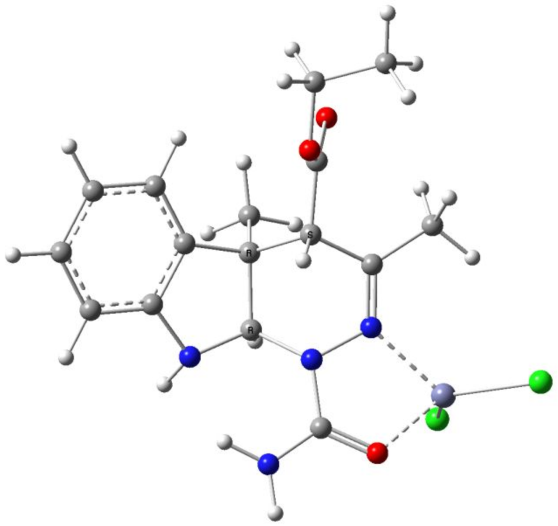

Energy = -3766.146972 au

Center Number	Atomic Number	Atomic Type	Coordinates (Angstroms) X	Y	Z
1	6	0	2.016686	1.977941	0.314551
2	8	0	2.134530	2.545009	1.383557
3	8	0	2.679875	2.305093	-0.802255
4	7	0	-1.198386	0.133914	0.007163
5	7	0	-0.611610	-1.065464	-0.439344
6	6	0	-1.539168	-1.872744	-1.113211
7	8	0	-2.714160	-1.475518	-1.303752
8	6	0	-0.365187	1.082723	0.276333
9	6	0	3.601761	3.433573	-0.730081
10	1	0	4.083794	3.426393	0.249467
11	1	0	4.341825	3.216463	-1.502038
12	6	0	-0.875776	2.399541	0.759665
13	1	0	-0.510823	2.600945	1.772011
14	1	0	-0.495460	3.203450	0.118728
15	1	0	-1.965541	2.426851	0.744910
16	6	0	1.098858	0.788273	0.056489
17	1	0	1.191047	0.515435	-1.001801
18	6	0	2.879308	4.743863	-0.994143
19	1	0	3.606521	5.563034	-1.006526
20	1	0	2.370813	4.720920	-1.963034
21	1	0	2.143701	4.951158	-0.211402
22	7	0	-1.110753	-3.040978	-1.594047
23	1	0	-1.764967	-3.605657	-2.117447
24	1	0	-0.128753	-3.289453	-1.526468
25	30	0	-3.289870	0.046078	-0.101298
26	17	0	-4.197107	1.801888	-1.175275
27	17	0	-4.206705	-0.830932	1.771509
28	6	0	2.782758	-1.113102	0.382420
29	6	0	2.546583	-2.392013	-0.122683
30	7	0	1.166449	-2.731432	-0.073940
31	1	0	0.978614	-3.615537	0.391299

32	6	0	3.577489	-3.195254	-0.602577
33	1	0	3.377434	-4.189035	-0.992050
34	6	0	4.877832	-2.682298	-0.562697
35	1	0	5.700389	-3.289786	-0.929181
36	6	0	5.130890	-1.402031	-0.059978
37	1	0	6.147359	-1.021220	-0.034363
38	6	0	4.081085	-0.610077	0.419607
39	1	0	4.289940	0.371864	0.834256
40	6	0	1.480204	-0.500079	0.873410
41	6	0	0.426449	-1.619610	0.510342
42	1	0	-0.115407	-1.944668	1.403056
43	6	0	1.495363	-0.264871	2.395475
44	1	0	1.684075	-1.212272	2.909960
45	1	0	2.277391	0.441966	2.676980
46	1	0	0.536279	0.128604	2.749833

(*cis*,*trans*)-**3ab**

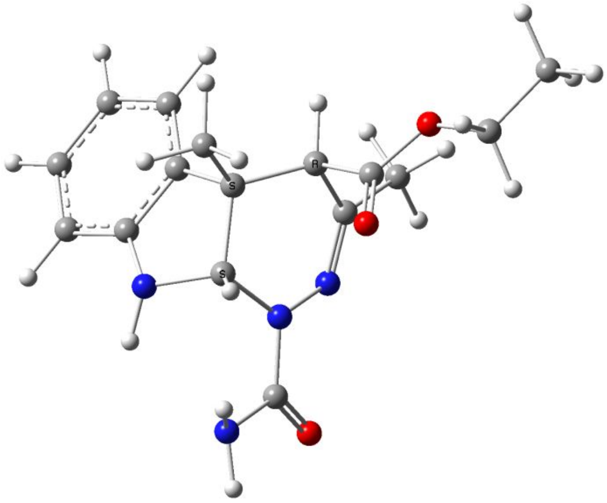

Energy = -1066.443165 au

Center Number	Atomic Number	Atomic Type	Coordinates (Angstroms) X	Y	Z
1	6	0	2.223891	-0.128774	-0.510816
2	8	0	2.262599	0.819806	-1.271069
3	8	0	3.310999	-0.761489	-0.042857
4	7	0	0.025379	1.115485	1.439095
5	1	0	-0.332165	1.342414	-1.809849
6	6	0	-0.791754	0.844031	-0.955207
7	6	0	-1.512054	-1.319045	-0.261483
8	6	0	-2.626260	-0.496947	-0.492706
9	6	0	-3.909307	-0.900278	-0.126145
10	1	0	-4.769608	-0.259784	-0.299184
11	6	0	-2.954425	-2.983442	0.708076
12	1	0	-3.090409	-3.951822	1.180461
13	6	0	-4.055818	-2.154629	0.480406
14	1	0	-5.047145	-2.483047	0.781647
15	6	0	-1.669204	-2.565265	0.328075
16	1	0	-0.813802	-3.213844	0.502768
17	6	0	-0.277438	-0.624815	-0.837980
18	6	0	4.600757	-0.239658	-0.470660
19	1	0	4.646189	-0.289826	-1.562329
20	1	0	4.663041	0.811517	-0.175069
21	7	0	-0.483131	1.647869	0.268829
22	6	0	0.010150	-1.196953	-2.243466
23	1	0	0.829204	-0.663011	-2.732609
24	1	0	0.276638	-2.256560	-2.166327
25	1	0	-0.881289	-1.116871	-2.872640
26	6	0	5.673920	-1.084588	0.185949
27	1	0	6.660889	-0.720640	-0.118115
28	1	0	5.606956	-1.026169	1.276969

29	1	0	5.586436	-2.133553	-0.114366
30	7	0	-1.500733	3.500736	-0.749855
31	1	0	-1.677533	4.492438	-0.640762
32	1	0	-1.052170	3.281719	-1.632093
33	6	0	-0.975623	2.951290	0.416199
34	8	0	-0.994170	3.556461	1.478552
35	7	0	-2.229973	0.672141	-1.156848
36	1	0	-2.790670	1.510009	-1.042702
37	6	0	0.684446	0.017718	1.390062
38	6	0	0.947687	-0.726156	0.088475
39	1	0	1.151054	-1.776926	0.313919
40	6	0	1.231433	-0.545896	2.669477
41	1	0	2.307761	-0.735138	2.577876
42	1	0	1.053881	0.145062	3.496567
43	1	0	0.754109	-1.507364	2.900749

IRC plots

[32CA] stepwise mechanism 1st step

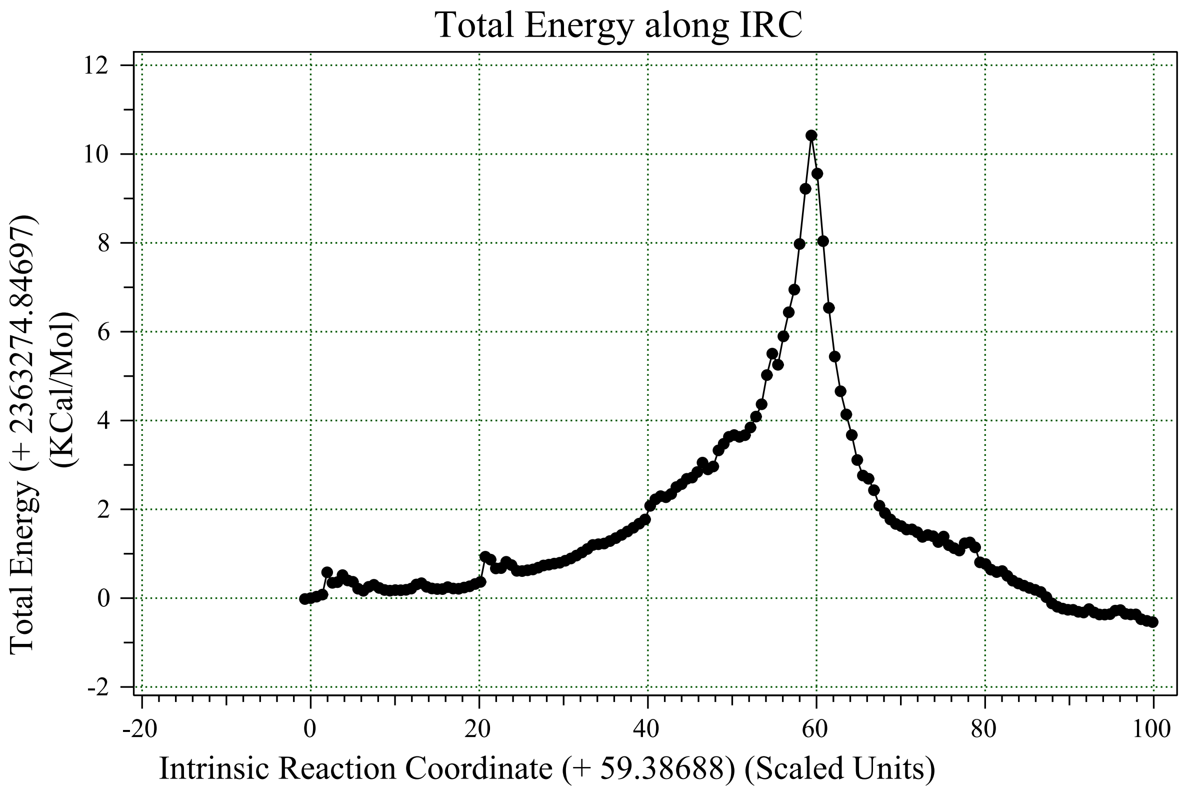

[32CA] stepwise mechanism 2nd step

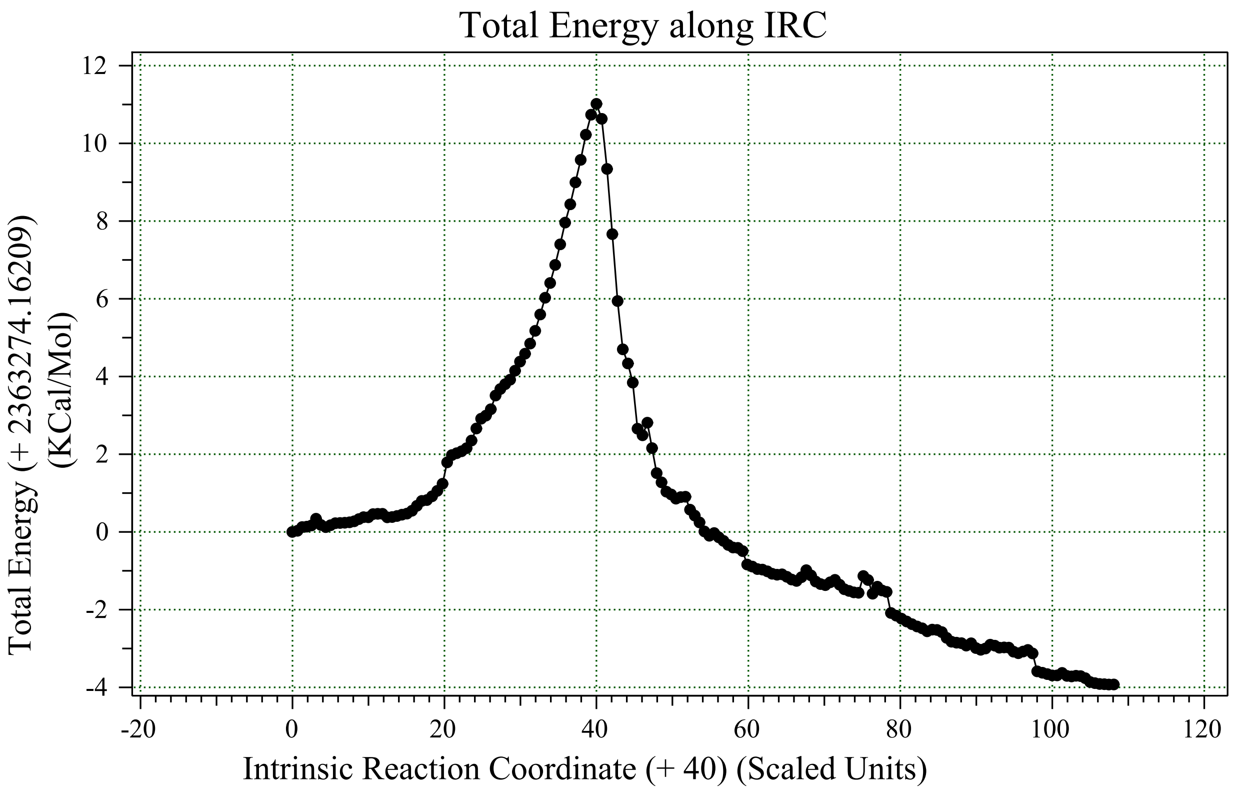

[32CA] concerted mechanism

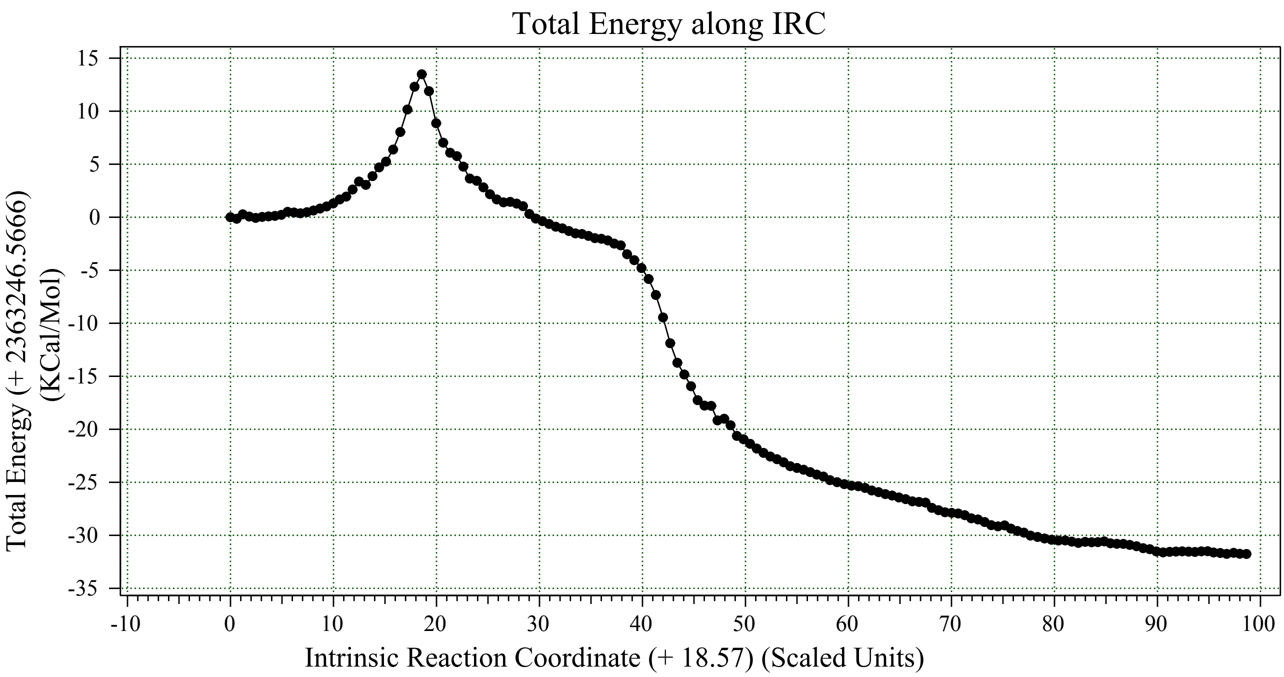

[42CA] concerted mechanism ***endo*** **pathway**

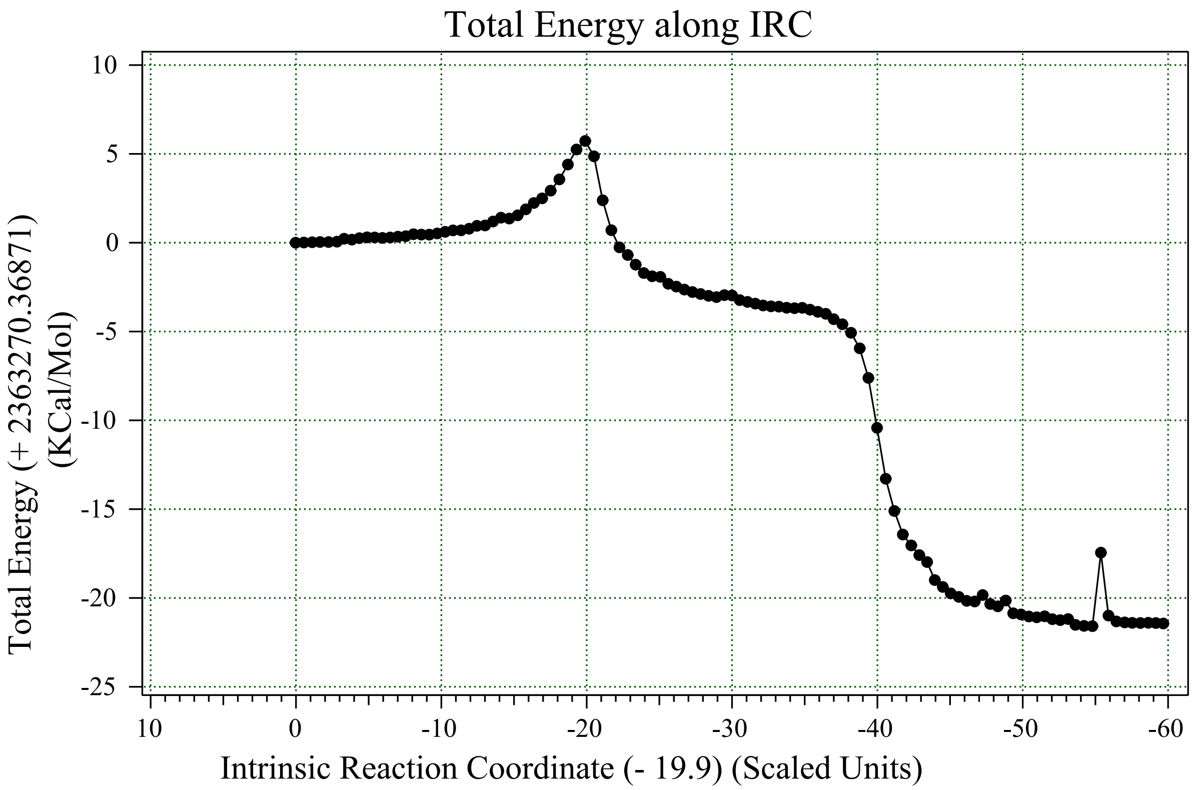

[42CA] concerted mechanism ***exo*** **pathway**

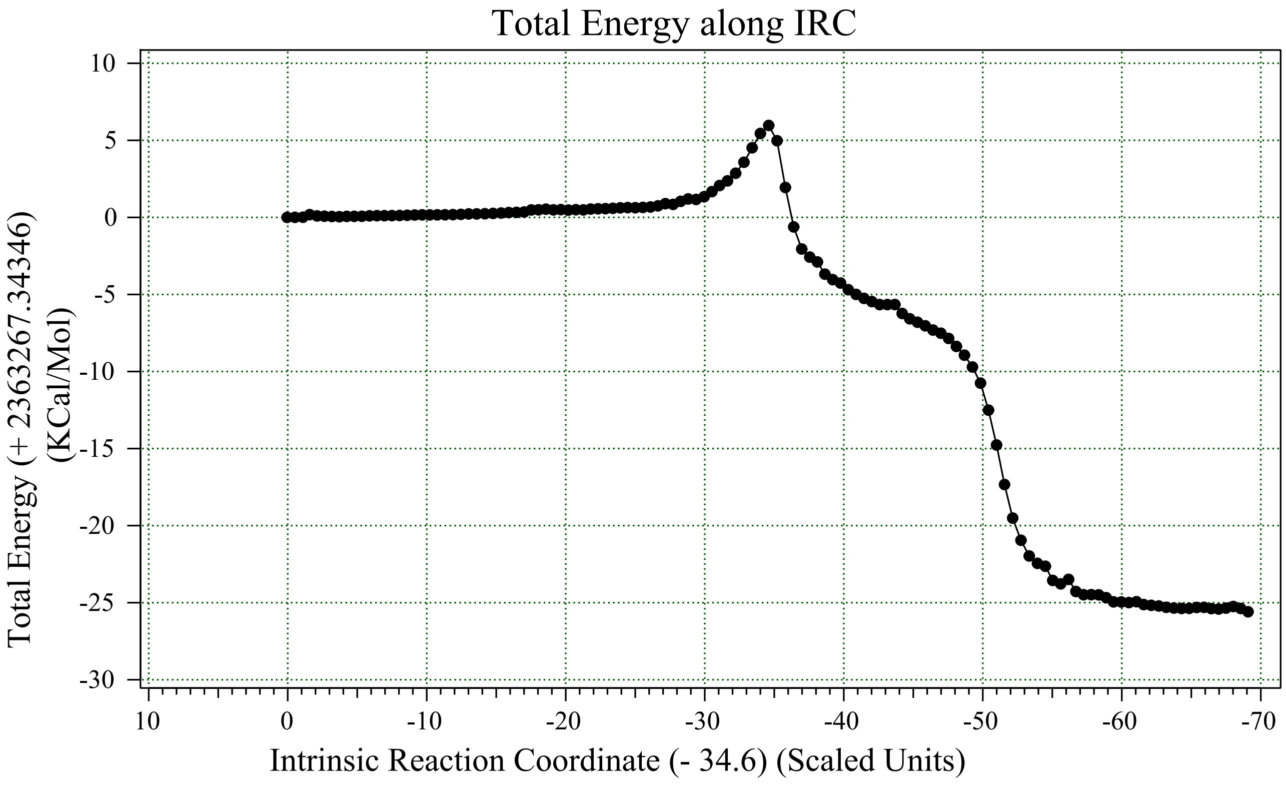

Supplement: Supplementary file 1 — jo0c01489_si_001.pdf [file jo0c01489_si_001.pdf]
