# Supplementary material for: Synthesis of Polycyclic Fused Indoline Scaffolds through a Substrate-Guided Reactivity Switch
Source: J Org Chem. 2020 Aug 12;85(17):11409–25. doi: 10.1021/acs.joc.0c01489 (PMC8010796; doi:10.1021/acs.joc.0c01489)

Synthesis of Polycylic Fused Indoline Scaffolds through a Substrate-Guided Reactivity Switch

Cecilia Ciccolini,† Giacomo Mari,† Francesco G. Gatti,‡ Giuseppe Gatti,† Gianluca Giorgi,§ Fabio Mantellini,† and Gianfranco Favi*,†

†Department of Biomolecular Sciences, Section of Chemistry and Pharmaceutical Technologies, University of Urbino "Carlo Bo", Via I Maggetti 24, 61029 Urbino, Italy

‡Department of Chemistry, Materials and Chemical Engineering "G. Natta", Piazza Leonardo da Vinci 32, 20133 Milano, Italy

§Department of Biotechnologies, Chemistry & Pharmacy, University of Siena, Via A. Moro 2, 53100 Siena, Italy

e-mail: gianfranco.favi@uniurb.it

SUPPORTING INFORMATION

Table of Contents

1. *Starting materials (Figure S1)* S2
2. *Optimization for reaction of* **1a** *with cyclic azoalkene* **2a** *(Table S1)* S3
3. *X-ray structure of compound* **3e** *(CCDC: 1950877)* S4
4. ^{1}H and ^{13}C NMR spectra of all products S5–S69

1. Starting materials (Figure S1):

Indoles

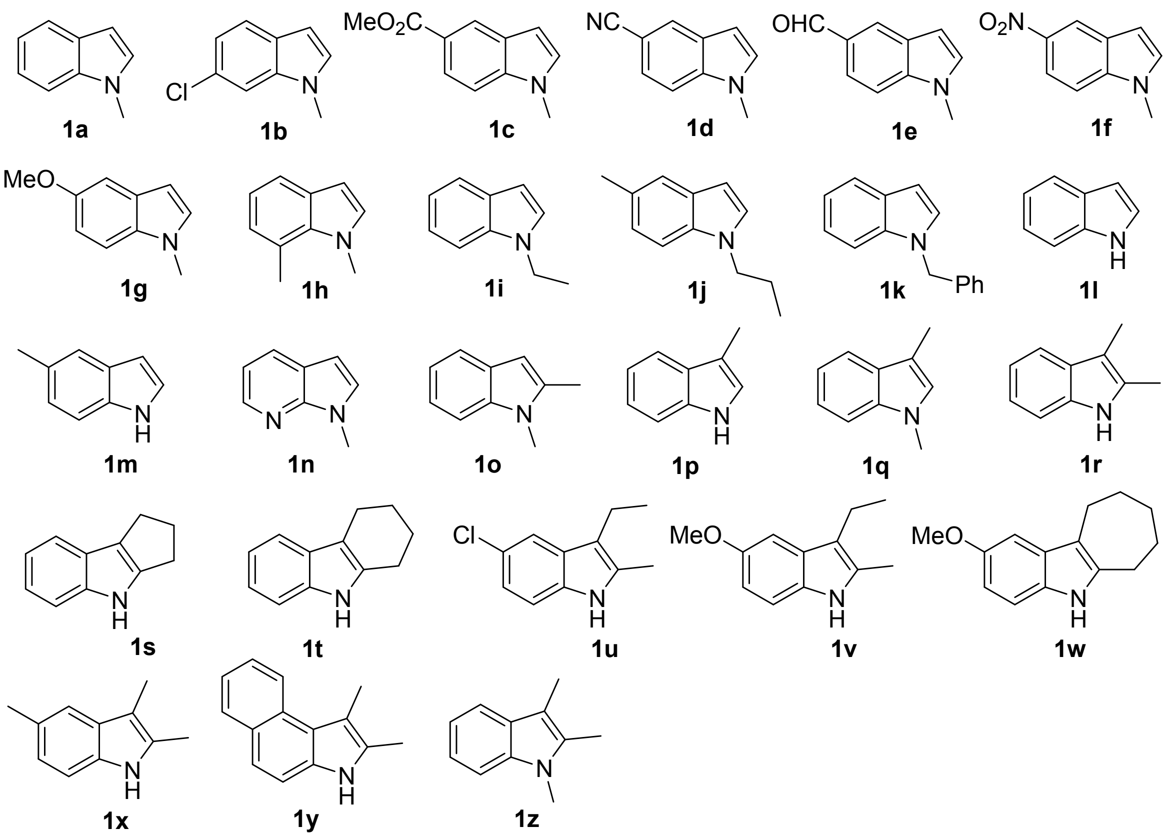

Azoalkenes

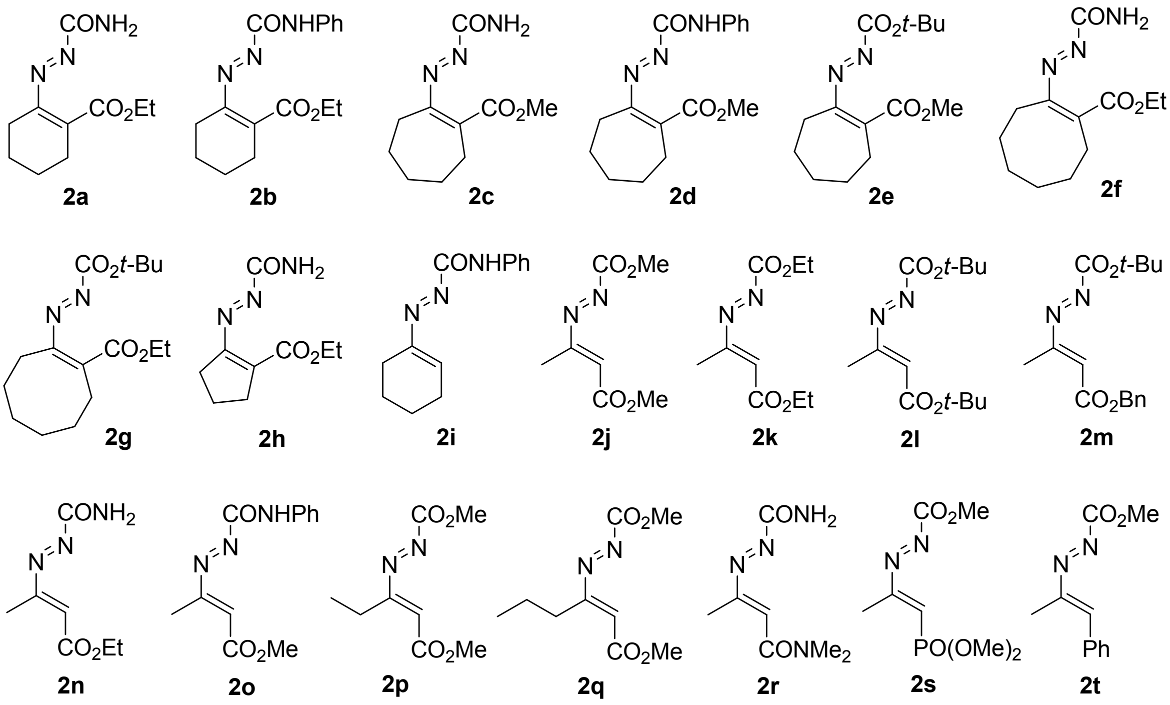

2. Supplementary Table (Table S1). Optimization Conditions for Reaction of *N*-Methylindole (**1a**) with Cyclic Azoalkene **2a**.[a]

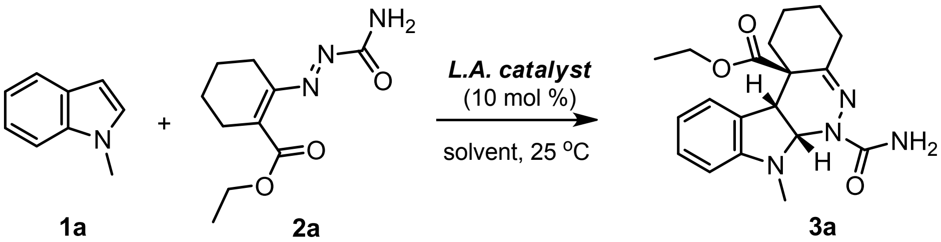

1a **2a** **3a**

entry	catalyst	solvent	yield (%)[b]
1	–	CH_2Cl_2	0
2	$Sc(OTf)_3$	CH_2Cl_2	0
3	$Zn(OAc)_2$	CH_2Cl_2	0
4	$ZnSO_4$	CH_2Cl_2	0
5	$Zn(OTf)_2$	CH_2Cl_2	0
6	$SmCl_3$ $6H_2O$	CH_2Cl_2	< 5
7	$LiClO_4$	CH_2Cl_2	0
8	LiCl	CH_2Cl_2	0
9	$CuCl_2$	CH_2Cl_2	0
10	$Cu(OTf)_2$	CH_2Cl_2	< 5
11	$CuBr_2$	CH_2Cl_2	0
12	$InBr_3$	CH_2Cl_2	0
13	$ZnBr_2$	CH_2Cl_2	37
14	$ZnCl_2$	CH_2Cl_2	39
15[c]	$ZnCl_2$	CH_2Cl_2	50
16[c]	$ZnCl_2$	Acetone	43
17[c]	$ZnCl_2$	THF	40
18[c]	$ZnCl_2$	CH_3CN	23
19[c]	$ZnCl_2$	Cyclohexane	< 5

[a]Reaction conditions: **1a** (0.3 mmol), **2a** (0.3 mmol), cat. (0.03 mmol, 10 mol %), DCM (2.0 mL), 25 °C for 12 h. [b]Yields determined by ^1H NMR analysis of the crude mixture using 1,1,2,2 tetrachloroethane as the internal standard. [c]2.0 equiv of **1a** was used.

Our work commenced by studying the reaction between indole **1a** and cyclic 1,2-diaza-1,3-diene **2a** (Table S1). No reaction took place, and both compounds remained inactive in the absence of Lewis acid catalyst. A series of Lewis acid catalysts [such as $Sc(OTf)_3$, $Zn(OAc)_2$, $ZnSO_4$, $Zn(OTf)_2$, $SmCl_3·6H_2O$, $LiClO_4$, LiCl, $CuCl_2$, $Cu(OTf)_2$, $CuBr_2$, $InBr_3$,

$ZnBr_2$, and $ZnCl_2$] and solvents [such as dichloromethane, acetone, tetrahydrofuran, acetonitrile and cyclohexane] were examined, and the combination of $ZnCl_2$ and CH_2Cl_2 (heterogeneous catalytic system) was found to be superior for this transformation. Noteworthy, compound **3a** was obtained as a single regio- and diastereoisomer (50% yield).

The relative stereochemistry of cycloadducts **3** were assigned based on the X-ray diffraction analysis of compound **3e**.

3. X-ray structure of compound 3e (CCDC: 1950877)

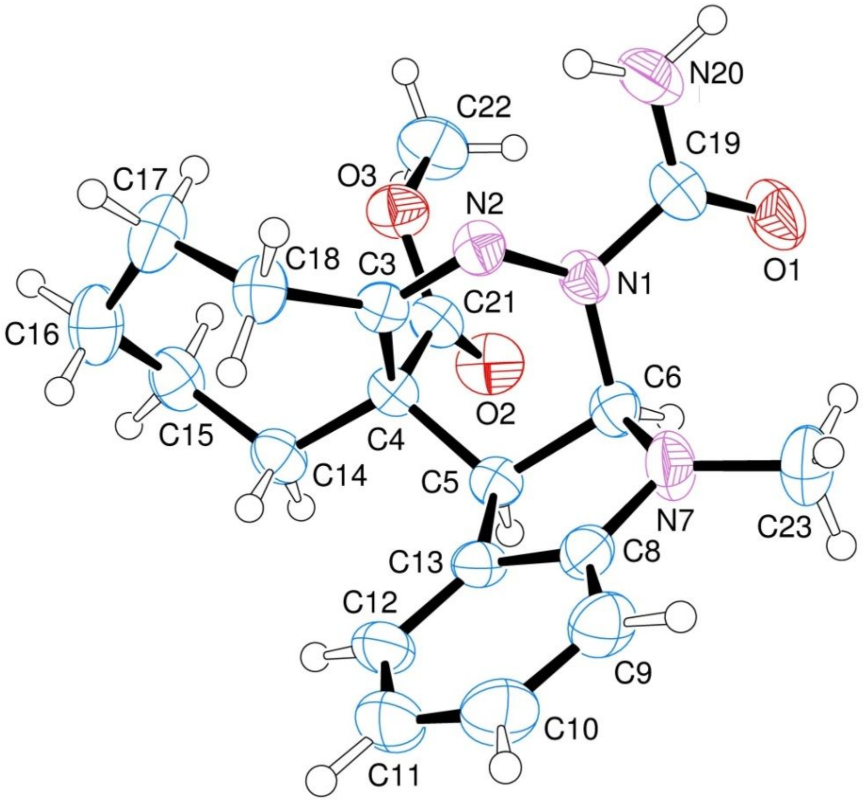

Ellipsoids enclose 50% probability

4. ^{1}H and ^{13}C NMR spectra of products.

^{1}H NMR of 3a (400 MHz, DMSO-d_6)

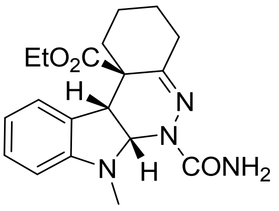

3a

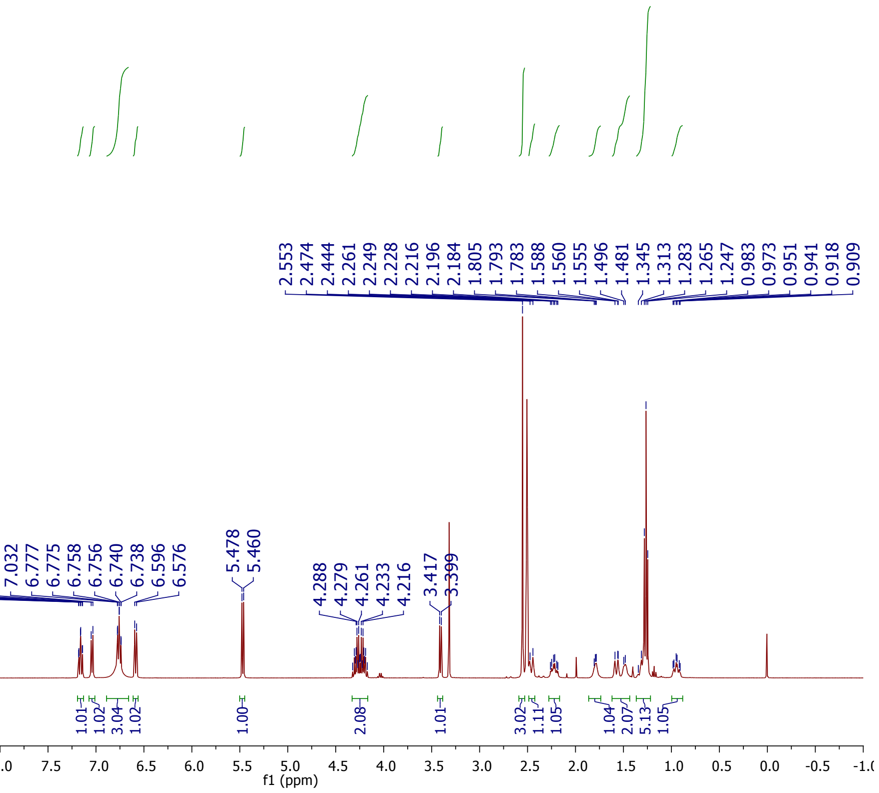

^{13}C NMR of 3a (100 MHz, $CDCl_3$)

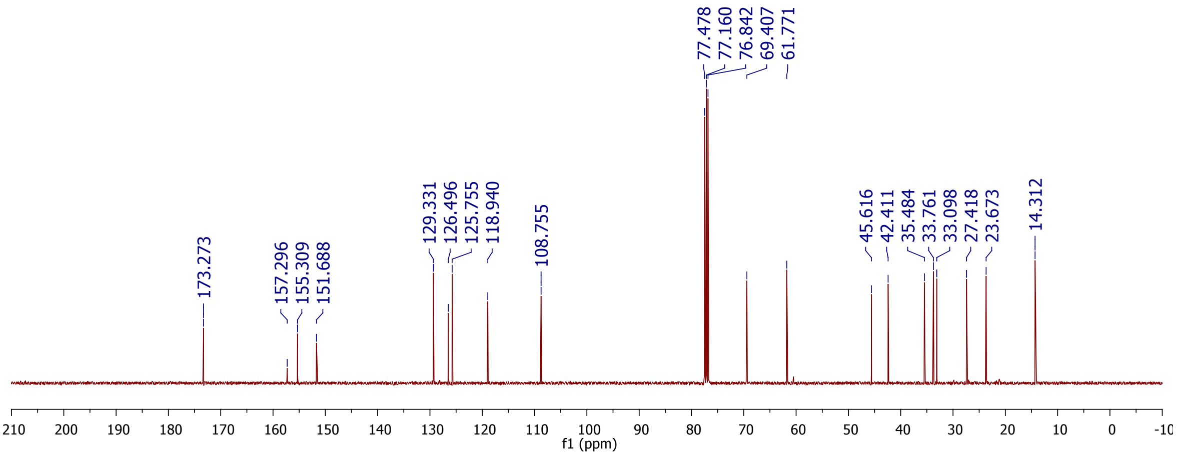

^{1}H-^{1}H COSY NMR of 3a (400 MHz, DMSO-d_6)

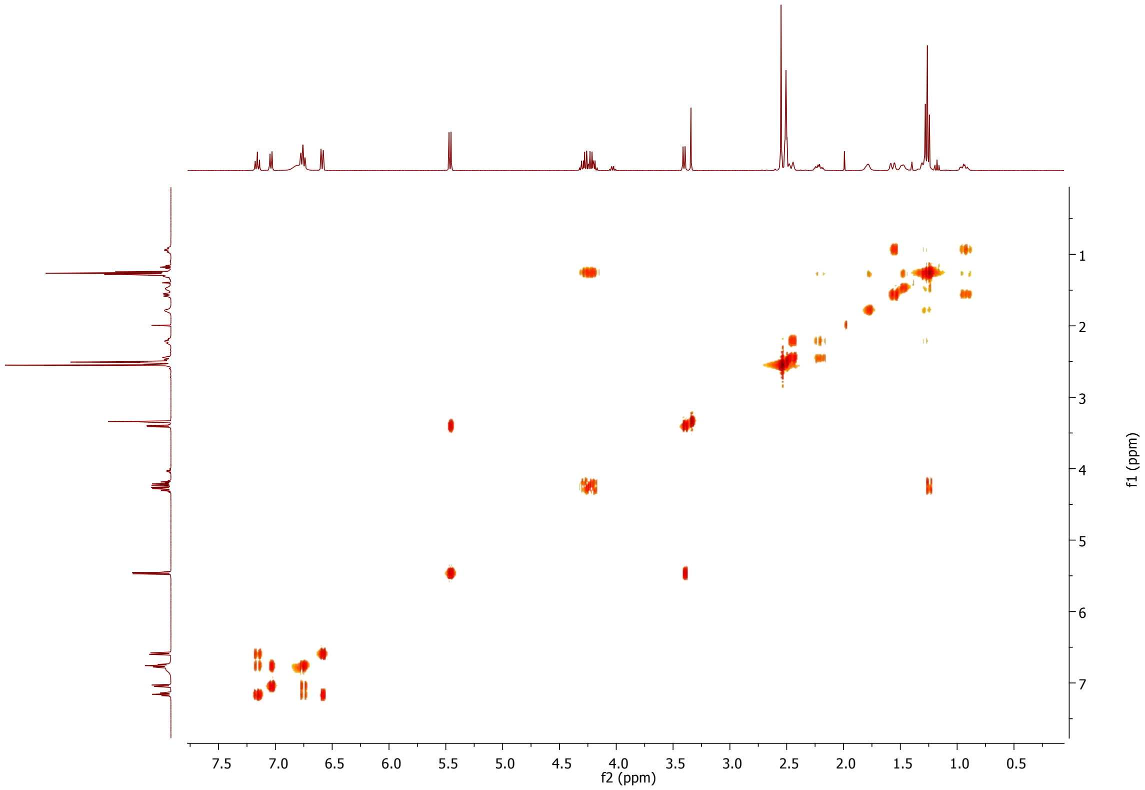

HMQC NMR of 3a (400 MHz, DMSO-d_6)

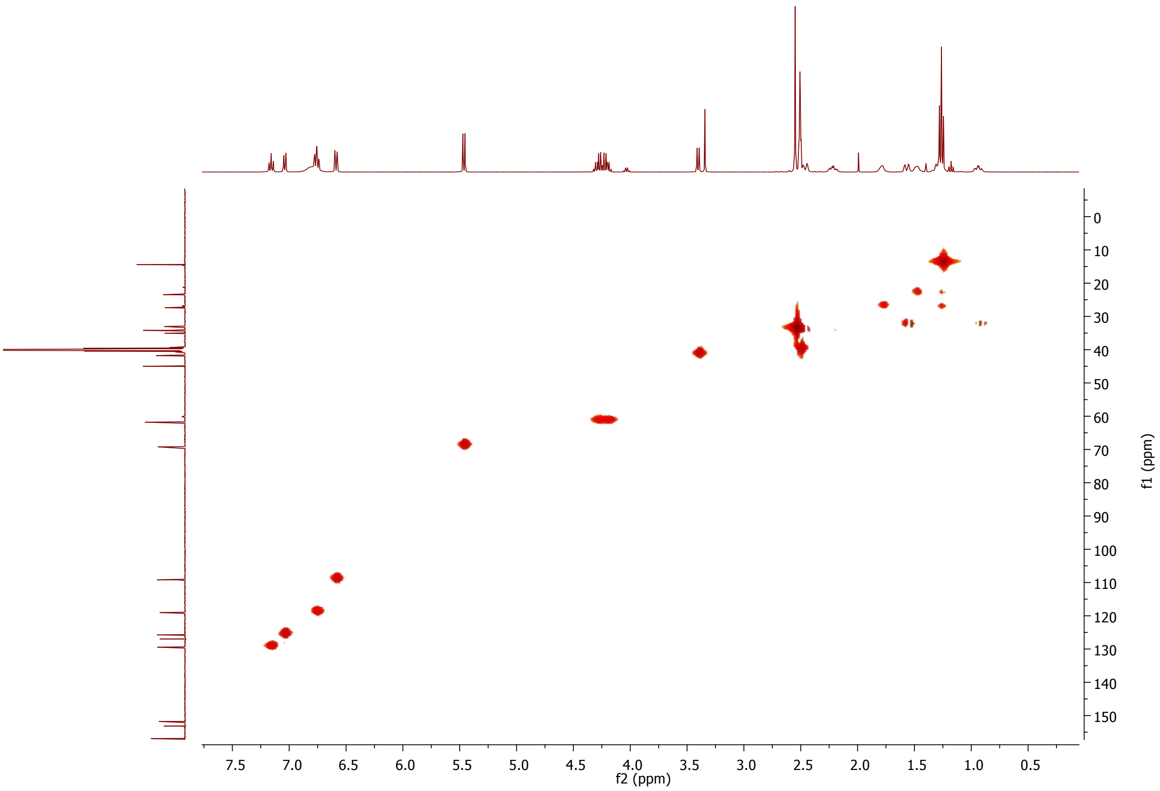

HMBC NMR of 3a (400 MHz, DMSO-d_6)

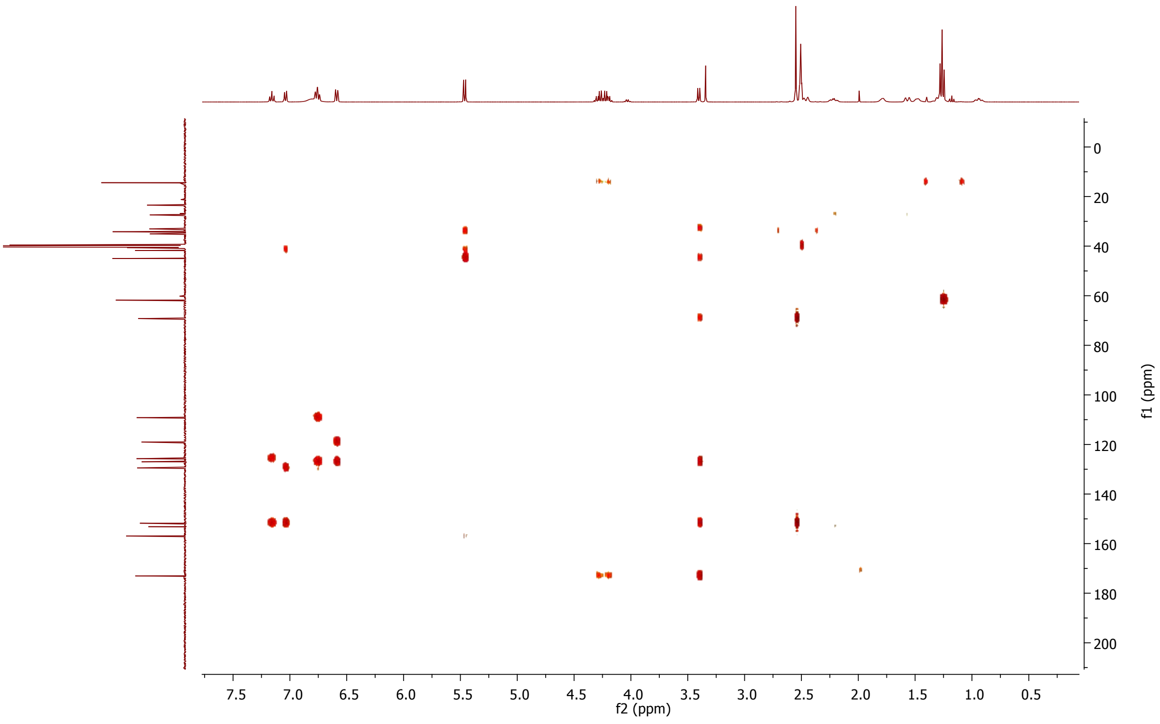

^{1}H NMR of 3b (400 MHz, DMSO-d_6)

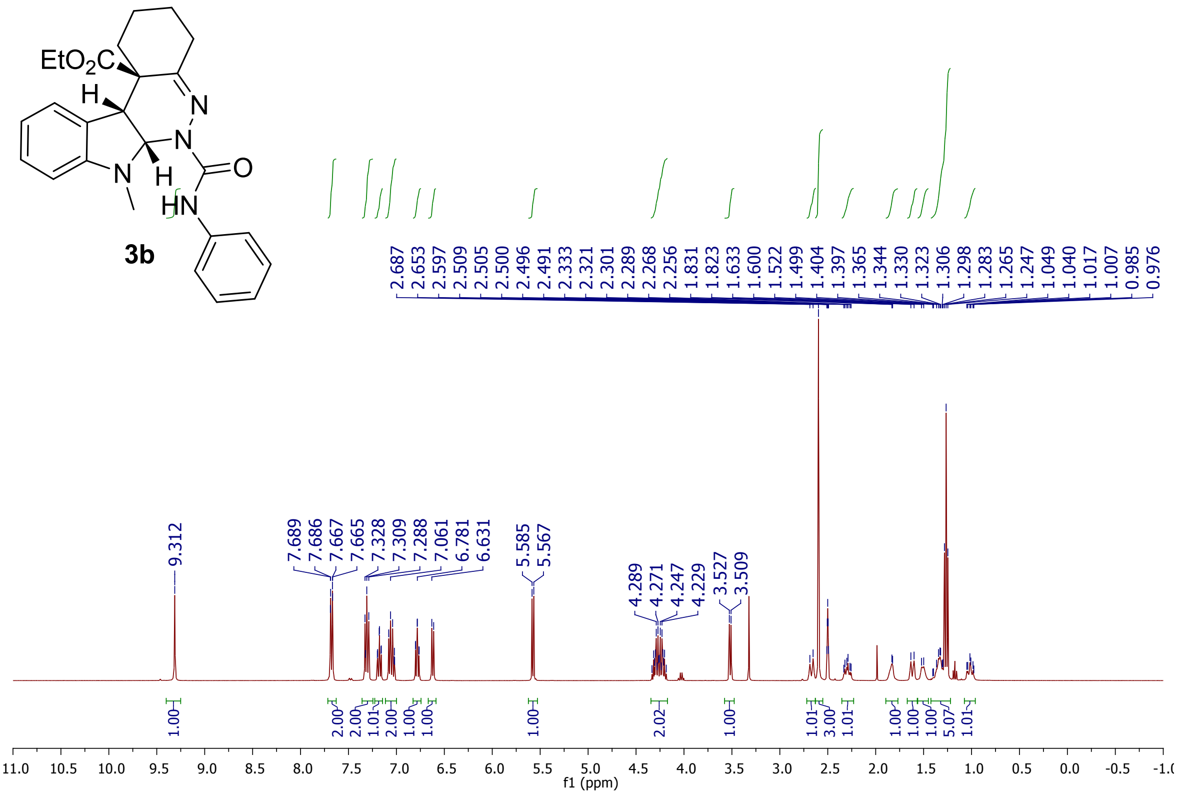

^{13}C NMR of 3b (100 MHz, DMSO-d_6)

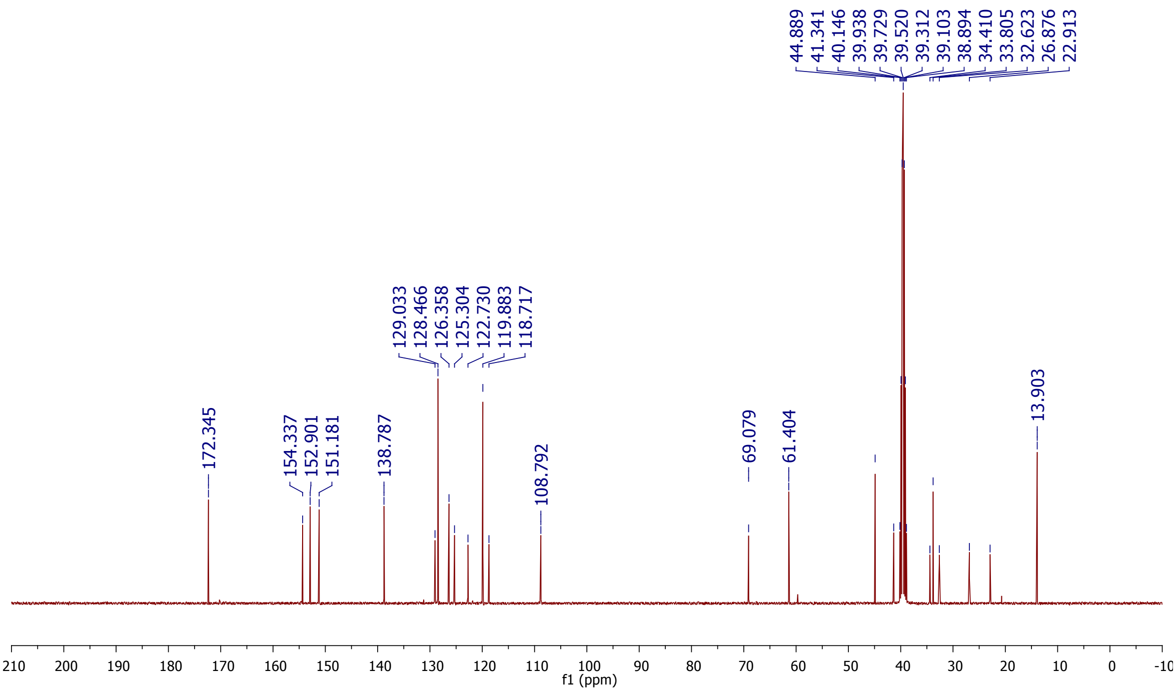

^{1}H NMR of 3c (400 MHz, DMSO-d_6)

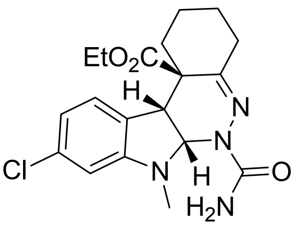

3c

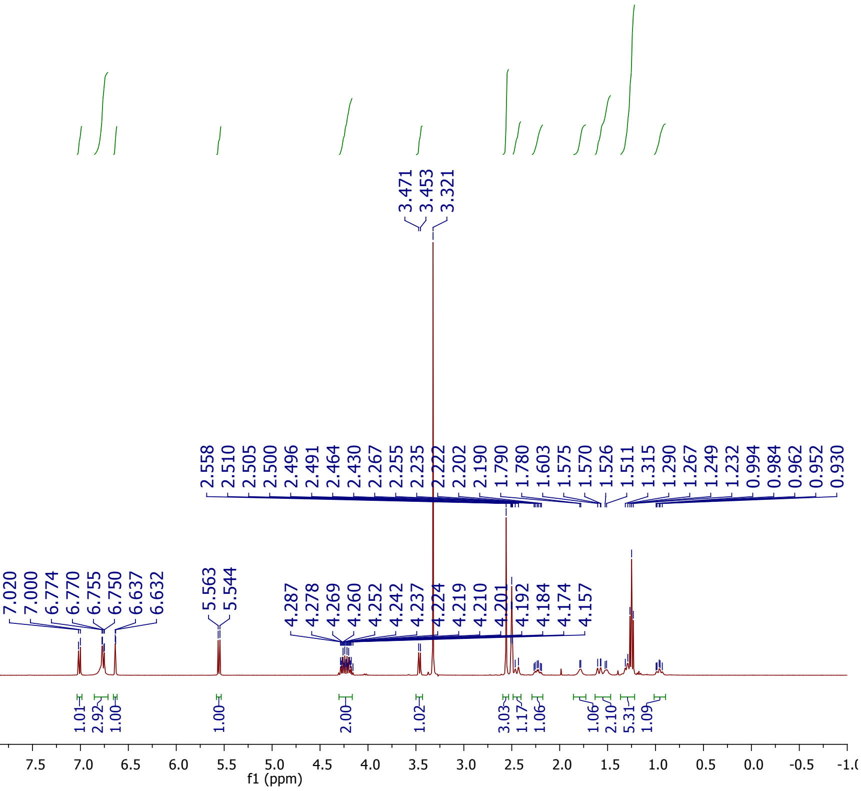

^{13}C NMR of 3c (100 MHz, DMSO-d_6)

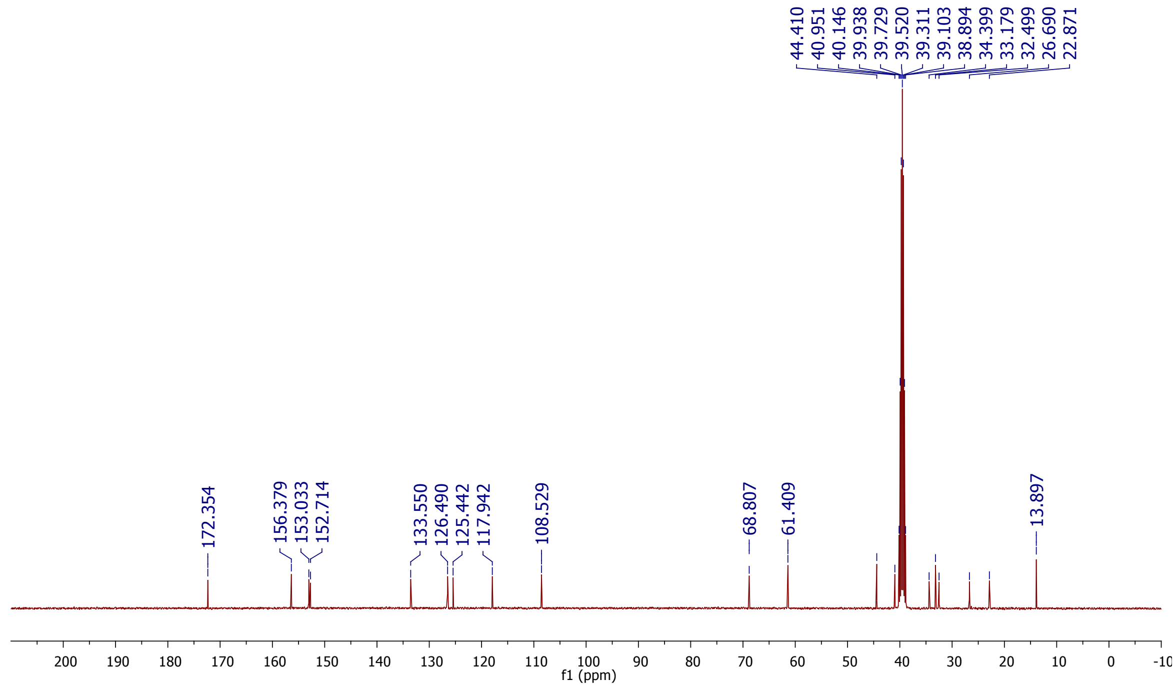

^{1}H NMR of 3d (400 MHz, DMSO-d_6)

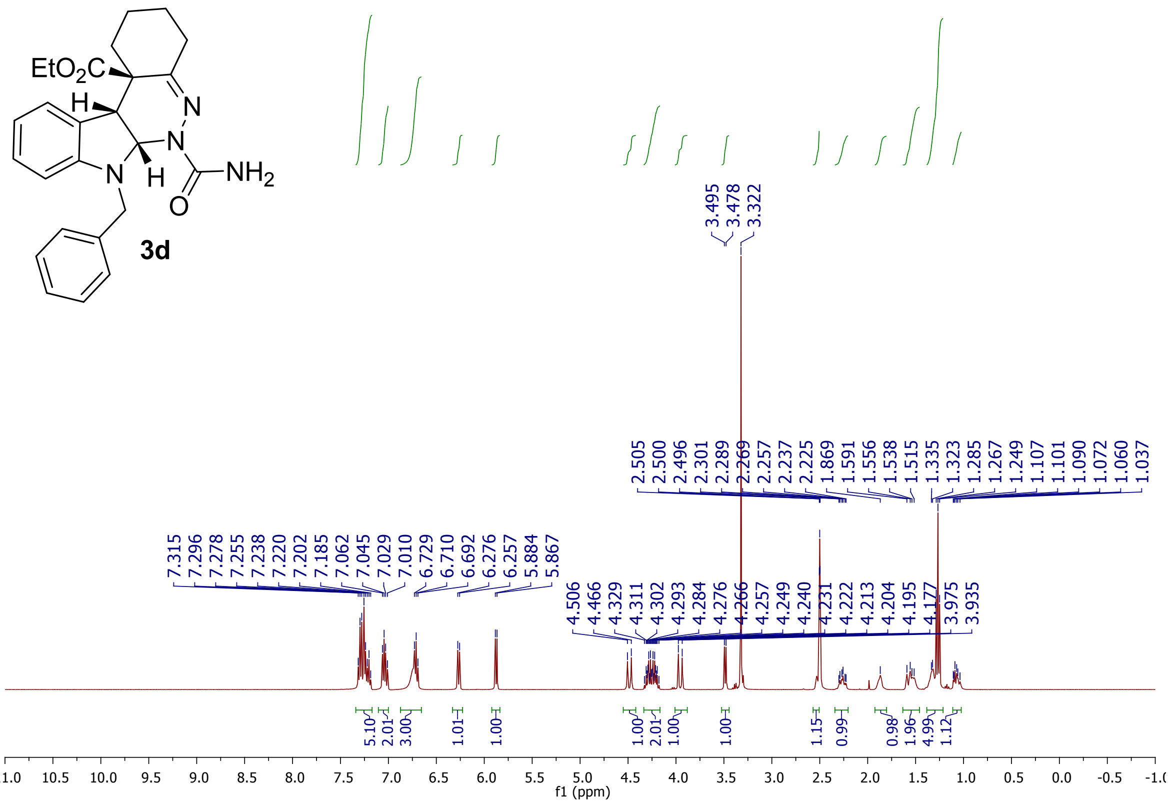

^{13}C NMR of 3d (100 MHz, DMSO-d_6)

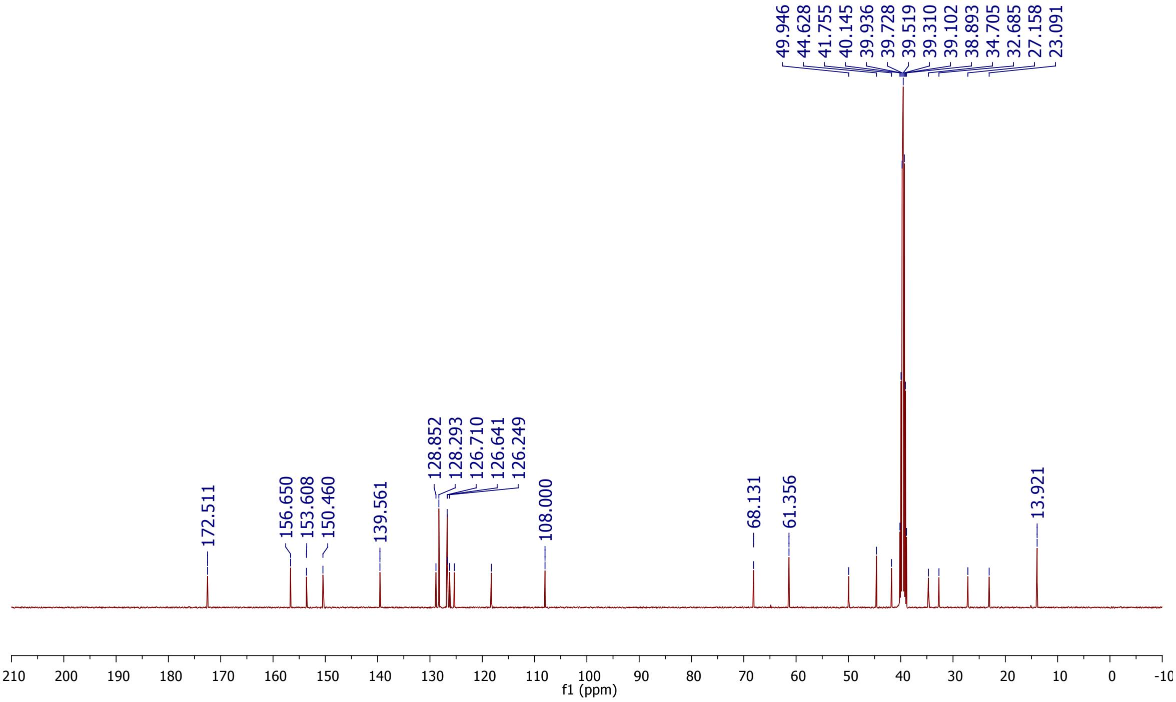

NOESY NMR of 3d (400 MHz, DMSO-d_6)

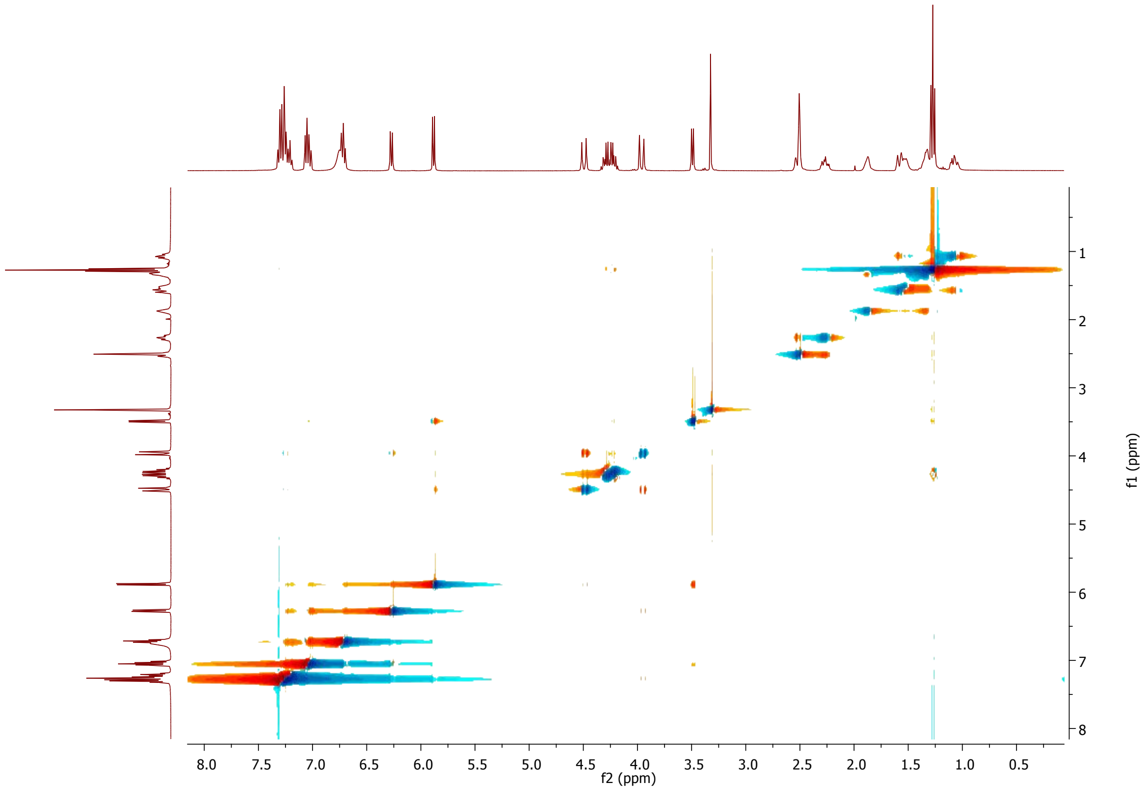

^{1}H NMR of 3e (400 MHz, DMSO-d_6)

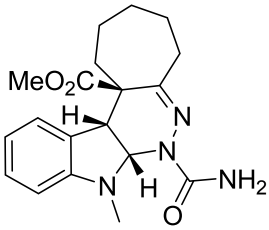

3e

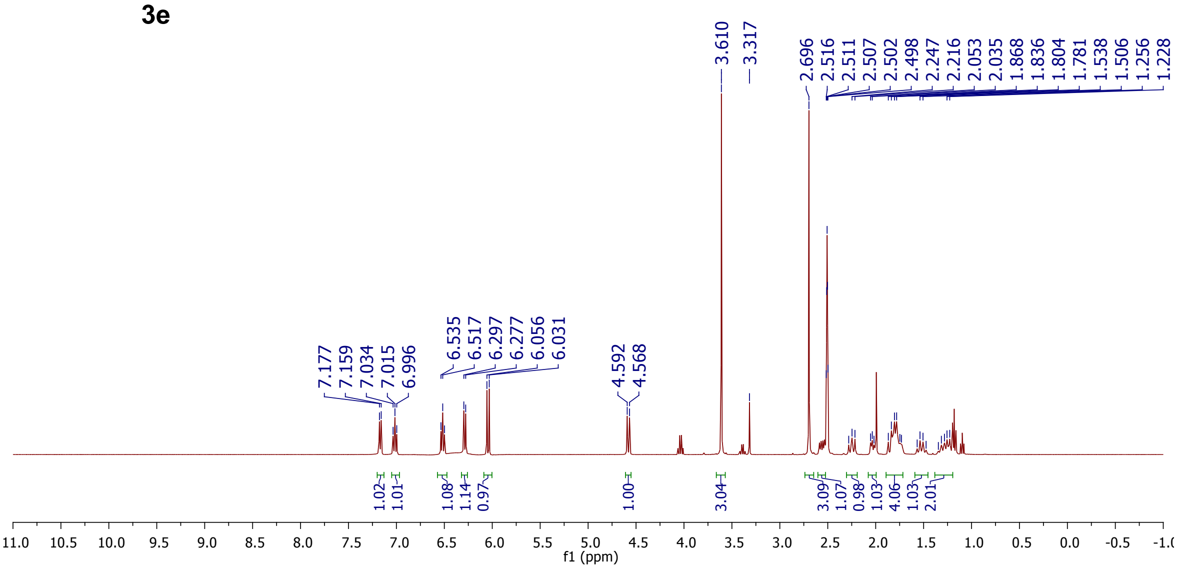

^{13}C NMR of 3e (100 MHz, DMSO-d_6)

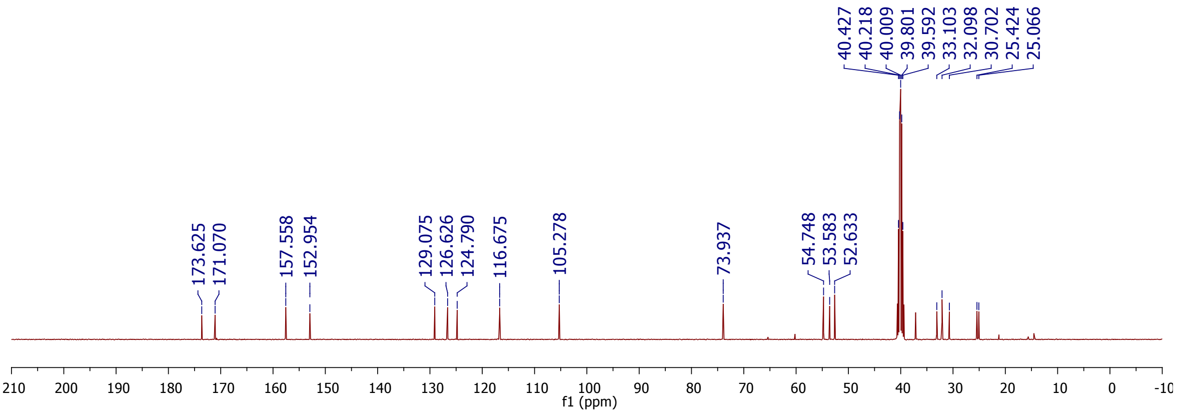

^{1}H NMR of 3f (400 MHz, DMSO-d_6)

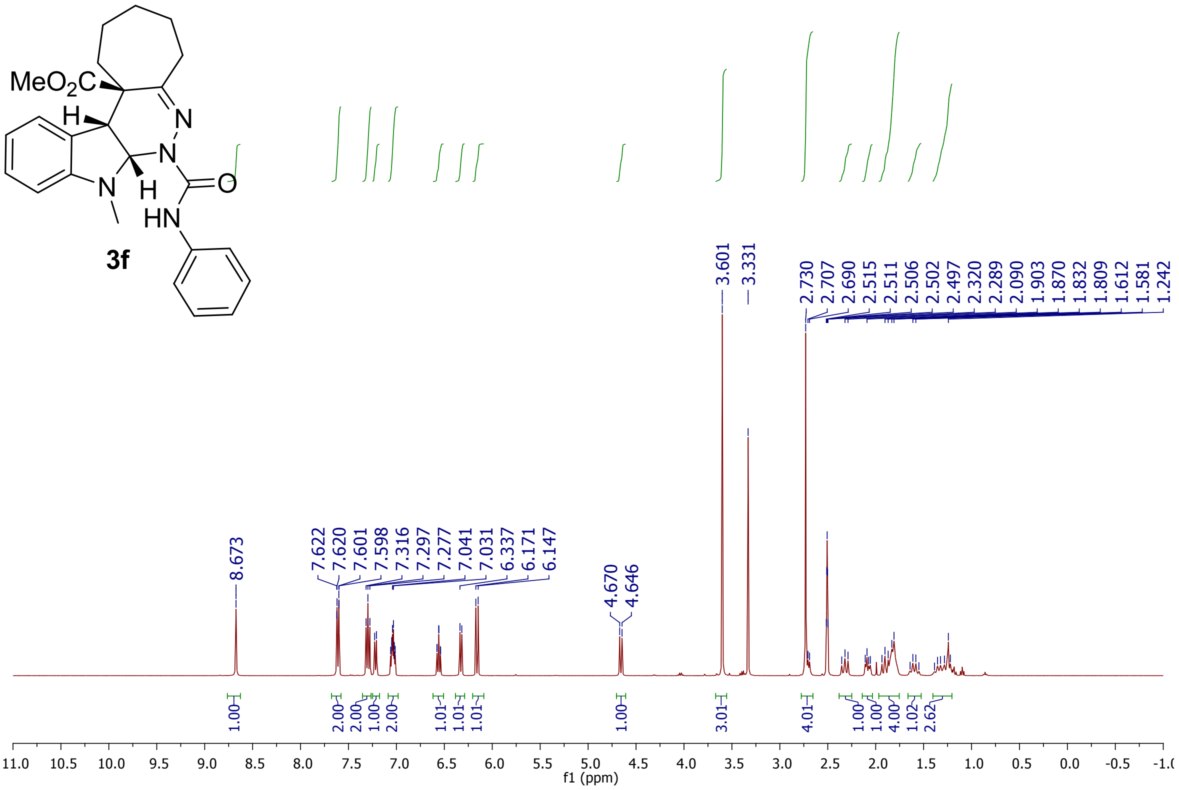

^{13}C NMR of 3f (100 MHz, DMSO-d_6)

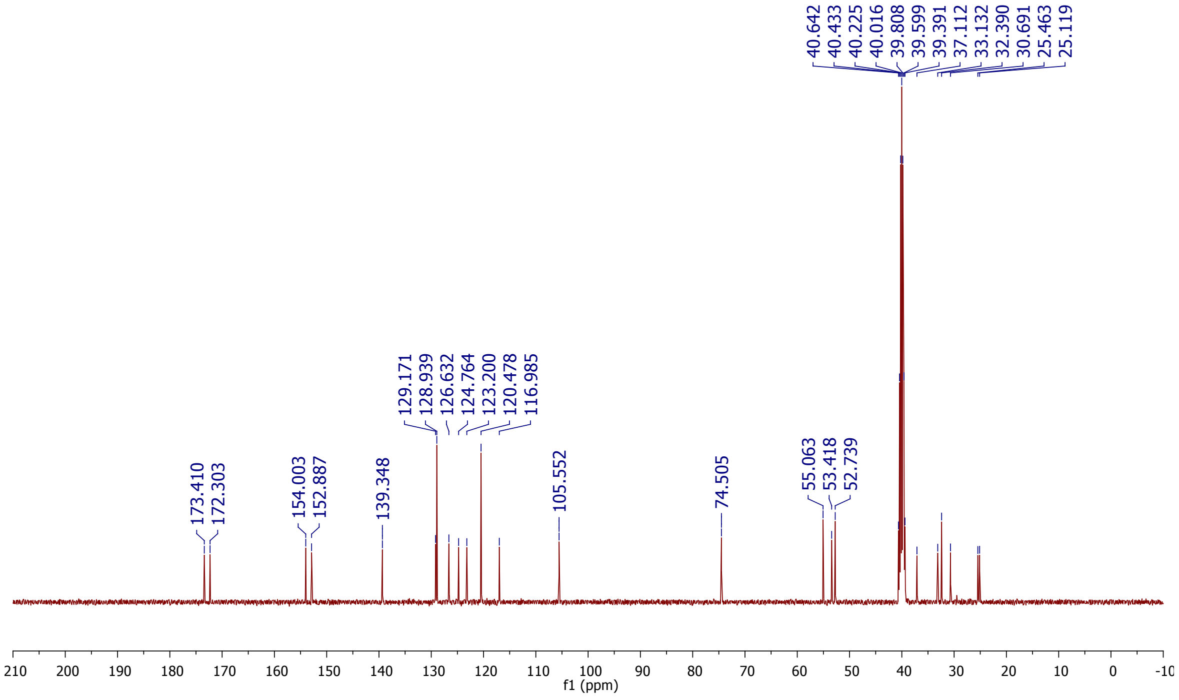

^{1}H NMR of 3g (400 MHz, DMSO-d_6)

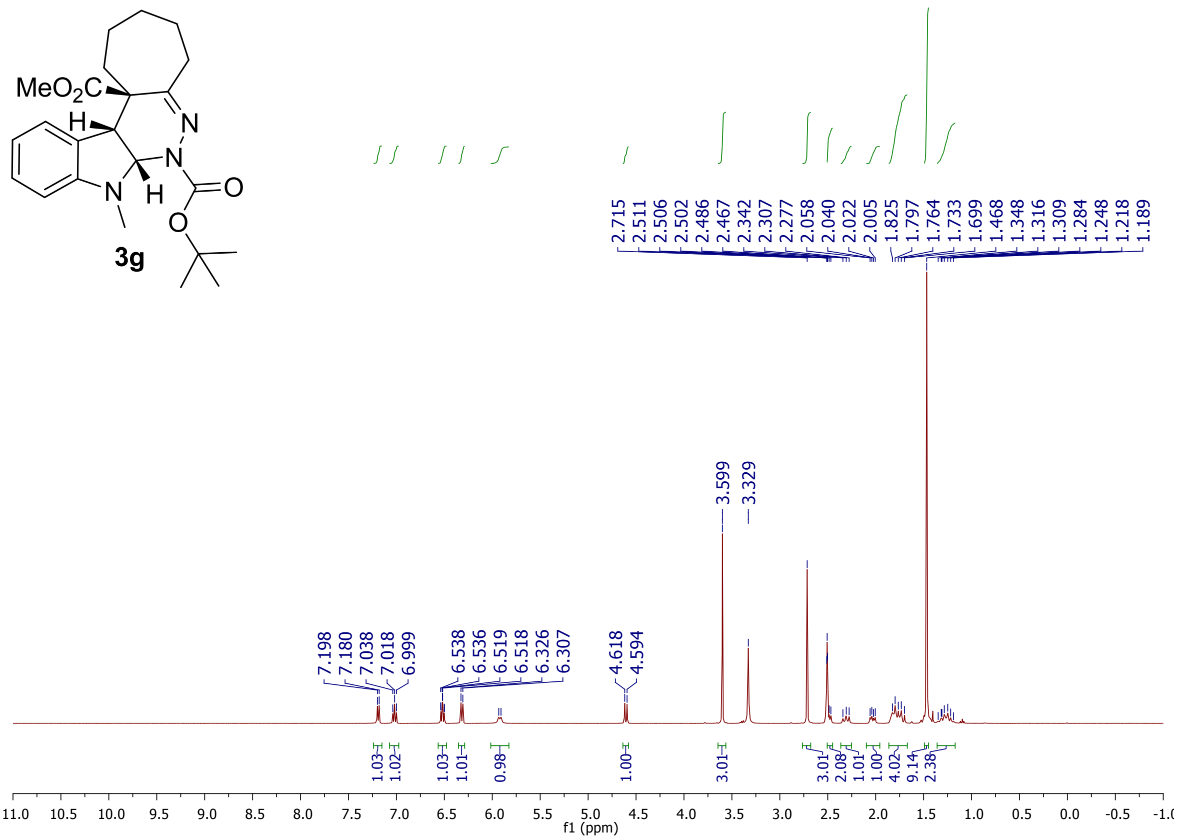

^{13}C NMR of 3g (100 MHz, DMSO-d_6)

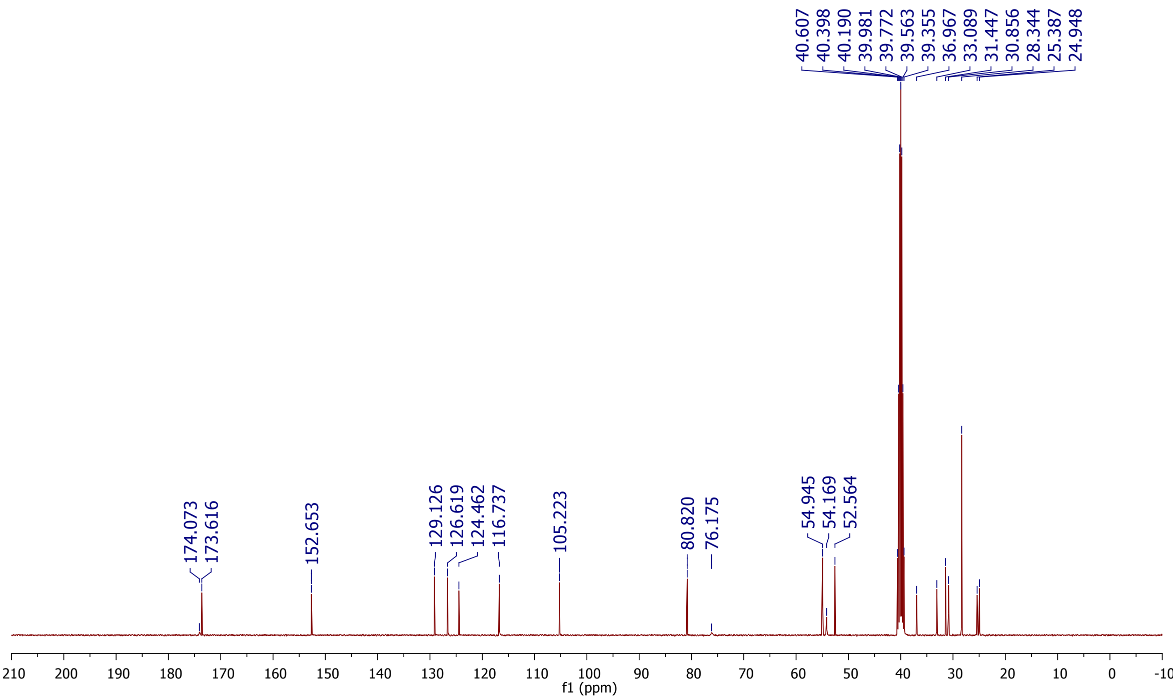

^{1}H NMR of 3h (400 MHz, DMSO-d_6)

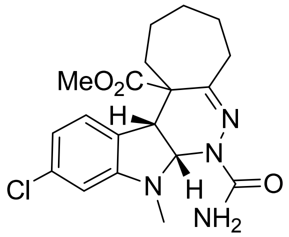

3h

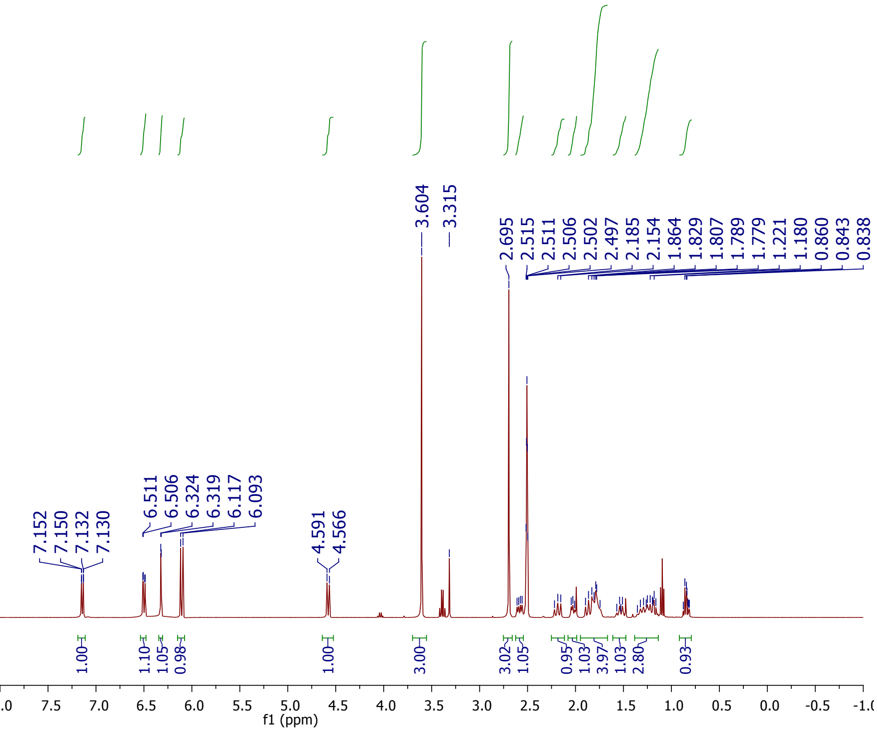

^{13}C NMR of 3h (100 MHz, DMSO-d_6)

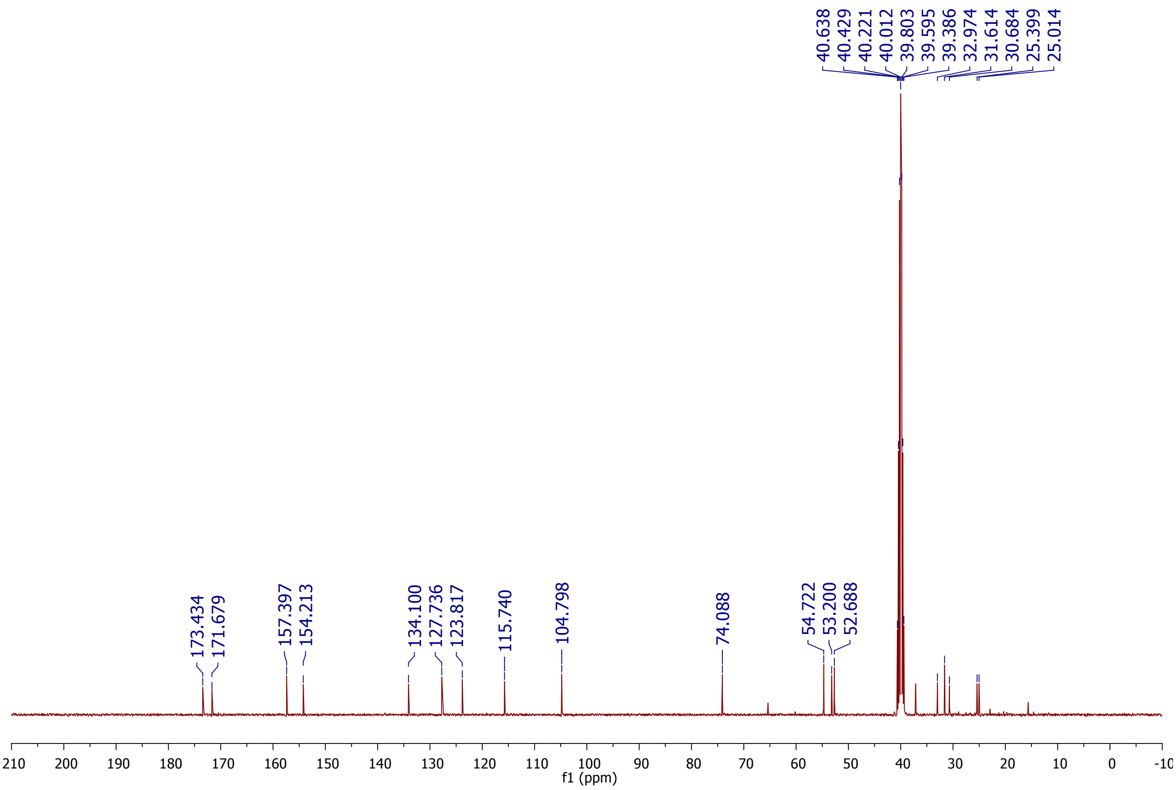

NOESY NMR of 3h (400 MHz, DMSO-d_6)

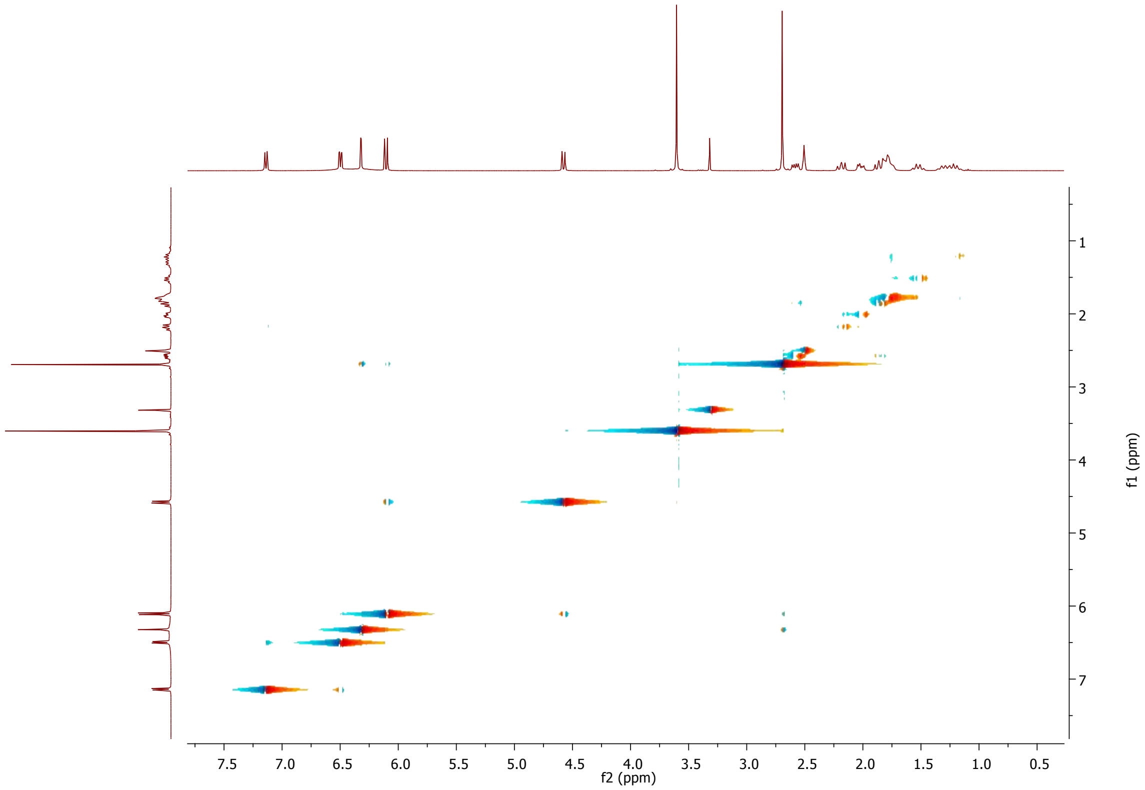

^{1}H NMR of 3i (400 MHz, DMSO-d_6)

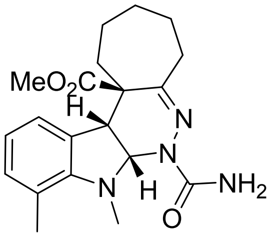

3i

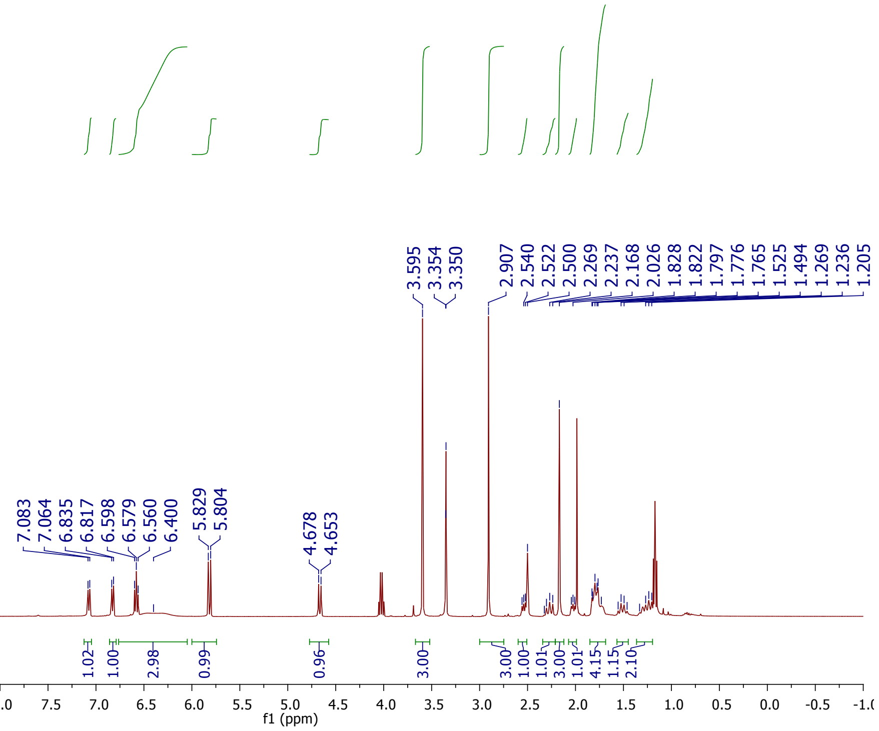

^{13}C NMR of 3i (100 MHz, DMSO-d_6)

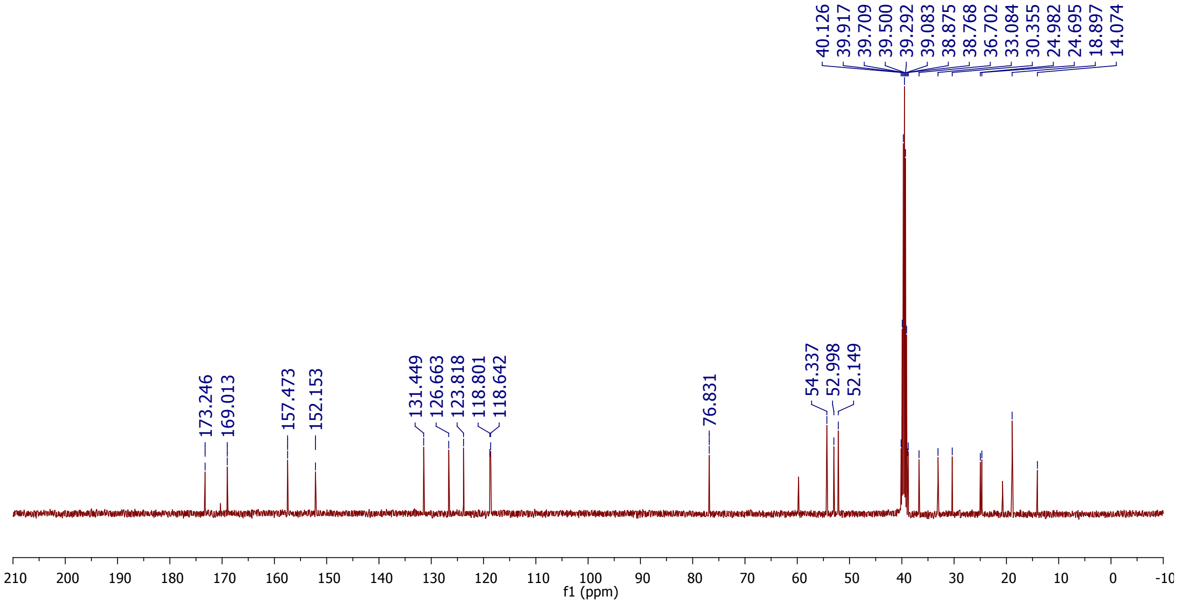

^{1}H NMR of 3j (400 MHz, DMSO-d_6)

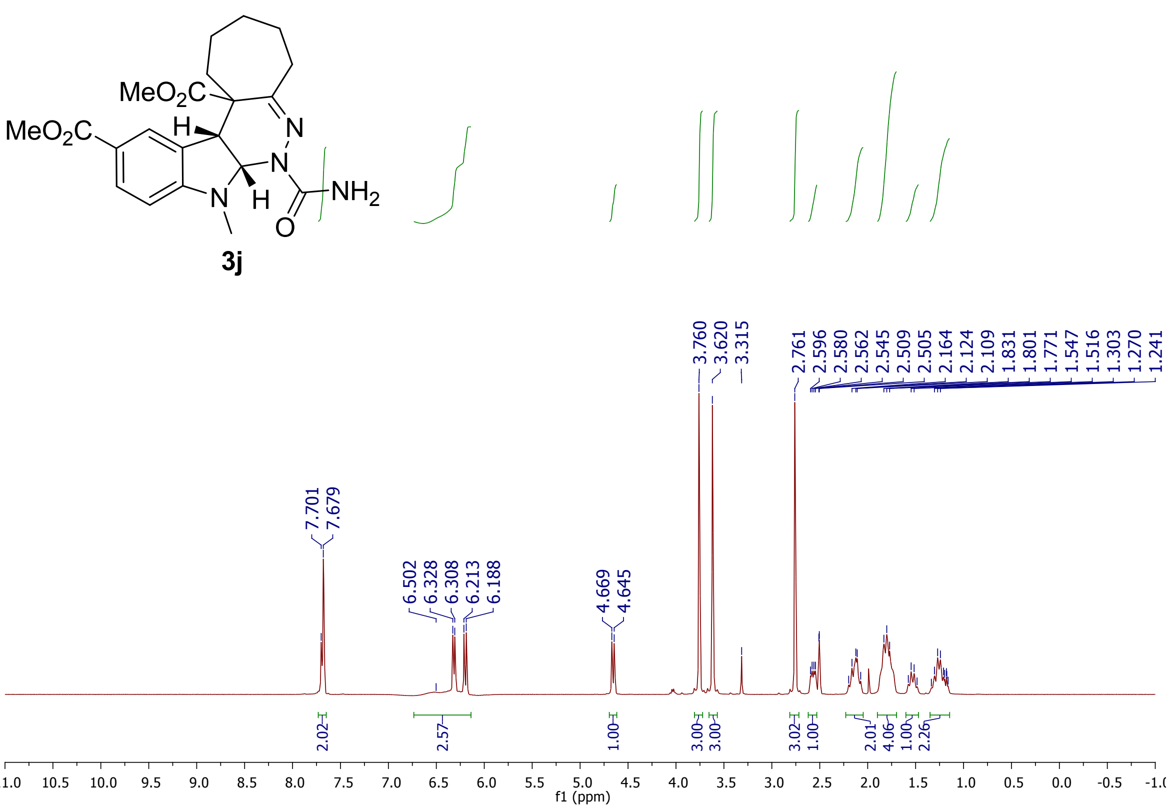

^{13}C NMR of 3j (100 MHz, DMSO-d_6)

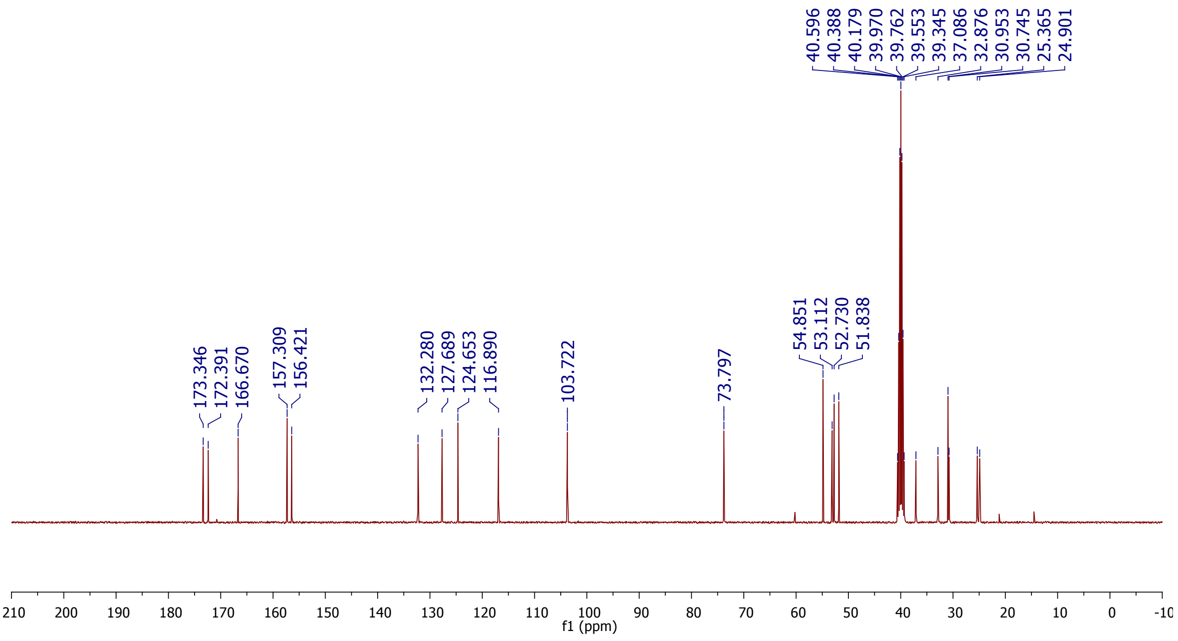

^{1}H NMR of 3k (400 MHz, DMSO-d_6)

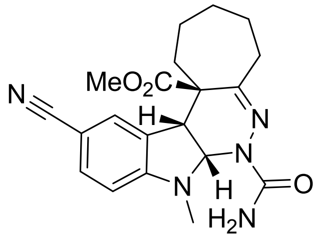

3k

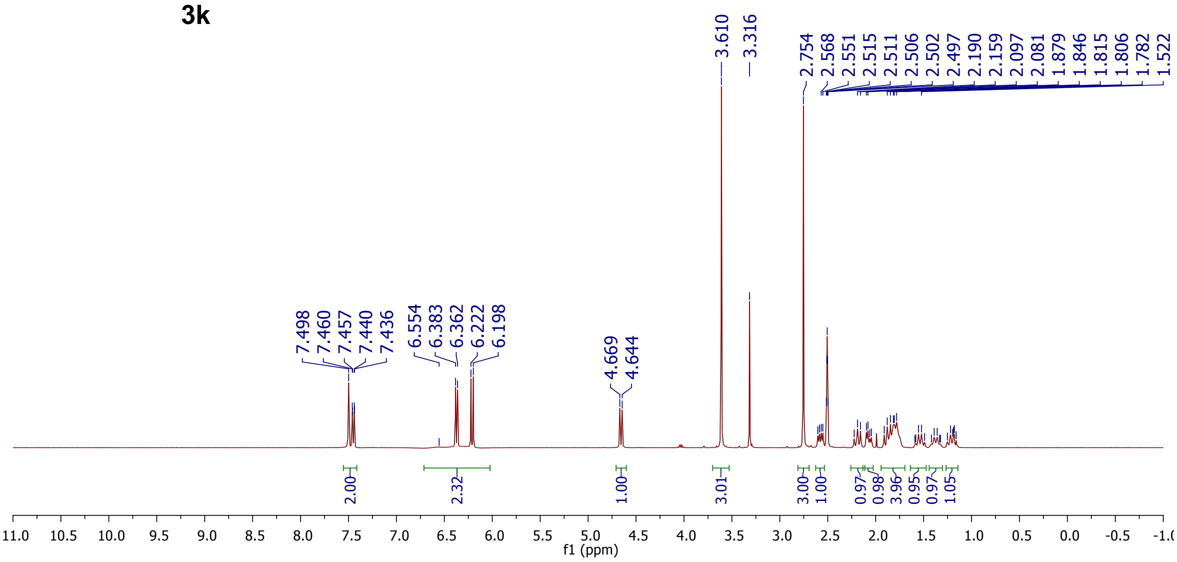

^{13}C NMR of 3k (100 MHz, DMSO-d_6)

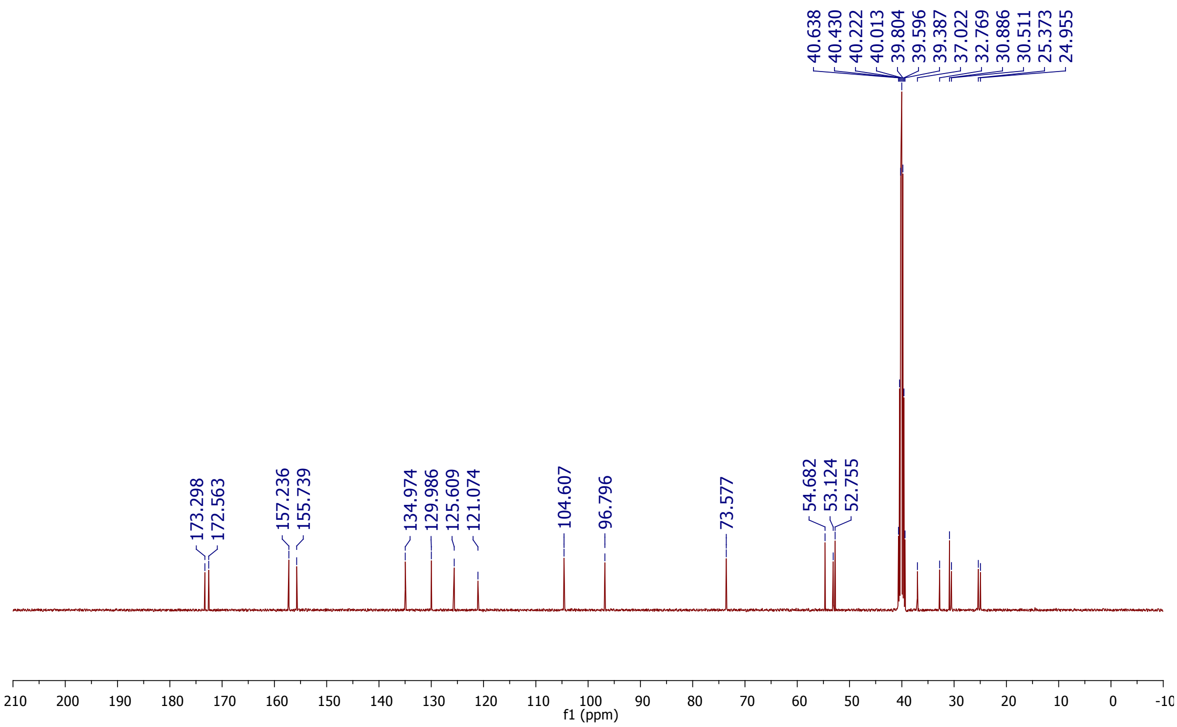

^{1}H NMR of 3l (400 MHz, DMSO-d_6)

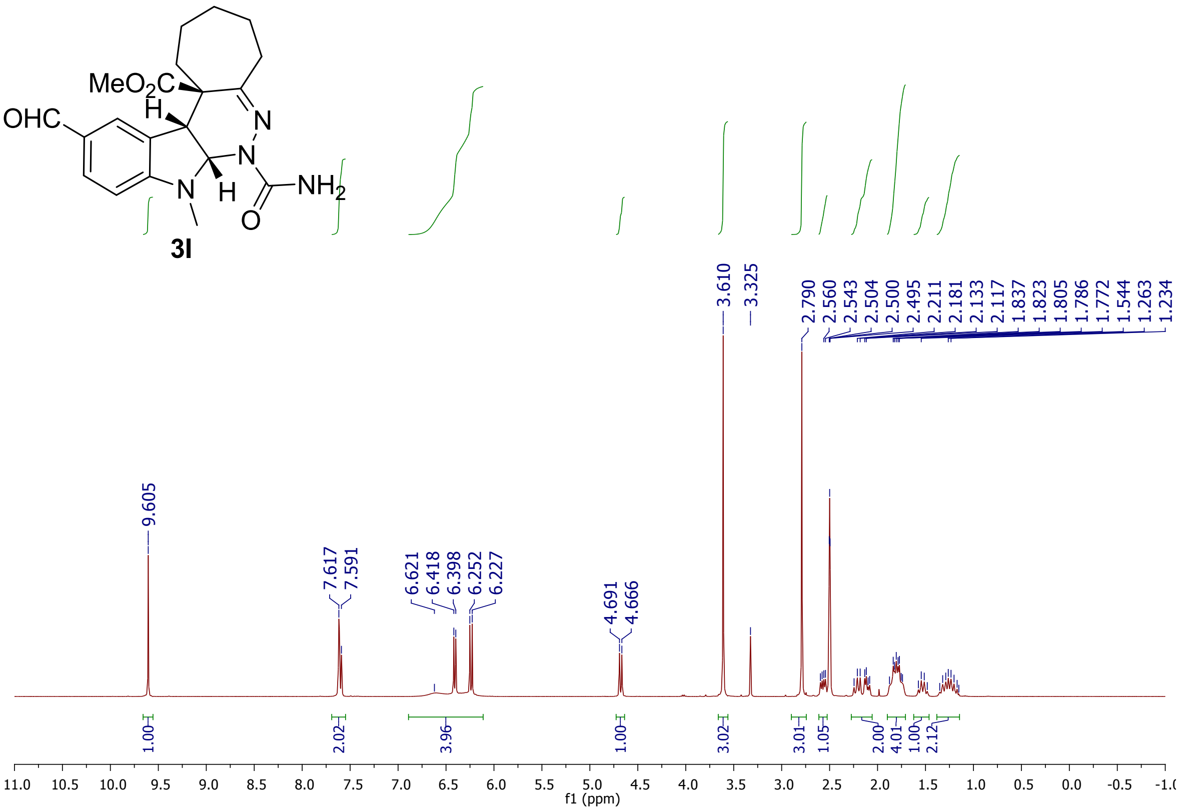

^{13}C NMR of 3l (100 MHz, DMSO-d_6)

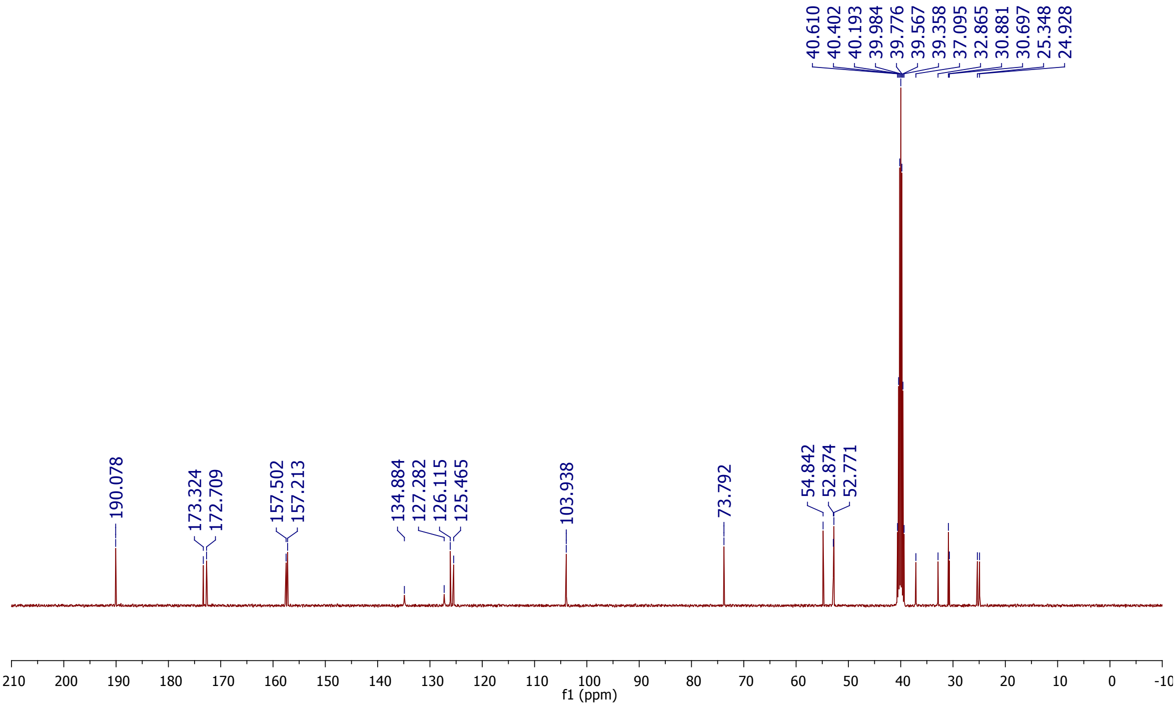

^{1}H NMR of 3m (400 MHz, DMSO-d_6)

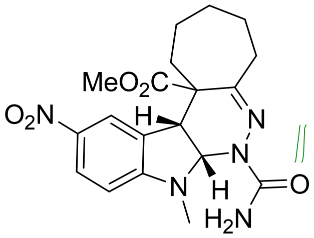

3m

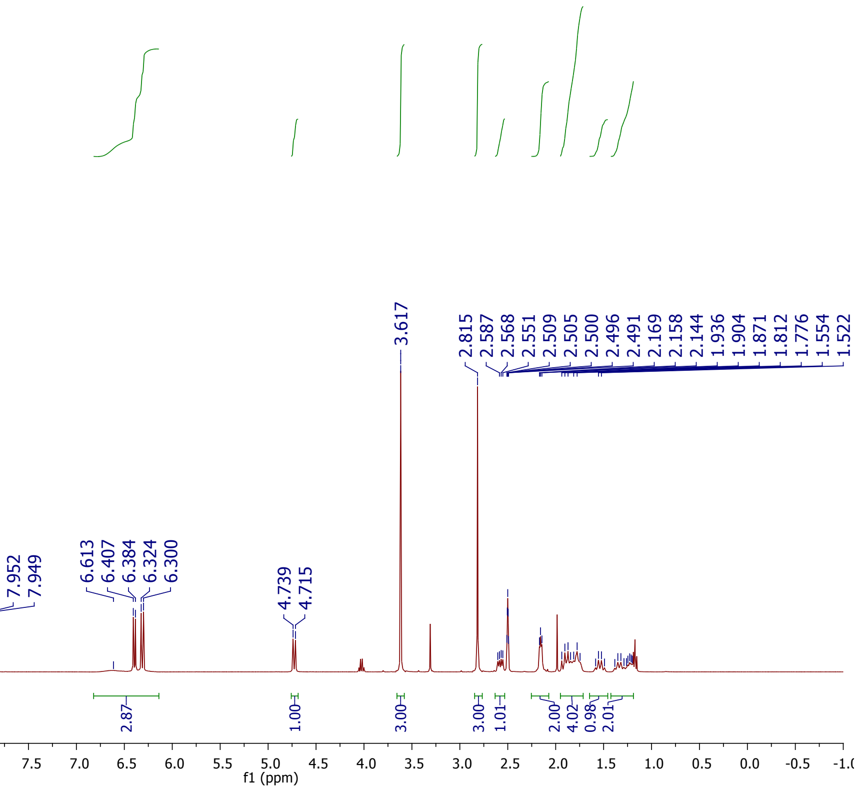

^{13}C NMR of 3m (100 MHz, DMSO-d_6)

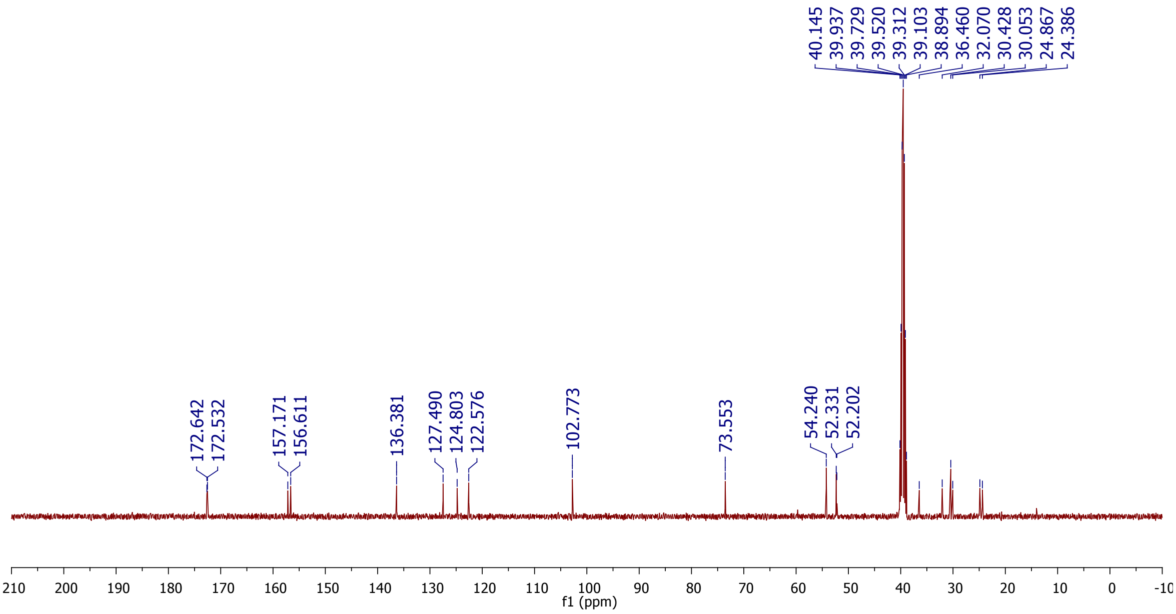

^{1}H NMR of 3n (400 MHz, DMSO-d_6)

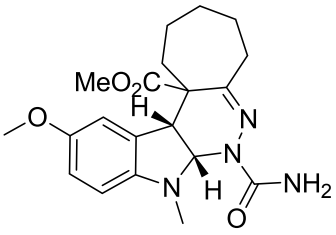

3n

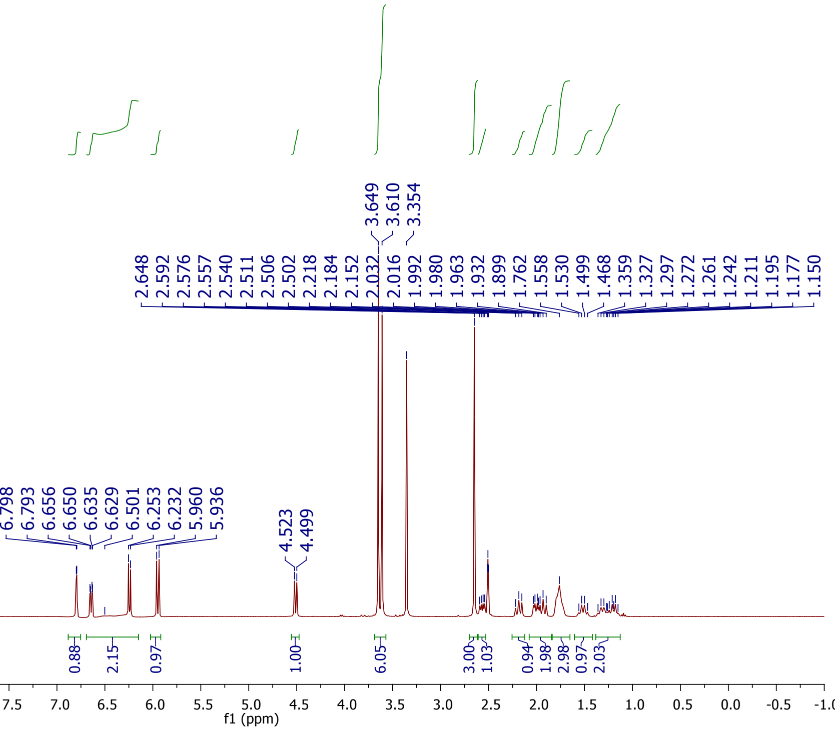

^{13}C NMR of 3n (100 MHz, DMSO-d_6)

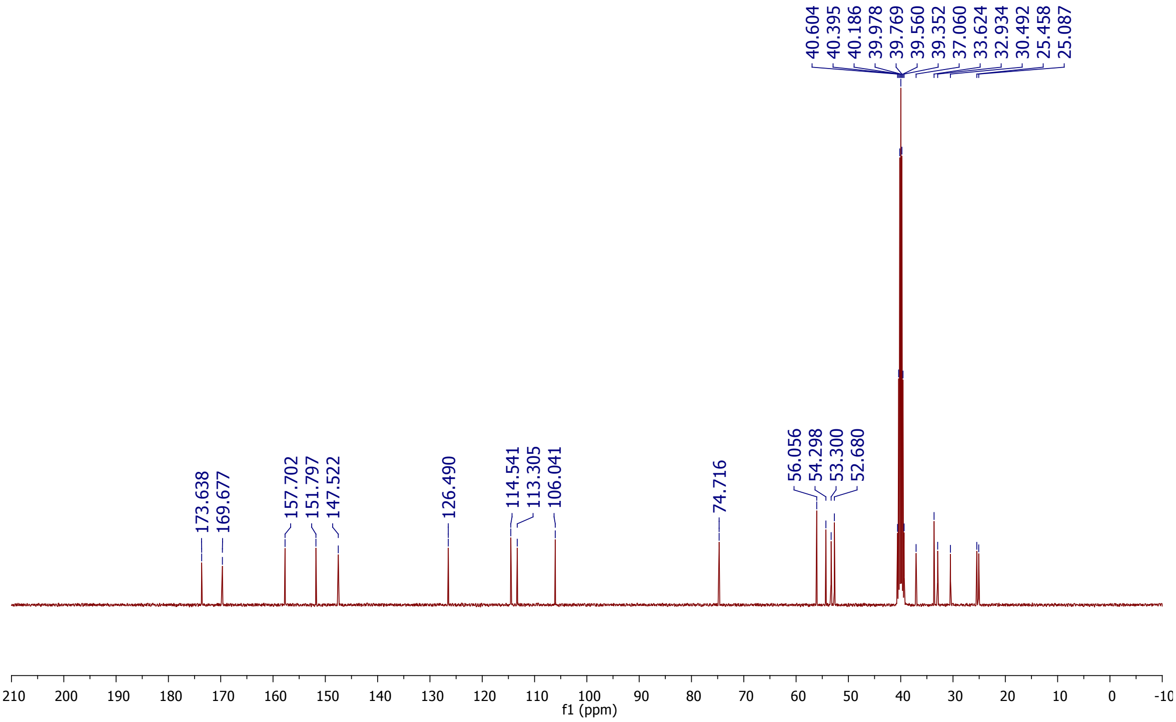

^{1}H NMR of 3o (400 MHz, DMSO-d_6)

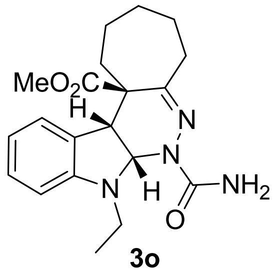

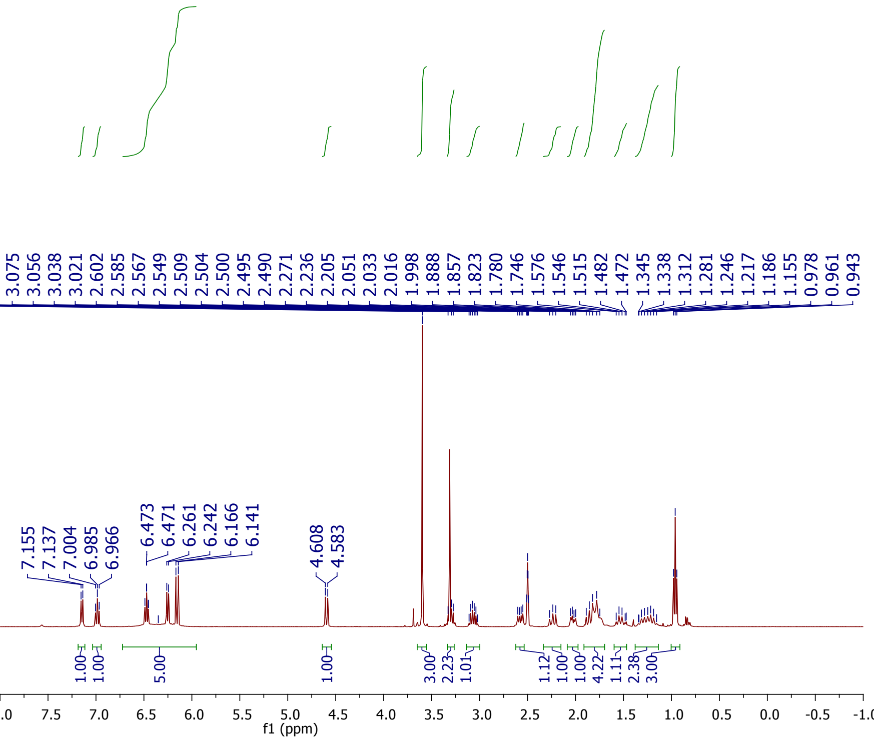

^{13}C NMR of 3o (100 MHz, DMSO-d_6)

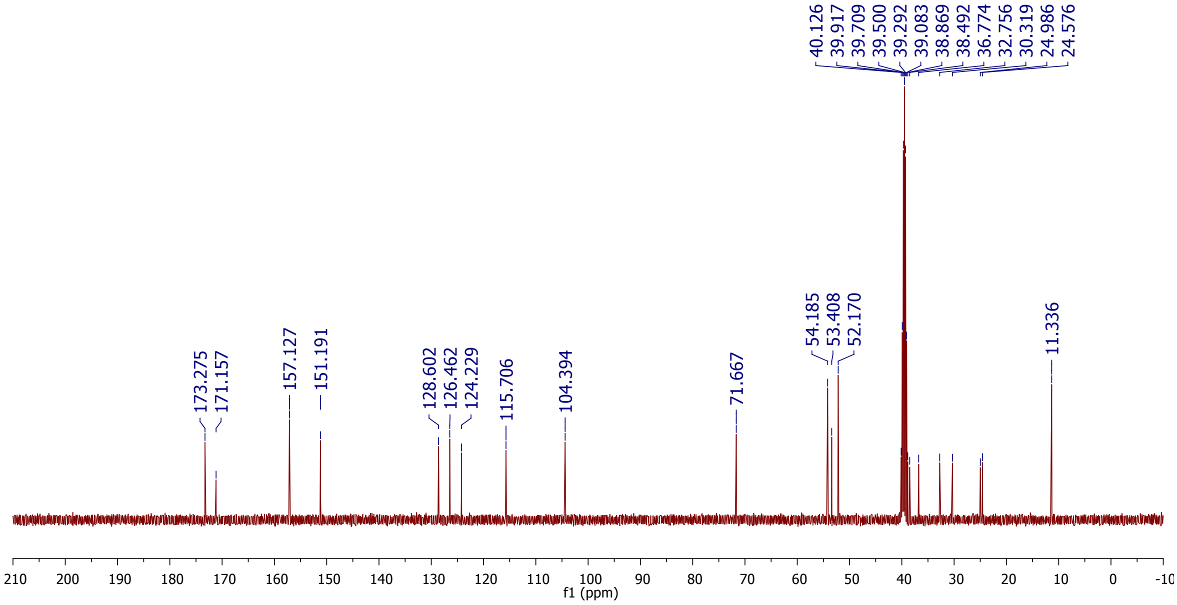

^{1}H NMR of 3p (400 MHz, DMSO-d_6)

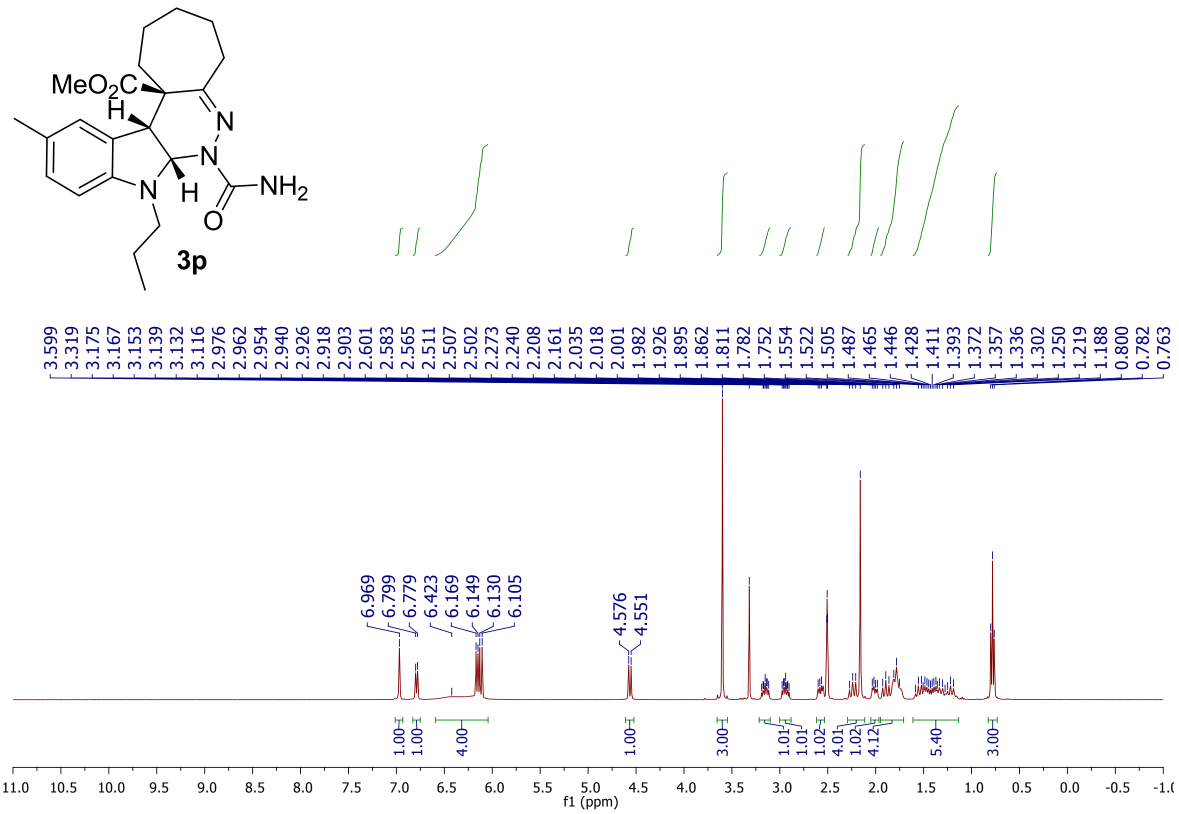

^{13}C NMR of 3p (100 MHz, DMSO-d_6)

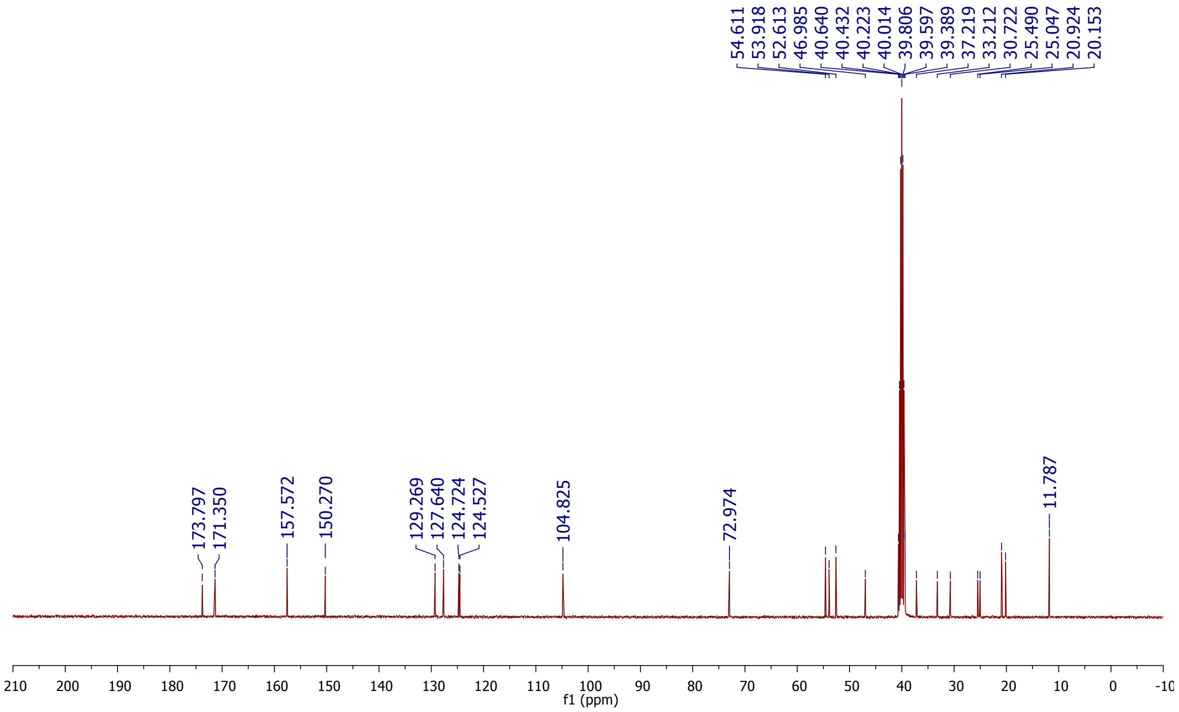

^{1}H NMR of 3q (400 MHz, DMSO-d_6)

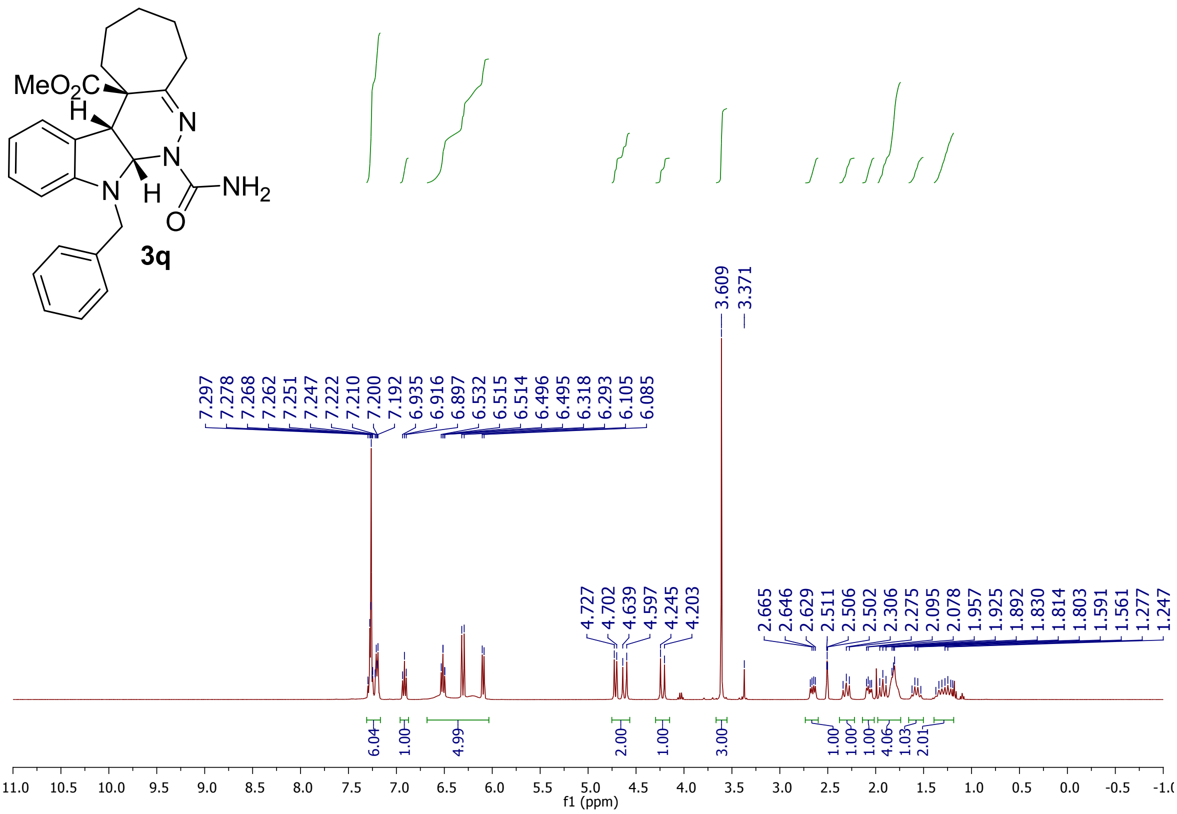

^{13}C NMR of 3q (100 MHz, DMSO-d_6)

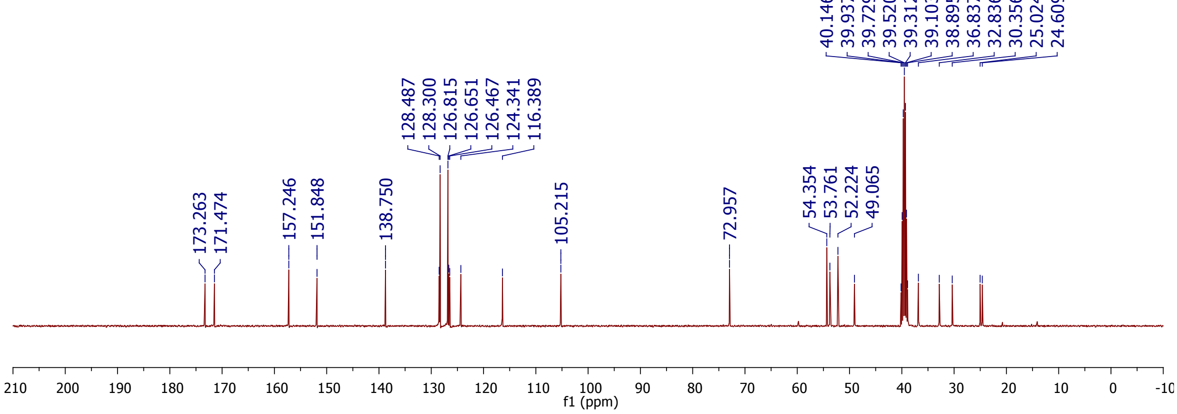

^{1}H NMR of 3r (400 MHz, DMSO-d_6)

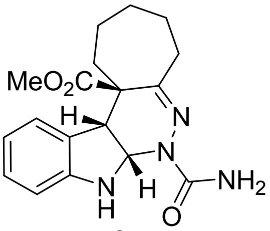

3r

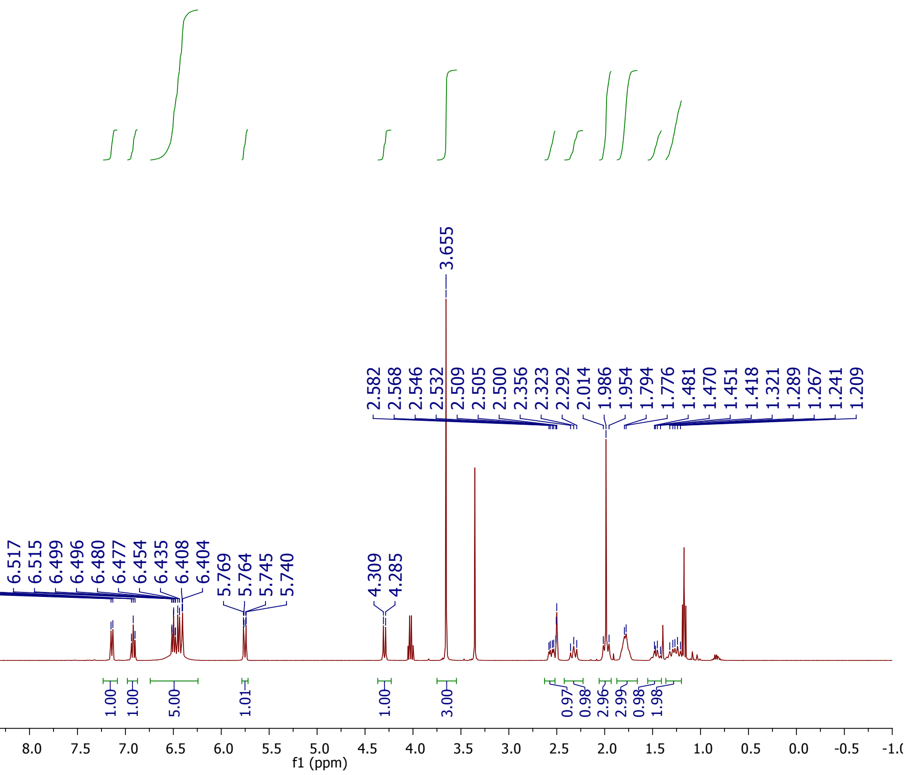

^{13}C NMR of 3r (100 MHz, DMSO-d_6)

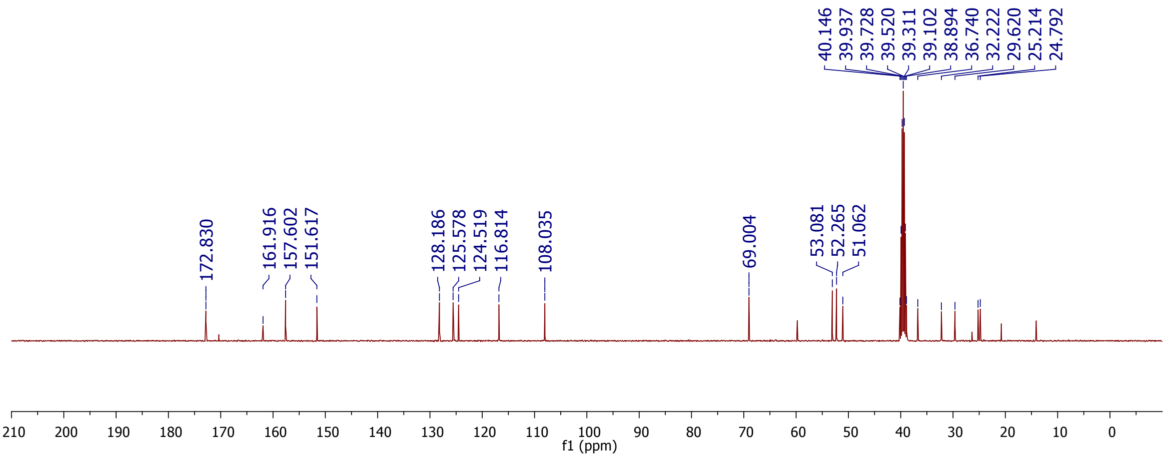

^{1}H NMR of 3s (400 MHz, DMSO-d_6)

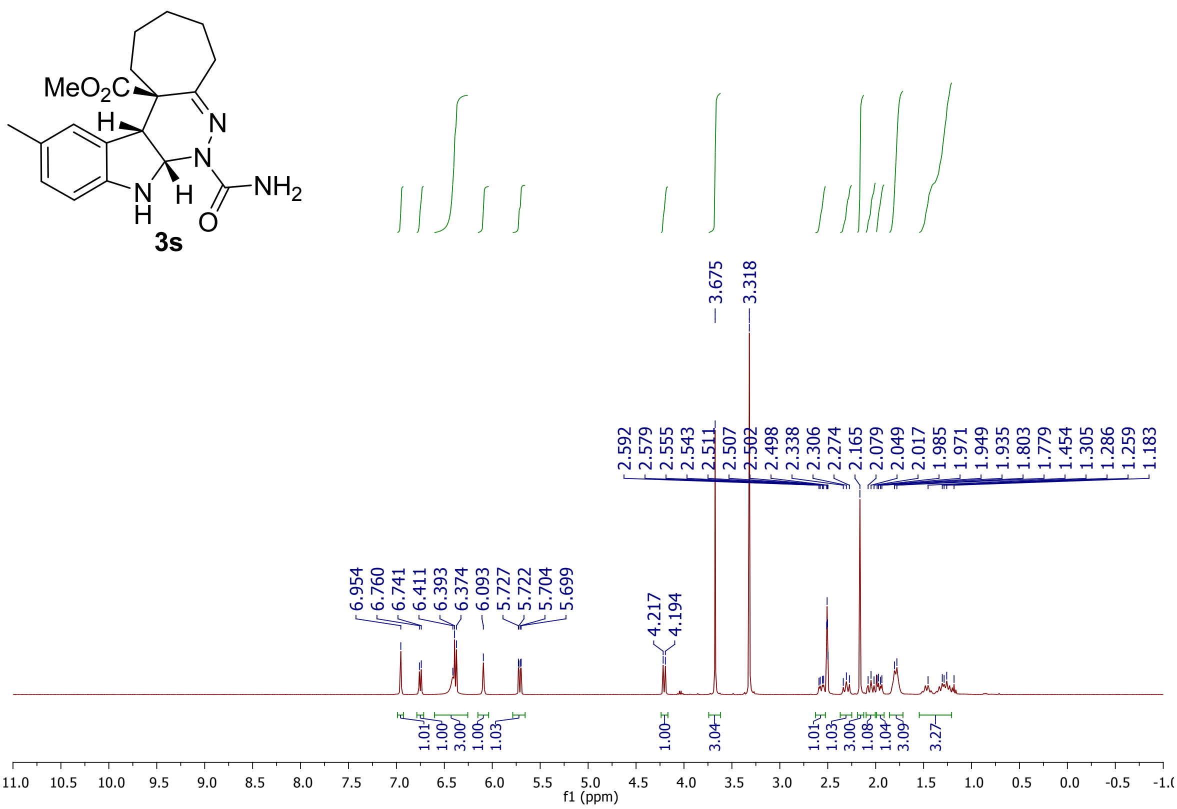

^{13}C NMR of 3s (100 MHz, DMSO-d_6)

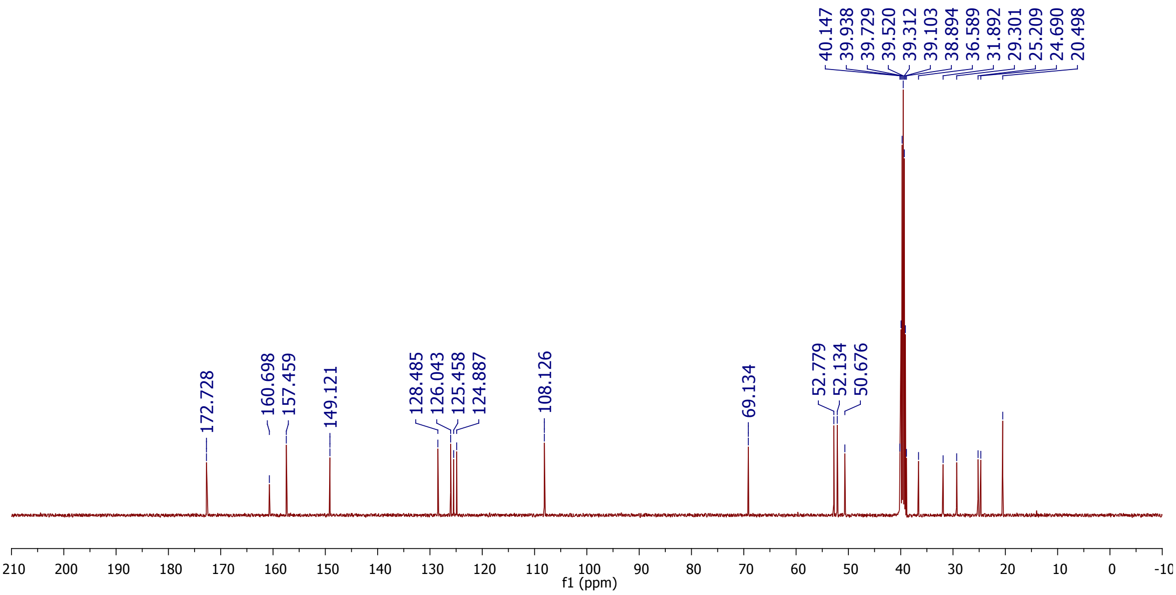

^{1}H NMR of 3t (400 MHz, DMSO-d_6)

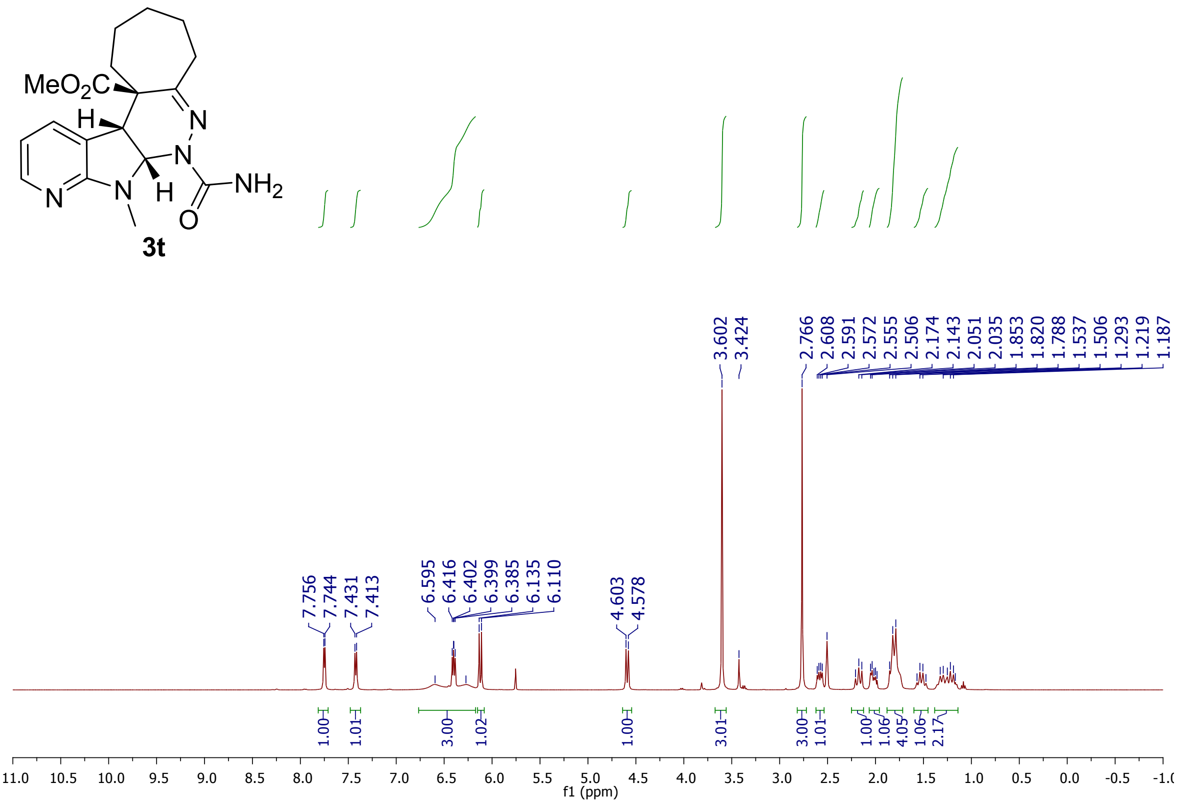

^{13}C NMR of 3t (100 MHz, DMSO-d_6)

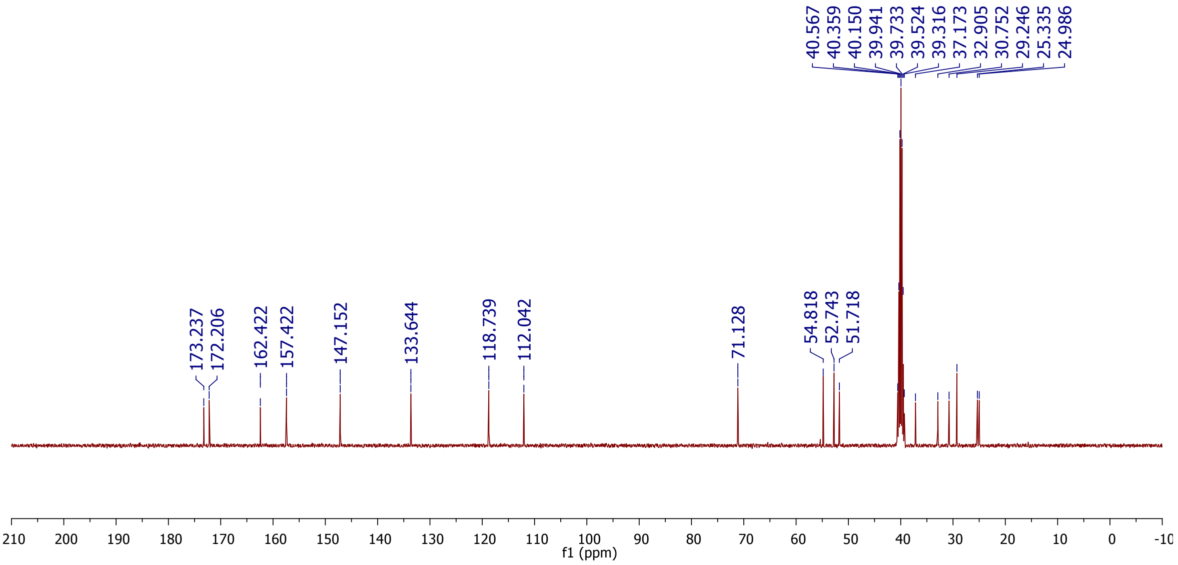

^{1}H NMR of 3u (400 MHz, DMSO-d_6)

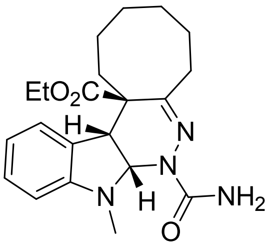

3u

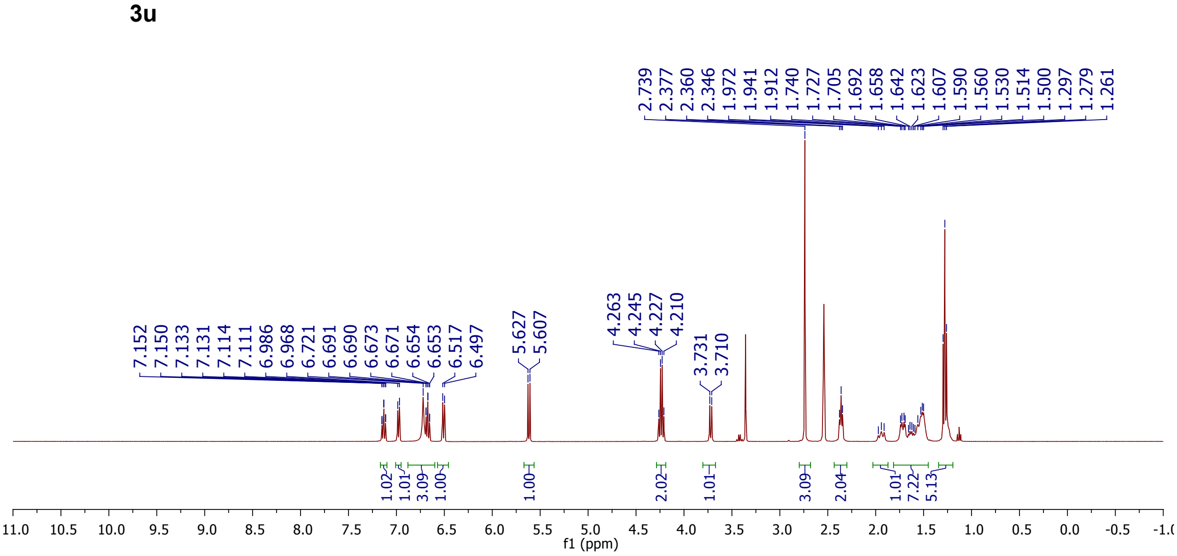

^{13}C NMR of 3u (100 MHz, DMSO-d_6)

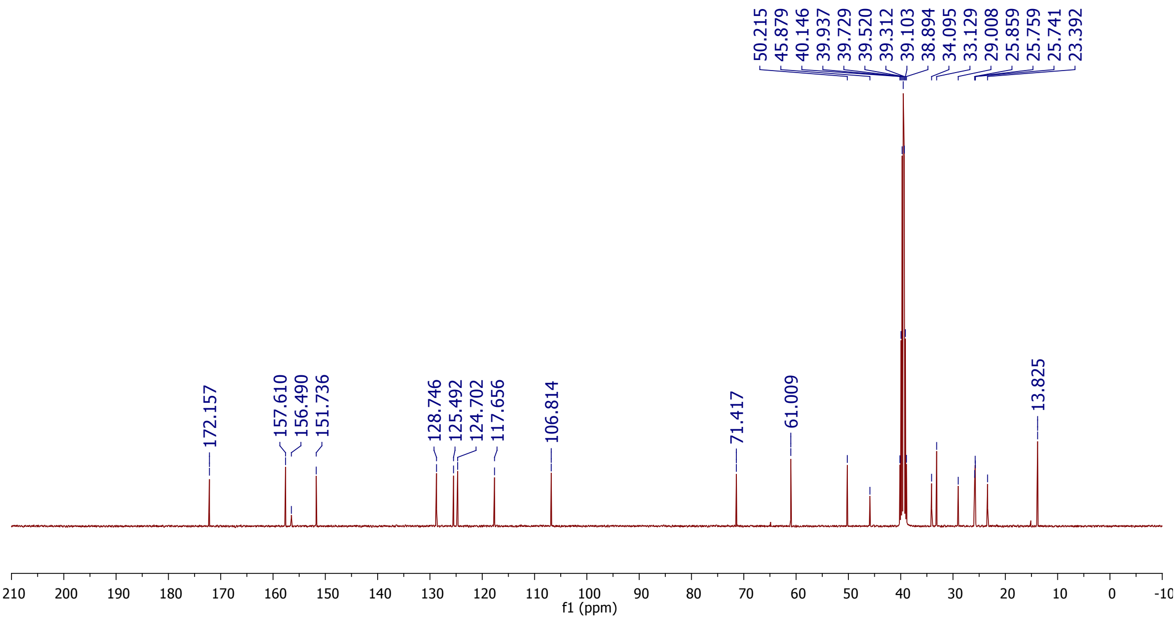

^{1}H NMR of 3v (400 MHz, DMSO-d_6)

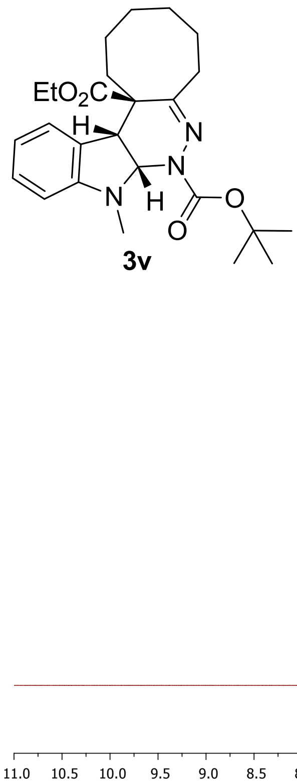

3v

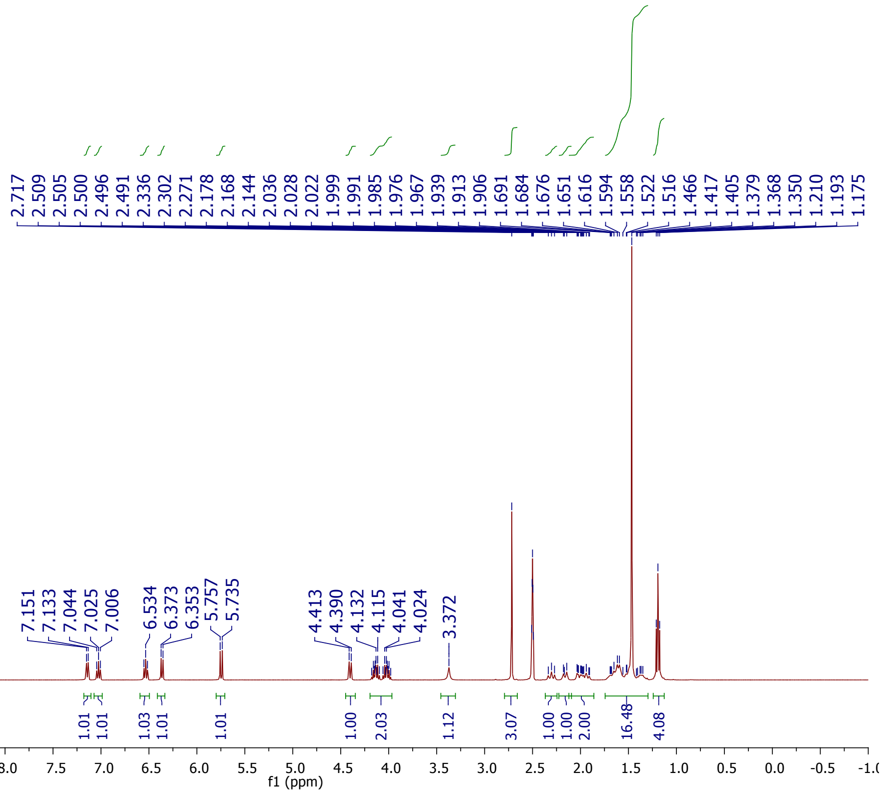

^{13}C NMR of 3v (100 MHz, DMSO-d_6)

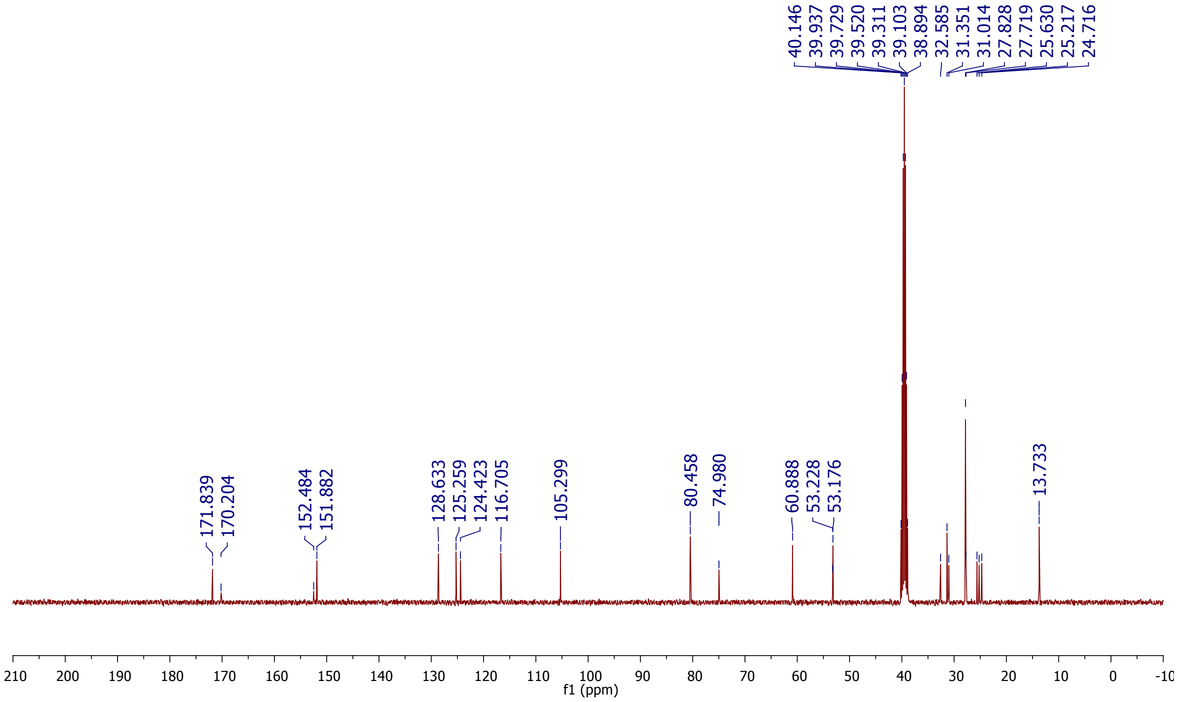

^{1}H NMR of 3w (400 MHz, DMSO-d_6)

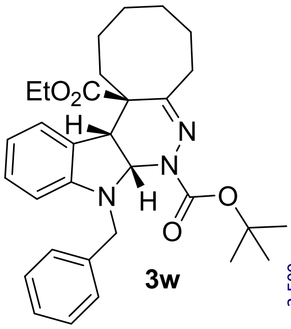

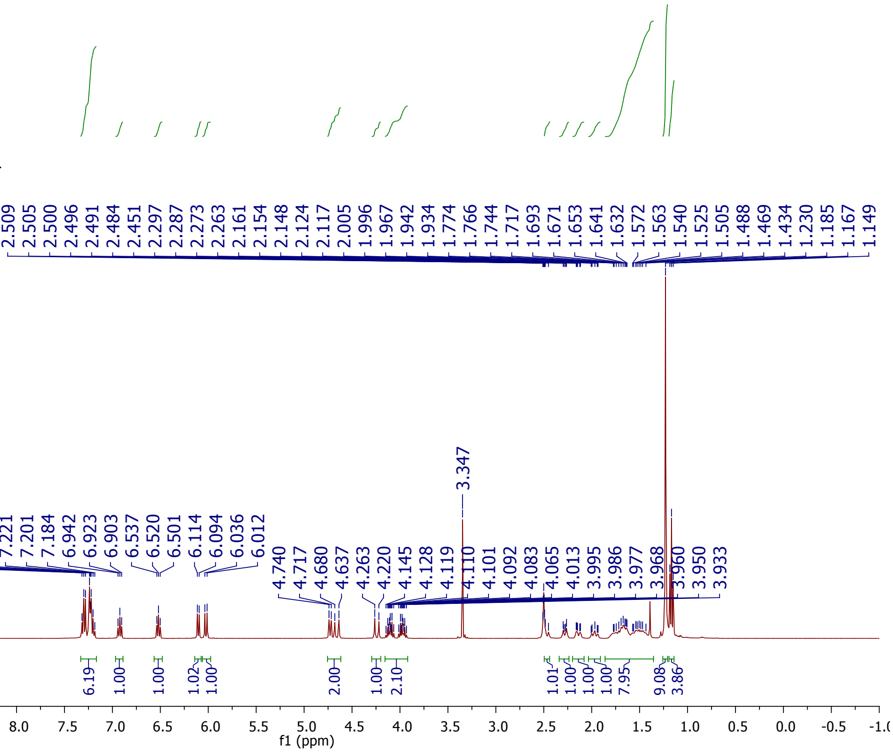

^{13}C NMR of 3w (100 MHz, DMSO-d_6)

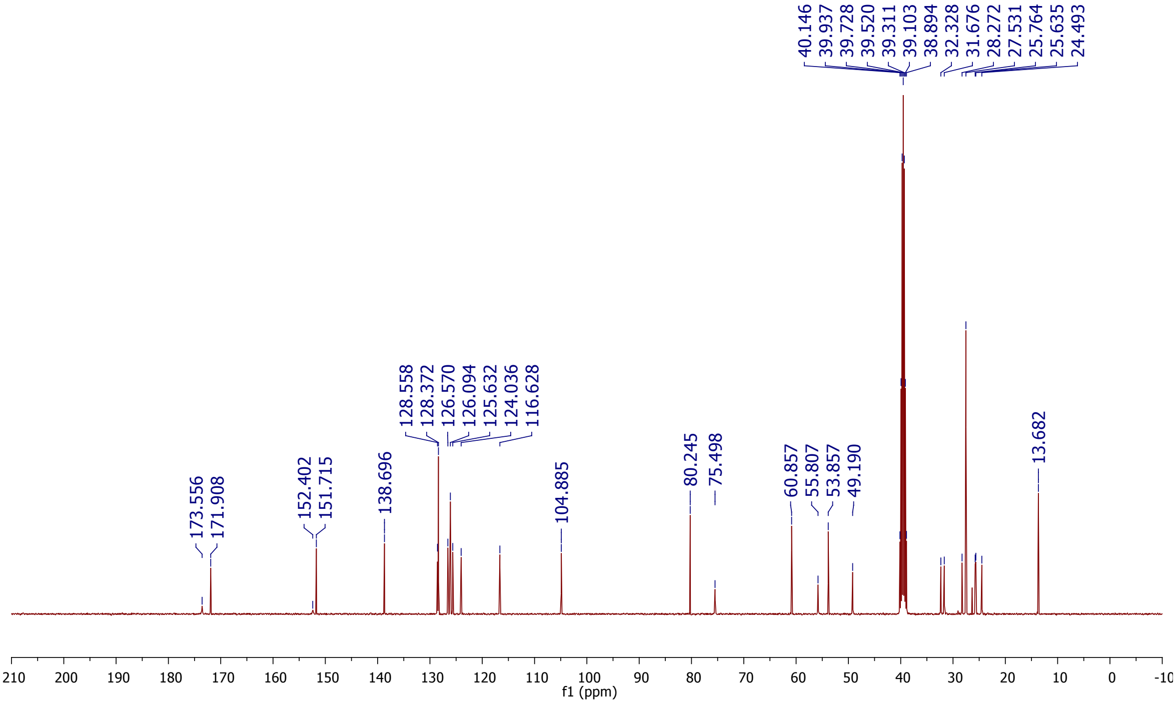

^{1}H NMR of 3x (400 MHz, DMSO-d_6)

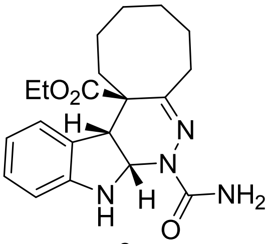

3x

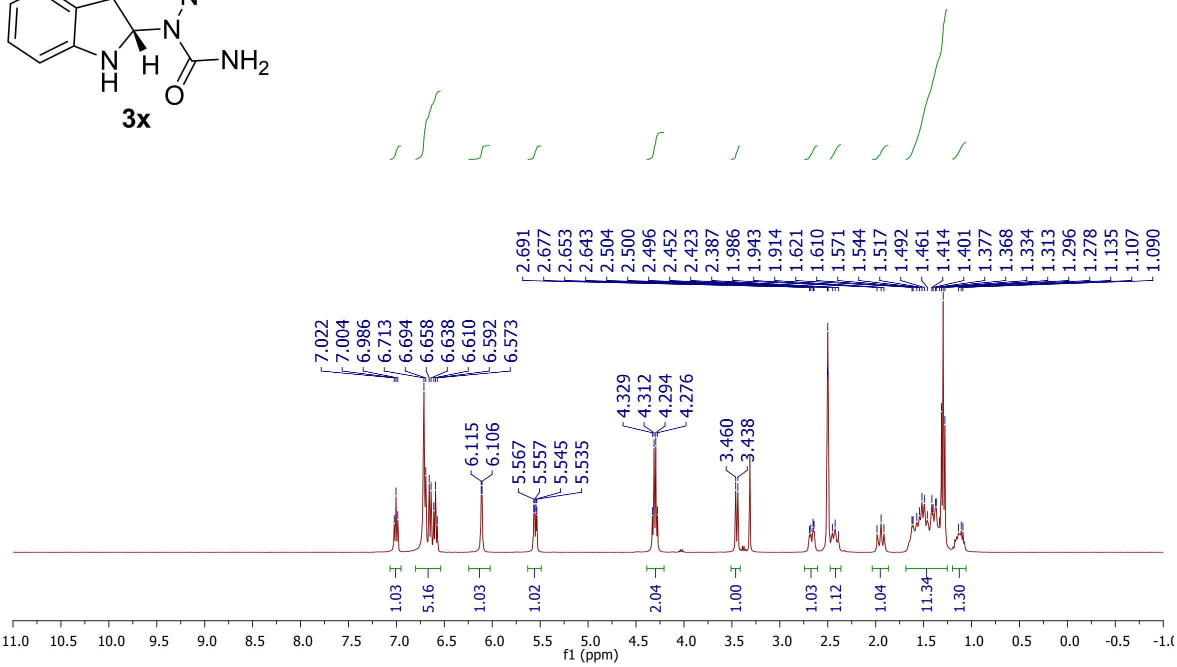

^{13}C NMR of 3x (100 MHz, DMSO-d_6)

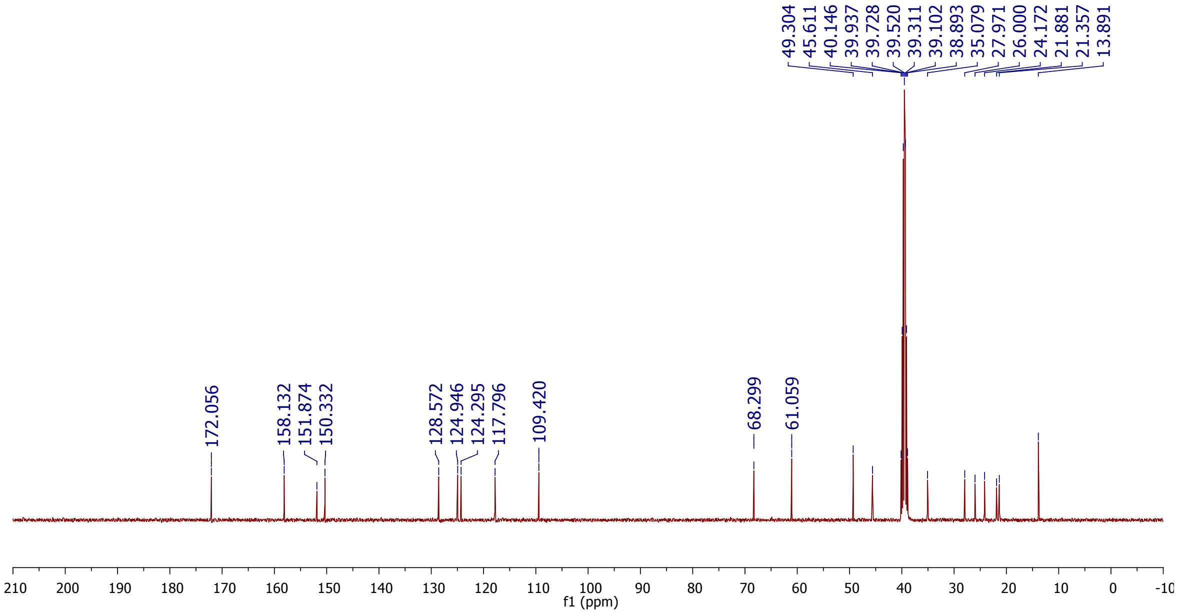

^{1}H NMR of (*cis,cis*)-3z (400 MHz, DMSO-d_6)

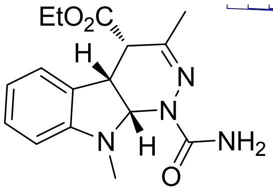

(*cis,cis*)-**3z**

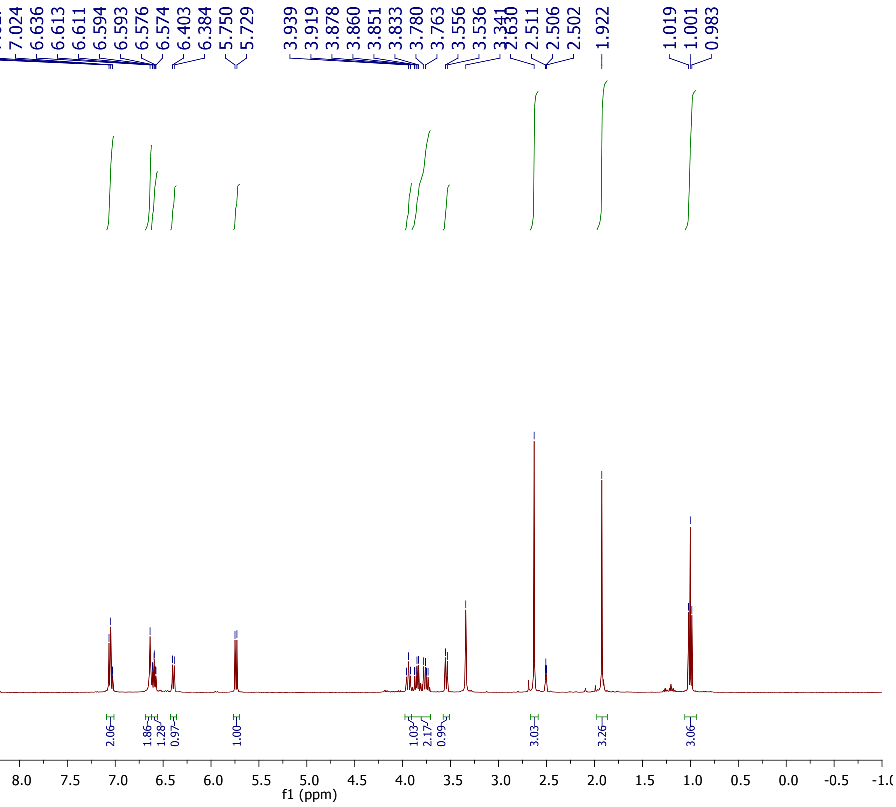

^{13}C NMR of (*cis,cis*)-3z (100 MHz, DMSO-d_6)

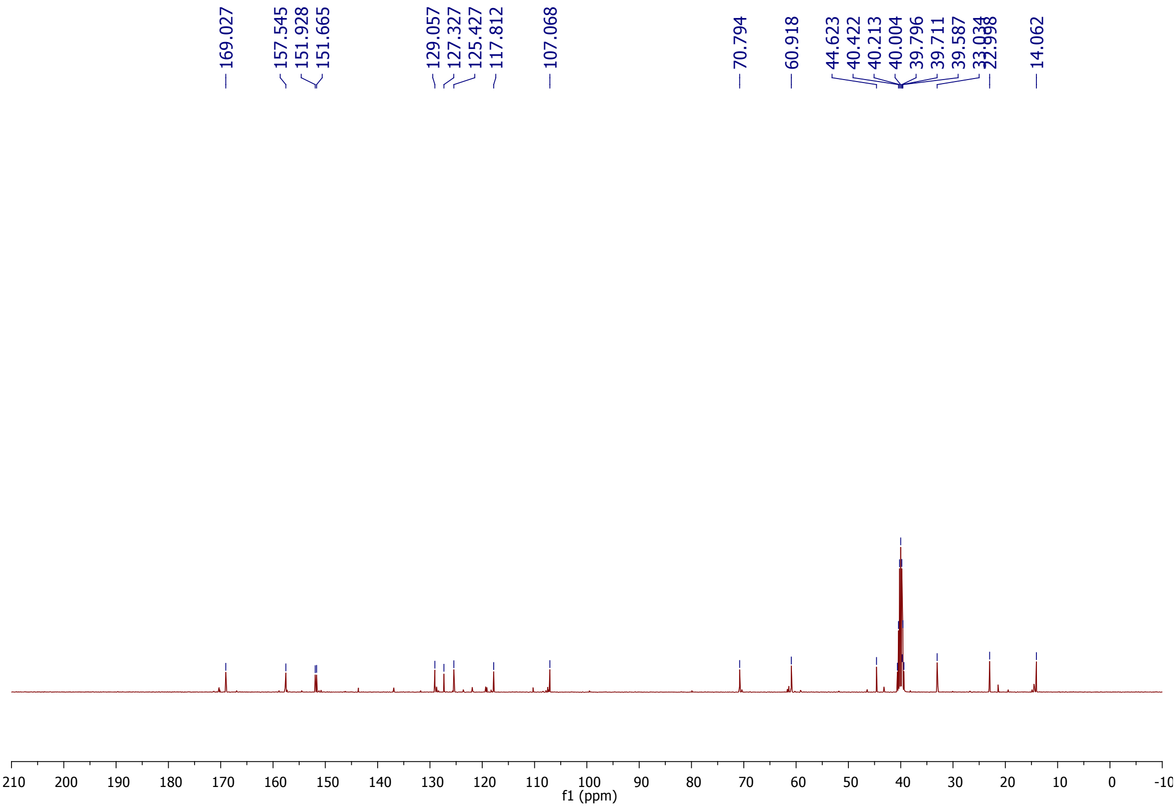

NOESY NMR of (*cis*,*cis*)-3z (400 MHz, DMSO-d_6)

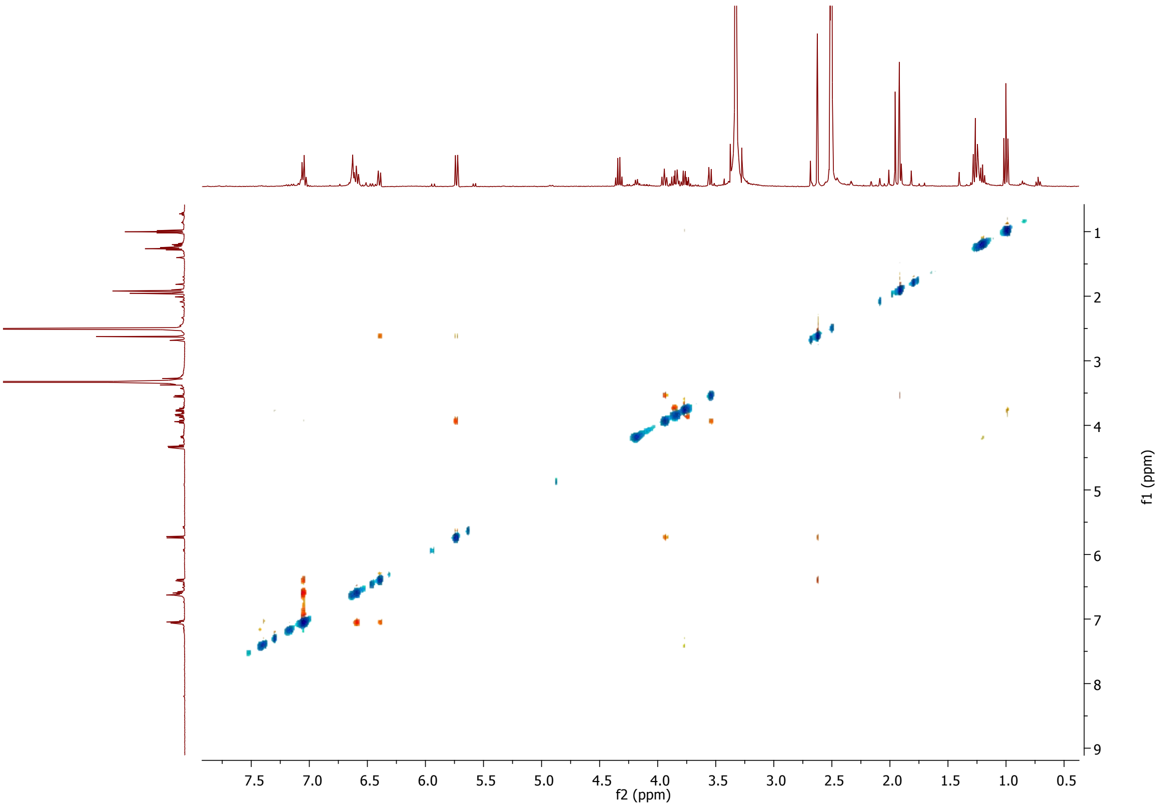

^{1}H NMR of (*cis*,*trans*)-3z (400 MHz, DMSO-d_6)

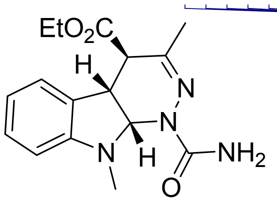

(*cis*,*trans*)-**3z**

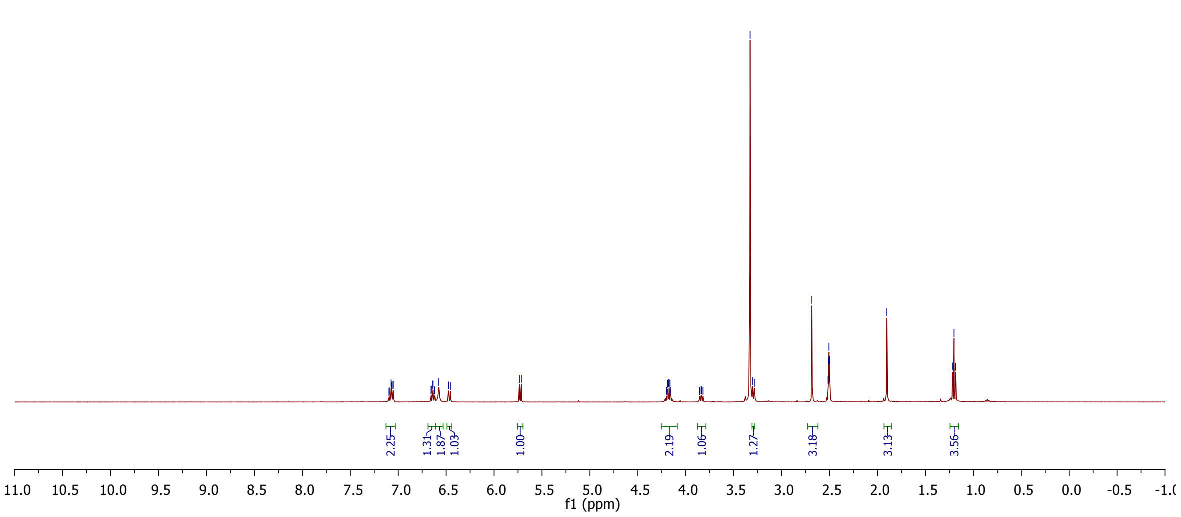

^{13}C NMR of (*cis*,*trans*)-3z (100 MHz, DMSO-d_6)

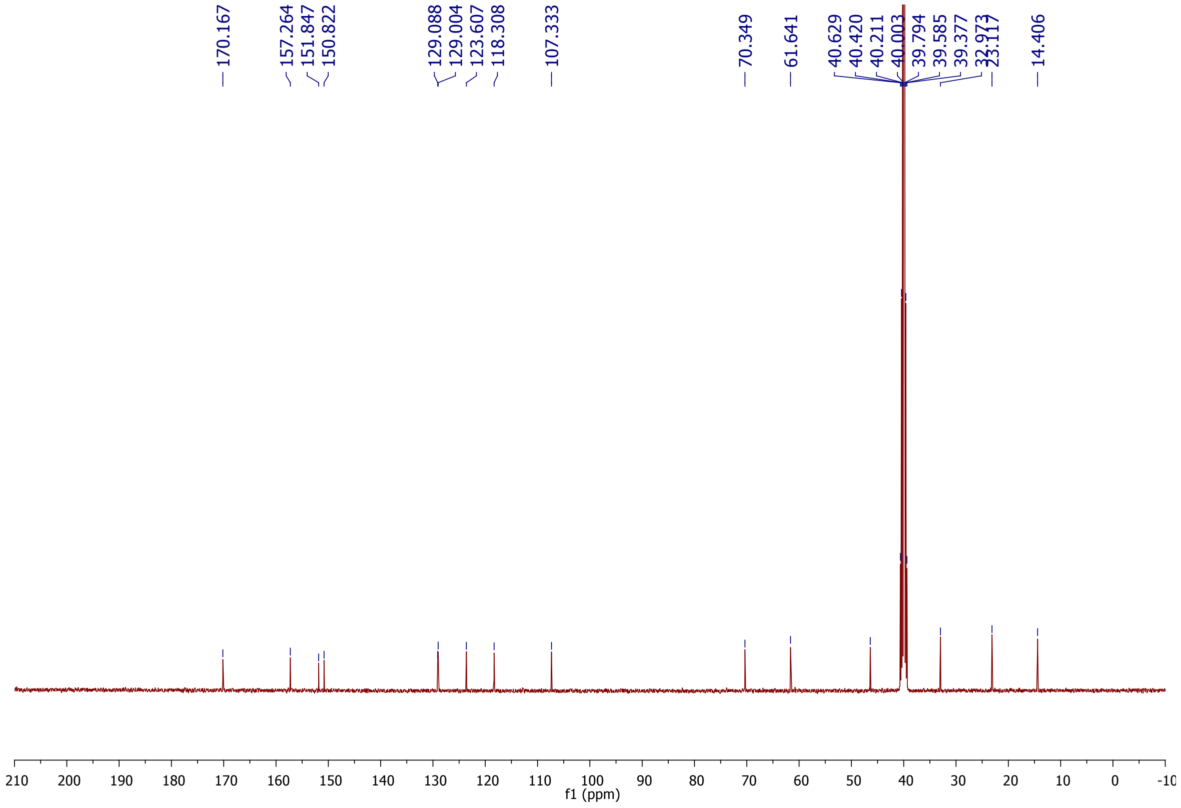

NOESY NMR of (*cis*,*trans*)-3z (400 MHz, DMSO-d_6)

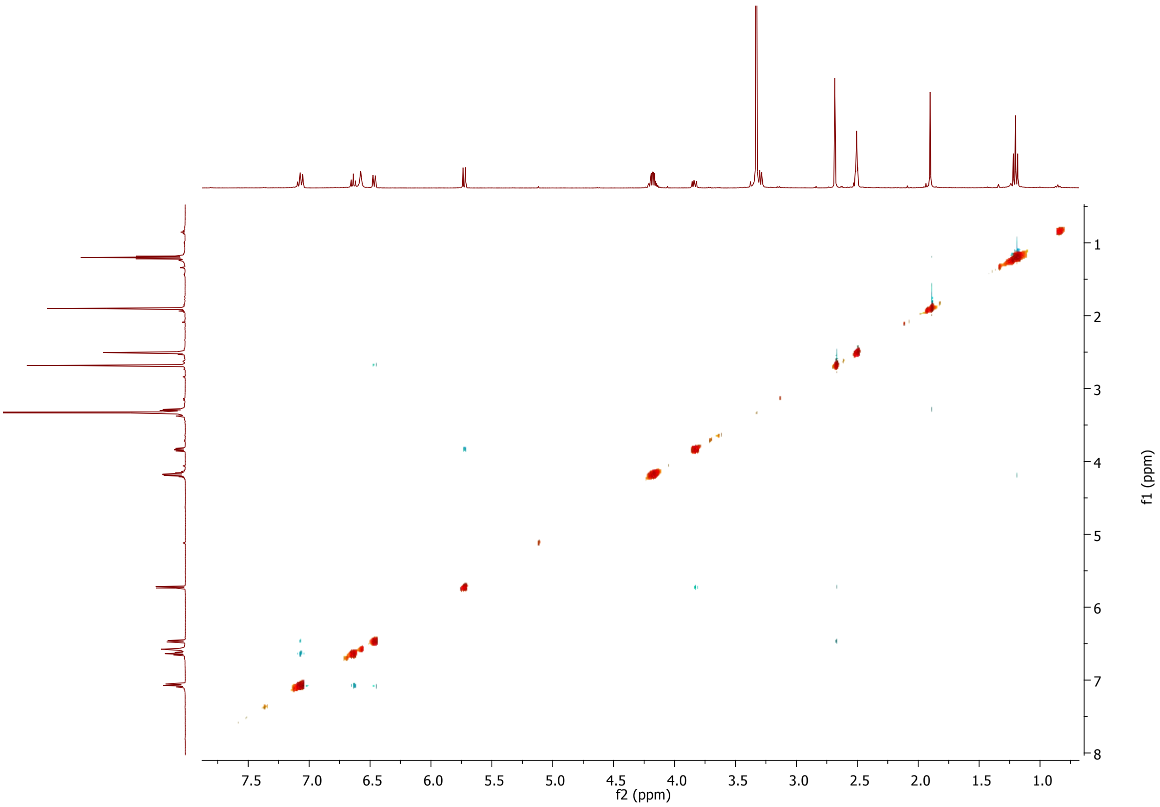

^{1}H NMR of (*cis*,*cis*)-3ab (400 MHz, DMSO-d_6)

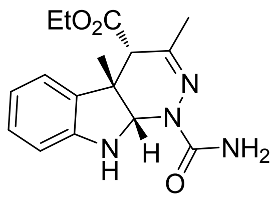

(*cis*,*cis*)-**3ab**

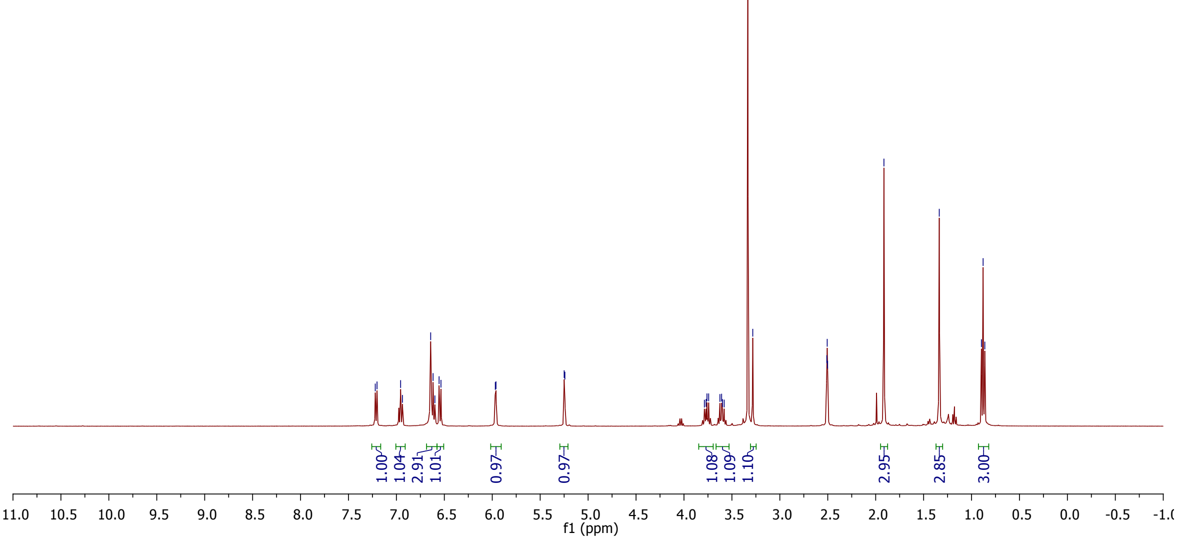

^{13}C NMR of (*cis*,*cis*)-3ab (100 MHz, DMSO-d_6)

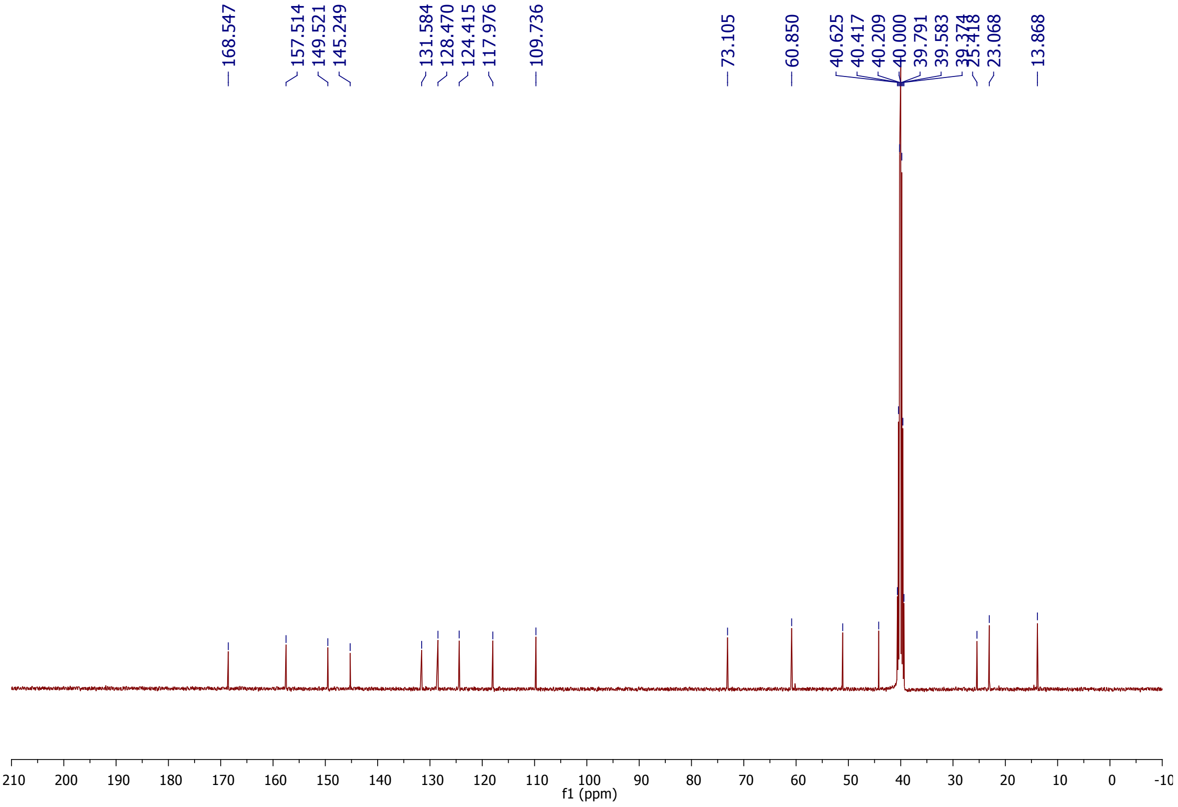

NOESY NMR of (*cis*,*cis*)-3ab (400 MHz, DMSO-d_6)

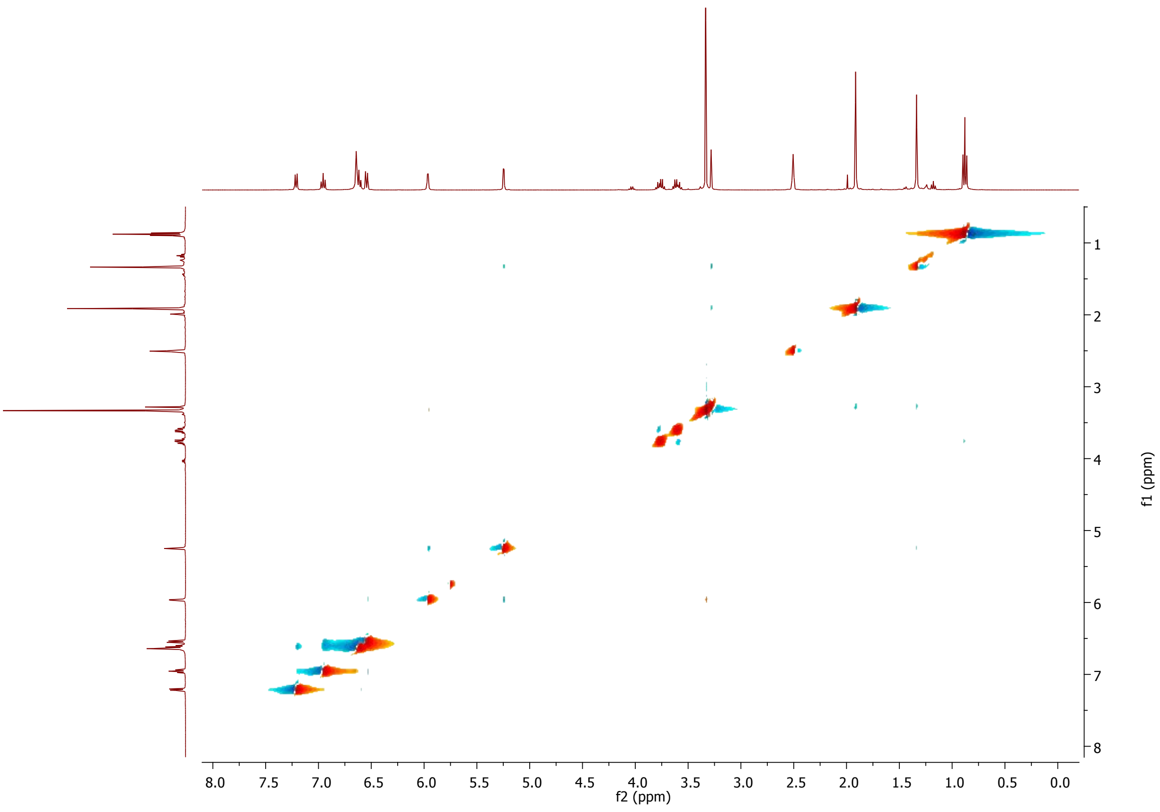

^{1}H NMR of 3ad (400 MHz, DMSO-d_6)

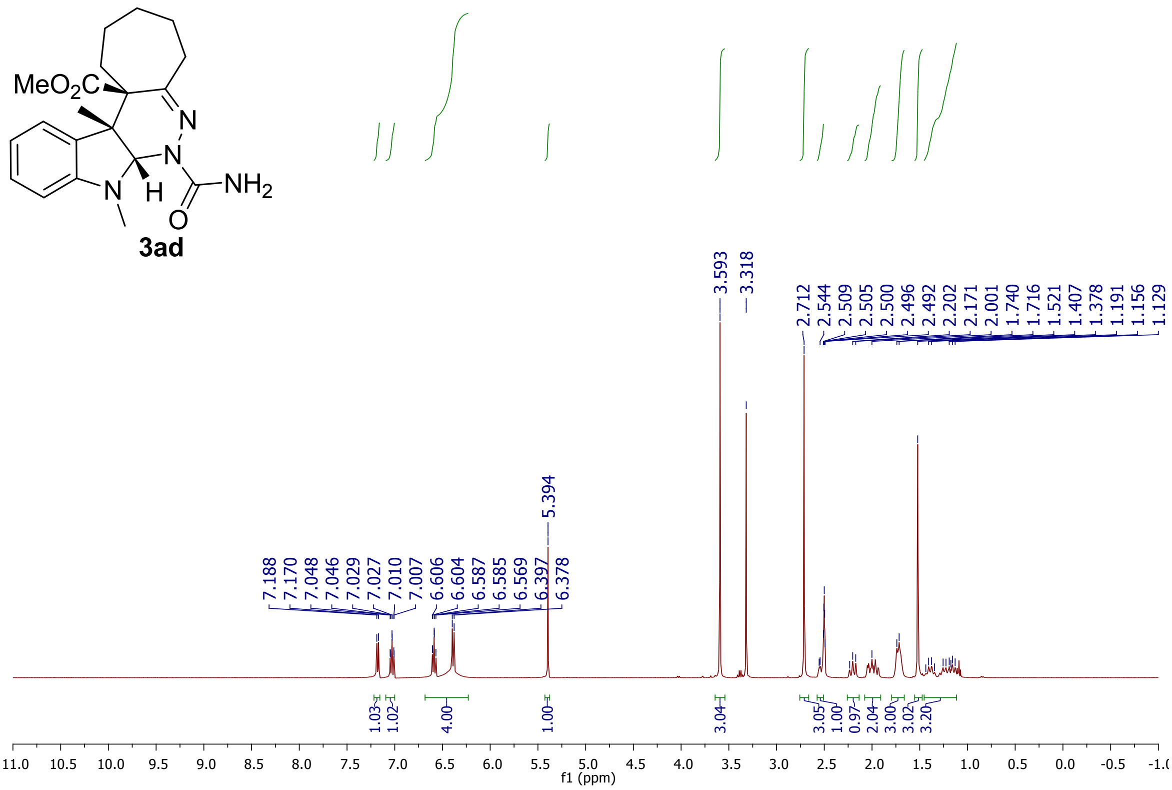

^{13}C NMR of 3ad (100 MHz, DMSO-d_6)

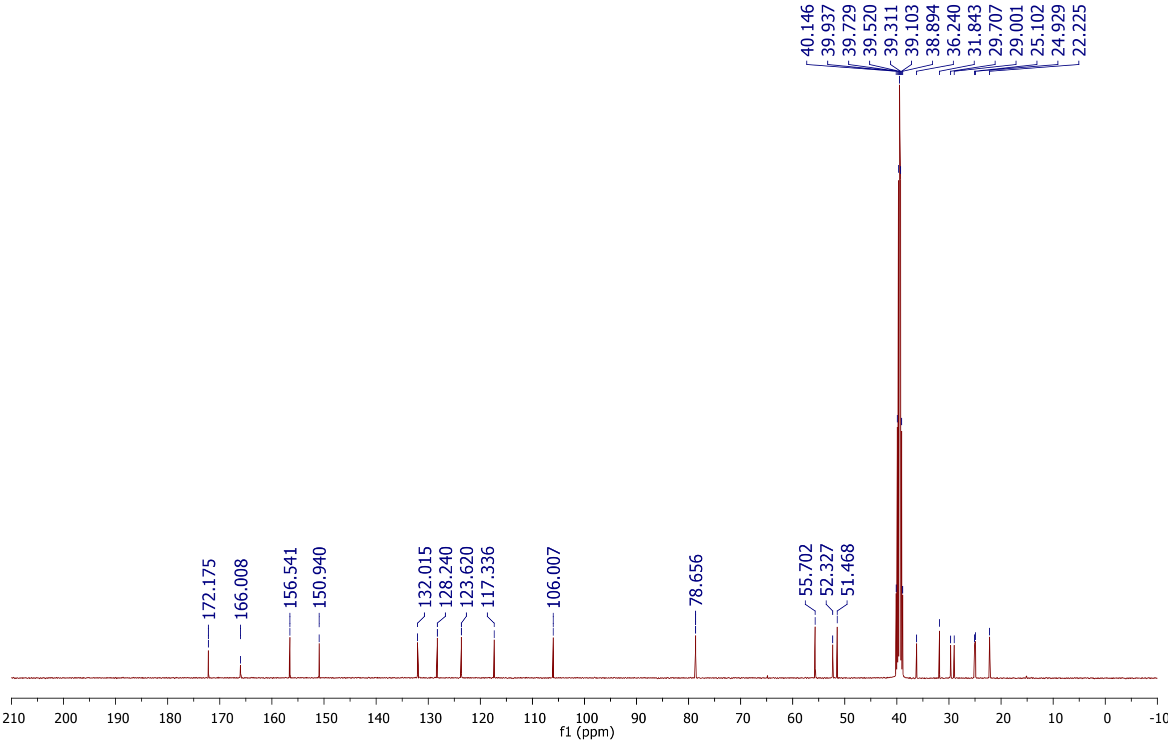

^{1}H NMR of 3ae (400 MHz, DMSO-d_6)

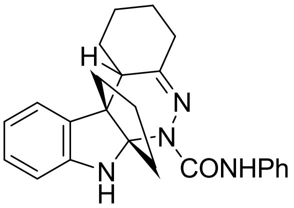

3ae

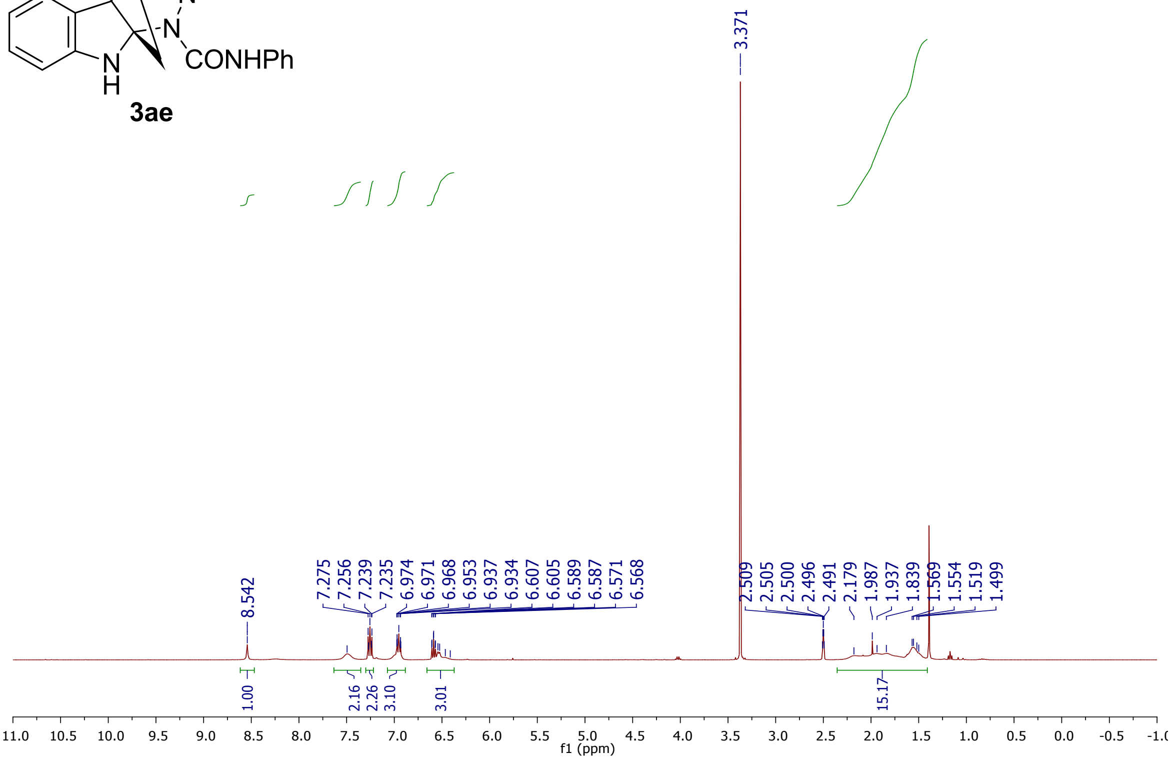

^{13}C NMR of 3ae (100 MHz, DMSO-d_6)

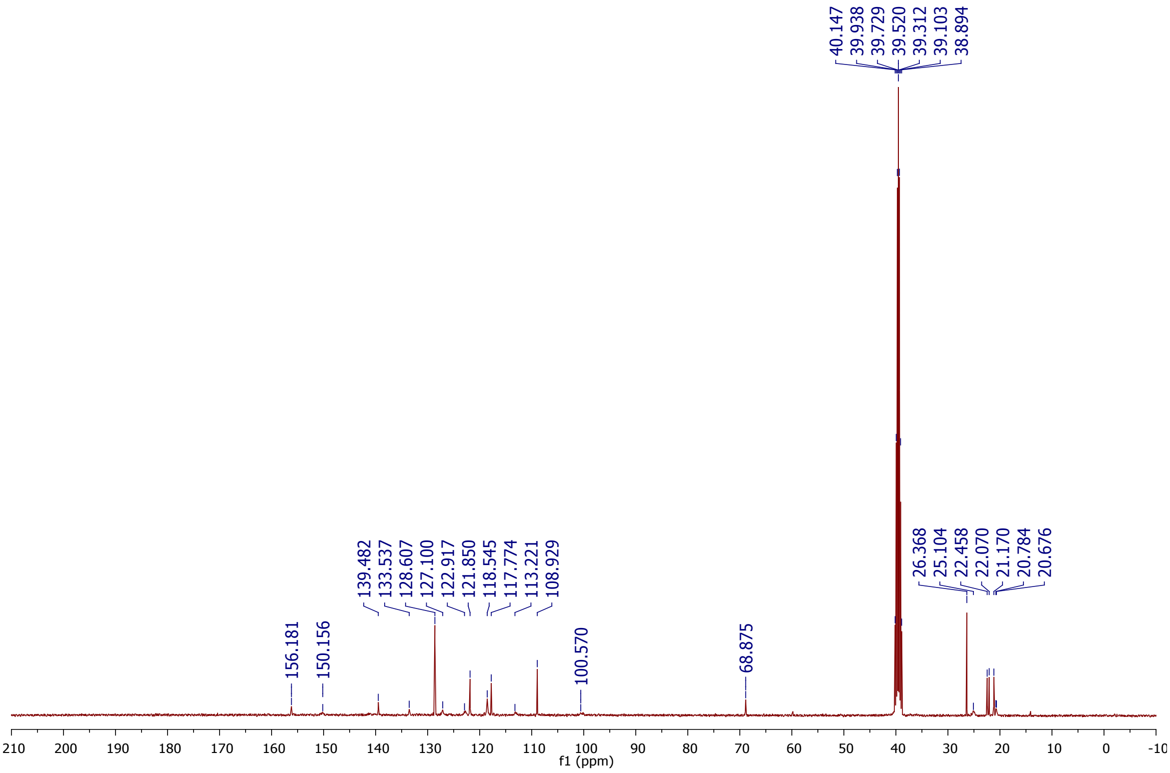

^{1}H NMR of 3af (400 MHz, $CDCl_3$)

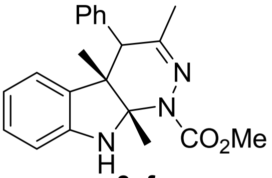

3af

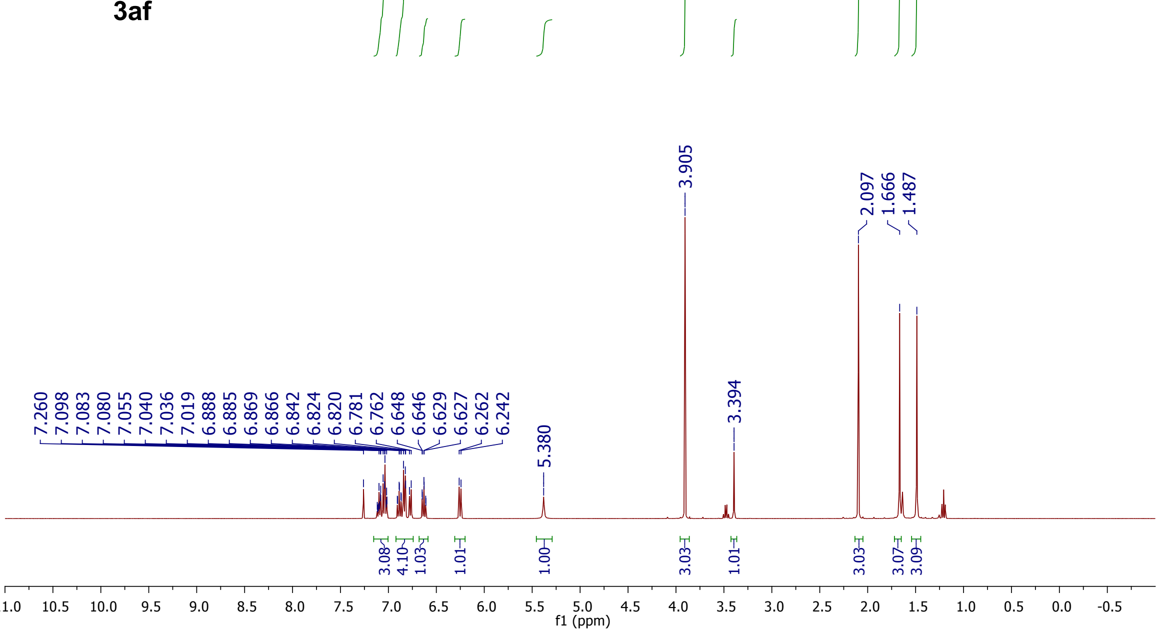

^{13}C NMR of 3af (100 MHz, $CDCl_3$)

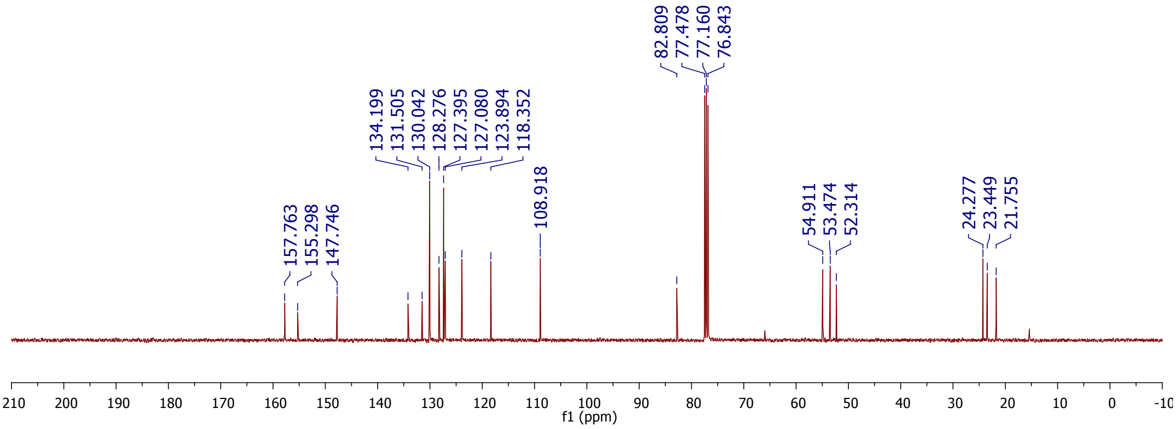

^{1}H NMR of 5a (400 MHz, DMSO-d_6)

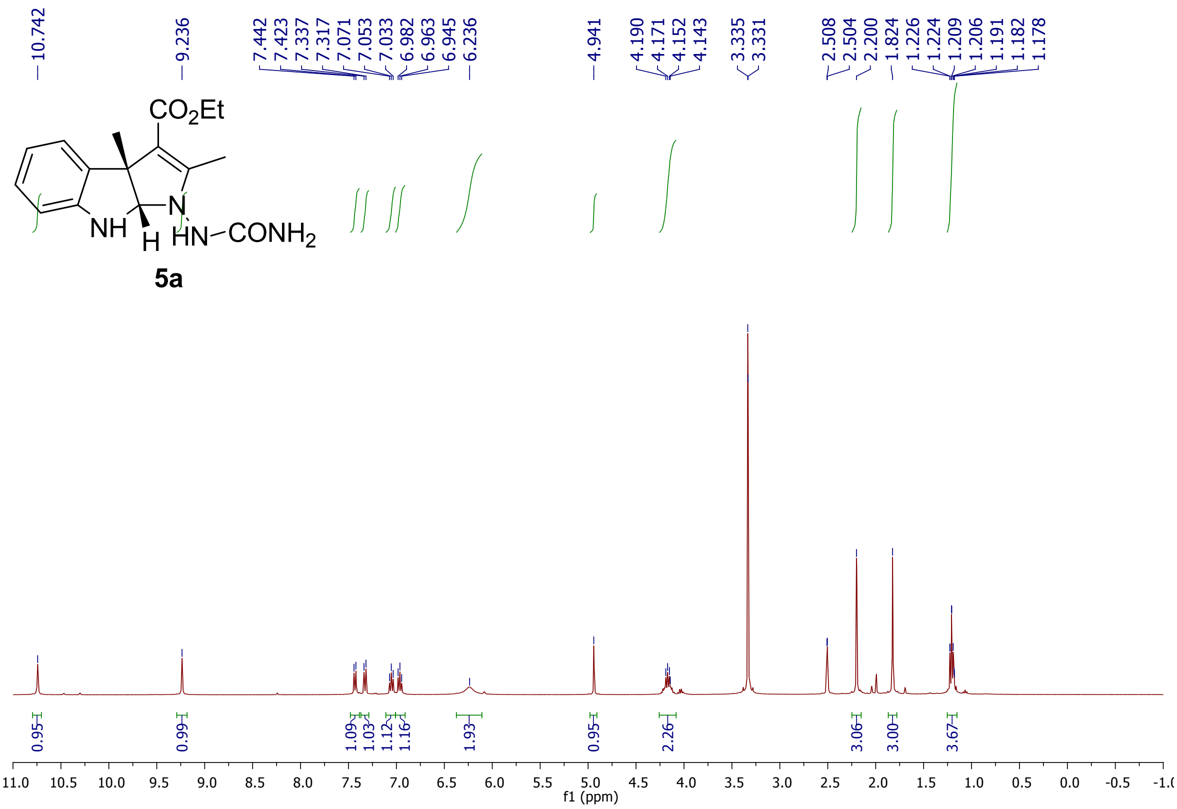

^{13}C NMR of 5a (100 MHz, DMSO-d_6)

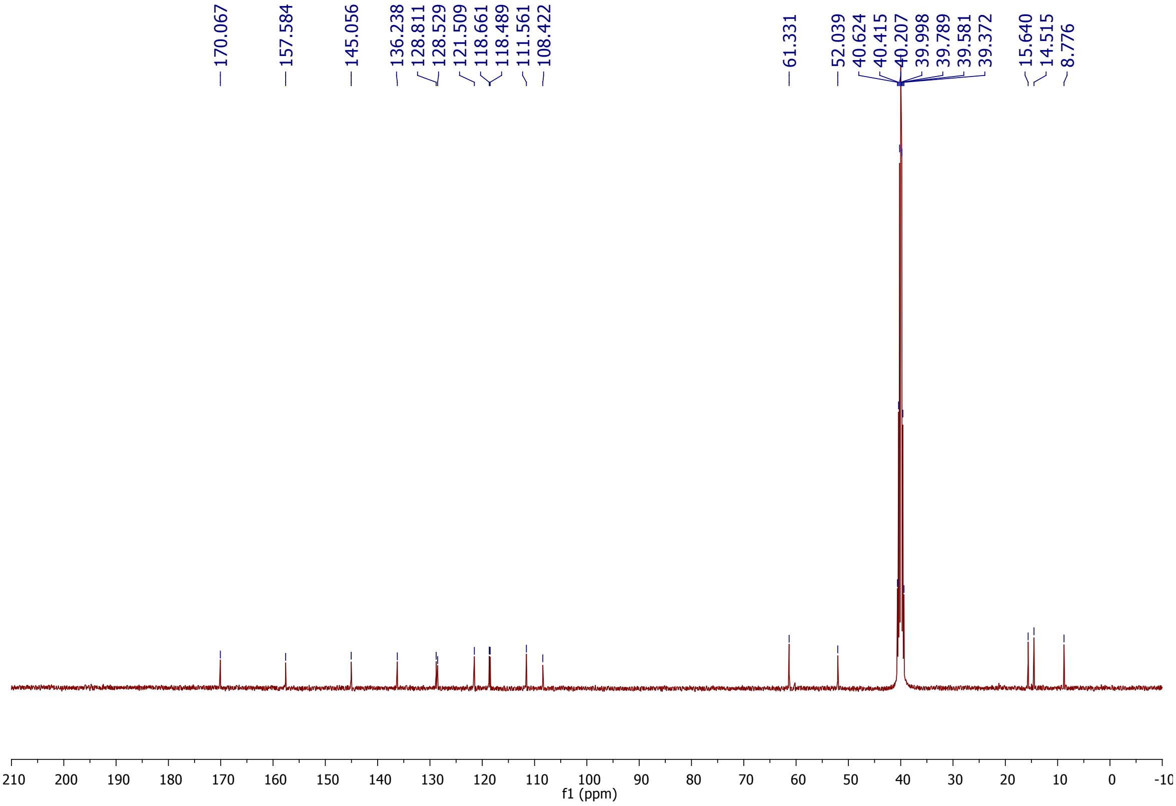

^{1}H NMR of 5b (400 MHz, $CDCl_3$)

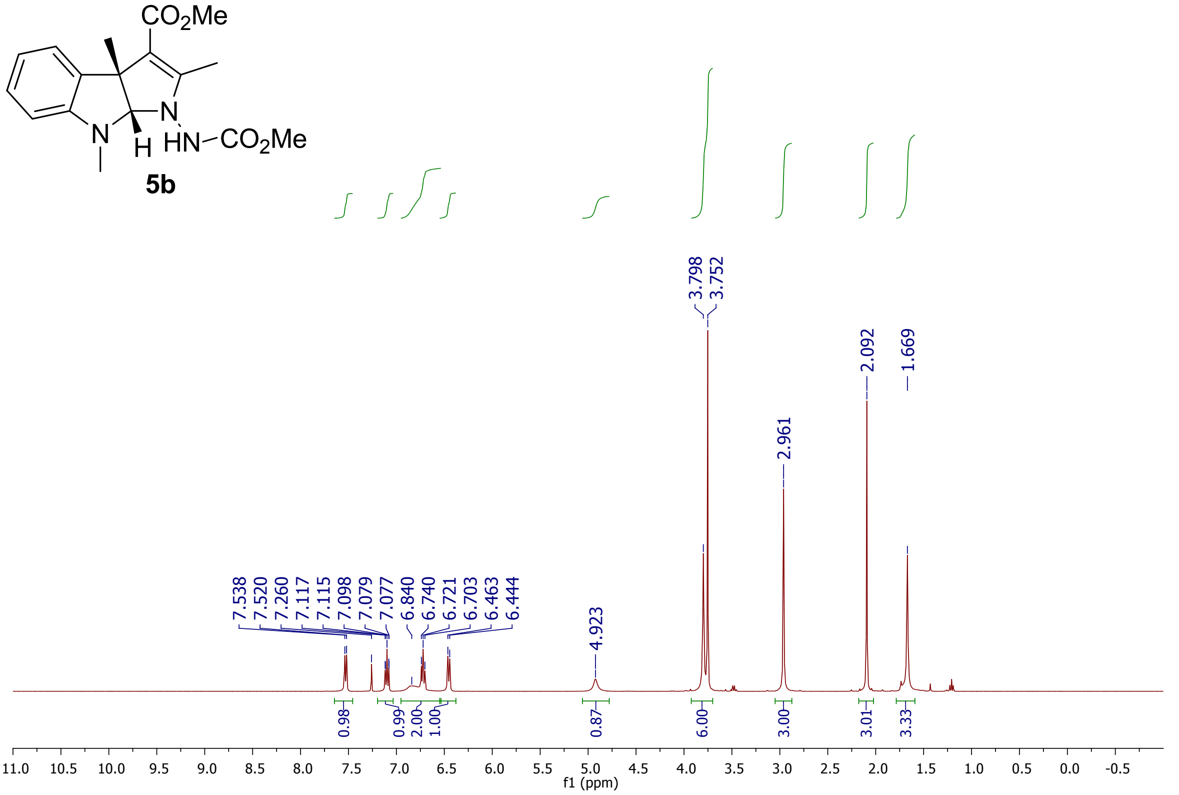

^{13}C NMR of 5b (100 MHz, $CDCl_3$)

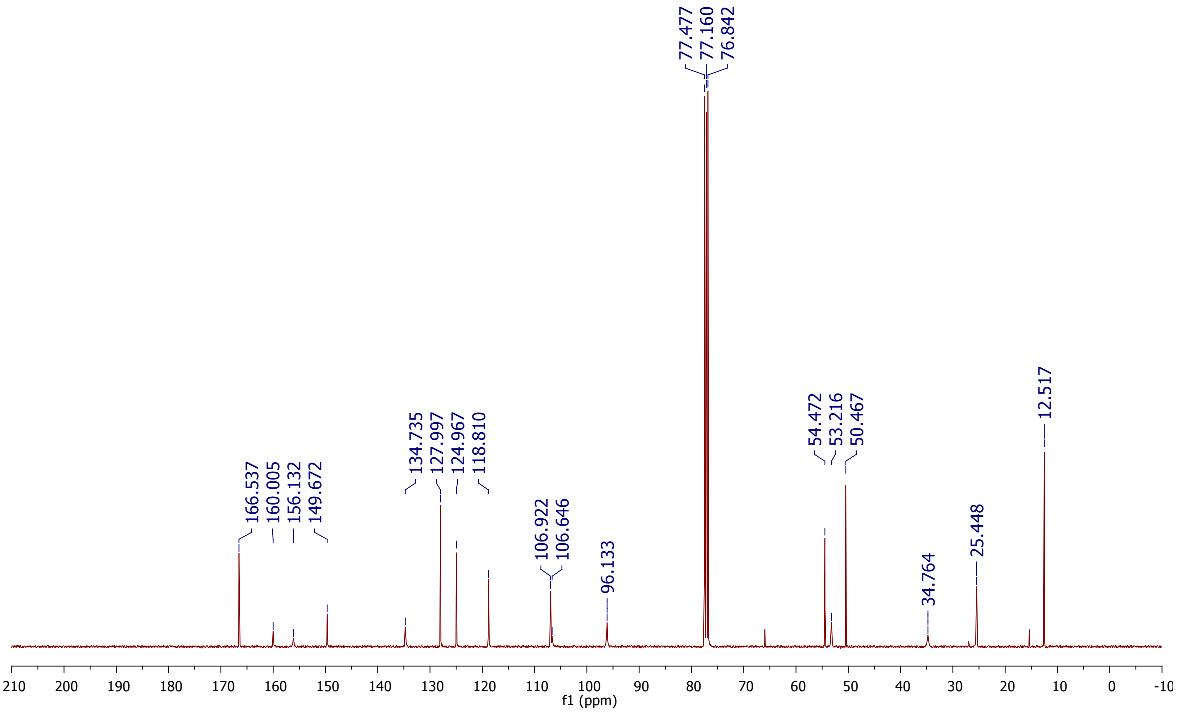

^{1}H NMR of 5c (400 MHz, DMSO-d_6)

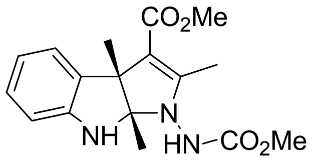

5c

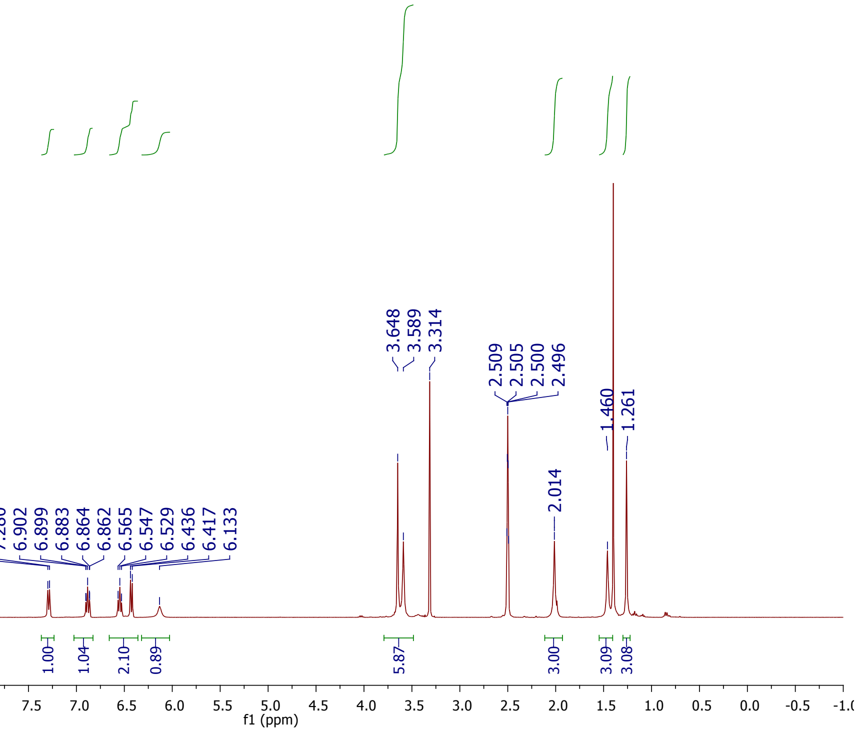

^{13}C NMR of 5c (100 MHz, DMSO-d_6)

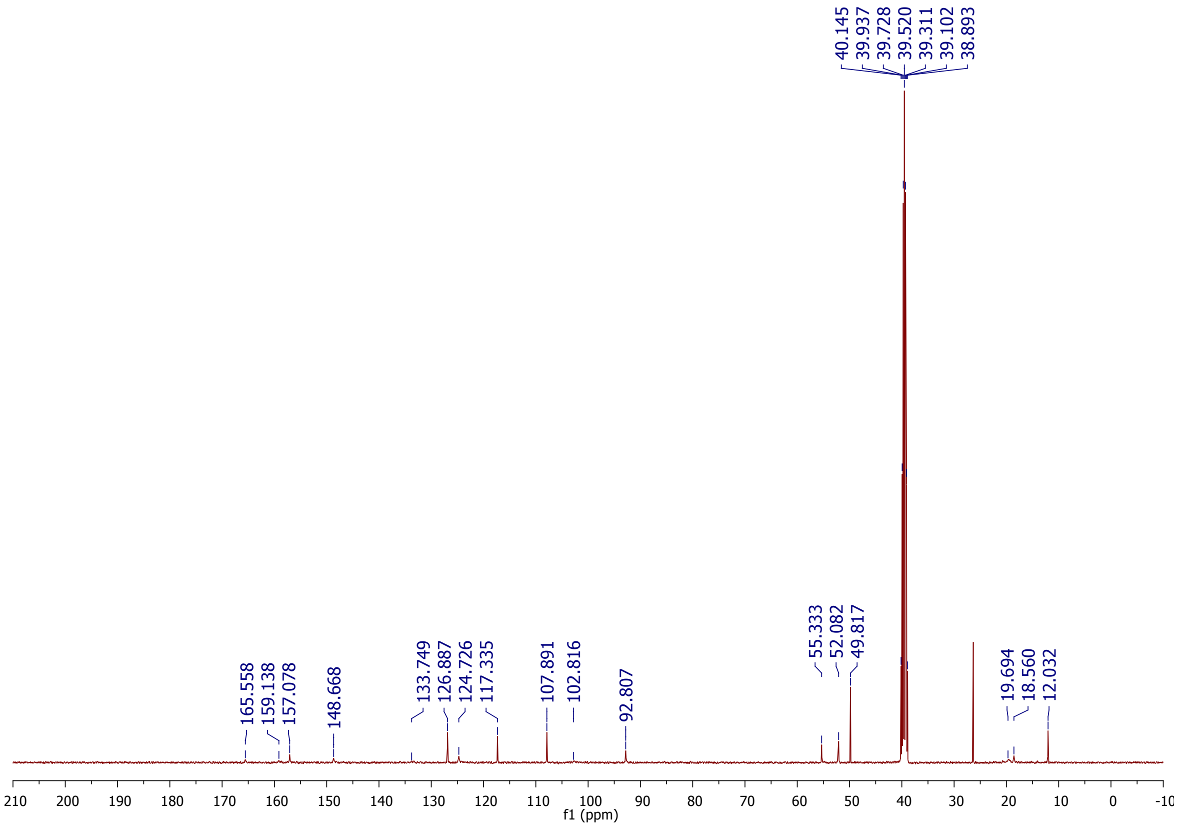

HMQC NMR of 5c (400 MHz, DMSO-d_6)

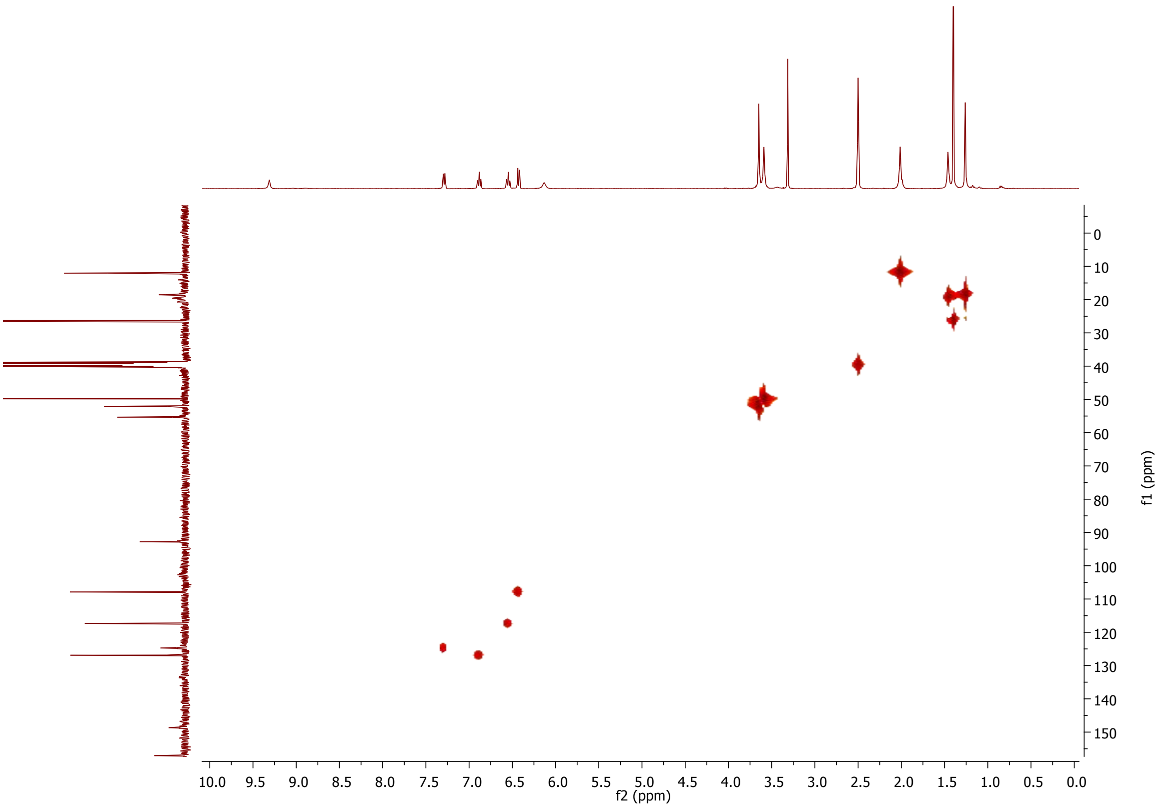

HMBC NMR of 5c (400 MHz, DMSO-d_6)

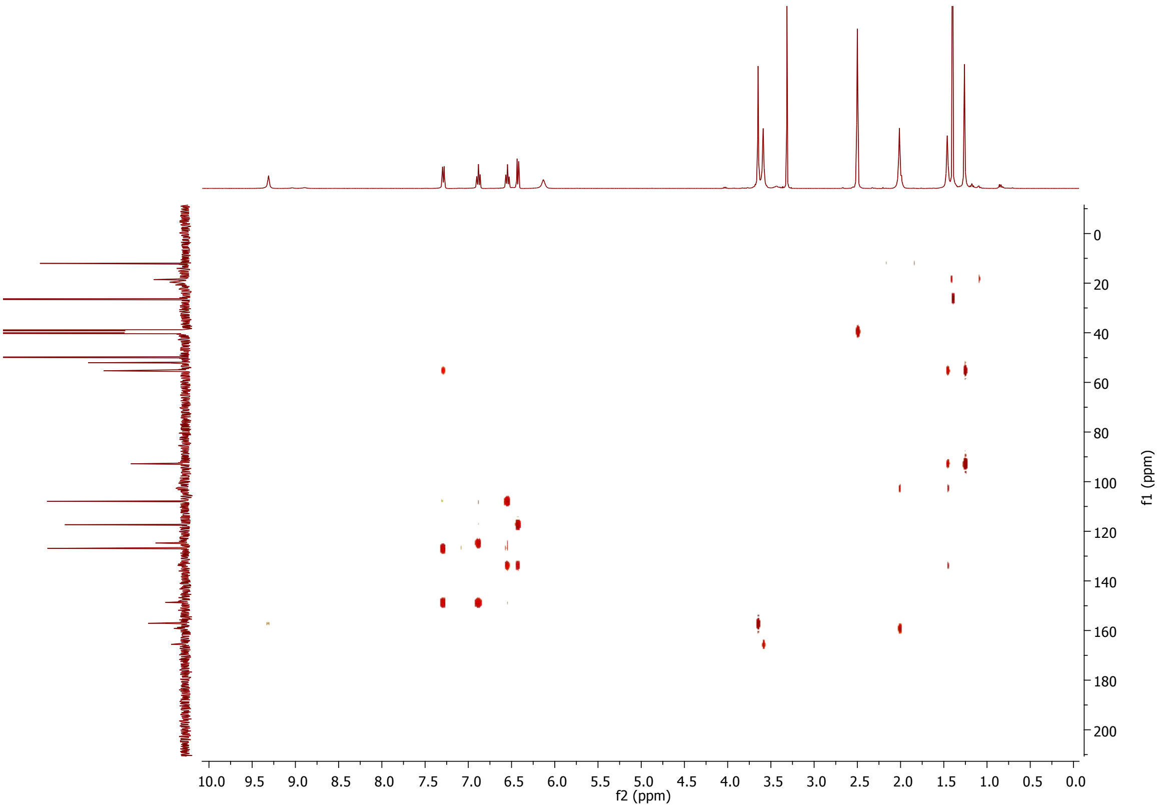

^{1}H NMR of 5d (400 MHz, DMSO-d_6)

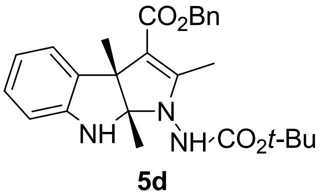

5d

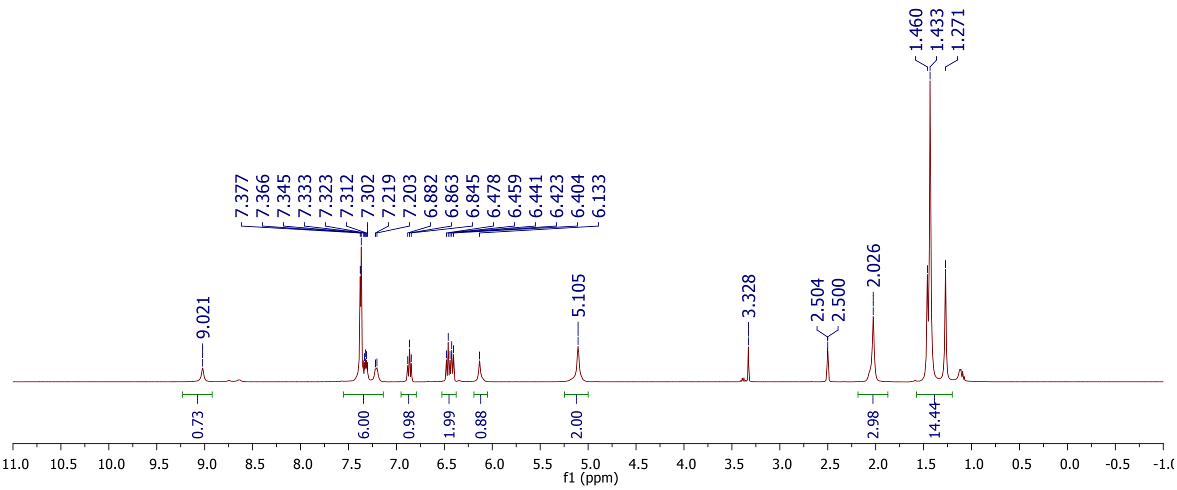

^{13}C NMR of 5d (100 MHz, DMSO-d_6)

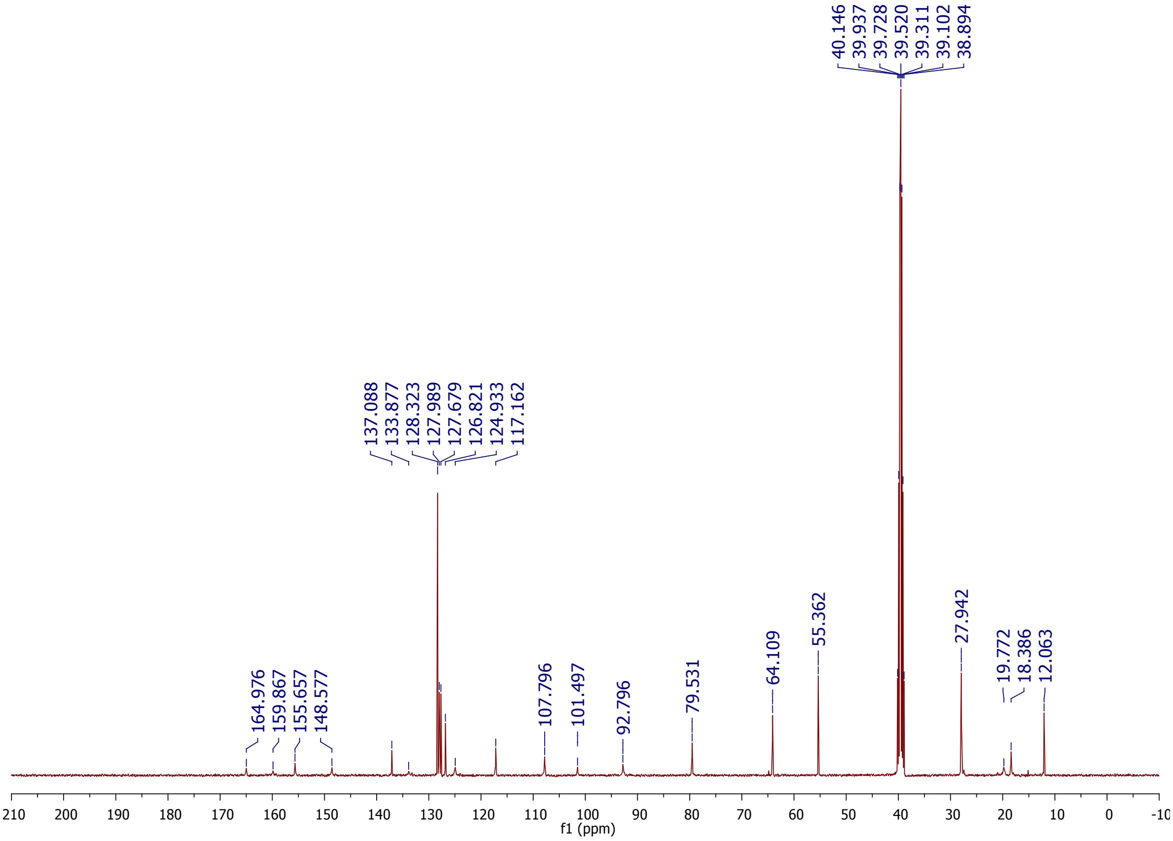

^{1}H NMR of 5e (400 MHz, DMSO-d_6)

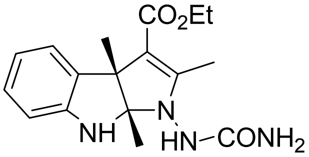

5e

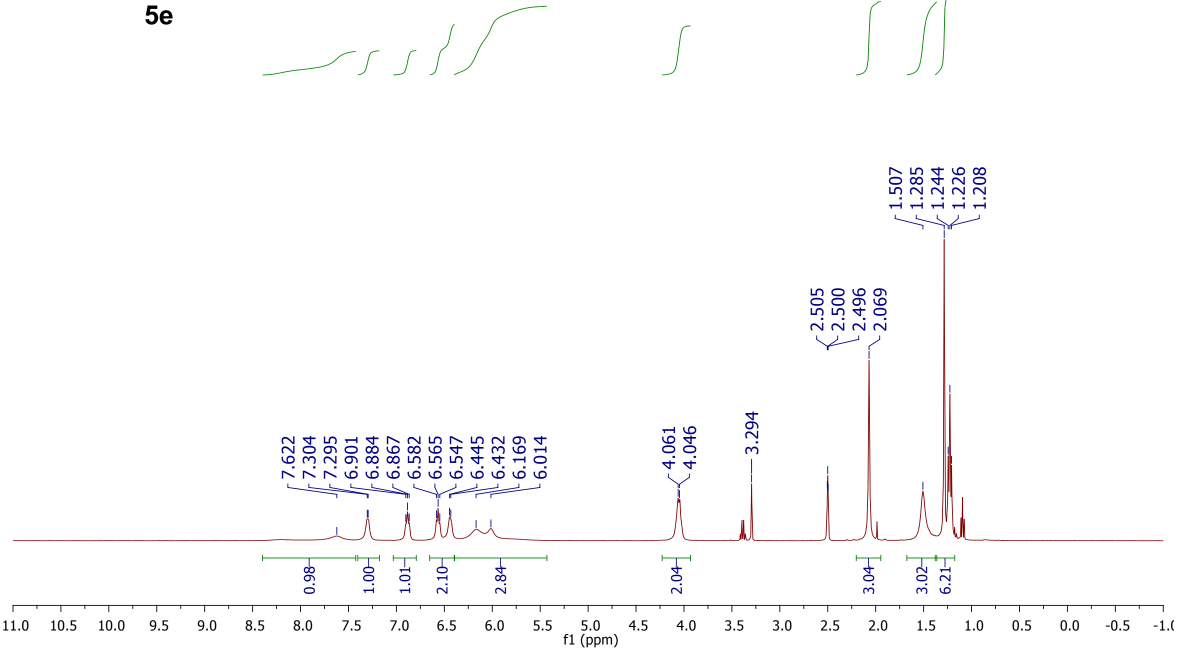

^{13}C NMR of 5e (100 MHz, DMSO-d_6)

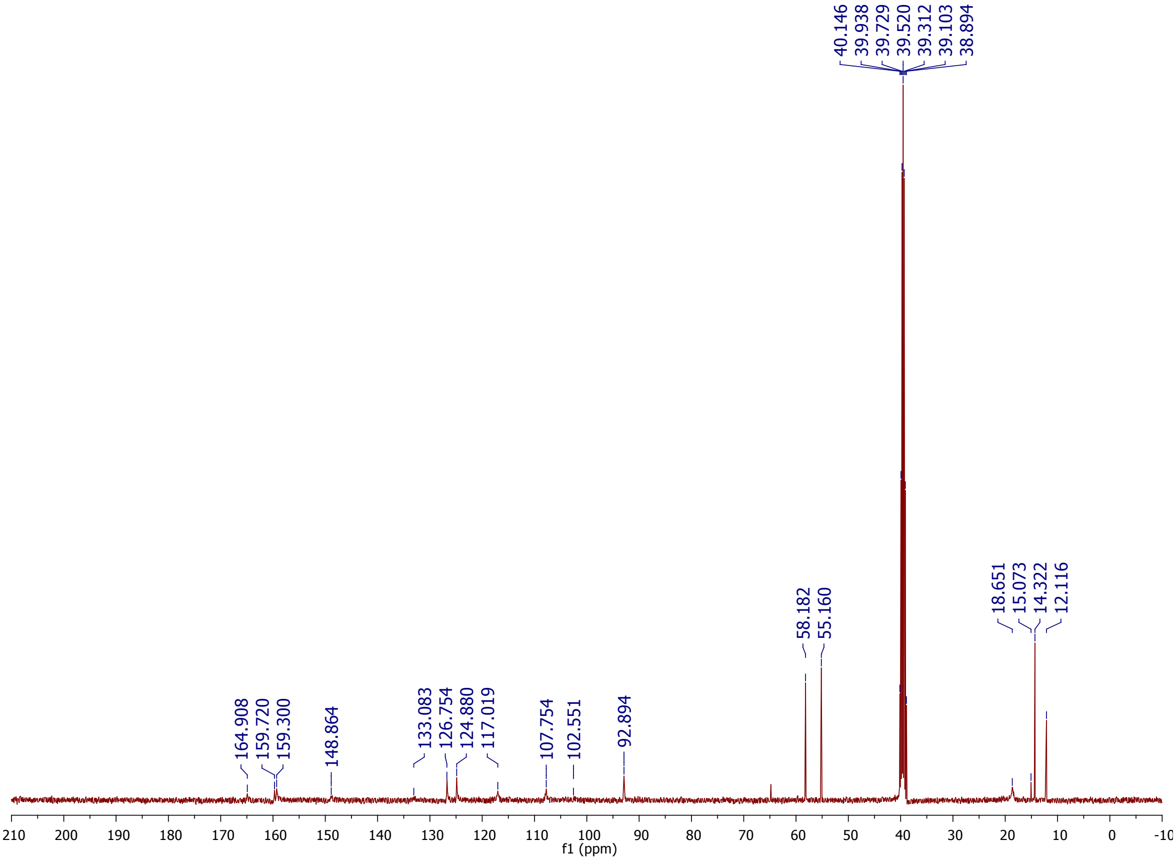

^{1}H NMR of 5f (400 MHz, DMSO-*d*$_6$)

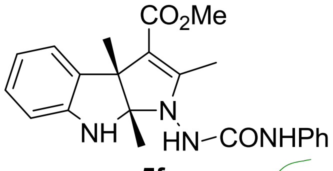

5f

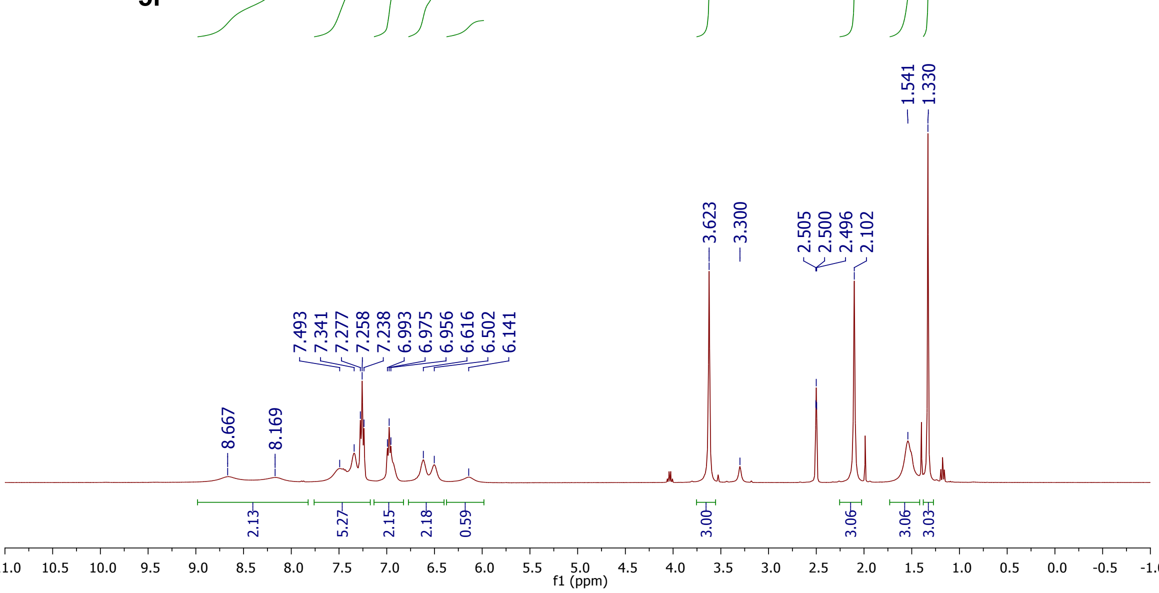

^{13}C NMR of 5f (100 MHz, DMSO-*d*$_6$)

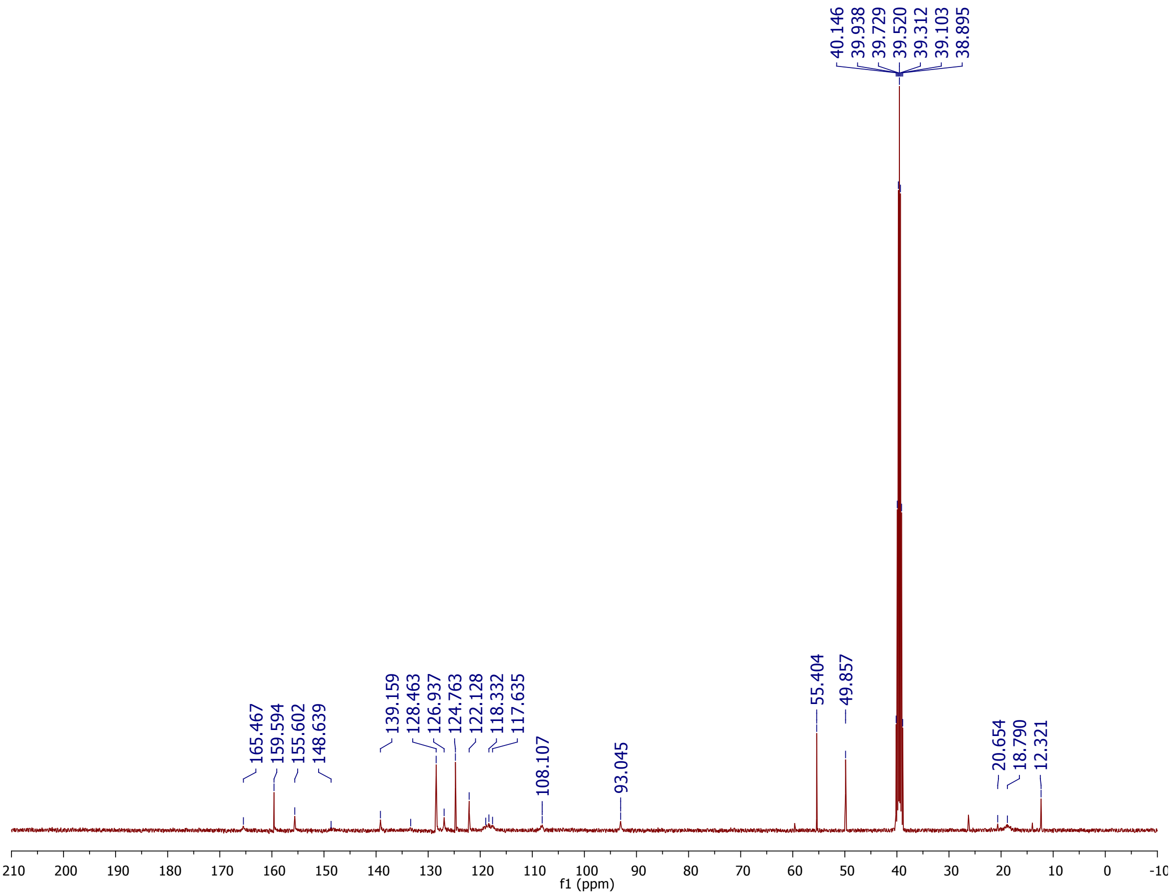

^{1}H NMR of 5g (400 MHz, CDCl$_3$)

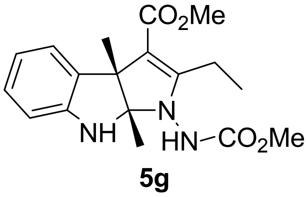

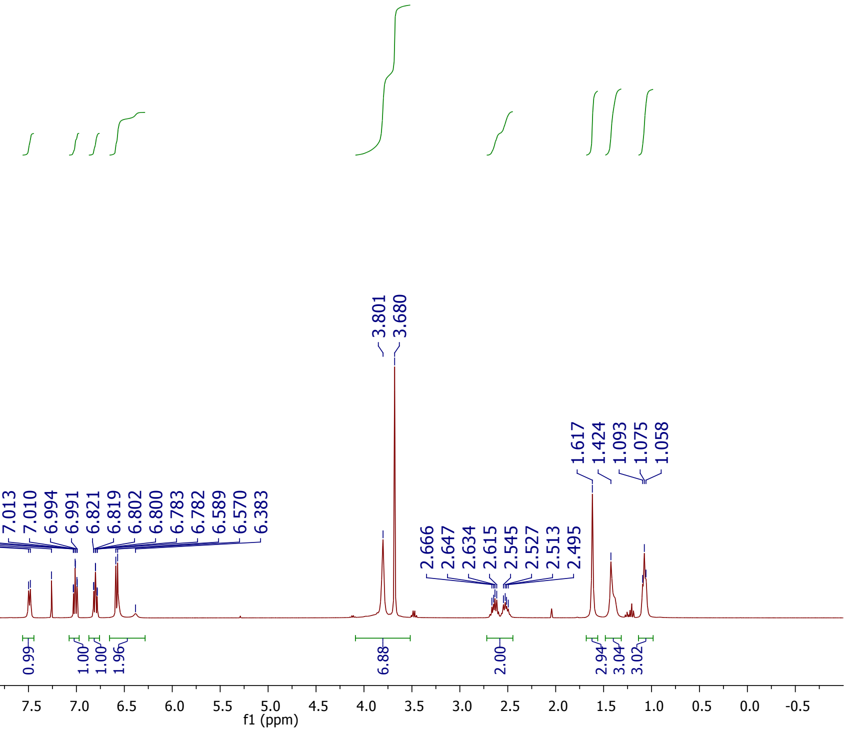

^{13}C NMR of 5g (100 MHz, CDCl$_3$)

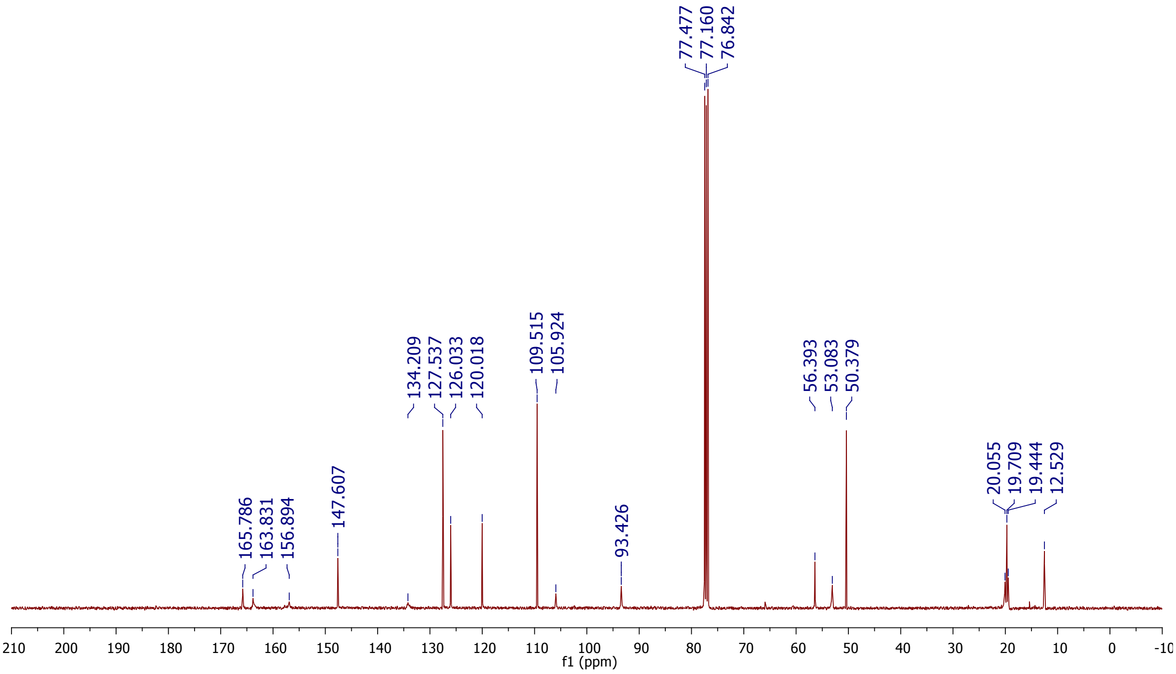

^{1}H NMR of 5h (400 MHz, $CDCl_3$)

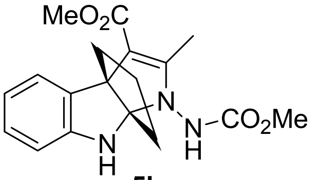

5h

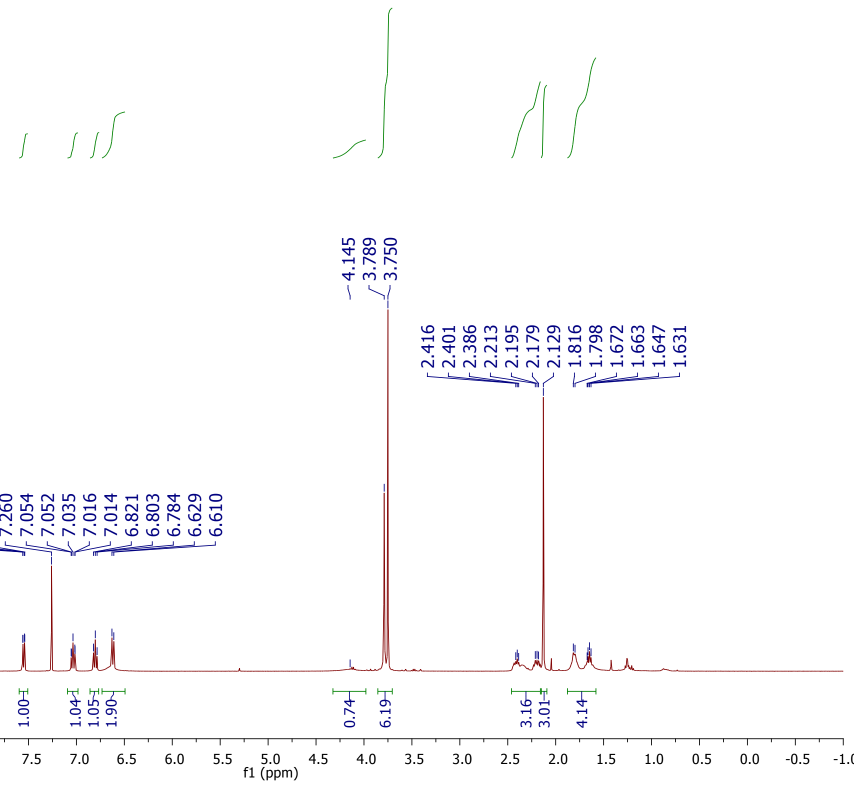

^{13}C NMR of 5h (100 MHz, $CDCl_3$)

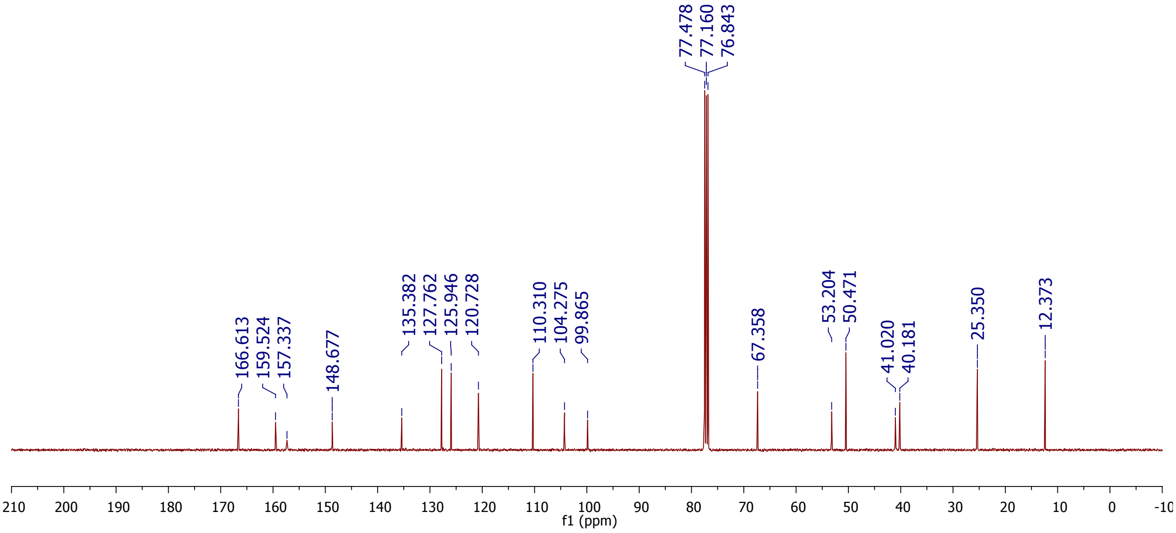

^{1}H NMR of 5i (400 MHz, DMSO-d_6)

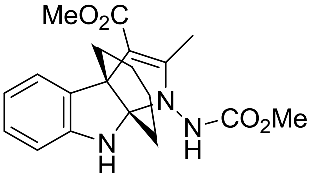

5i

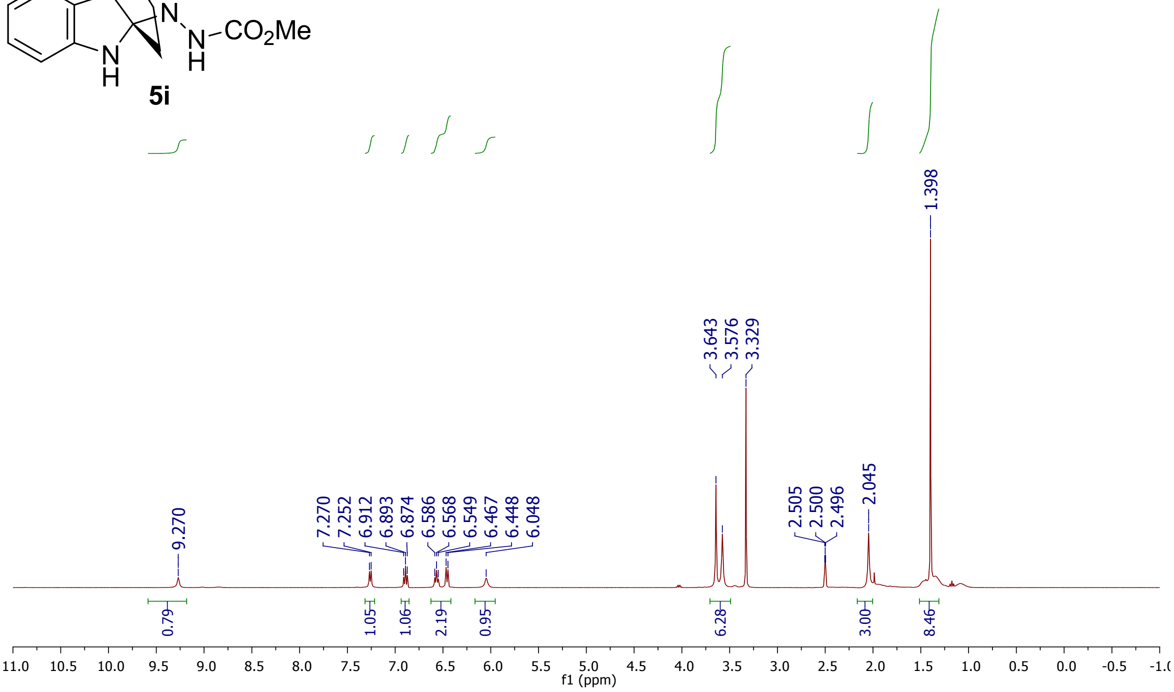

^{13}C NMR of 5i (100 MHz, DMSO-d_6)

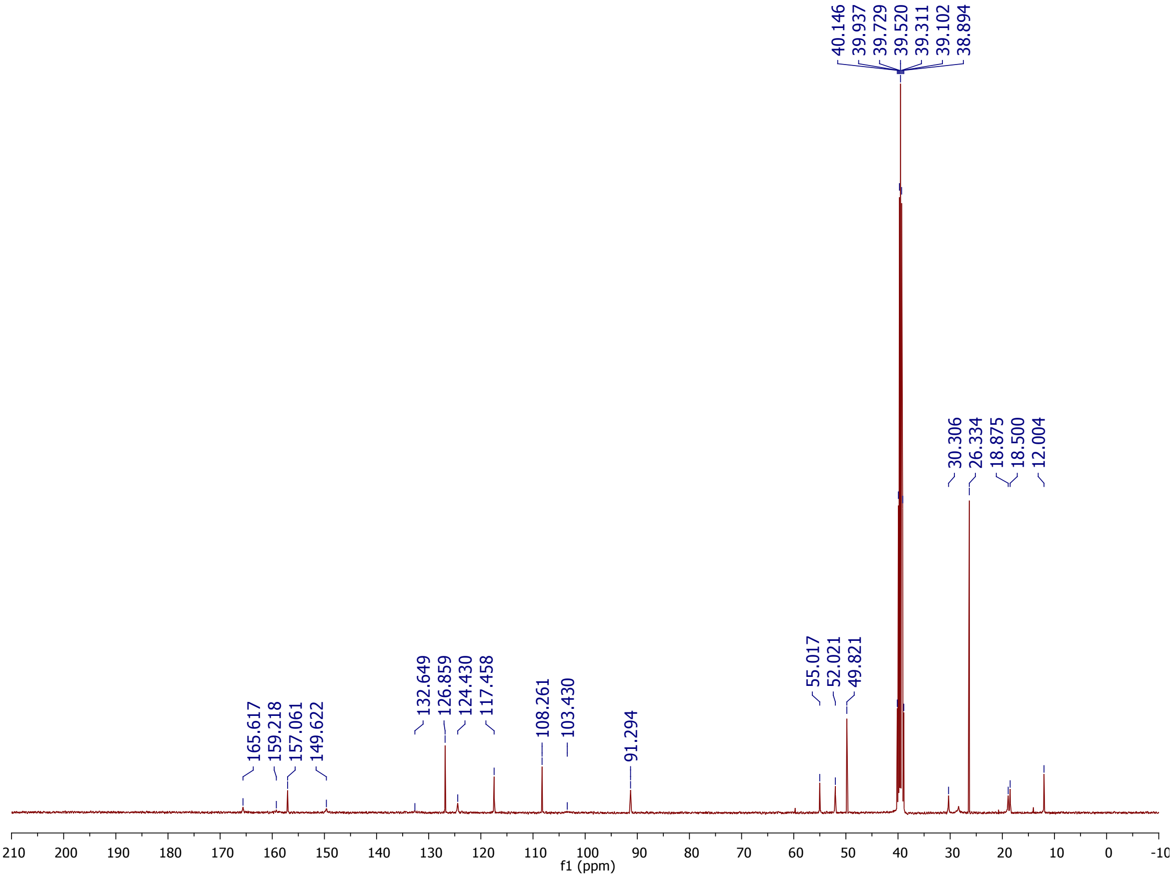

^{1}H NMR of 5j (400 MHz, DMSO-d_6)

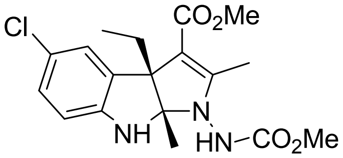

5j

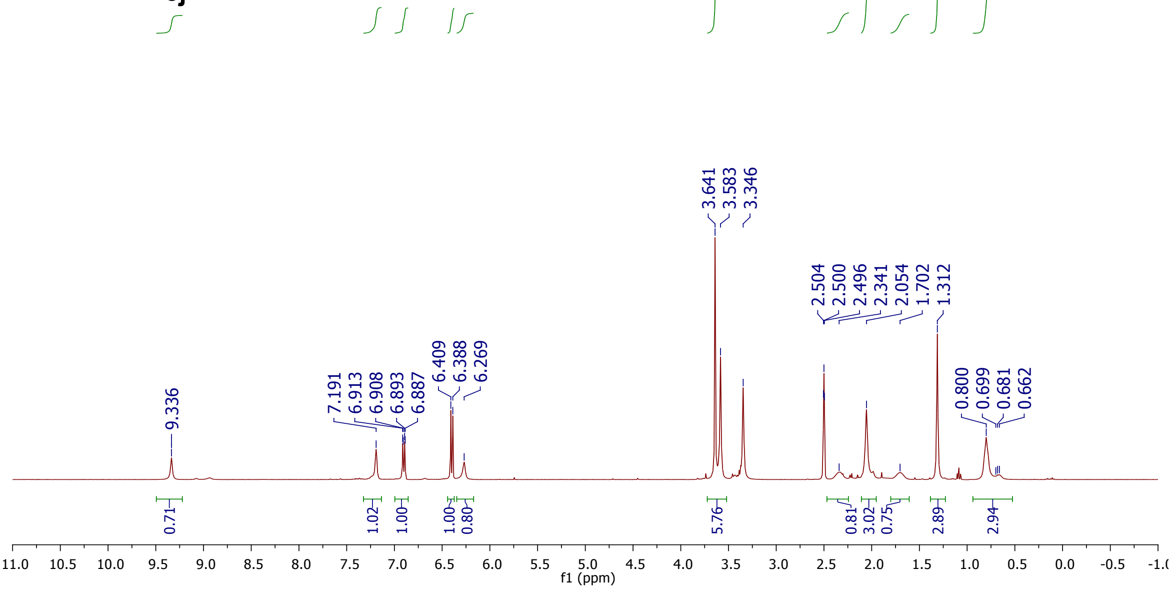

^{13}C NMR of 5j (100 MHz, DMSO-d_6)

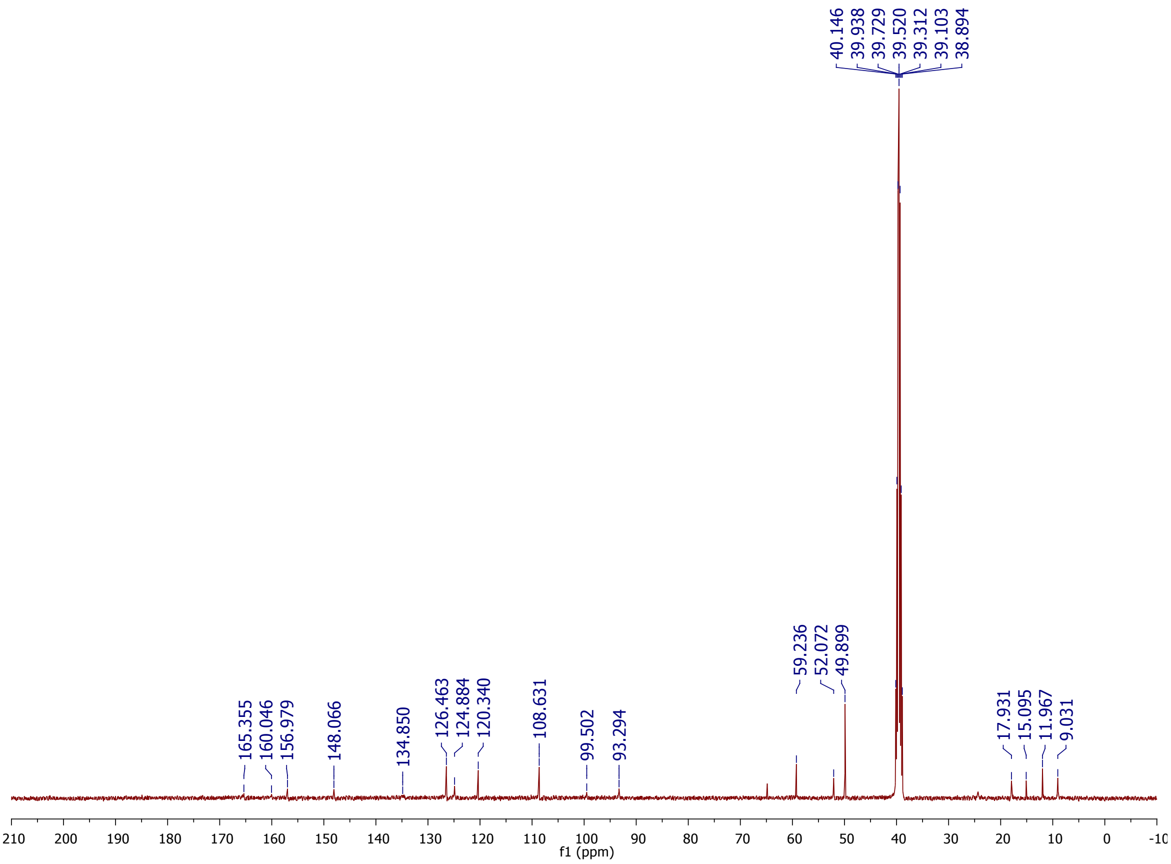

^{1}H NMR of 5k (400 MHz, DMSO-d_6)

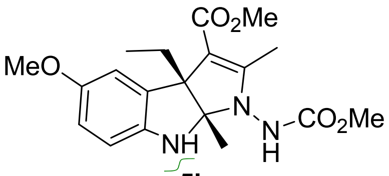

5k

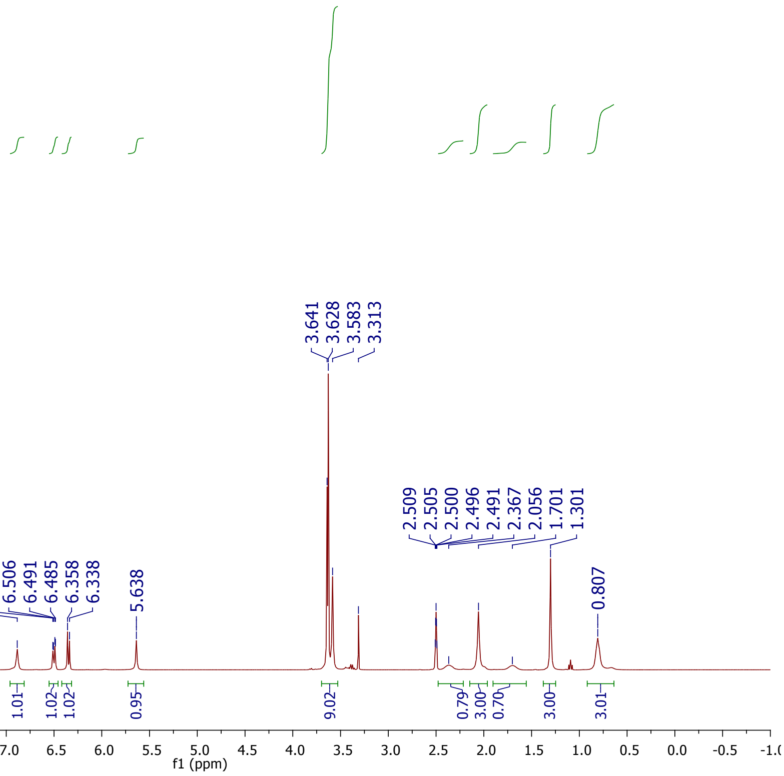

^{13}C NMR of 5k (100 MHz, DMSO-d_6)

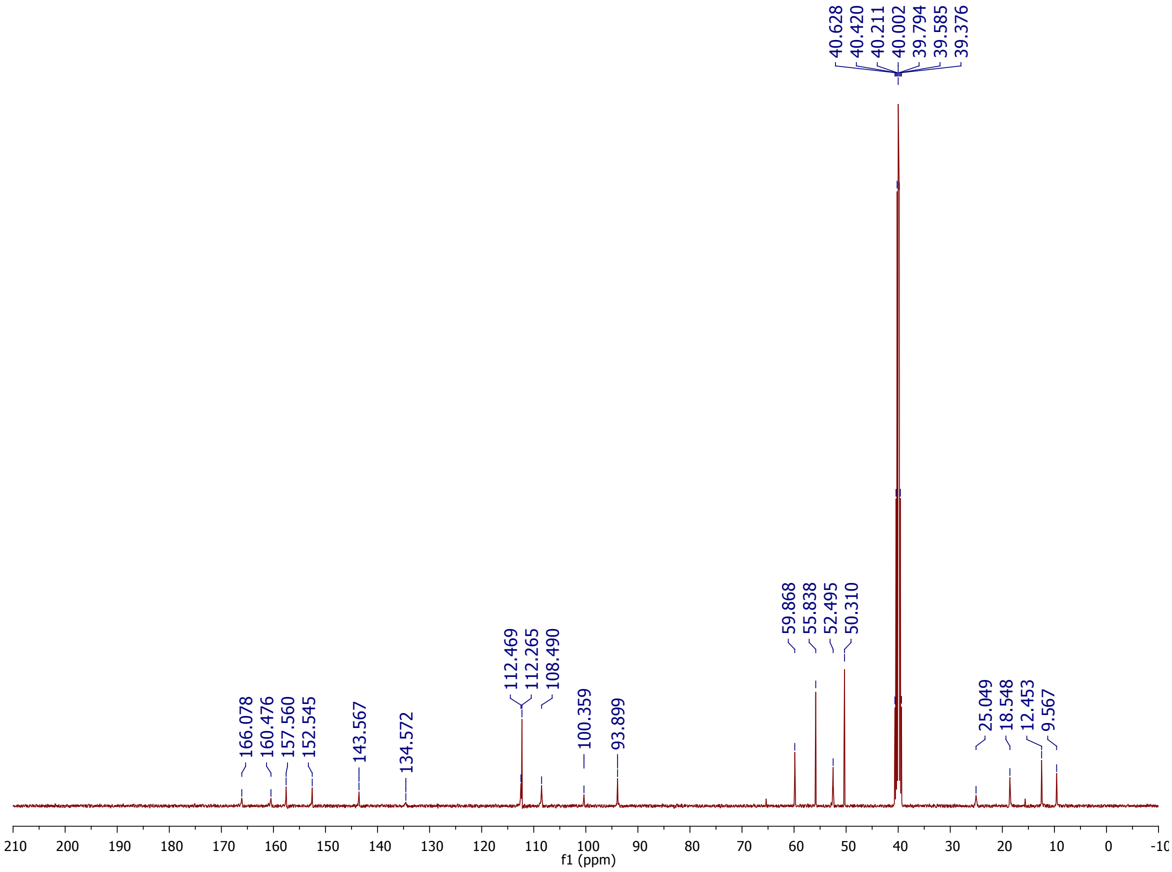

^{1}H NMR of 5l (400 MHz, $CDCl_3$)

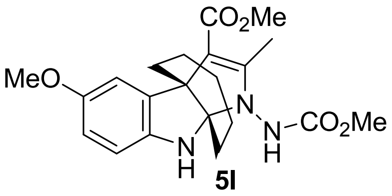

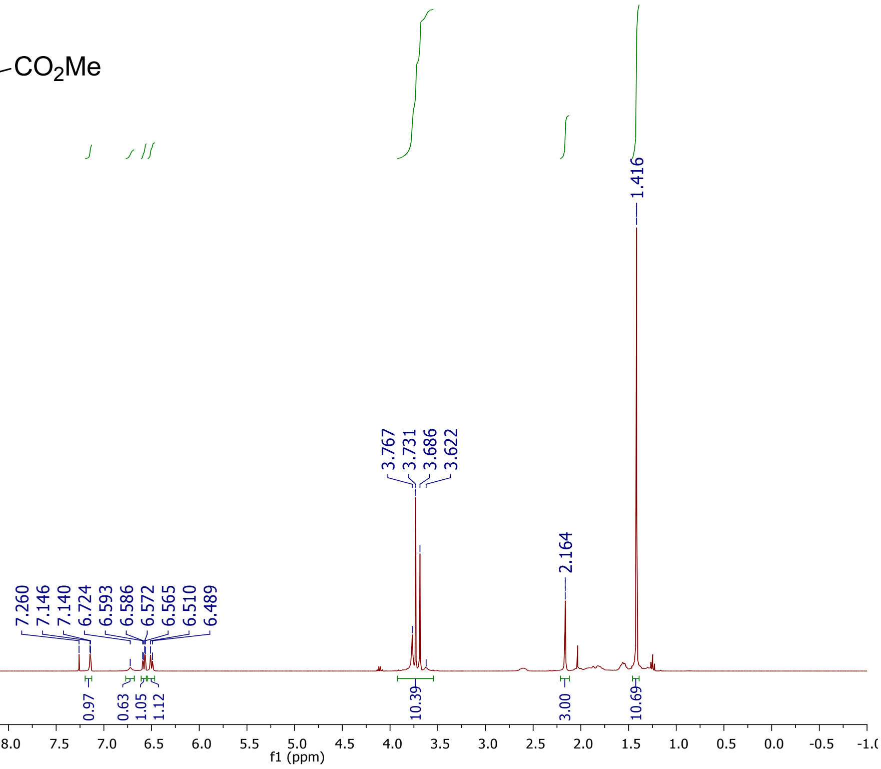

^{13}C NMR of 5l (100 MHz, $CDCl_3$)

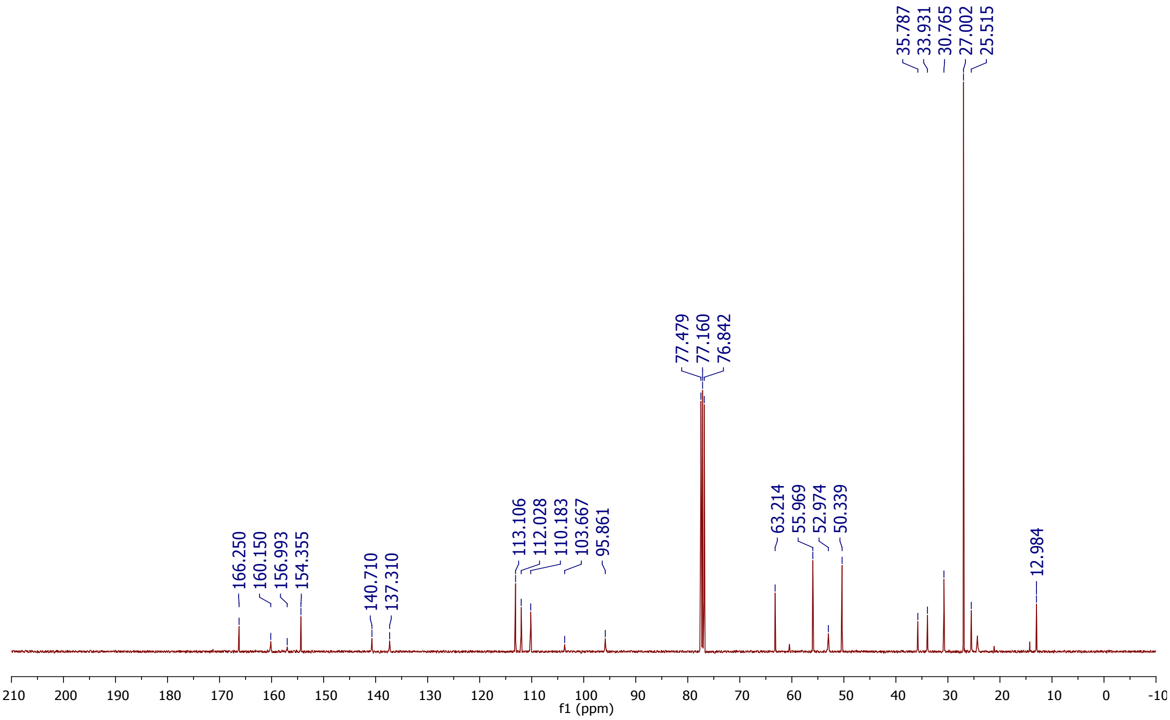

^{1}H NMR of 5m (400 MHz, DMSO-d_6)

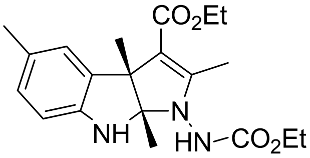

5m

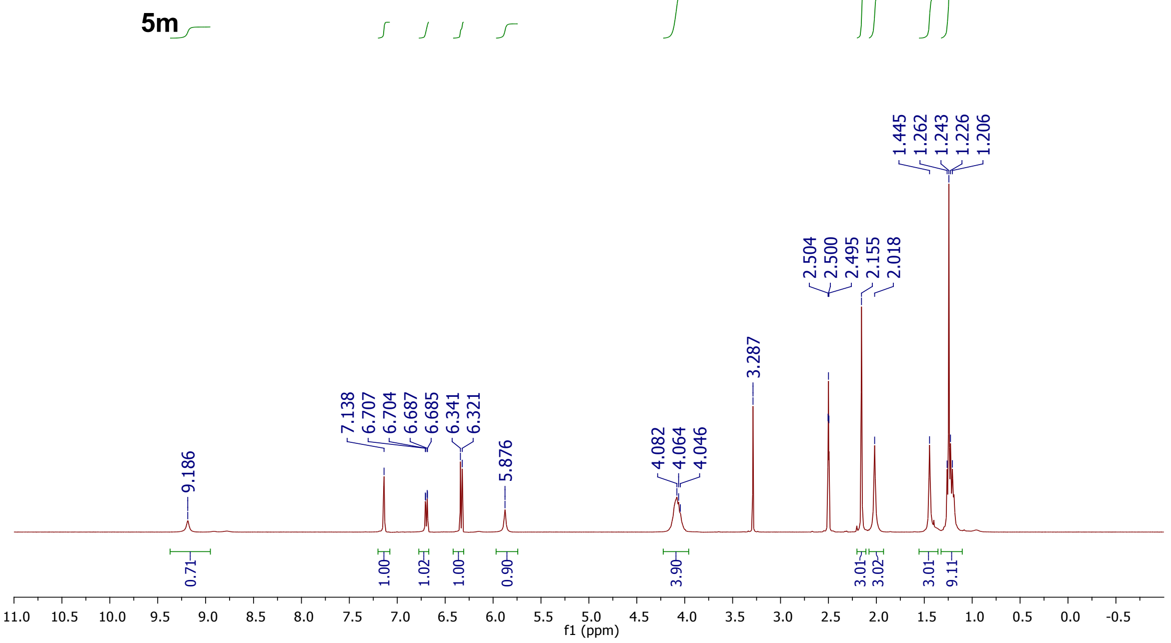

^{13}C NMR of 5m (100 MHz, DMSO-d_6)

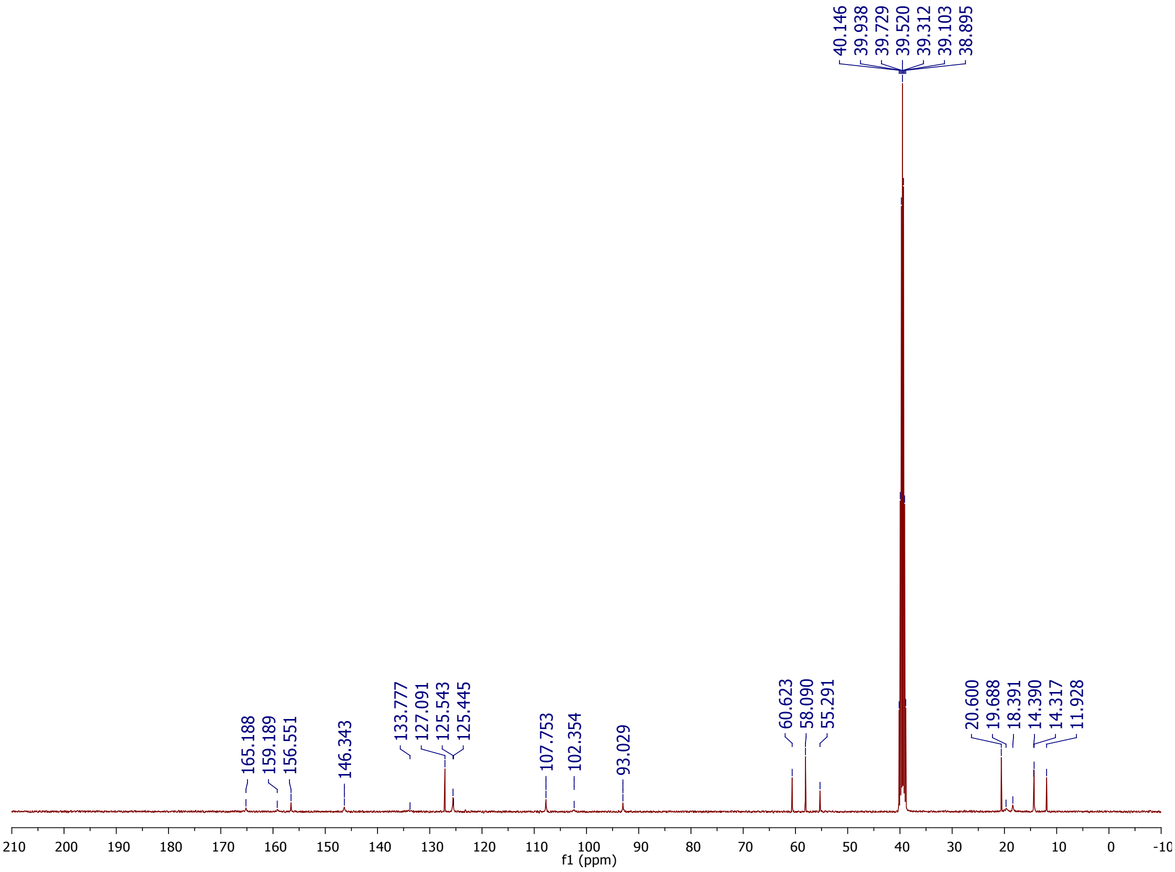

^{1}H NMR of 5n (400 MHz, DMSO-d_6)

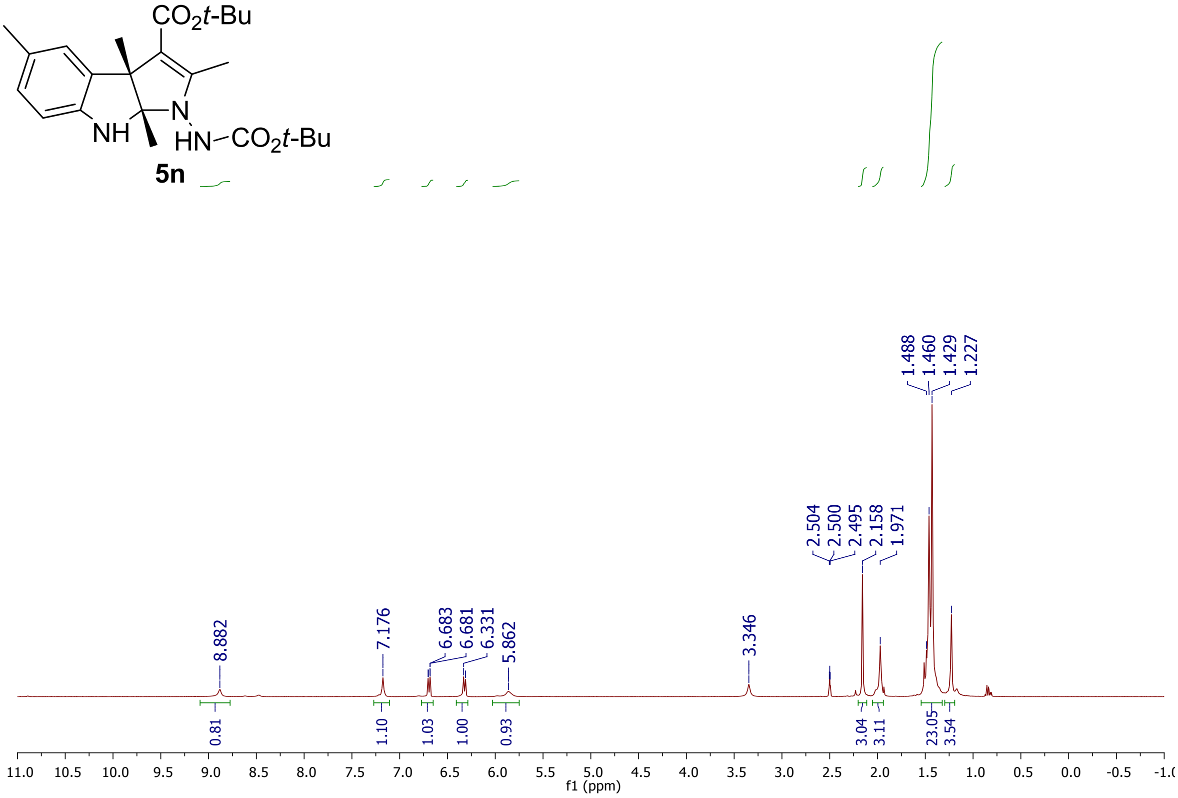

^{13}C NMR of 5n (100 MHz, DMSO-d_6)

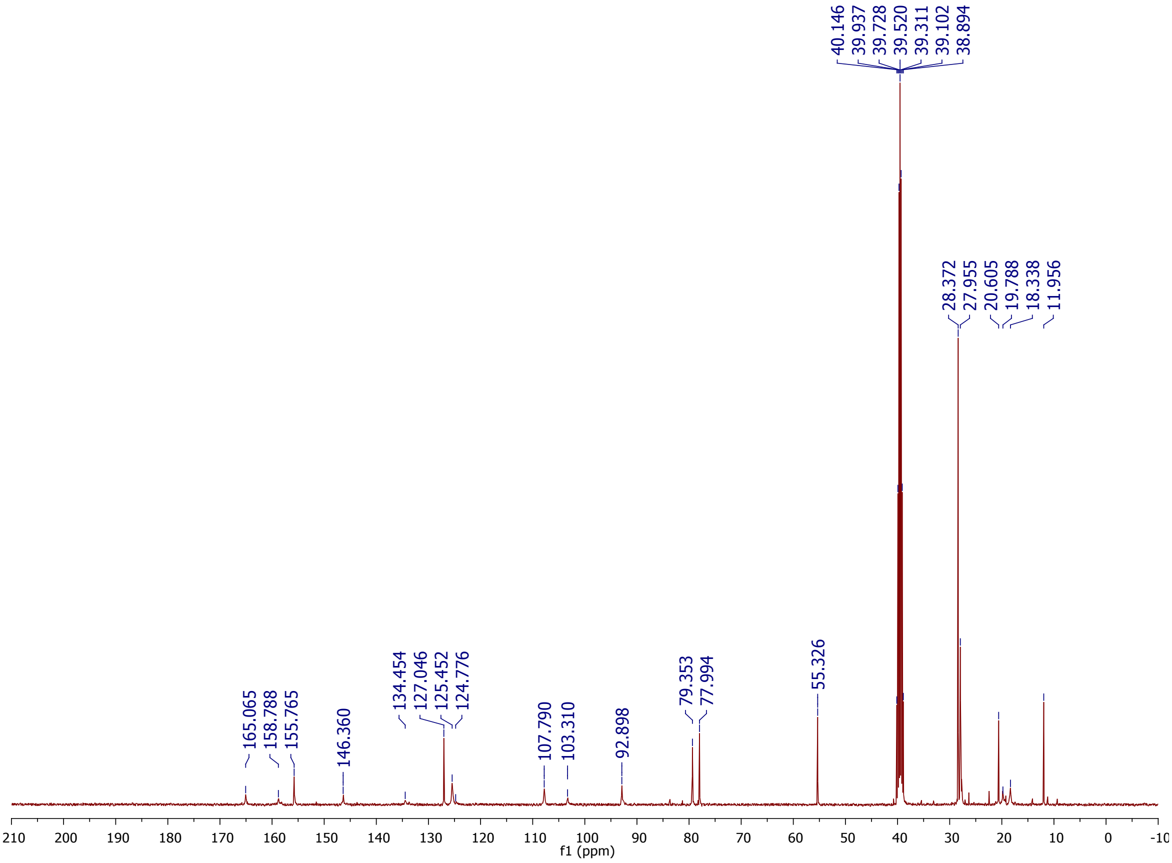

^{1}H NMR of 5o (400 MHz, CDCl$_3$)

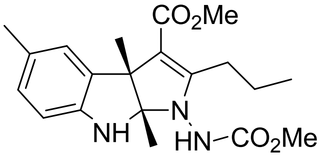

5o

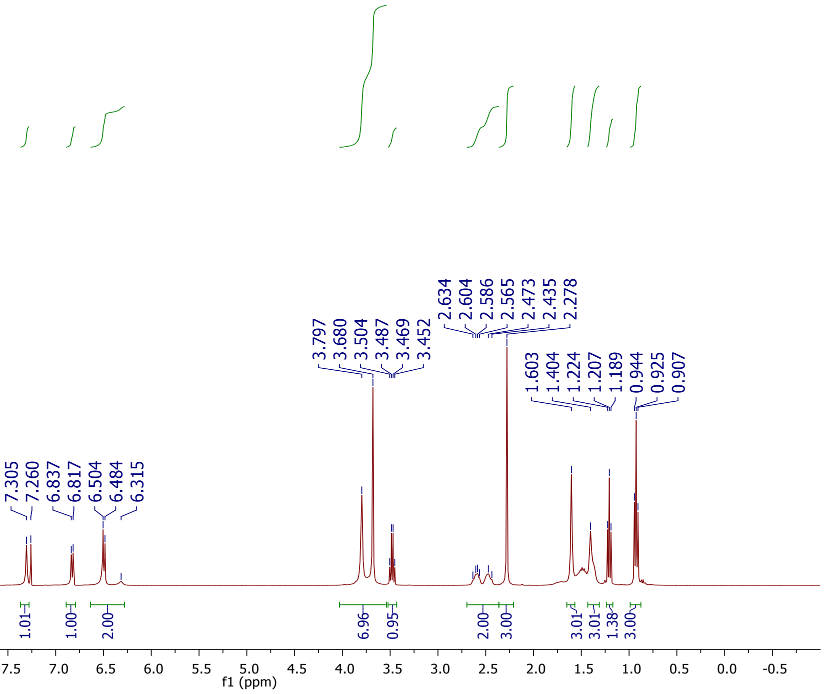

^{13}C NMR of 5o (100 MHz, CDCl$_3$)

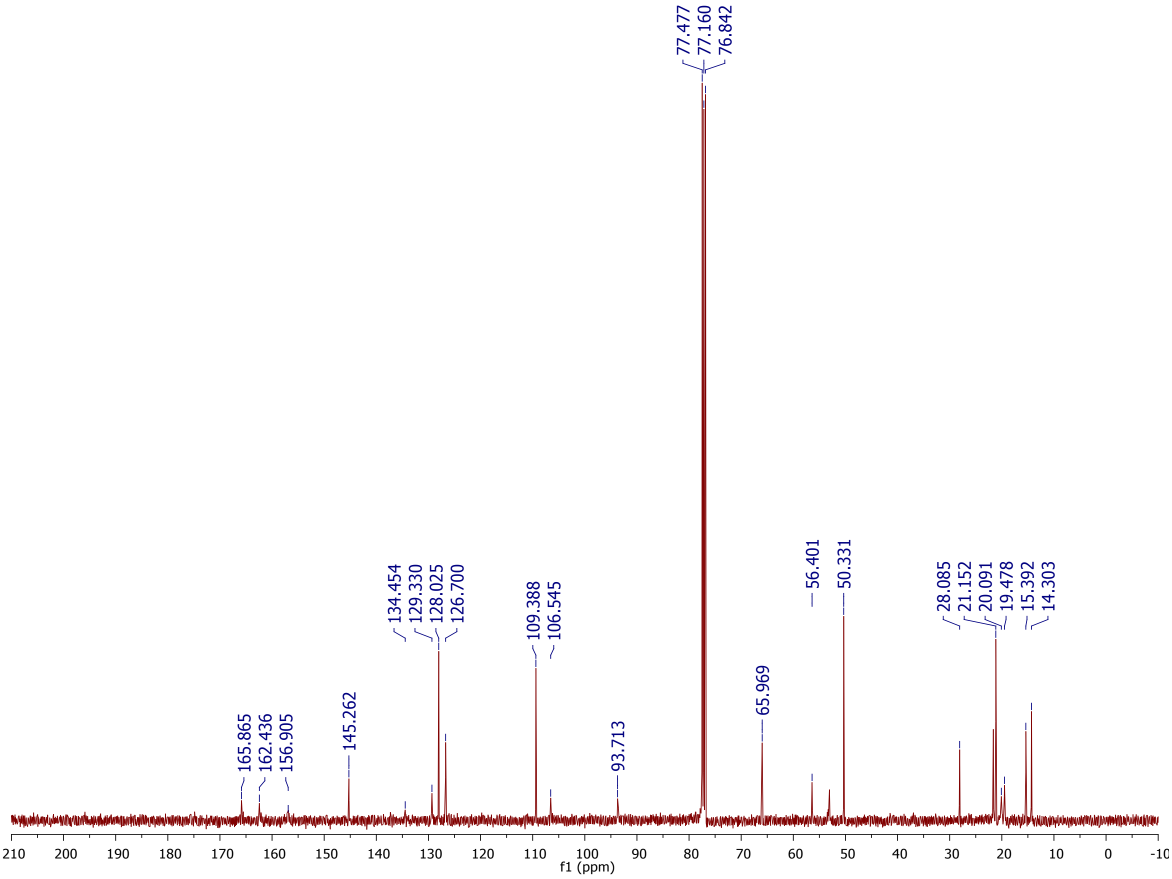

^{1}H NMR of 5p (400 MHz, $CDCl_3$)

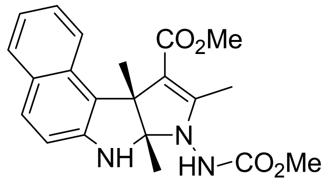

5p

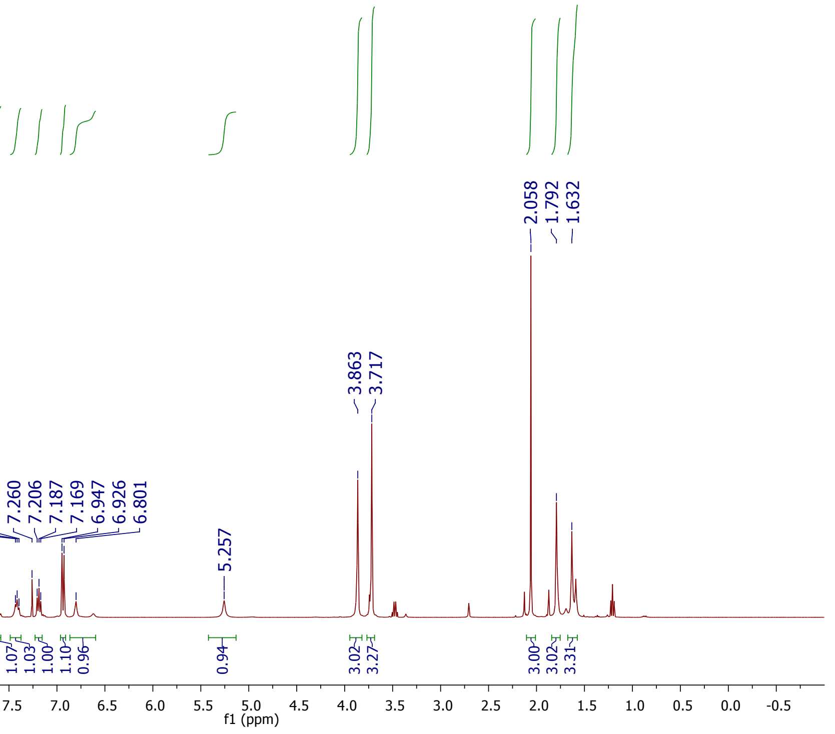

^{13}C NMR of 5p (100 MHz, $CDCl_3$)

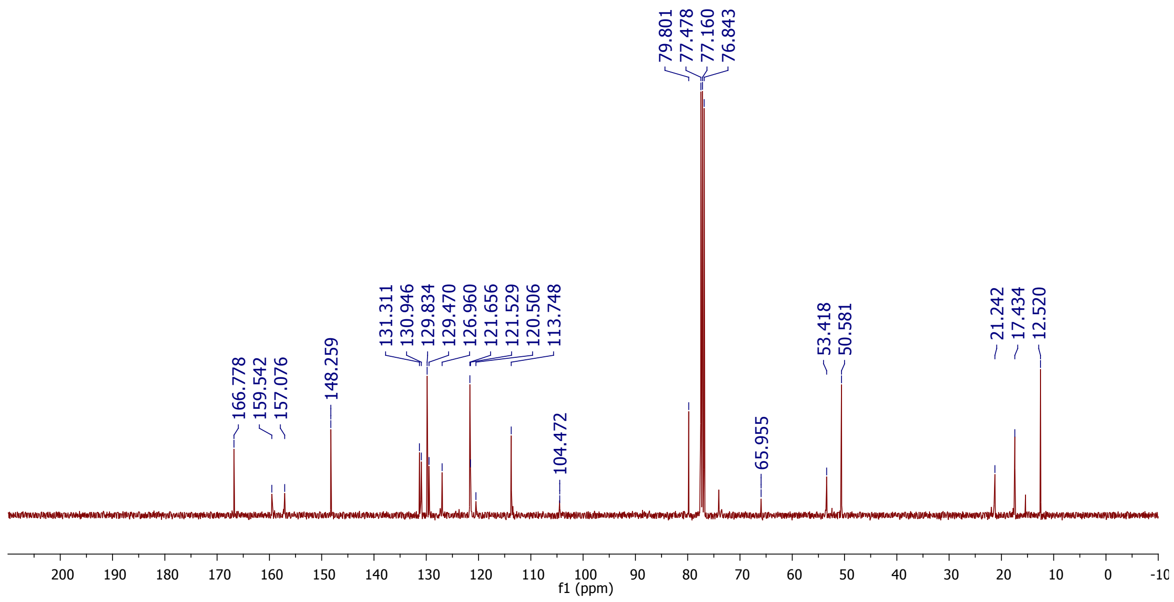

^{1}H NMR of 5q (400 MHz, DMSO-*d*$_6$)

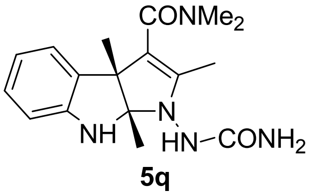

5q

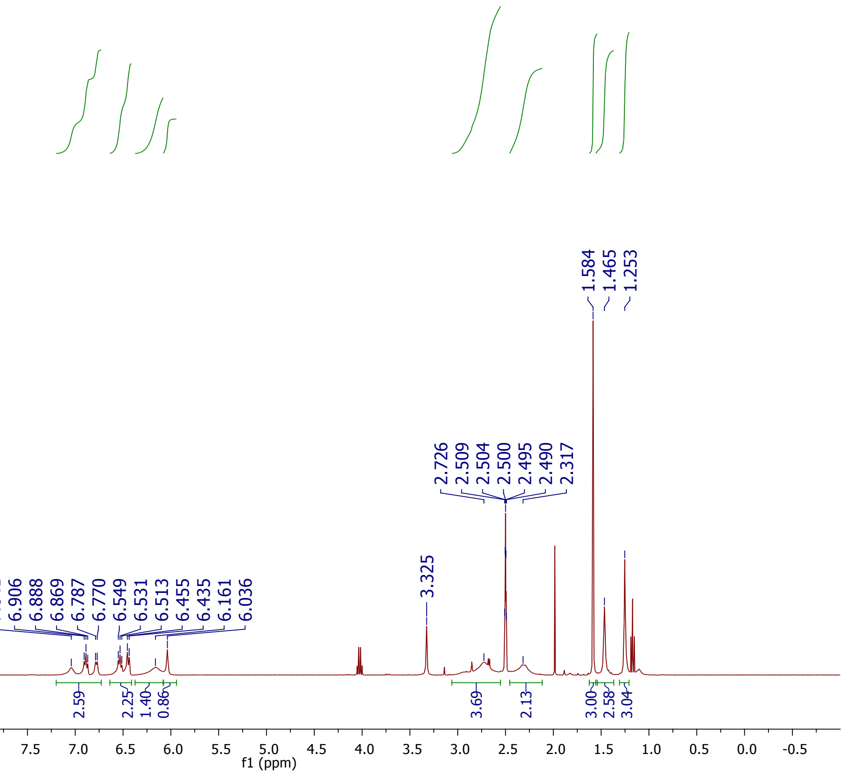

^{13}C NMR of 5q (100 MHz, DMSO-*d*$_6$)

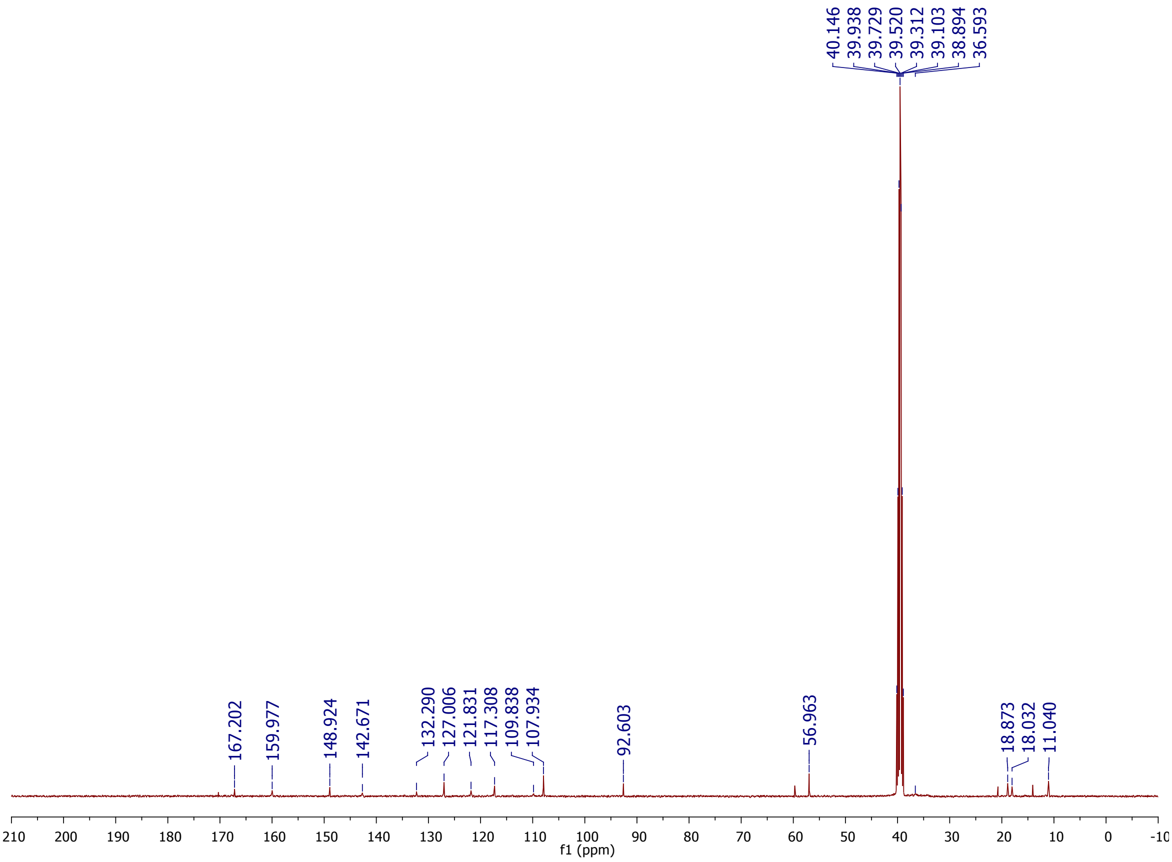

^{1}H NMR of 5r (400 MHz, DMSO-*d*[6])

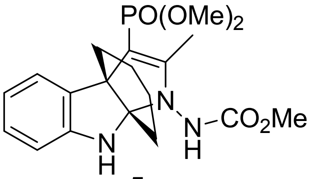

5r

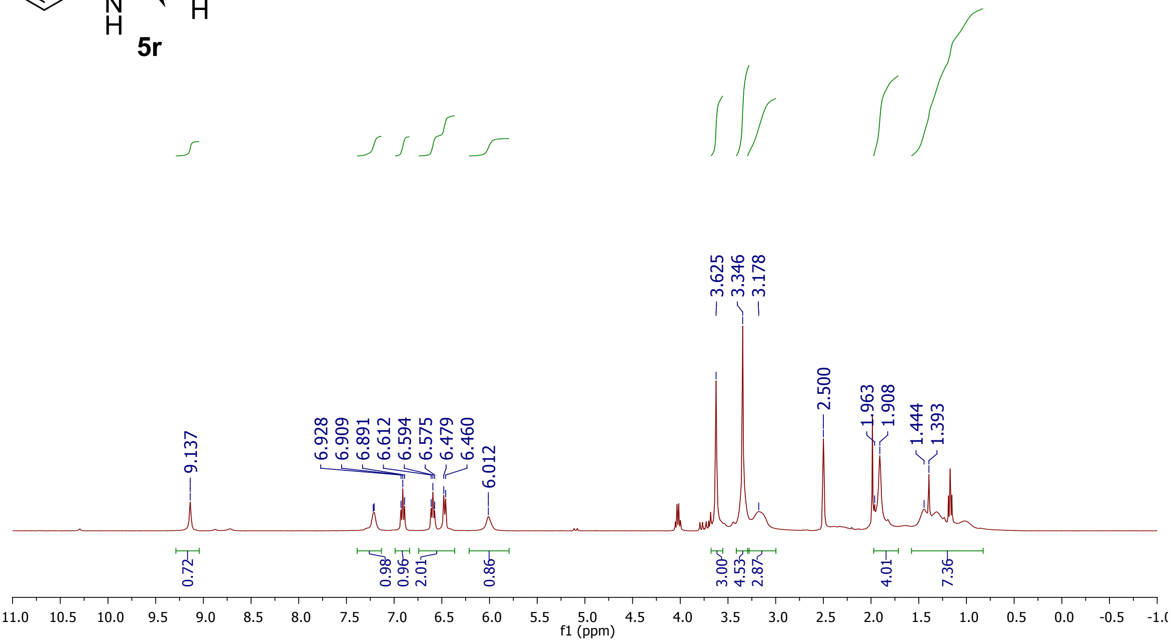

^{13}C NMR of 5r (100 MHz, DMSO-*d*[6])

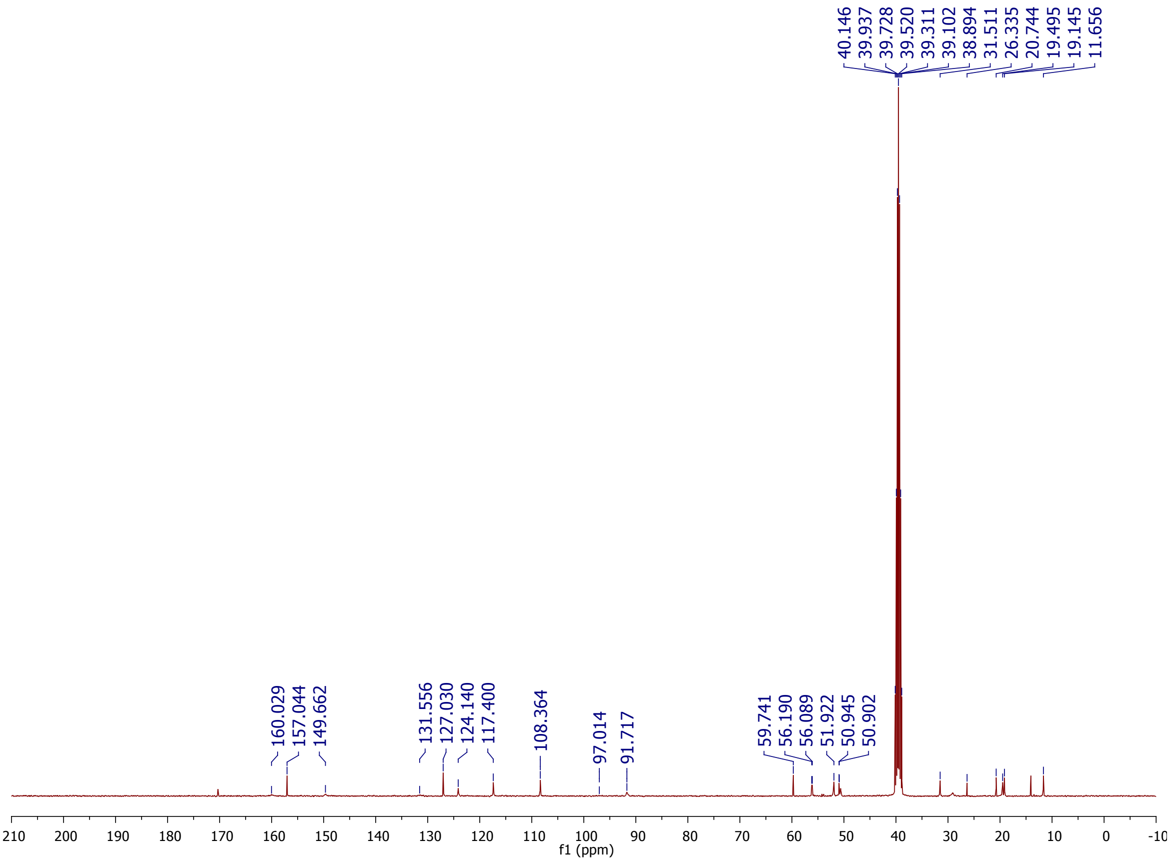

^{1}H NMR of 5s (400 MHz, DMSO-d_6)

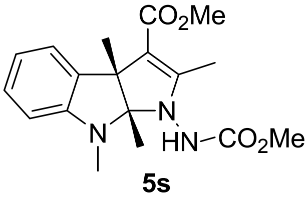

5s

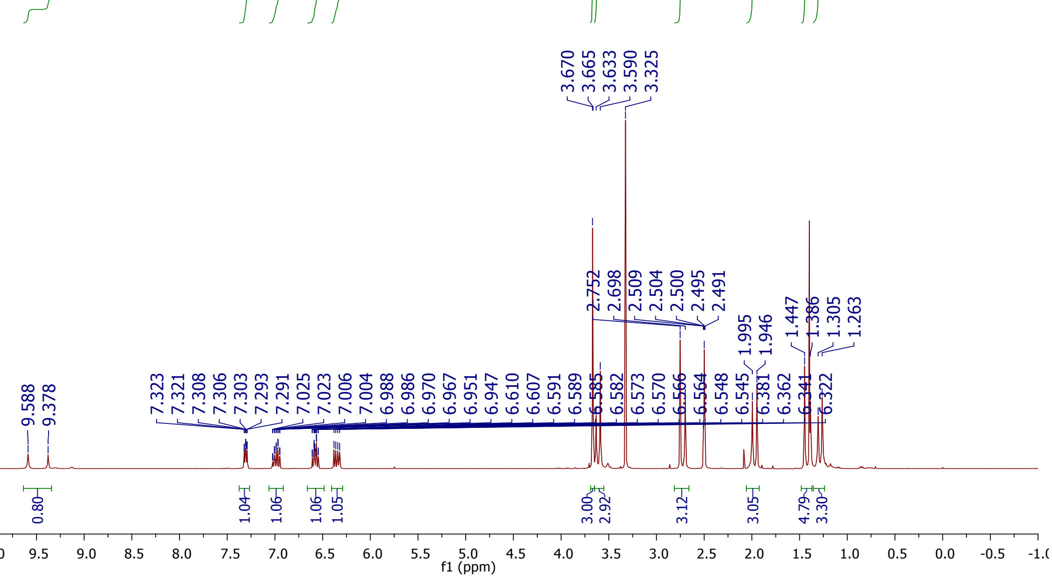

^{13}C NMR of 5s (100 MHz, DMSO-d_6)

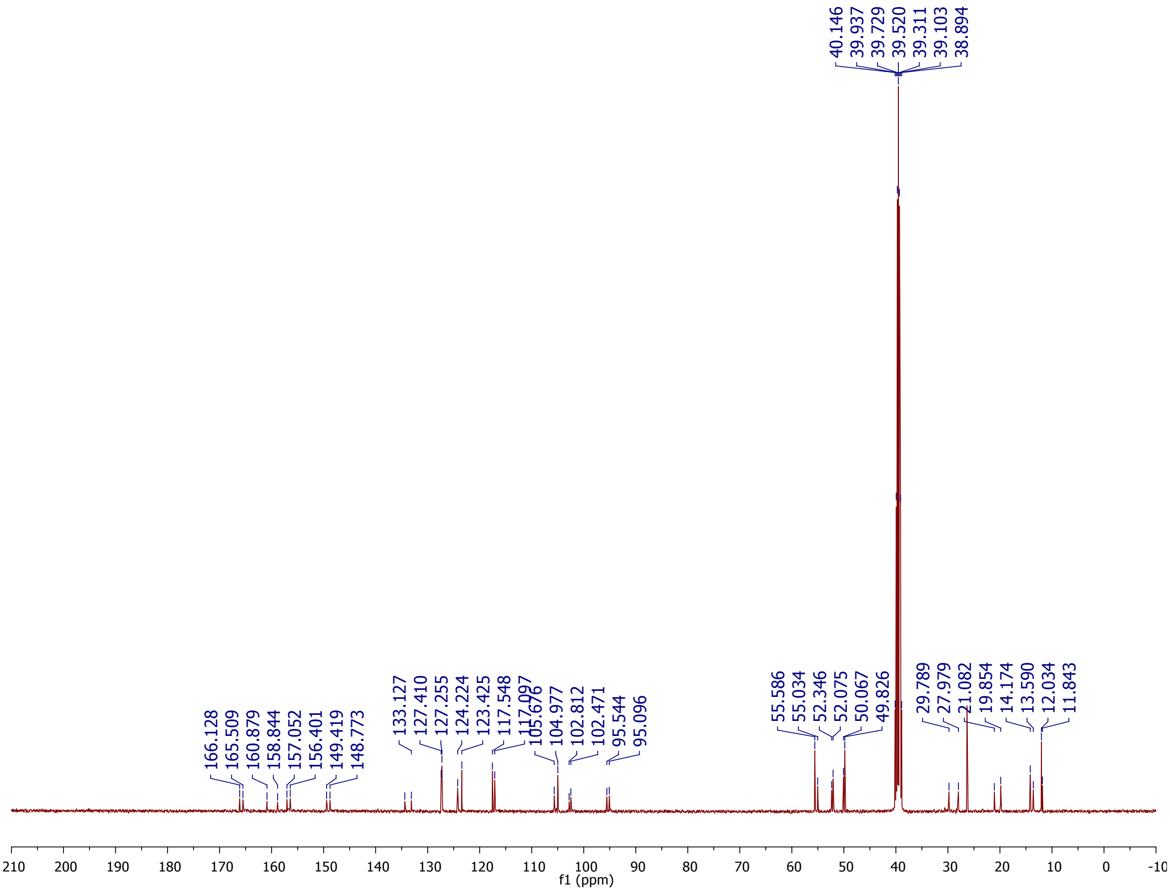

^{1}H NMR of 4a (400 MHz, CDCl$_3$)

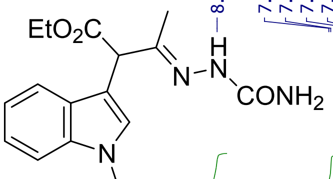

4a

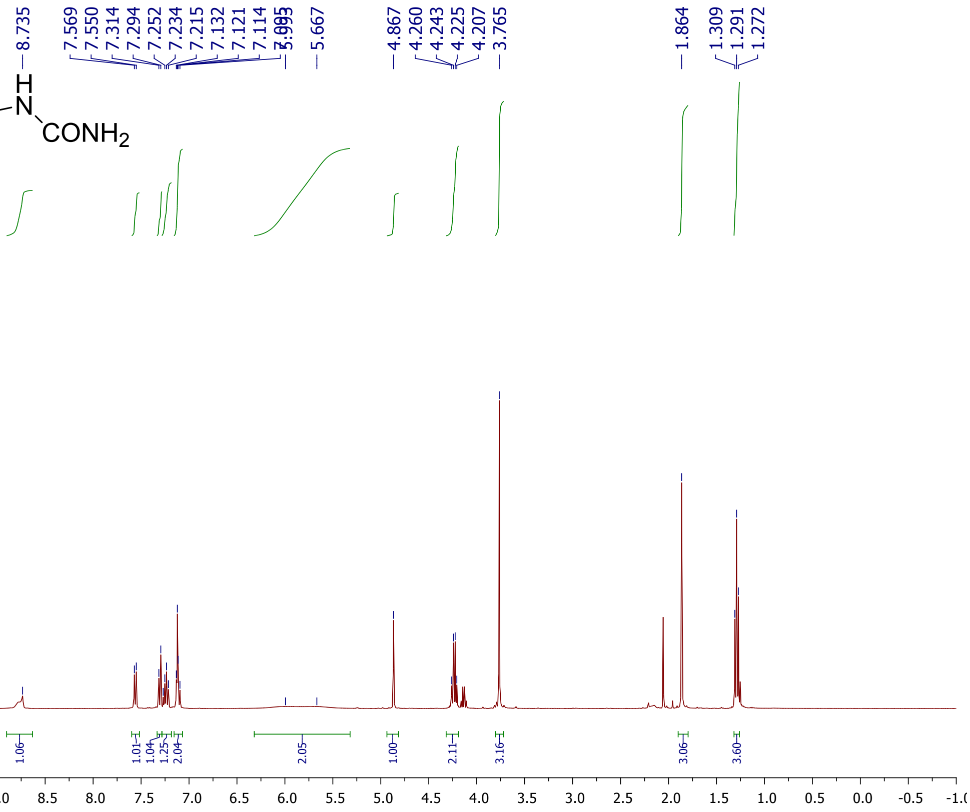

^{13}C NMR of 4a (100 MHz, CDCl$_3$)

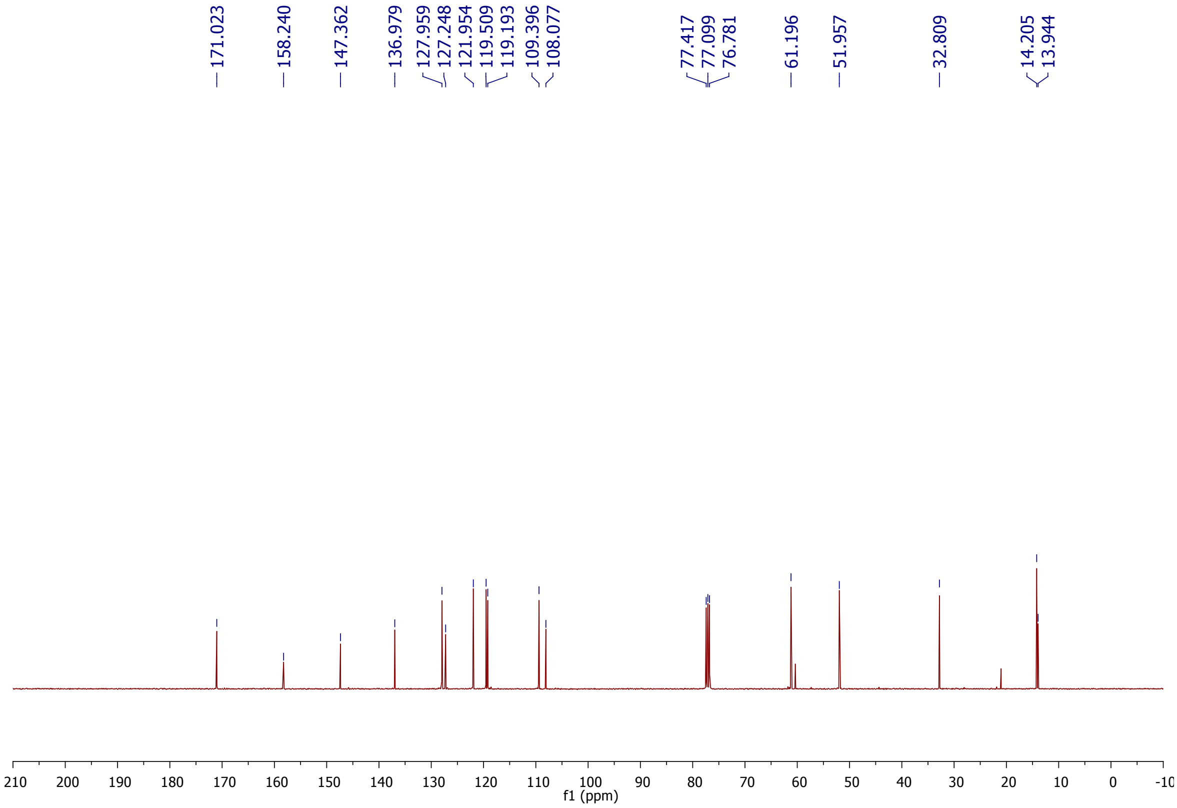

^{1}H NMR of 4b (400 MHz, DMSO-d_6)

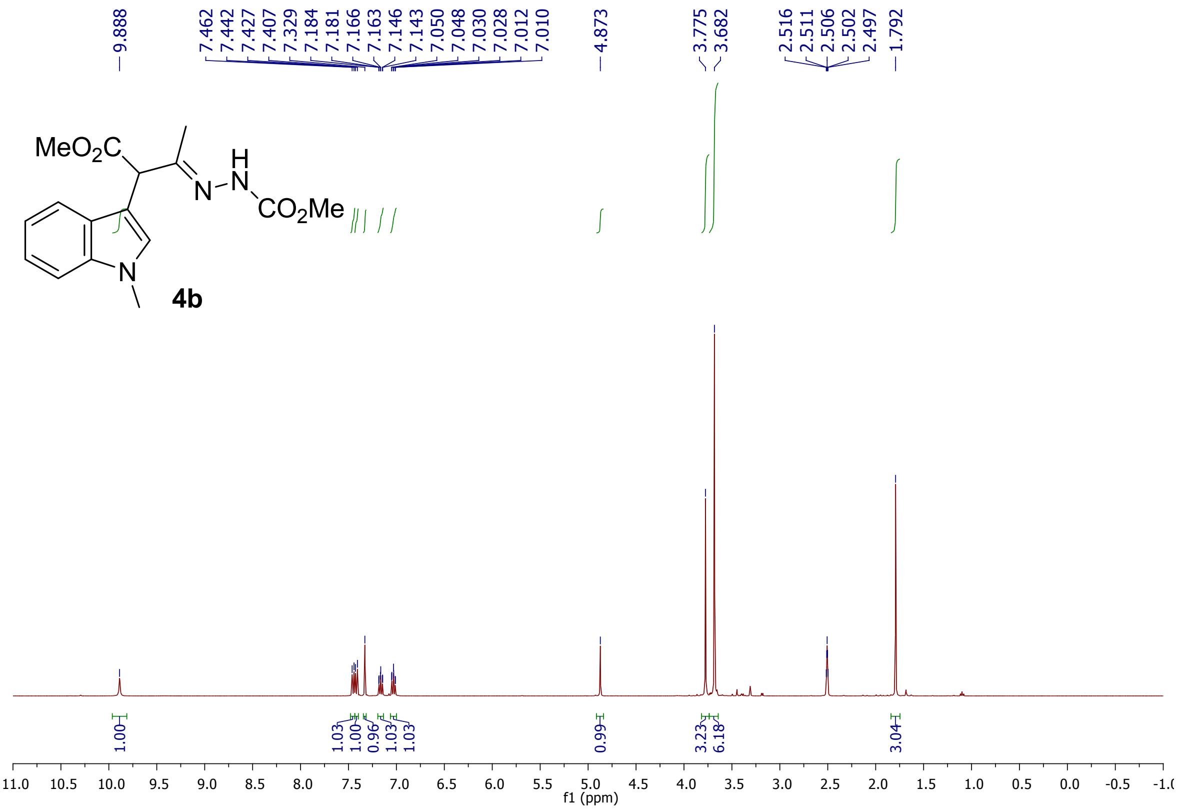

^{13}C NMR of 4b (100 MHz, DMSO-d_6)

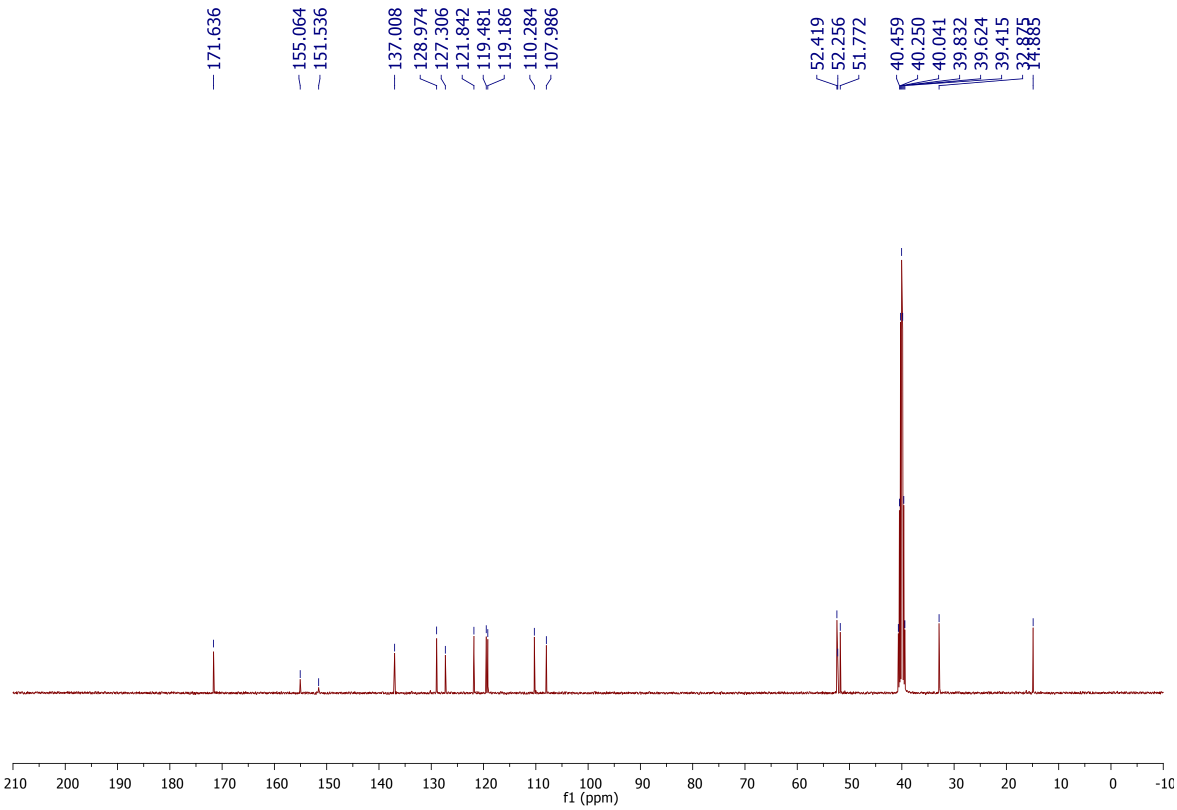

^{1}H NMR of 4c (400 MHz, DMSO-d_6)

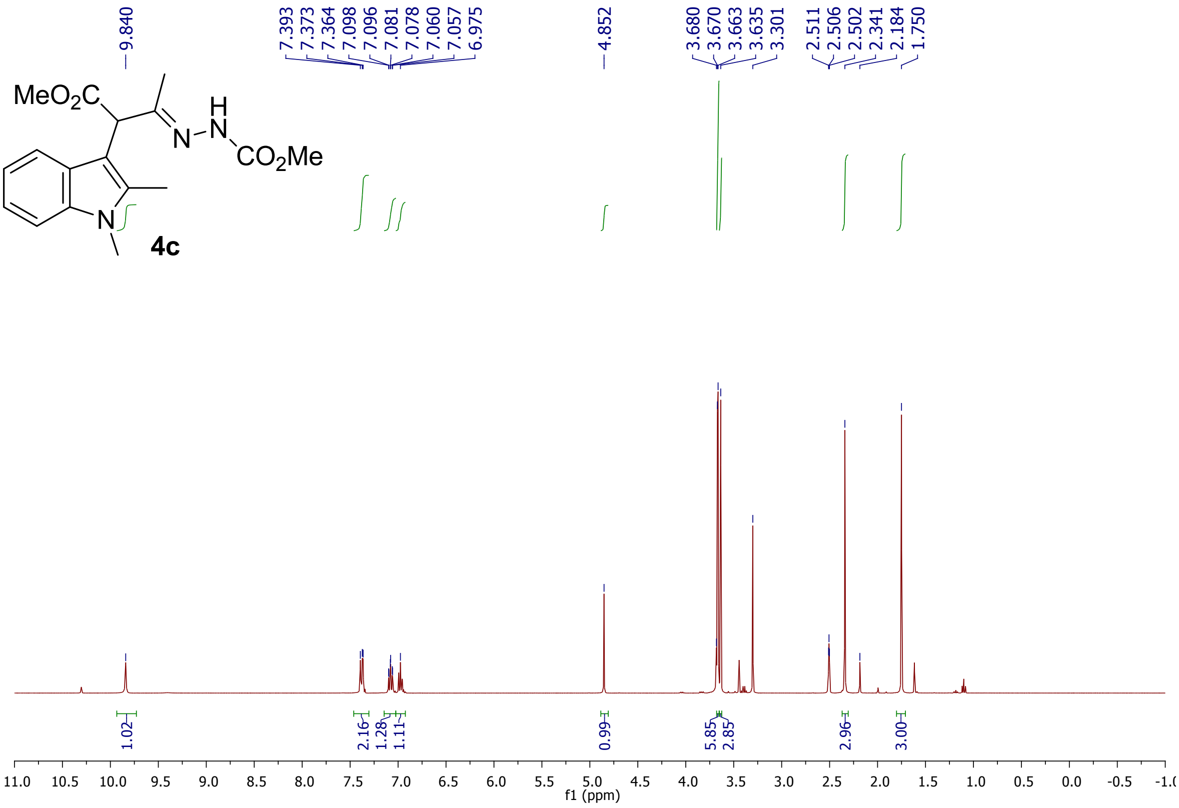

^{13}C NMR of 4c (100 MHz, DMSO-d_6)

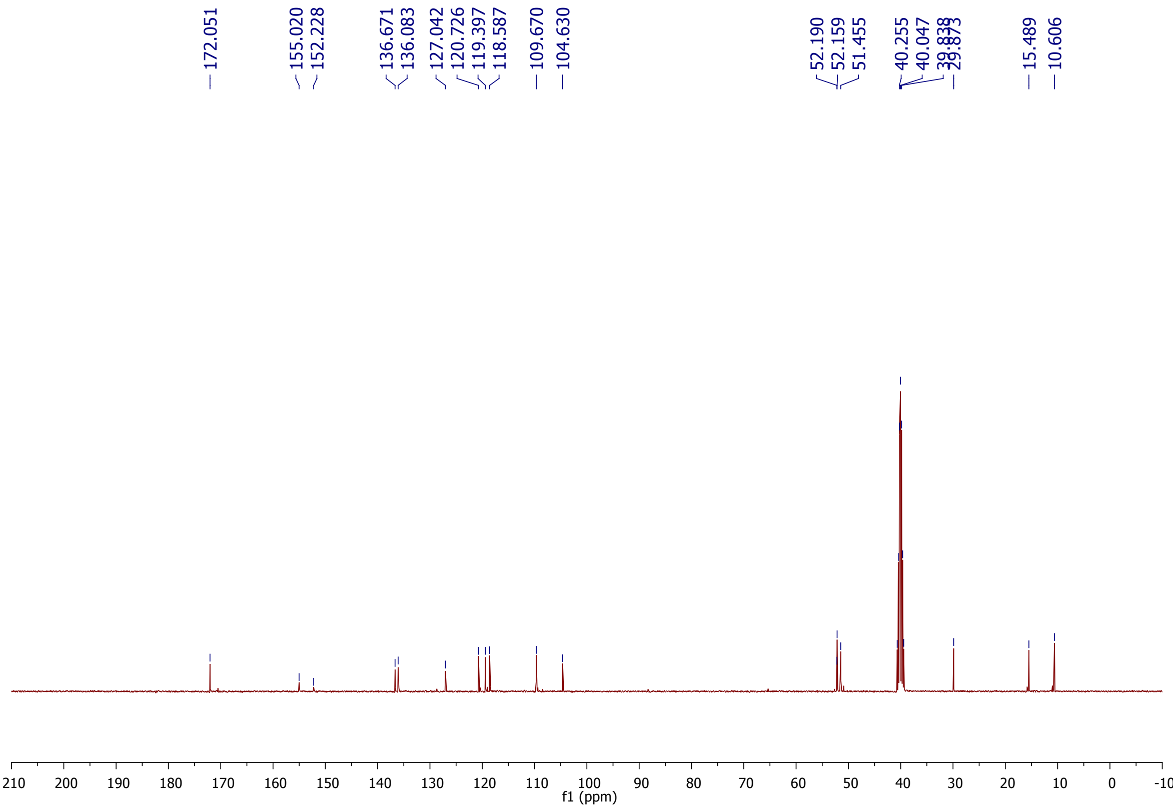

^{1}H NMR of 4d (400 MHz, DMSO-d_6)

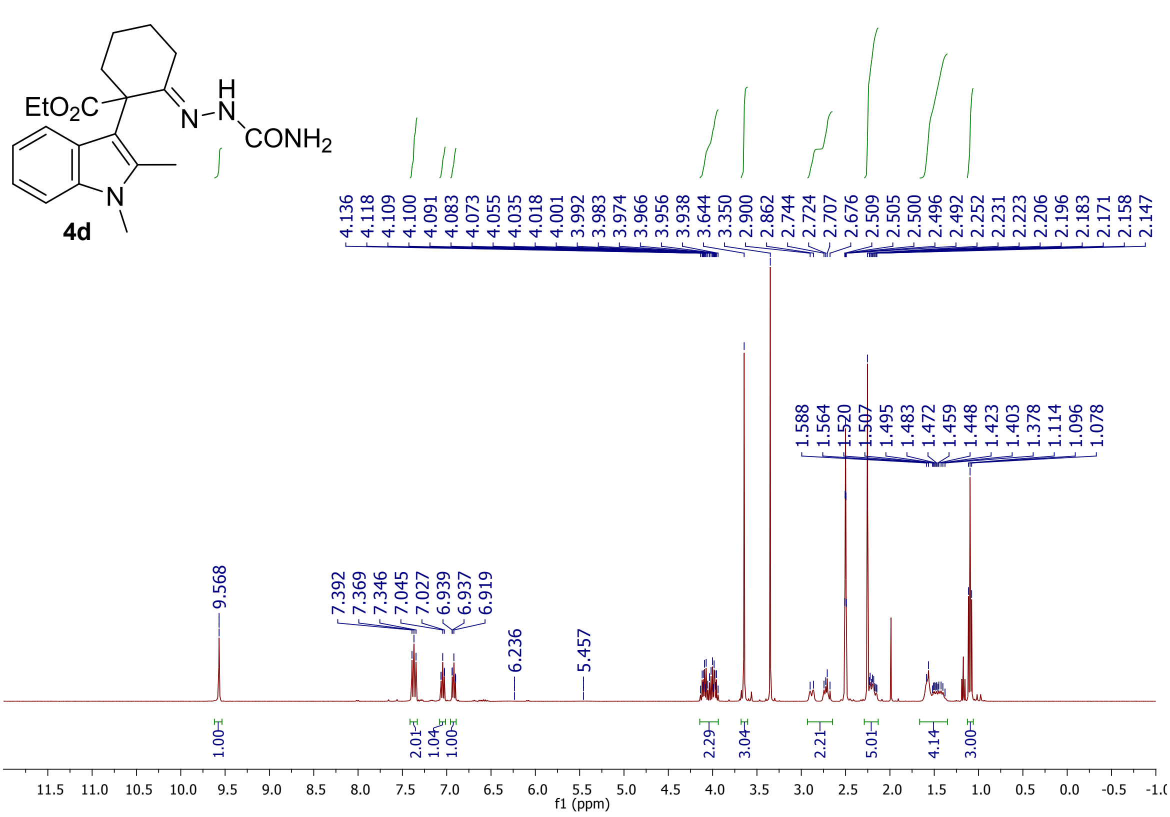

4d

^{13}C NMR of 4d (100 MHz, DMSO-d_6)

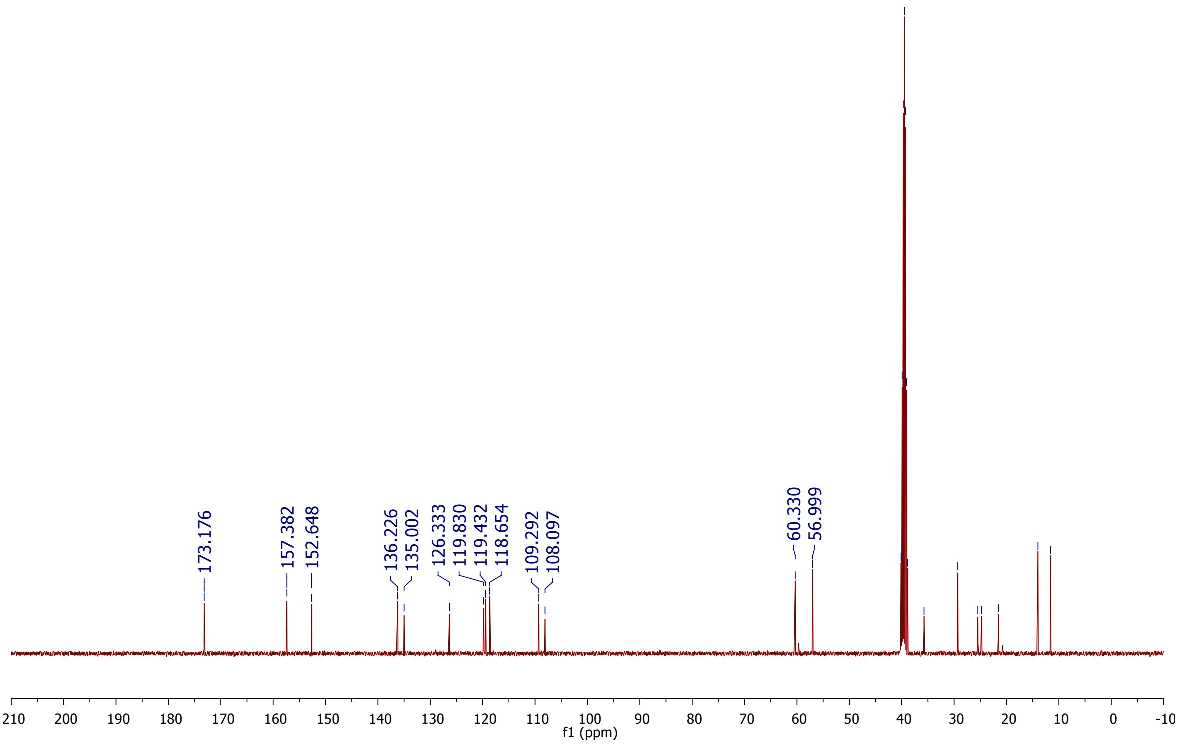

^{1}H NMR of 4e (400 MHz, DMSO-d_6)

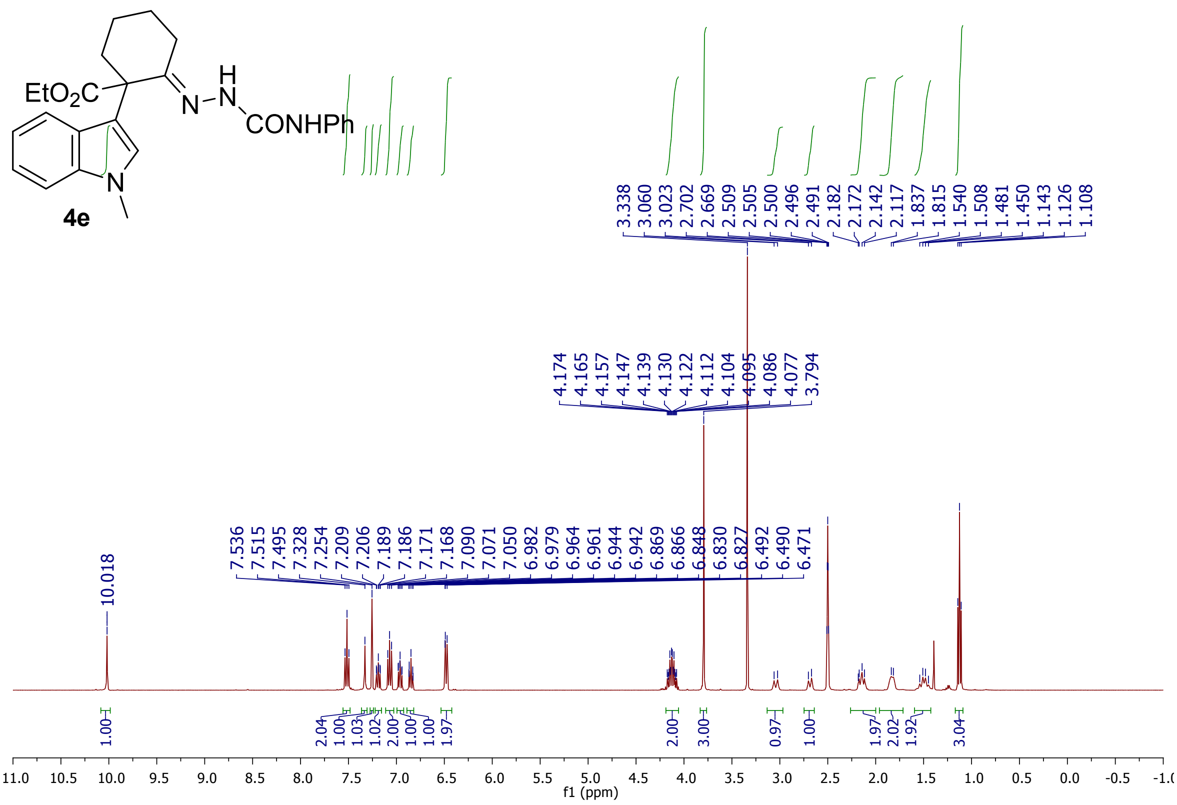

^{13}C NMR of 4e (100 MHz, DMSO-d_6

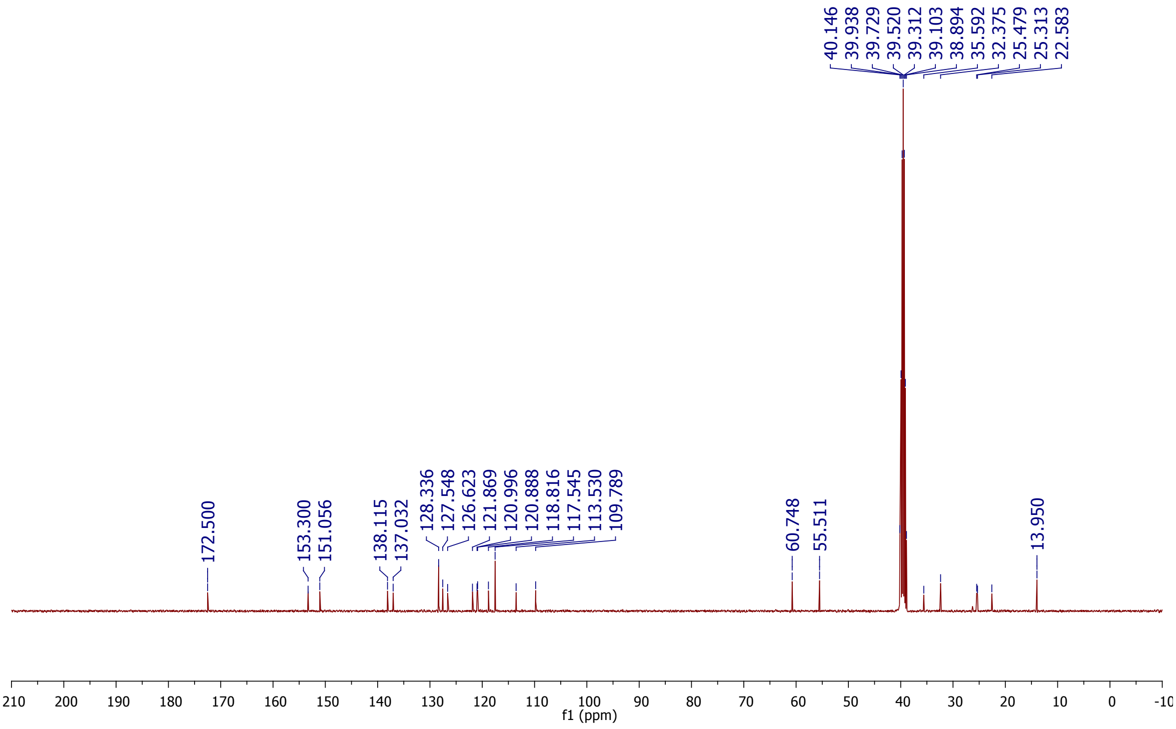

^{1}H NMR of 4f (400 MHz, DMSO-d_6

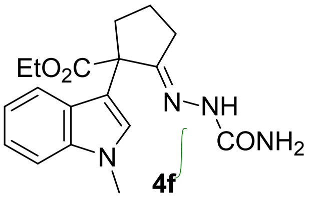

4f

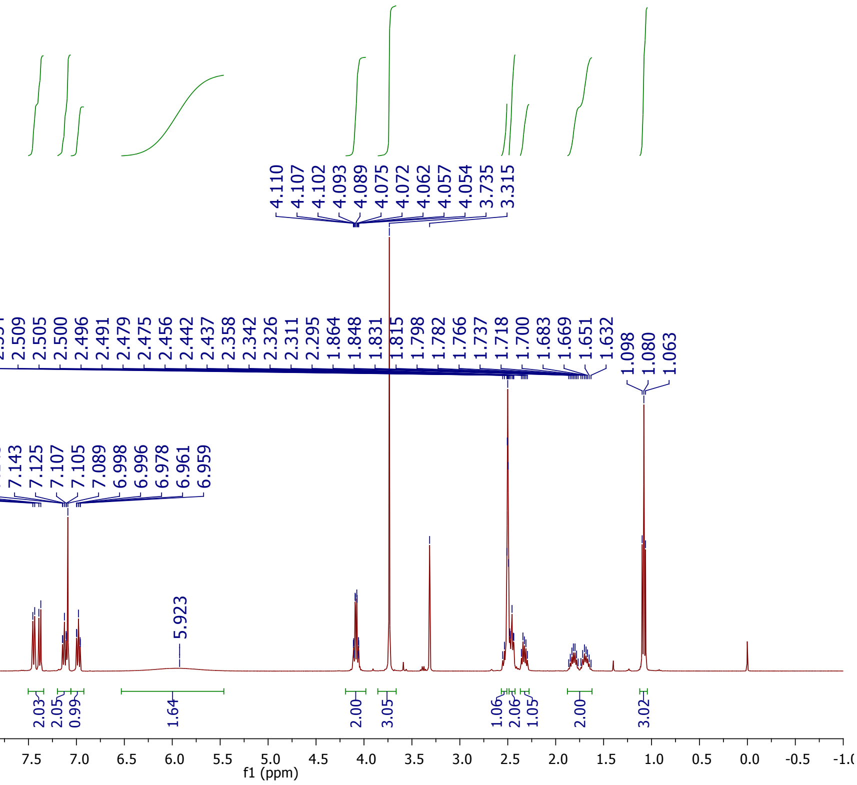

^{13}C NMR of 4f (100 MHz, DMSO-d_6

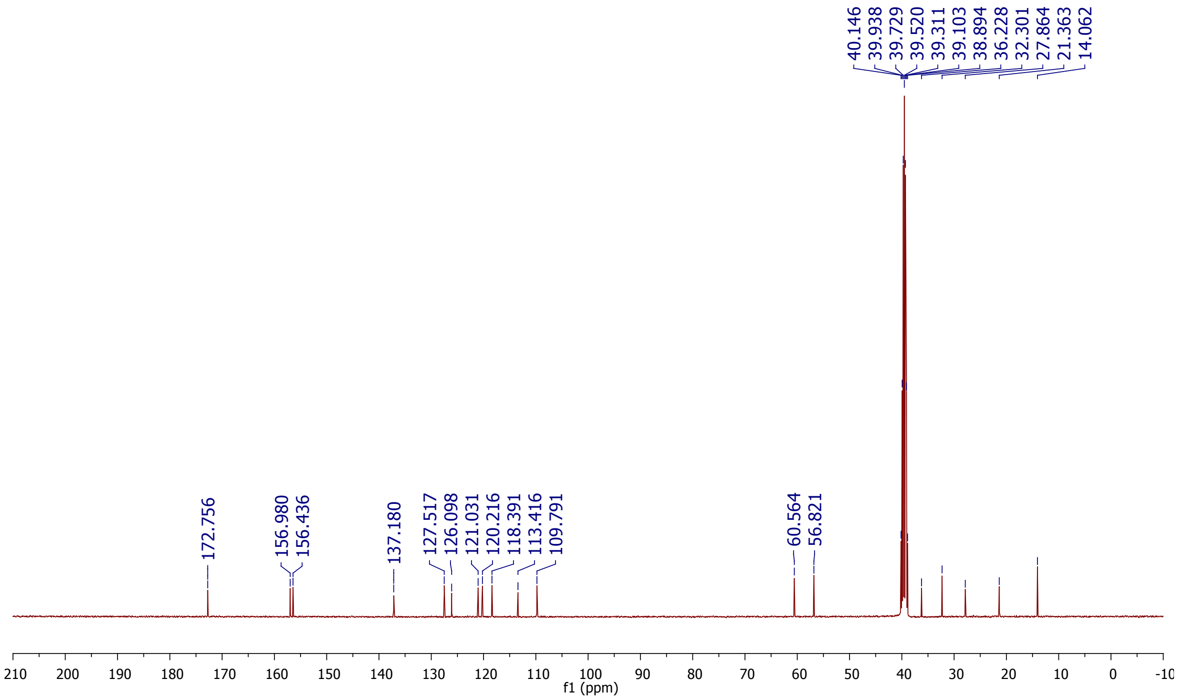

^{1}H NMR of 6a (400 MHz, DMSO-d_6

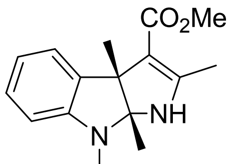

6a

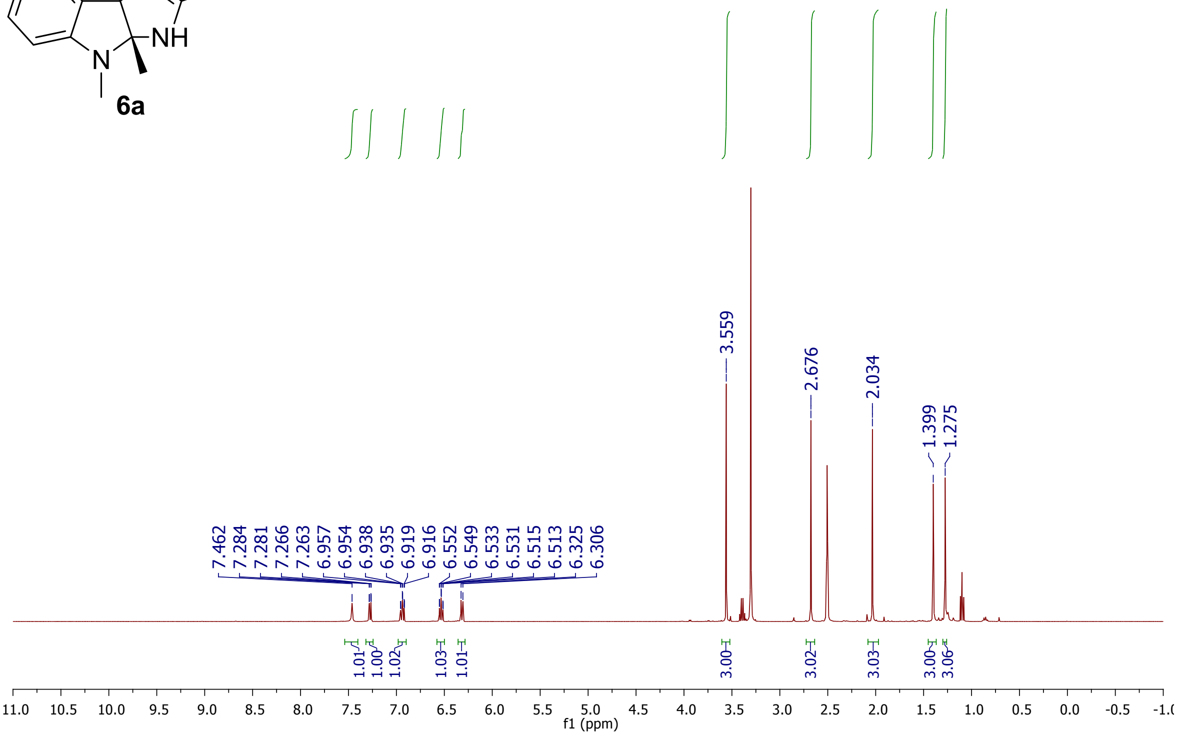

^{13}C NMR of 6a (100 MHz, DMSO-d_6

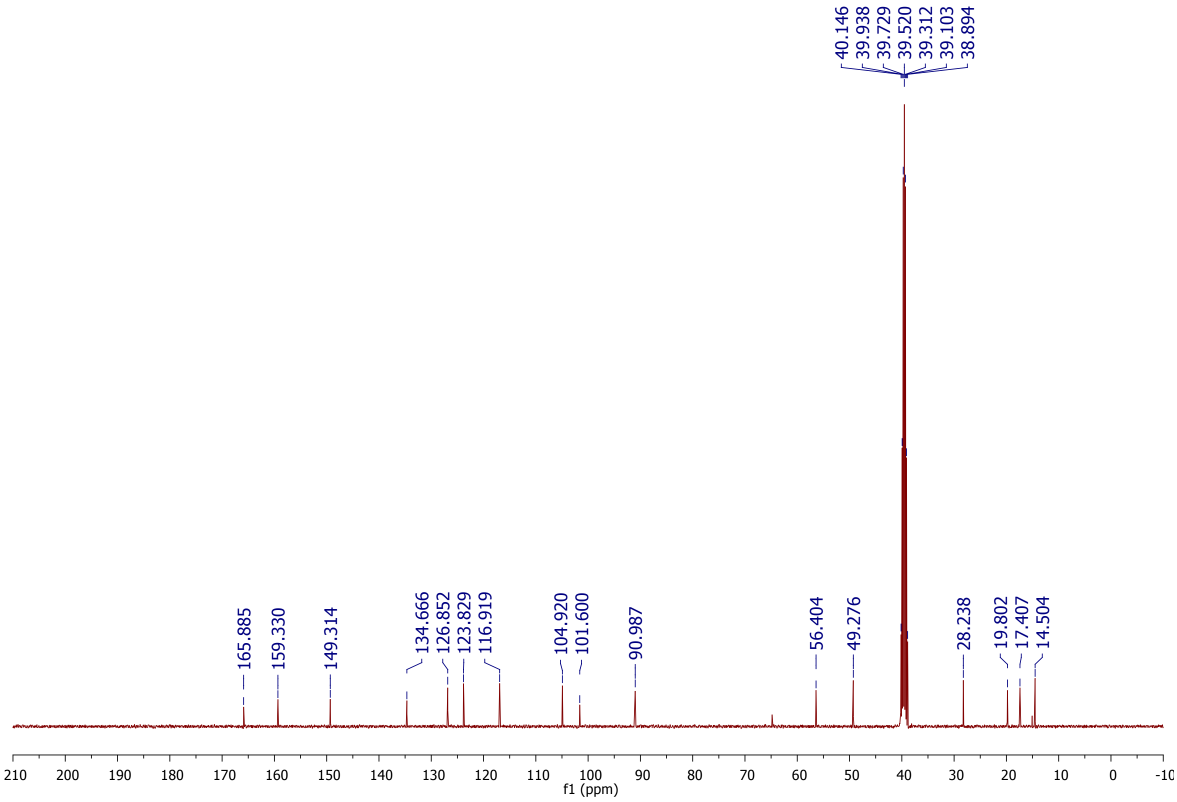

HMQC NMR of 6a (400 MHz, DMSO-d_6)

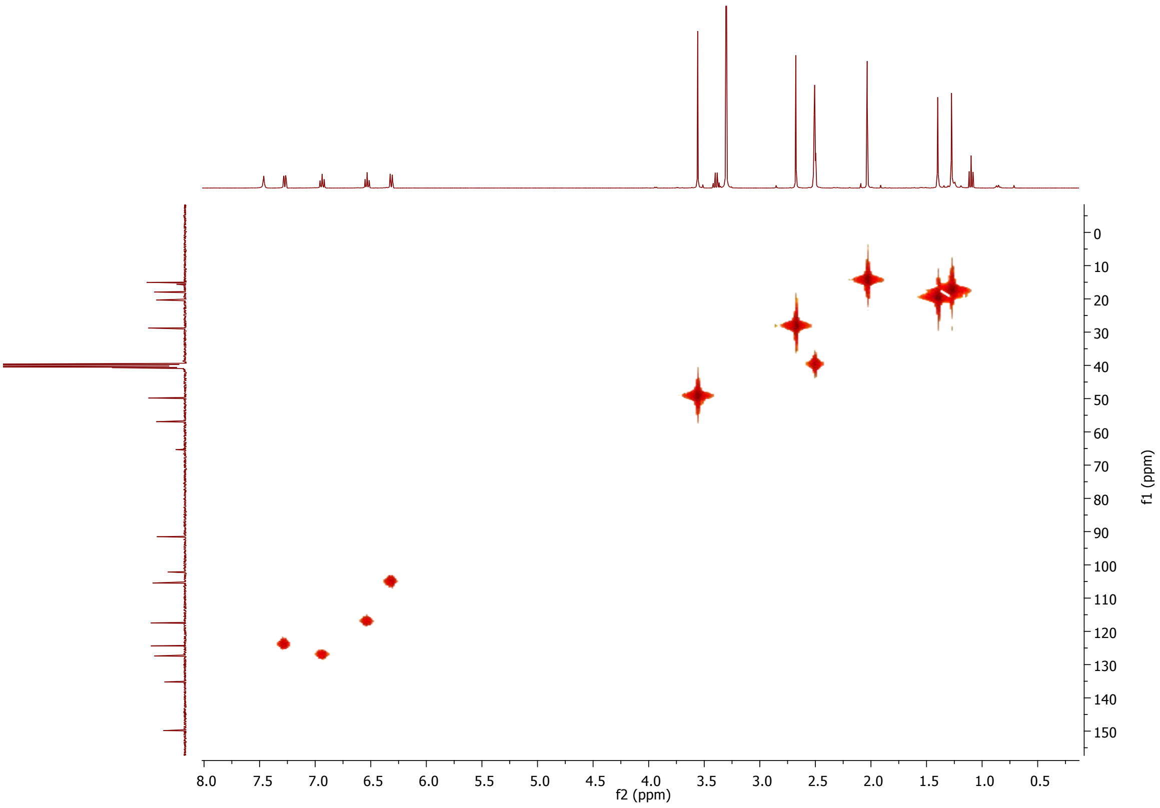

HMBC NMR of 6a (400 MHz, DMSO-d_6)

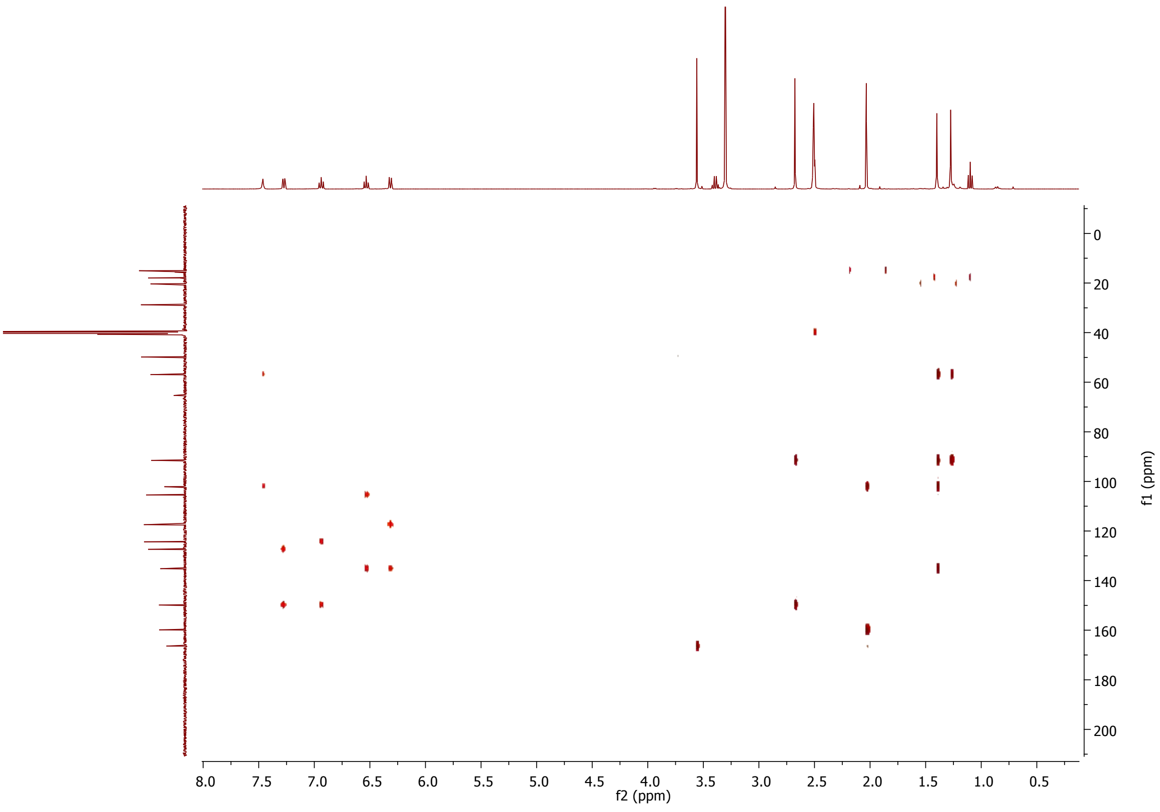

Supplement: Supplementary file 3 — jo0c01489_si_003.pdf [file jo0c01489_si_003.pdf]
